# Tick borne relapsing fever - a systematic review and analysis of the literature

**DOI:** 10.1371/journal.pntd.0010212

**Published:** 2022-02-16

**Authors:** Ákos Jakab, Pascal Kahlig, Esther Kuenzli, Andreas Neumayr

**Affiliations:** 1 Swiss Tropical and Public Health Institute, Basel, Switzerland; 2 University of Basel, Basel, Switzerland; 3 Department of Public Health and Tropical Medicine, College of Public Health, Medical and Veterinary Sciences, James Cook University, Queensland, Australia; UAMS, UNITED STATES

## Abstract

Tick borne relapsing fever (TBRF) is a zoonosis caused by various *Borrelia* species transmitted to humans by both soft-bodied and (more recently recognized) hard-bodied ticks. In recent years, molecular diagnostic techniques have allowed to extend our knowledge on the global epidemiological picture of this neglected disease. Nevertheless, due to the patchy occurrence of the disease and the lack of large clinical studies, the knowledge on several clinical aspects of the disease remains limited. In order to shed light on some of these aspects, we have systematically reviewed the literature on TBRF and summarized the existing data on epidemiology and clinical aspects of the disease. Publications were identified by using a predefined search strategy on electronic databases and a subsequent review of the reference lists of the obtained publications. All publications reporting patients with a confirmed diagnosis of TBRF published in English, French, Italian, German, and Hungarian were included. Maps showing the epidemiogeographic mosaic of the different TBRF *Borrelia* species were compiled and data on clinical aspects of TBRF were analysed.

The epidemiogeographic mosaic of TBRF is complex and still continues to evolve. Ticks harbouring TBRF *Borrelia* have been reported worldwide, with the exception of Antarctica and Australia. Although only molecular diagnostic methods allow for species identification, microscopy remains the diagnostic gold standard in most clinical settings. The most suggestive symptom in TBRF is the eponymous relapsing fever (present in 100% of the cases). Thrombocytopenia is the most suggestive laboratory finding in TBRF. Neurological complications are frequent in TBRF. Treatment is with beta-lactams, tetracyclines or macrolids. The risk of Jarisch-Herxheimer reaction (JHR) appears to be lower in TBRF (19.3%) compared to louse-borne relapsing fever (LBRF) (55.8%). The overall case fatality rate of TBRF (6.5%) and LBRF (4–10.2%) appears to not differ. Unlike LBRF, where perinatal fatalities are primarily attributable to abortion, TBRF-related perinatal fatalities appear to primarily affect newborns.

## Author summary

Tick-borne relapsing fever (TBRF) is a bacterial disease characterized by eponymous recurrent fever episodes. The disease is common on all continents except Australia and Antarctica and is caused by several species of *Borrelia* bacteria. The *Borrelia* bacteria causing relapsing fever circulate naturally between ticks and various animal hosts (usually small rodents). Humans become infected when they are accidentally bitten by an infected tick.

Although the disease has been known since 1904, many aspects of the disease have never been investigated in larger studies and are therefore still not conclusively understood. To shed light on some of these aspects, we reviewed the published literature on TBRF and analysed all reported data on the geographic distribution of the different TBRF-causing *Borrelia* spp. as well as on the clinical presentations of the disease, its complications, its diagnosis and treatment and its outcome, and compiled them in this review.

## Introduction

Two febrile illnesses related to human pathogenic spirochetes belonging to the genera *Borrelia* present as “relapsing fevers”, louse-borne relapsing fever (LBRF) and tick-borne relapsing fever (TBRF). While LBRF is an anthroponotic disease exclusively caused by *Borrelia recurrentis*, TBRF is a zoonotic disease caused by various *Borrelia* species. In different regions of the world different *Borrelia* spp. have been identified to be endemic. They differ in their natural enzootic cycles (involving different tick species and their hosts, mostly small rodents) and they are capable of infecting humans as accidental dead-end hosts [1]. An exemption is *B*. *duttonii*, for which humans may be the reservoir [2]. Since LBRF and TBRF present clinically identical and LBRF- and TBRF-*Borrelia* are microscopically indistinguishable, differentiation between the two diseases was historically limited to the epidemiological circumstances (LBRF: outbreaks, epidemics, occurrence in vulnerable populations exposed to body lice; TBRF: sporadic cases in persons exposed to ticks). Finally, the advent of molecular diagnostic techniques not only enabled to distinguish TBRF from LBRF, but also significantly changed the understanding of the diversity and epidemiology of TBRF. To summarize the current knowledge on epidemiology and clinical relevant aspects of TBRF we reviewed and analysed the existing literature, analogously to our recently published review on LBRF [3,4].

### Epidemiology

Historically, in most regions of the world TBRF has always been overshadowed by LBRF which was more prominent and epidemiologically relevant because of its epidemic occurrence. With the decline of lice-infested populations in most regions of the world, LBRF became a rare disease, while TBRF received increasing attention, especially in recent years.

**Fig 1:**
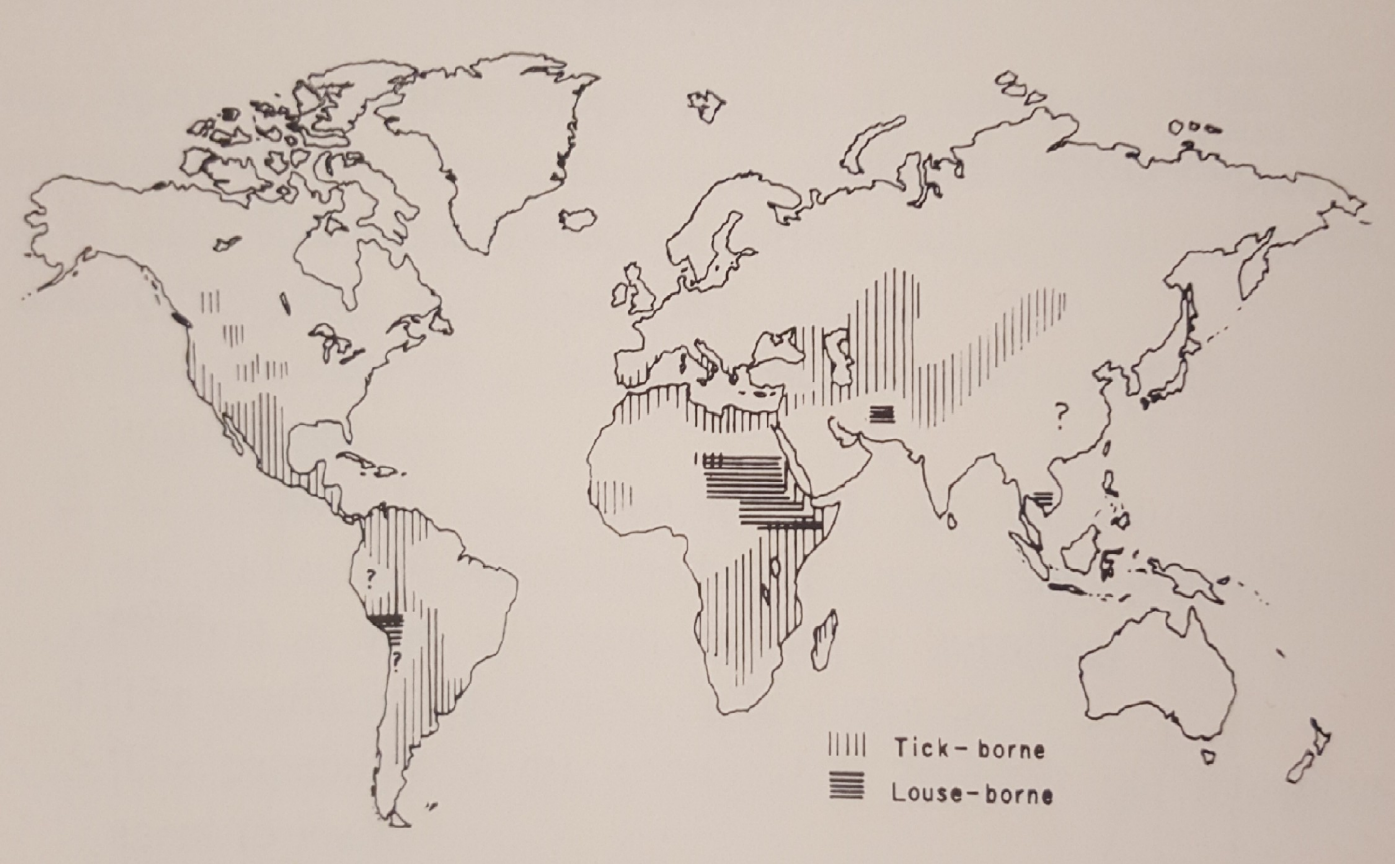
Assumed global distribution of TBRF and LBRF, 1950–1969 (Felsenfeld O. *Borrelia*; Strains, Vectors, Human and Animal Borreliosis. St. Louis: Warren H. Green; 1971[8]).

TBRF has been recognized in Africa since 1904, owing to the researches of Ross, Dutton and others [5]. In the early 1920s, TBRF was also recognized as an endemic disease in the United States of America (USA), although a tick vector was not recognized until 1930 [6]. In the following years, case reports of TBRF showed the extend of endemic areas in the USA and various tick species were identified as vectors [7]. Fig 1 shows a map with the assumed global distribution of TBRF and LBRF published in 1971 [8].

Today, TBRF is reported from all continents except Australia and Antarctica [9] and constitutes an important public health problem in some parts of the world. In Western Africa, TBRF accounts for about 13% of febrile illnesses [10] and in endemic regions of East Africa, TBRF is one of the diseases with the highest lethality among children [11].

### Tick vectors

Historically, TBRF was considered to be exclusively transmitted by soft ticks (*Ornithodoros* spp.) [8]. In 2011, this paradigm changed when *Borrelia miyamotoi*, a *Borrelia* species discovered in Japan in 1995 [12], was reported to cause TBRF transmitted by hard ixodid ticks in Russia [4], a finding later confirmed in Europe, Japan and the USA [13–15]. Nevertheless, since most TBRF *Borrelia* are transmitted by soft ticks, several distinct and epidemiological relevant differences between soft and hard ticks deserve to be highlighted. Soft ticks differ from hard ticks not only by the eponymous lack of a hard shell around the mouthparts, but also by the fact that they do not wait on leaves or blades of grass for their prey to walk by. Instead, they live in close proximity to their small mammal hosts (e.g. mice, rats, squirrels, rabbits) and rarely leave the confines of their hosts’ nest or burrow. Humans may be targeted by these night active ticks when sleeping close to their habitats. Because soft ticks feed rapidly (15–90 minutes) and then return to the place from which they came, their attack is rarely noticed. Persistent infection of the ticks’ salivary glands [16] allows quick transmission of TBRF *Borrelia* during the short feeding period, possibly after only 30 seconds of attachment [17]. Soft tick females lay clutches of eggs after each blood meal. This reproductive pattern is strikingly different from that of hard ticks, where adult females reproduce only once in their lifetime [18]. The life cycle stages of soft ticks include egg, larva, several successive nymphs and the adult. After hatching, all stages are obligate blood feeders and capable of transmitting *Borrelia* [18]. Once infected, ticks remain infectious for the duration of their life. Since soft ticks may live for more than 10 years and survive up to 5 years without feeding [19], they can outlive their rodent hosts and infect several cohorts of rodents over the course of their lifespan [20].

### Clinical picture

The incubation period of TBRF is 4–18 days. Thereafter, up to 12 recurrent febrile episodes occur. These fever episodes last 2–7 days and are separated by afebrile periods of up to 10 days [18,21,22]. A broad range of accompanying unspecific symptoms (e.g. headache, myalgia, chills, nausea, vomiting, arthralgia) as well as neurologic complications (e.g. meningitis, encephalitis, hemiplegia, facial palsy, radiculopathy, occasionally subarachnoid hemorrhage) may occur. They generally become more prominent after the second febrile episode [1,23,24]. The characteristic disease pattern of recurrent febrile episodes, interspersed with afebrile episodes, is attributable to the antigenic variation of different, sequentially expressed versions of the bacterium’s outer-membrane lipoprotein (vmp), allowing the bacterium to temporarily evade the host’s humoral immune response. Once the host’s immune system mounts antibodies against a specific vmp variant, a new vmp variant is expressed by the *Borrelia*, camouflaging itself, until antibodies are also generated against the new vmp variant [25]. The clinical presentation of LBRF is very similar to TBRF and the pathophysiological mechanism of the recurrent fever episodes is identical. However, the number of recurrent fever episodes is overall lower in LBRF (mostly less than 2) compared to TBRF (≥2), while the paroxysms last longer in LBRF (up to 10 days) compared to TBRF (≤7 days). The frequently observed neurological complications in TBRF are rare in LBRF, and TBRF is usually milder and lethality reportedly lower compared to LBRF [26].

### Diagnostics

**Fig 2:**
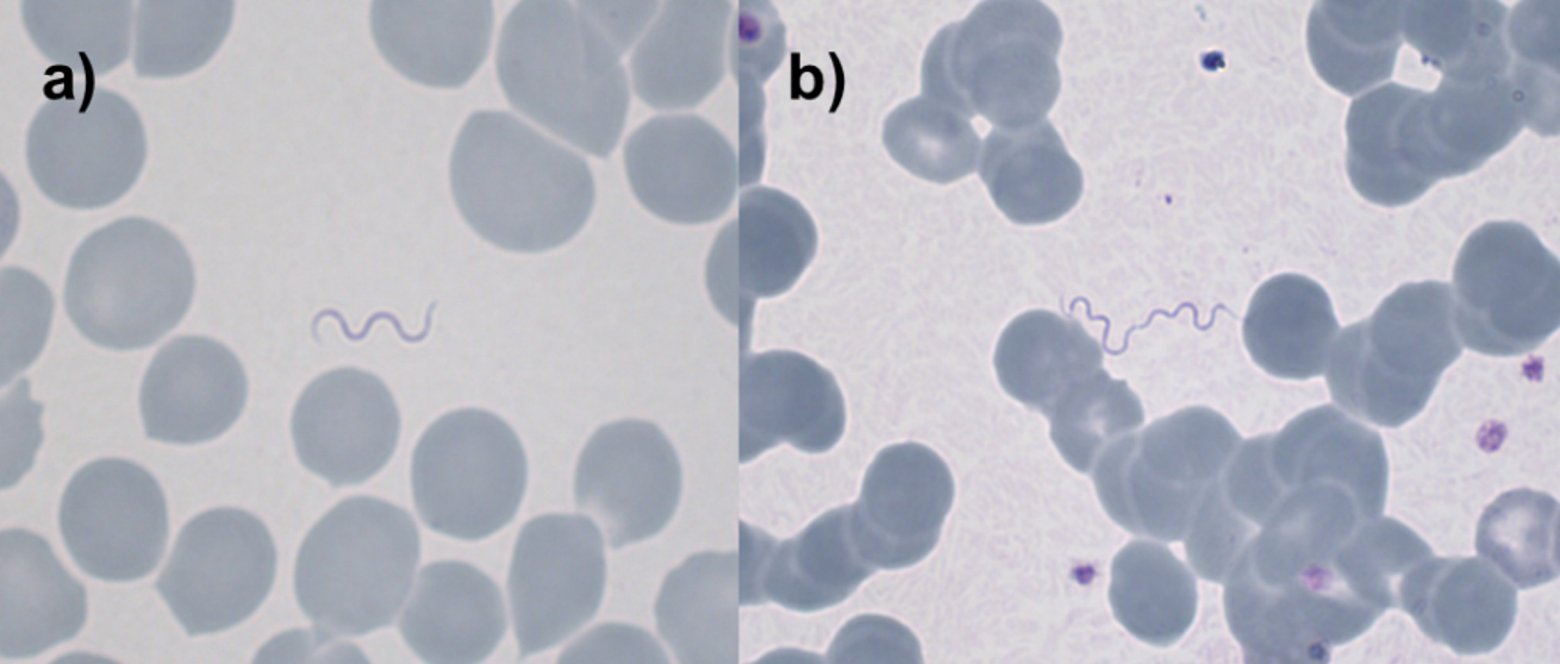
Microscopical detection of TBRF *Borrelia* in blood films. Microscopic images of Giemsa-stained thin blood films (original magnifications ×1’000) showing TBRF *Borrelia* in a patient suffering from TBRF fever due to *Borrelia persica* (courtesy of Dr. Veronika Muigg).

Relapsing fever *Borrelia* cause massive, microscopically visible bacteremia during febrile episodes. Therefore, the microscopic examination of blood smears (Fig 2) has been the diagnostic method of choice since relapsing fever *Borrelia* were first microscopically detected in the blood of patients by Obermeier in 1873 [3]. The optimum time to obtain blood is during the presence of fever, as *Borrelia* are usually not detectable once the temperature is decreasing or back to normal. Thick and thin blood films are taken and stained with e.g. Giemsa, May-Grünwald Giemsa, Wright, Wright-Giemsa, Field’s or Diff-Quick stain. Various techniques, including centrifugation of the blood samples before microscopy [27], quantitative buffy coat (QBC) preparation [28], dark-field microscopy and direct or indirect immunofluorescence [29] have been used to improve the sensitivity of microscopic detection.

No commercial serological assays have been developed to diagnose TBRF. This is due to cross-reactivity among *Borrelia* spp., including LBRF, Lyme disease and other spirochetes (e.g. *Treponema pallidum*) [30] as well as the fact that serology is not helpful to diagnose acute infection due to the time to seroconversion. Culture of *Borrelia* spp. is difficult and time-consuming and thus largely remains restricted to research institutions. Animal inoculation was considered as a putative adjunct diagnostic tool in the late 1940s [31] but, being cumbersome, was never routinely used for diagnostic reasons alone.

With the introduction of polymerase chain reaction (PCR) and sequencing techniques in the 1980s, highly sensitive and specific diagnostic tools became available. However, the availability of these molecular diagnostic tools still remains largely restricted to research institutions and microscopy remains the diagnostic gold standard for TBRF even in affluent countries [32,33]. Table 1 summarizes the advantages and disadvantages of the different diagnostic methods.

**Table 1:** Overview of laboratory methods applied in TBRF and their advantages, disadvantages and use.

Method	Advantage	Disadvantage	Use
**PCR**	Species specific; high sensitivity allows to differentiate TBRF- from LBRF-*Borrelia* and among TBRF-*Borrelia*	Currently no standardized protocol available; availability in resource-poor countries limited	Largely restricted to research institutions
**Microscopy**	Fast; widely available	Variable sensitivity (spirochete density, inter-observer variability, methodological differences); does not allow species differentiation	Diagnostic gold standard
**Culture**	Isolation and growth of *Borrelia* spp.	Time and resource demanding; overall challenging	Largely restricted to research institutions
**Animal inoculation**	Enhanced sensitivity in cases with negative microscopy; allows differentation between TBRF and LBRF*	Time and resource demanding	Historical research method; formerly also used to "transport" *Borrelia*
**Serology**	Allows retrospective evaluation of infection	Not useful as acute diagnostic method due to delayed seroconversion; cross-reactivity with other non-RF *Borrelia*	Restricted to epidemiological studies

LBRF, louse borne relapsing fever; PCR, polymerase chain reaction; TBRF, tick borne relapsing fever; RF: relapsing fever.

* Note: rodents are susceptible to TBRF *Borrelia* spp. but not susceptible to *B*. *recurrentis* infection.

(Table adapted from [3])

### Molecular epidemiology

Historically, TBRF *Borrelia* species are geographically grouped into Old World species (e.g. *Borrelia duttonii*, *B*. *persica*, *B*. *hispanica*, *B*. *crocidurae* a.o.) and New World species (e.g. *B*. *hermsii*, *B*. *turicatae*, *B*. *parkeri* a.o.). For some *Borrelia* species (identified in vectors and animal hosts only) their humanpathogenic potential still remains to be determined (e.g. *B*. *cachapoal*, *B*. *osphepa* a.o. [34,35]).

With the advent of PCR and sequencing techniques, it became not only possible to differentiate TBRF from LBRF, but these techniques also allowed genetic characterization of the different TBRF *Borrelia* species. Currently, 12 different *Borrelia* spp. and an additional 4 proposed "*Candidatus*" spp. have been reported to cause TBRF. The *Candidatus* status is used for newly discovered species for which more than a mere nucleic acid sequence is available but for which characteristics required for description according to the *International Code of Nomenclature of Bacteria* are lacking [36,37]. In general, to confirm the novelty of a bacterial species, 16S rRNA gene sequencing is performed and the sequence is compared to archived reference sequences. A threshold of 98.7% of 16S rRNA gene sequence similarity with the phylogenetically closest species with standing in the nomenclature was suggested by Stackebrandt and Ebers to classify a new bacterial species [38]. With the increasing availability of molecular diagnostic techniques, the number of reported species is likely to continue expanding in the future.

### Treatment, Jarisch-Herxheimer reaction (JHR) and outcome

In the first half of the 20th century, arsenicals and emetine bismuth iodide were the only available drugs for the treatment of relapsing fever [39]. After the discovery of penicillin, treatment shifted towards this antimicrobial agent in the second half of the century with alternative therapeutic agents becoming available over time (i.e. tetracyclines, macrolides). Today, the preferred antibiotics to treat TBRF are tetracyclines, β-lactams and macrolids [40] to which *Borrelia* are invariably susceptible [41].

Treatment may be complicated by Jarisch-Herxheimer reaction (JHR), which mostly occurs after administering the first dose of the antibiotic. JHR is characterized by intense chills and a rise in temperature about 1–2 hours after initiating antibiotic treatment and may be complicated by hypotension. JHR shares pathophysiological features of a classic endotoxin reaction mediated by proinflammatory cytokines (tumor necrosis factor α [TNF-α], interleukin 6 (IL-6), IL-8) [42]. JHR is not restricted to relapsing fever, but may also occur when treating other spirochete infections like syphilis, leptospirosis, and Lyme disease. In TBRF, JHR is reported to occur in up to 54.1% of cases [43]. Symptoms usually resolve within a few hours. Although JHR is rarely fatal, it is a clinically relevant complication which may require appropriate clinical therapeutic measures [4].

The lethality of untreated TBRF is reported to be 2–10% [44]. With antibiotic treatment the lethality is reported to be <2% [45]. Of note, TBRF is more serious in expatriates and visitors to an endemic area compared to indigenous people, who have usually been exposed to the pathogen previously [26].

TBRF infection during pregnancy is associated with an increased risk of death in pregnant women [46,47]. Infections during pregnancy are claimed to cause up to 10–15% of neonatal deaths worldwide and a perinatal lethality of up to 43.6% has been reported [1,23,44,48–50].

The aim of this study is to review and analyse the existing literature on TBRF and to summarize the epidemiological, clinical, diagnostic and treatment aspects of the disease, including its transmission through ticks, its vector reservoir and its clinical outcome.

## Methods

We performed a systematic literature search of the databases Biosis Citation Index, Biosis Previews, CINAHL, Cochrane, Current Contents Connect, Data Citation Index, Derwent Innovations Index, EMBASE Elsevier, EMBASE Ovid, Inspec, Medline, PMC, PubMed, SciELO Citation Index, Scopus, Web of Science, and Zoological Record on 04/Dec/2020, using the search term ("tick" OR "ticks" OR "tick borne" OR "*Ornithodoros*" OR "*Borrelia*" OR "*Borrelia miyamotoi*" OR "*Borrelia turicatae*" OR "*Borrelia hermsii*" OR "*Borrelia parkeri*" OR "*Borrelia persica*" OR "*Borrelia hispanica*" OR "*Borrelia crocidurae*" OR "*Borrelia duttonii*" OR "*Borrelia caucasica*" OR "*Borrelia microti*" OR "*Borrelia brasiliensis*" OR "*Borrelia mazzottii*" OR "*Borrelia venezuelensis*" OR "*Borrelia graingeri*" OR "*Borrelia latyschewii*" OR "*Borrelia dugesii*" OR "*Borrelia* infections" OR "*Borrelia*") AND ("relapsing fever" OR "recurrent fever" OR "relapsing fever disease") adapted to the search format of the different databases.

A detailed description of the literature search is available in S2 Text. After removing duplicates by EndNote (Version X9.2, Clarivate Analytics) and manually, the publications were pre-screened by title and abstract, removing those not concerning TBRF or not including the objectives of this study (epidemiology, transmission, vector, clinic, diagnostic, treatment, outcome). A full-text review of the remaining publications was then performed excluding those not meeting the inclusion criteria, according to the systematic review protocol (concerning TBRF and the objectives of the study, published in English, German, French, Italian or Hungarian), as shown in S1 Text. Publications that could neither be retrieved through the respective journals, nor by contacting libraries, or after contacting the authors, were classified as ‘not retrievable’ and excluded. During the full-text review, the reference lists of the articles were screened for additional relevant publications not identified previously («snowball-search» strategy). From the finally identified eligible studies, the following data were extracted: author, title, year of publication, type of study, study location, study period, location of acquisition/infection of *Borrelia*, *Borrelia* species, tick species, vector, percentage of ticks or vectors infected with *Borrelia*, hospital location for diagnosis, diagnostic method (microscopy, serology, molecular diagnostic, animal inoculation), grade of diagnostic certainty, number of patients, age of patient(s) (median and range), gender, symptoms, number of fever relapses, pregnancies, complications, used drug(s) and treatment regimen(s), number of treated or untreated patients, lethality of treated or untreated patients, frequency of JHR. To minimize bias, the same reviewer conducted a second full data extraction one month after the first extraction. Discrepancies and unclear cases were resolved by consulting a second reviewer. The probability of a correctly diagnosed TBRF was graded according to the diagnostic method used in the different studies, with PCR having the highest (grade A) and serology the lowest (grade C) evidence for a correct diagnosis (Table 2).

**Table 2:** Diagnostic grading system to judge the certainty of the correct diagnosis of TBRF.

Diagnostic method	Grade of diagnostic certainty	Case classification	Comment
**PCR**	A	Confirmed diagnosis	Highest level of evidence, detection even at low level of spirochetemia
**Microscopy**	B	Microscopic diagnosis	High level of evidence, easy to carry out, examiner-dependent, likelihood of detection depends on level of spirochetemia
**Culture**	B	Microscopic diagnosis	High level of evidence, difficult to carry out, time demanding
**Animal inoculation**	B	Microscopic diagnosis	High level of evidence, difficult to carry out, time demanding
**Serology**	C	Indirect evidence	Intermediate level of evidence, not standardized, cross-reaction with other *Borrelia* (e.g. Lyme disease) possible

PCR, polymerase chain reaction.

The data extraction sheet is available in S1 Table.

To visualize the worldwide distribution of TBRF cases, the causative TBRF *Borrelia* spp. and the transmitting tick species, we used the free online geographic application *Mapchart* (www.mapchart.net).

## Results

**Fig 3:**
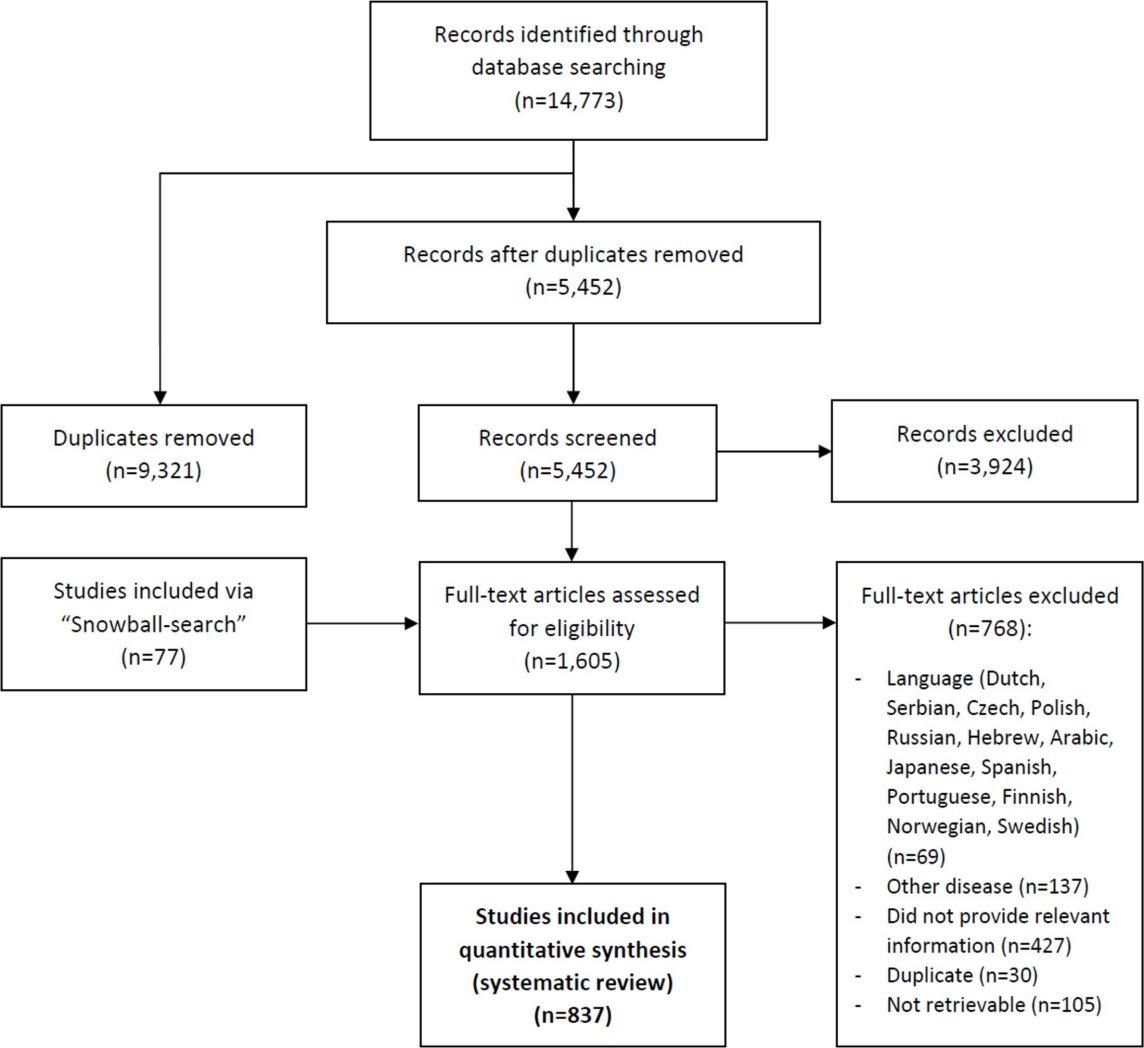
Flow diagram of search and selection of eligible publications.

Our search identified 14,773 publications, of which 837 proved to be eligible for inclusion in the review (Fig 3). The reference list of the included and excluded publications and the PRISMA Checklist for systematic reviews are available in S3 Text and S1 PRISMA Checklist.

**Fig 4:**
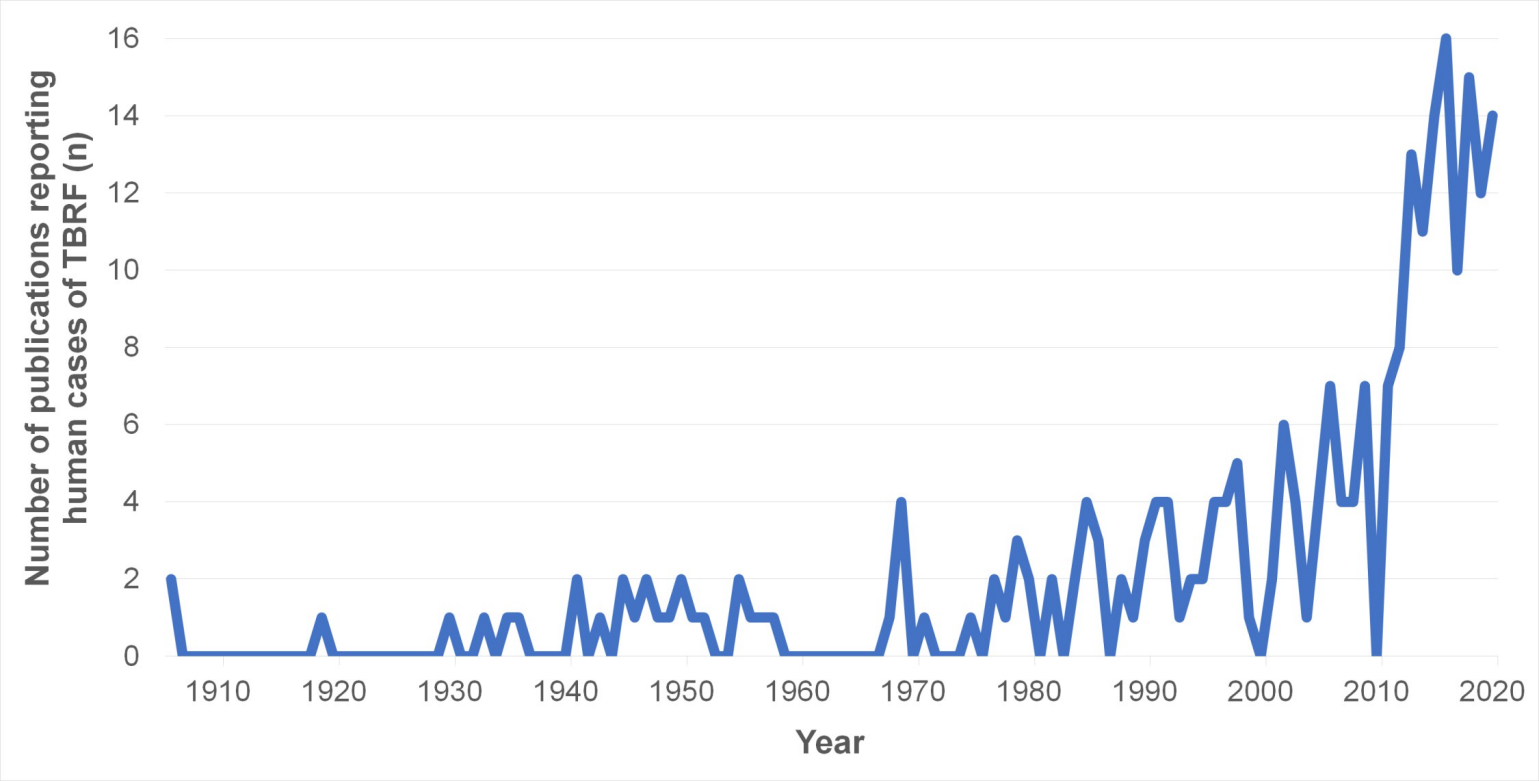
Number of TBRF case studies published from 1906 to 2020. TBRF, tick borne relapsing fever.

Fig 4 shows the number of TBRF case studies published from 1906 to 2020.

### Geographic distribution of human TBRF cases and worldwide prevalence of TBRF-transmitting ticks and *Borrelia* species

**Fig 5:**
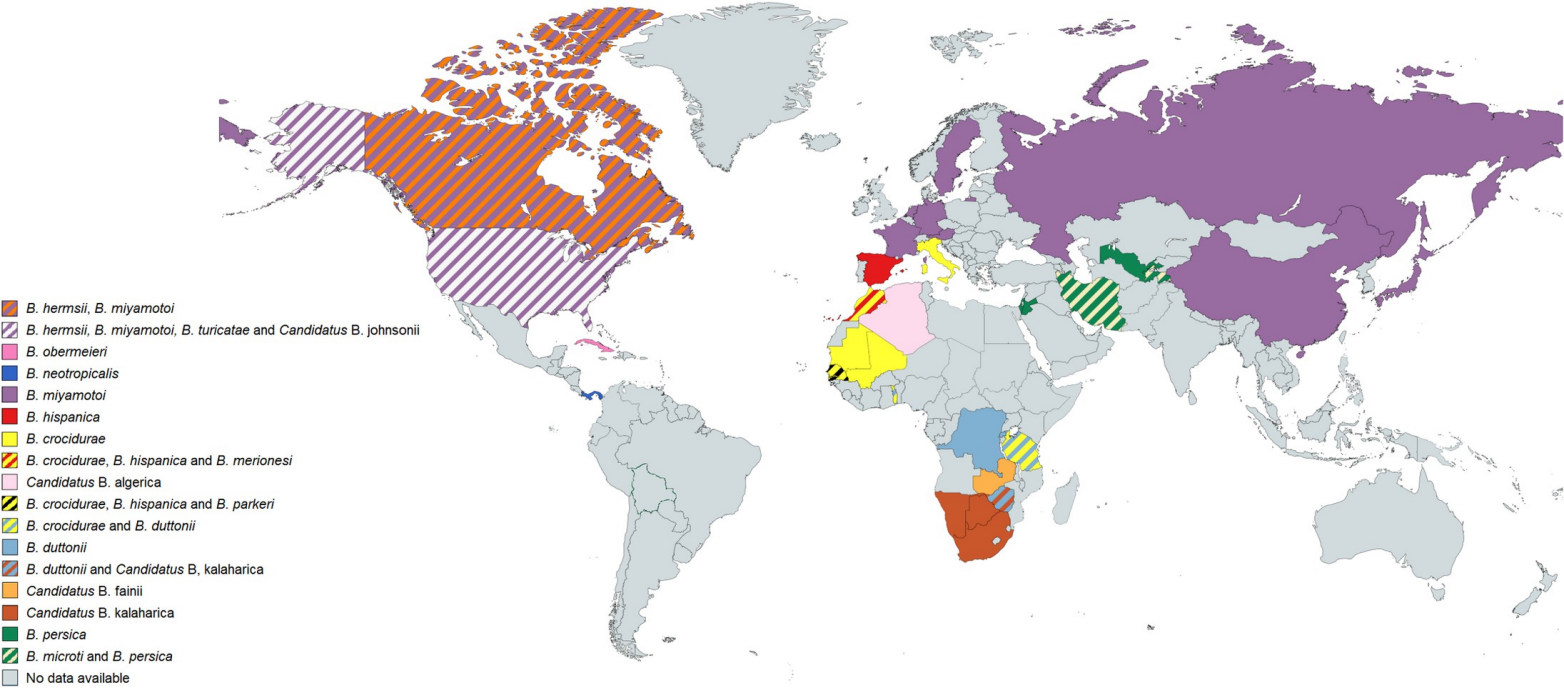
Reported TBRF cases by country and causative *Borrelia* species. B., *Borrelia*. Map created on www.mapchart.net.

**Fig 6:**
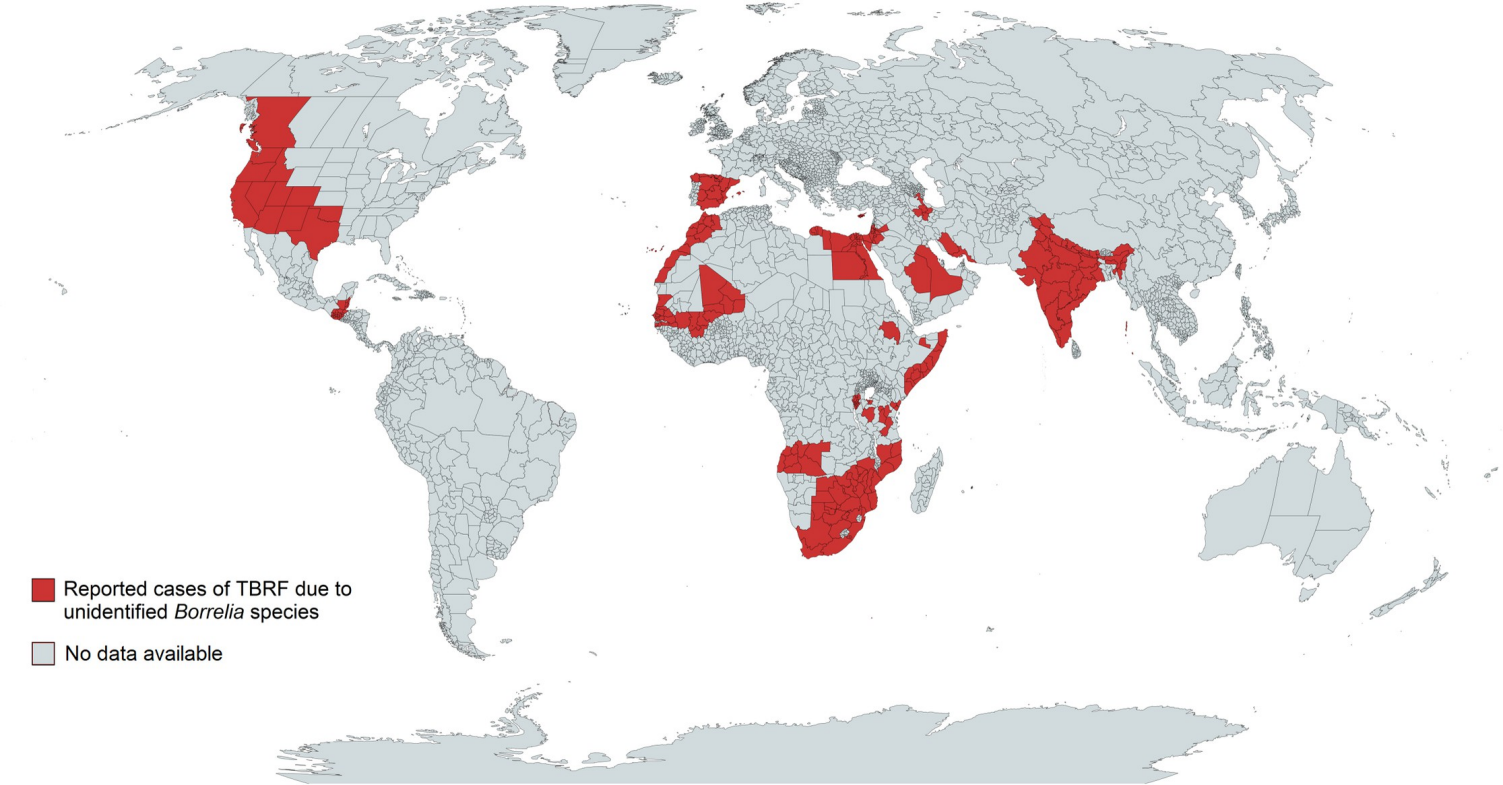
Reported TBRF cases caused by unidentified *Borrelia* species. TBRF, Tick borne relapsing fever. Map created on www.mapchart.net.

**Fig 7:**
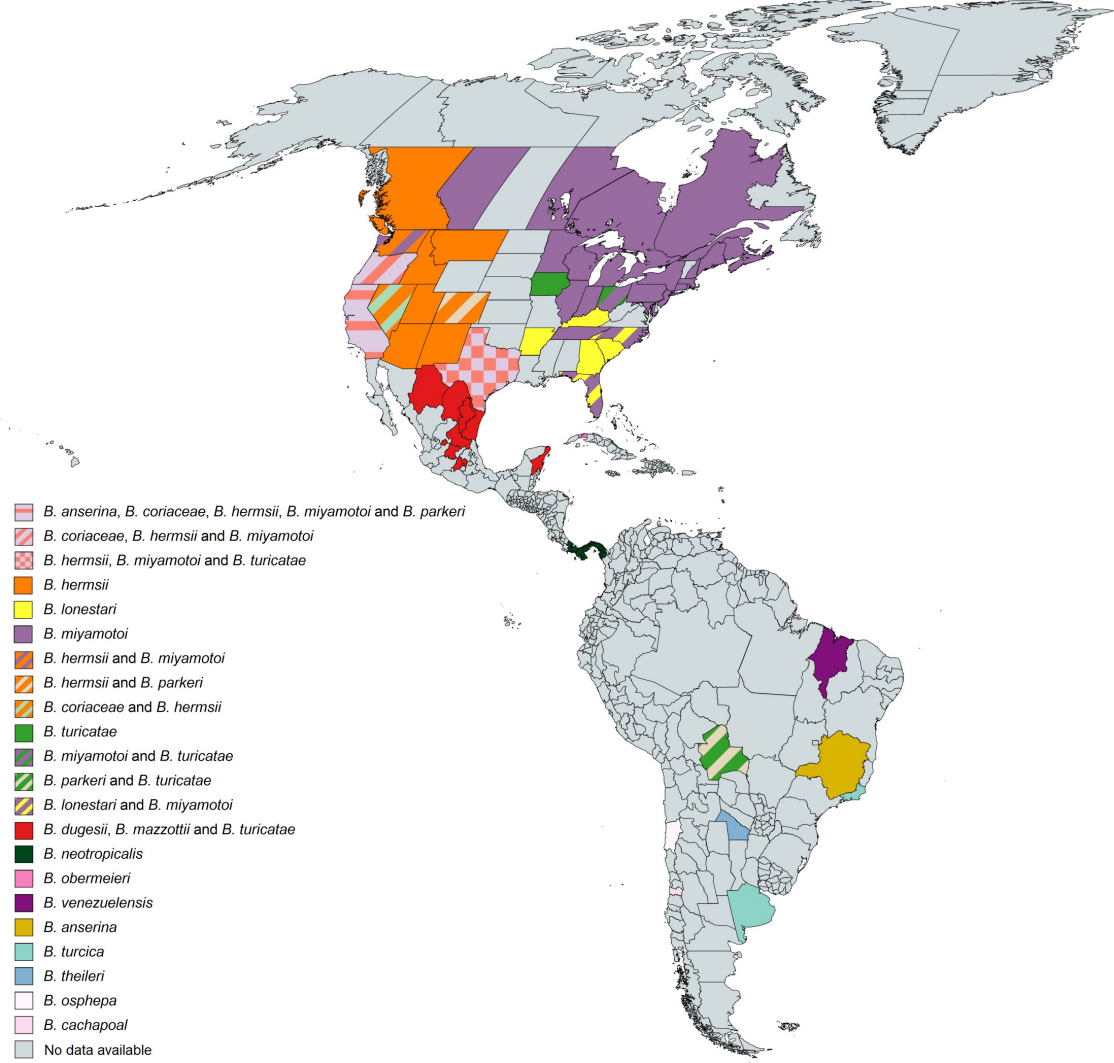
Reported presence of TBRF *Borrelia* species in ticks and animal hosts in America. B., *Borrelia*. Map created on www.mapchart.net.

**Fig 8:**
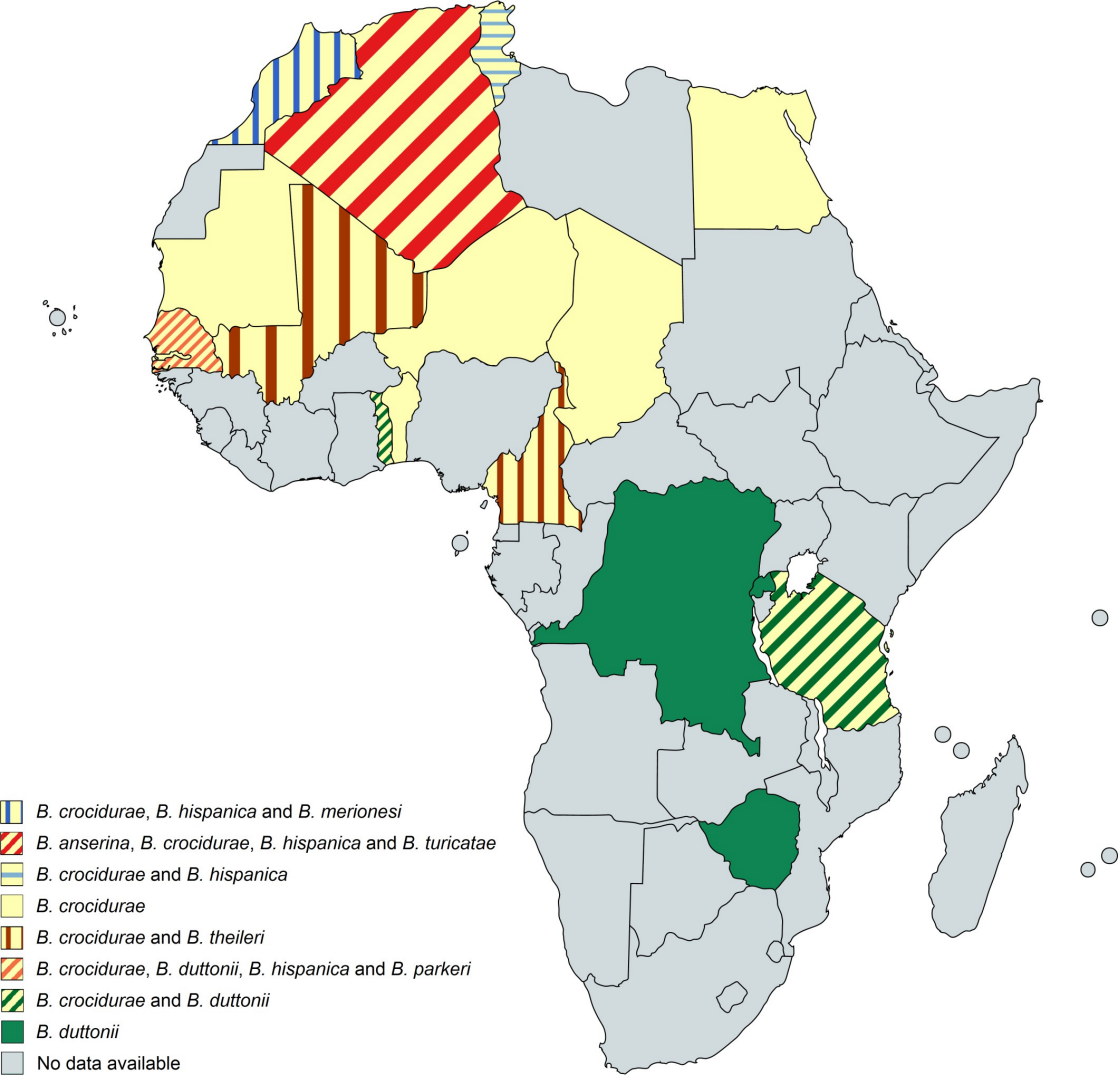
Reported presence of TBRF *Borrelia* species in ticks and animal hosts in Africa. B., *Borrelia*. Map created on www.mapchart.net.

**Fig 9:**
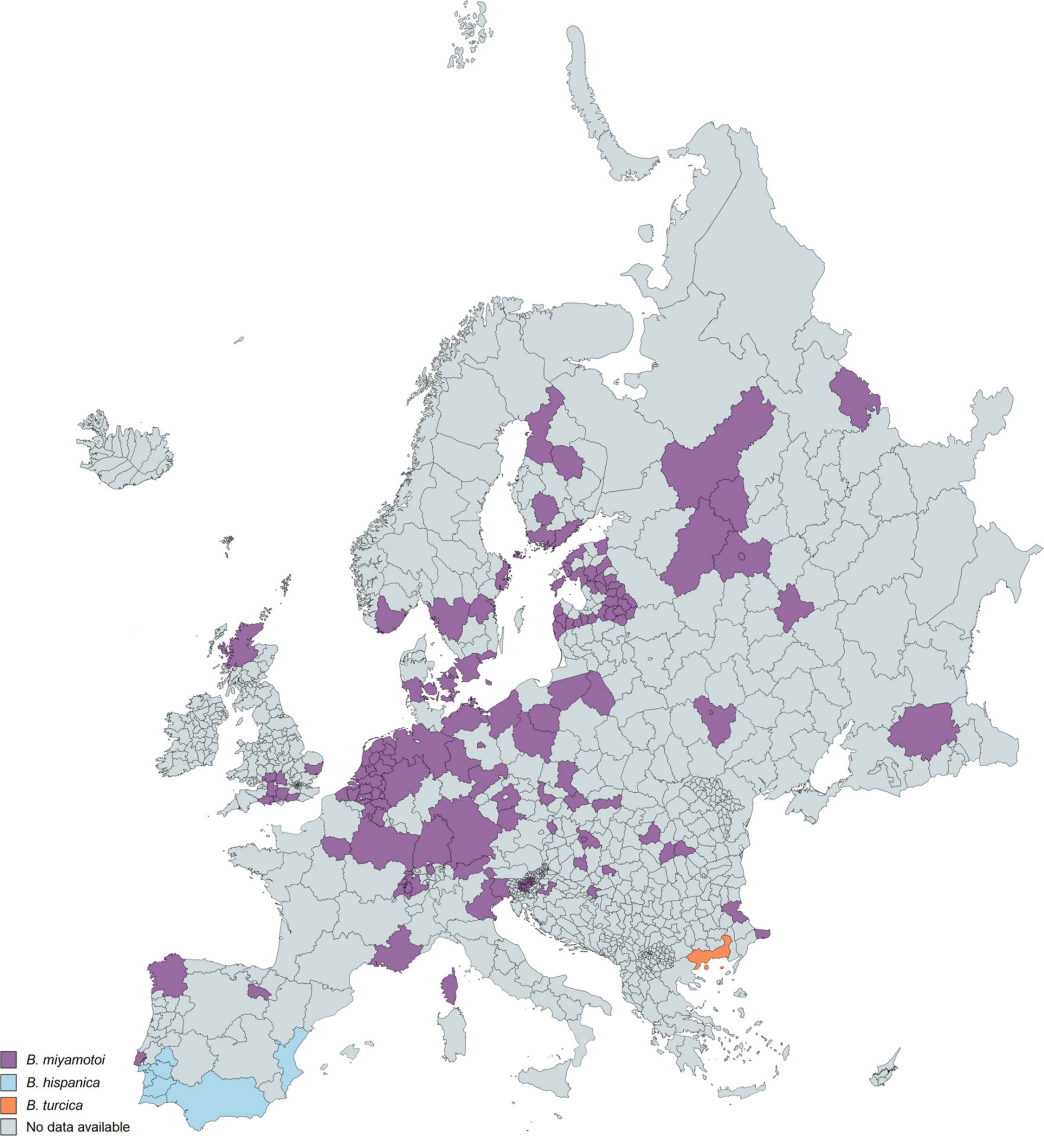
Reported presence of TBRF *Borrelia* species in ticks and animal hosts in Europe. B., *Borrelia*. Map created on www.mapchart.net.

**Fig 10:**
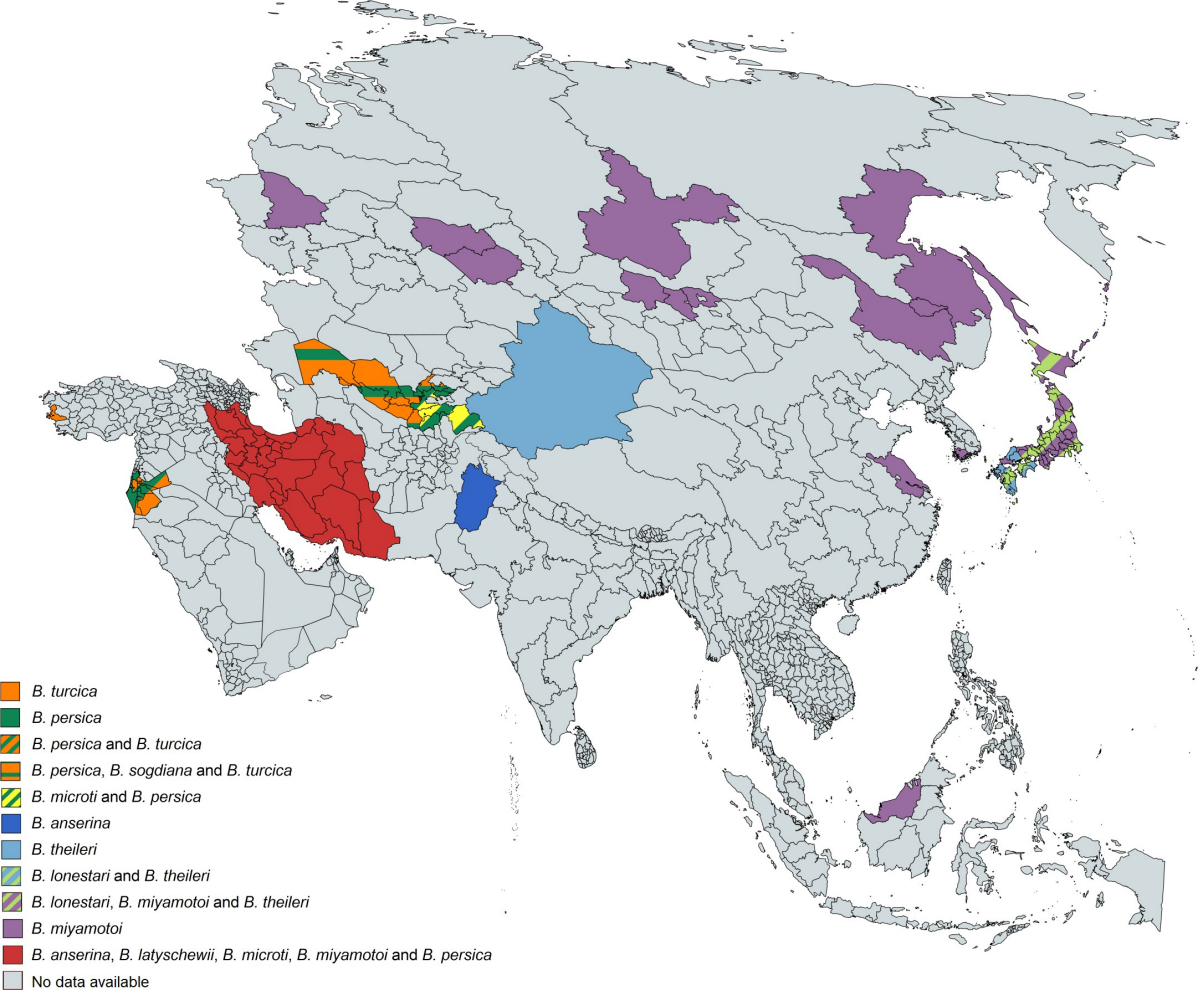
Reported presence of TBRF *Borrelia* species in ticks and animal hosts in Asia. B., *Borrelia*. Map created on www.mapchart.net.

385 of the 837 analysed studies reported the distribution of either ticks, *Borrelia* spp. or both. Some of the reported *Borrelia* are not yet acknowledged as official species and are currently considered “*Candidatus*” species. In North America, four *Borrelia* species (*B*. *miyamotoi*, *B*. *hermsii*, *B*. *turicatae*, *Candidatus* B. johnsonii) causing TBRF in humans are reported [15,43,51–104], in Central and South America four species (*B*. *obermeieri*, *B*. *neotropicalis*, *B*. *turicatae*, *B*. *parkeri*) [105–107], in Africa eight species (*B*. *crocidurae*, *B*. *hispanica*, *B*. *merionesi*, *B*. *parkeri*, *B*. *duttonii*, *Candidatus* B. algerica, *Candidatus* B. fainii, *Candidatus* B. kalaharica) [10,50,108–141], in Europe three species (*B*. *miyamotoi*, *B*. *hispanica*, *B*. *crocidurae*) [13,142–154] and in Asia three species (*B*. *miyamotoi*, *B*. *persica*, *B*. *microti*) [14,155–176]. No cases of TBRF or ticks known to transmit TBRF *Borrelia* are reported in Australia. The detailed list of *Borrelia* and ticks reported in the different continents and regions can be found in S1 Data. The worldwide distribution of reported TBRF cases by country and the causative *Borrelia* species are shown in Fig 5. The worldwide distribution of reported TBRF cases caused by unidentified *Borrelia* species is shown in Fig 6. The prevalence of *Borrelia* species causing TBRF in America, Africa, Europe, and Asia (based on detection in animal blood samples and/or ticks) is shown in Figs 7, 8, 9 and 10, respectively. The prevalence of competent vector ticks for TBRF *Borrelia* in America, Africa, Europe, and Asia can be found in S1 Fig.

### Known and putative TBRF spp. and their animal host(s) and transmitting tick species

124 studies reported data on *Borrelia* spp. and their associated animal hosts and transmitting ticks. Table 3 lists the known humanpathogenic TBRF *Borrelia* spp. as well as *Borrelia* spp. with yet unknown humanpathogenic potential, their known animal hosts and transmitting tick species.

**Table 3:** Known and putative TBRF *Borrelia* spp. and their animal host(s) and transmitting tick species.

*Borrelia* spp. causing TBRF (number of reported human cases with unequivocal species identification*)	**Animal host(s)**	**Transmitting tick species**
***B*. *crocidurae* (425)**	Rodents, shrews	*O*. *erraticus*, *O*. *sonrai*
***B*. *duttonii* (141)**	Chicken, pigs	*O*. *moubata*, *O*. *porcinus*
***B*. *hermsii* (616)**	Chipmunks, deer, dogs, owls, rodents, squirrels	*O*. *hermsii*
***B*. *hispanica* (128)**	Cats, cattle, dogs, hedgehogs, pigs, rodents, sheep, warblers	*O*. *erraticus*, *O*. *marocanus*, *O*. *occidentalis*
***B*. *merionesi***	?	?
***B*. *microti* (1)**	Hedgehogs, rodents, toads	*O*. *erraticus*
***B*. *miyamotoi* (639)**	Birds, cats, cattle, deer, dogs, hedgehogs, ponies, rodents, sheep, squirrels, wild boar	*Am*. *americanum*, *D*. *reticulatus*, *D*. *variabilis*, *Ha*. *concinna*, *Ha*. *inermis*, *Ha*. *longicornis*, *Ha*. *punctata*, *I*. *dentatus*, *I*. *hexagonus*, *I*. *nipponensis*, *I*. *pacificus*, *I*. *pavlovskyi*, *I*. *persulcatus*, *I*. *ricinus*, *I*. *scapularis*
***B*. *neotropicalis* (106)**	?	?
***B*. *obermeieri* (1)**	?	?
***B*. *parkeri***	Horses	*O*. *parkeri*
***B*. *persica* (415)**	Camel, cat, cattle, dog, hyrax	*O*. *tholozani*
***B*. *turicatae* (4)**	Birds, coyotes, dogs, foxes, rats, tortoises	*C*. *capensis*, *C*. *kelleyi*, *O*. *turicata*
***Candidatus* B. algerica (1)**	?	?
***Candidatus* B. fainii (1)**	Rodents	?
***Candidatus* B. johnsonii (1)**	Bats	*C*. *kelleyi*
***Candidatus* B. kalaharica (2)**	?	*O*. *savigni*
***Borrelia* spp. with yet unknown human-pathogenic potential**	**Animal host(s)**	**Transmitting tick species**
***B*. *anserina***	Birds	*Ar*. *minatus*, *Ar*. *persicus*
***B*. *baltazardii***	?	?
***B*. *brasiliensis***	?	*O*. *brasiliensis*
***B*. *caucasica***	?	?
***B*. *coriaceae***	Deer	*O*. *coriaceus*
***B*. *dugesii***	Rats	?
***B*. *graingeri***	?	*O*. *graingeri*
***B*. *latyschewii***	Birds	*O*. *tartakovskyi*
***B*. *lonestari***	Birds, deer, dogs	*Am*. *americanum*, *C*. *capensis*
***B*. *lonestari-like***	Deer	*Ha*. spp.
***B*. *mazzottii***	Rats	*O*. *talaje*
***B*. *osphepa***	?	*O*. *spheniscus*
***B*. *sogdiana***	Rodents	*O*. *papillipes*
***B*. *theileri***	Bats, deer	*Rh*. *geigyi*
***B*. *turcica***	Birds, camels, cattle, tortoises	*Am*. *aureolatum*, *Am*. *longirostre*, *Hy*. *aegyptium*
***B*. *venezuelensis***	?	*O*. *rudis*
***Candidatus* B. mvumi**	?	*O*. *porcinus*
***Candidatus* B. texasensis**	Coyotes	*D*. *variabilis*
**Unidentified *Borrelia* spp.**	Bats, buffalos, cats, cattle, chipmunks, deer, dogs, lizards, penguins, rabbits, rodents, sheep, shrews, snakes, tortoises, turtles, wild boar	Multiple tick species

Am., *Amblyomma*; Ar., *Argas*; B., *Borrelia*; C., *Carios*; D., *Dermacentor*; Ha, *Haemaphysialis*; Hy., *Hyalomma*; I., *Ixodes*; O., *Ornithodoros*; Rh., *Rhipicephalus*; spp., species (plural);?, unknown.

*In total, we found 9,372 reported cases of TBRF in the literature. The table contains only the unequivocally attributable (PCR confirmed) number of cases caused by the respective *Borrelia* species.

### TBRF case studies

228 (27.2%) of the 837 analysed publications reported a total of 9,372 human TBRF cases. For 5,755 cases, the patients’ gender was reported: 3,164 (55.0%) were male, 2,591 (45.0%) were female. For 2,775 cases, the patient’s age was reported: the median age of male patients was 32.7 years (range <1–90), the median age of female patients was 34.6 years (range <1–90). Table 4 lists the countries where the infections were acquired.

**Table 4:** Number of publications on TBRF cases by country where the infections were most likely acquired (n = 240 studies).

Number of publications	Country where the TBRF cases reported in the publication acquired their infection (number of cases)
**84**	USA (1,341; 182*)
**20**	Senegal (229; 238*)
**14**	Iran (2,538), Israel (753)
**13**	Spain (267; 3*)
**12**	Tanzania (930)
**7**	Canada (55; 182*)
**6**	Morocco (131; 3*), Russia (317)
**5**	India (158; 1*)
**4**	Japan (5), Mali (3; 238*), South Africa (23; 3*), Tajikistan (2; 2*)
**3**	Botswana (3*), Cyprus (111), France (58), Mauritania (3; 238*), Namibia (1; 2*), Netherland (3), Rwanda (109), Uzbekistan (1; 2*), Jordan (237), Zimbabwe (14; 1*)
**2**	Egypt (1; 9*), Libya (4; 9*), Mexico (2), Saudi Arabia (3), Somalia (1,147)
**1**	Algeria (1), Angola (4), Austria (1), Belize (1*), Burundi (1), China (14), Cuba (1), Democratic Republic of the Congo (13), Ethiopia (262), Germany (1), Guatemala (1*), Italy (1), Kenya (49), Mozambique (1*), Nepal (1*), Palestine (4), Panama (106), Sweden (2), Togo (21), Zambia (1)

TBRF, tick borne relapsing fever; USA, United States of America.

* Number of additional cases which may have contracted TBRF in the respective country, but since the ill person visited additional countries within the presumed incubation period, the infection may have also been acquired elsewhere.

Information about travel-related TBRF cases are shown in Table 5.

**Table 5:** Case analysis on TBRF in travelers.

Year	No. cases	Infection acquired in	Imported to	*Borrelia* spp.	Complications	Ref.
1982	1	Namibia	South Africa	?	None reported	[177]
1985	1	Cyprus	England	?	None reported	[178]
1988	1	Israel	USA	?	JHR (n = 1)	[179]
1991	2	Senegal	Belgium	?	Meningoencephalitis (n = 1), JHR (n = 1)	[180]
1993	3	USA	Canada	*B*. *hermsii*	JHR (n = 1)	[67]
1995	1	Saudi Arabia	USA	?	None reported	[181]
1996	1	Nepal or India	Denmark	?	None reported	[182]
1999	2	Gambia or Senegal	Netherlands	*B*. *crocidurae*	Meningitis (n = 1)	[116]
1999	1	Senegal	Italy	?	None reported	[183]
2005	3	Spain or Morocco	France	*B*. *crocidurae*, *B*. *hispanica*	None reported	[140]
2006	1	Guatemala or Belize	Netherlands	?	None reported	[184]
2006	1	Senegal	Italy	*B*. *crocidurae*	None reported	[125]
2007	1	Mali	France	?	None reported	[185]
2008	4	Senegal	France	?	Meningoencephalitis (n = 1), JHR (n = 1)	[186]
2009	1	Senegal	France	*B*. *crocidurae*	None reported	[120]
2010	1	Senegal	Belgium	*B*. *crocidurae*	Meningoencephalitis (n = 1), JHR (n = 1)	[112]
2010	1	Uzbekistan	Japan	*B*. *persica*	None reported	[164]
2011	1	Uzbekistan or Tajikistan	France	*B*. *persica*	None reported	[175]
2009–2011	4	Senegal	France	*B*. *crocidurae*	Encephalitis (n = 2), meningitis (n = 3)	[119]
2015	1	Southern Africa	Germany	*Candidatus* B. kalaharica	None reported	[109]
2016	1	Southern Africa	Germany	*Candidatus* B. kalaharica	JHR (n = 1)	[110]
2017	1	Morocco	Belgium	*B*. *hispanica*	None reported	[137]
2017	1	USA	Japan	*B*. *miyamotoi*	None reported	[95]
2018	1	Senegal	France	*B*. *crocidurae*	None reported	[118]
2019	1	Botswana or South Africa	Netherlands	?	None reported	[187]
2019	1	Tajikistan	Switzerland	*B*. *persica*	JHR (n = 1)	[174]
2020	1	Jordan	USA	*B*. *persica*	None reported	[170]
2020	2	Mali	France	*B*. *crocidurae*	None reported	[117]
2020	1	Tajikistan	Italy	*B*. *microti*	None reported	[155]

B., *Borrelia*; JHR, Jarisch-Herxheimer reaction; Ref., reference; spp., species (plural); USA, United States of America; ?, unknown.

### Symptoms related to TBRF

**Fig 11:**
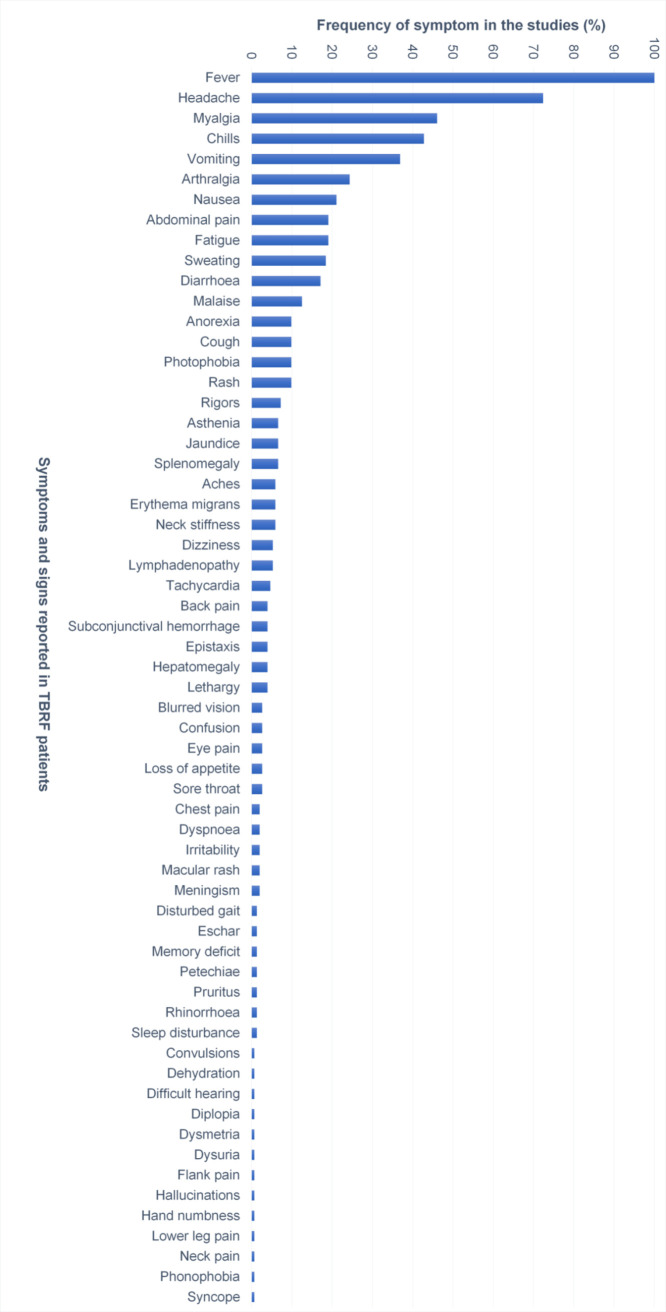
Relative frequency of signs and symptoms (in %) related to TBRF (n = 152 studies). TBRF, tick borne relapsing fever.

A total of 152 publications reported specific symptoms related to TBRF. Fig 11 shows the relative frequency of these symptoms.

**Fig 12:**
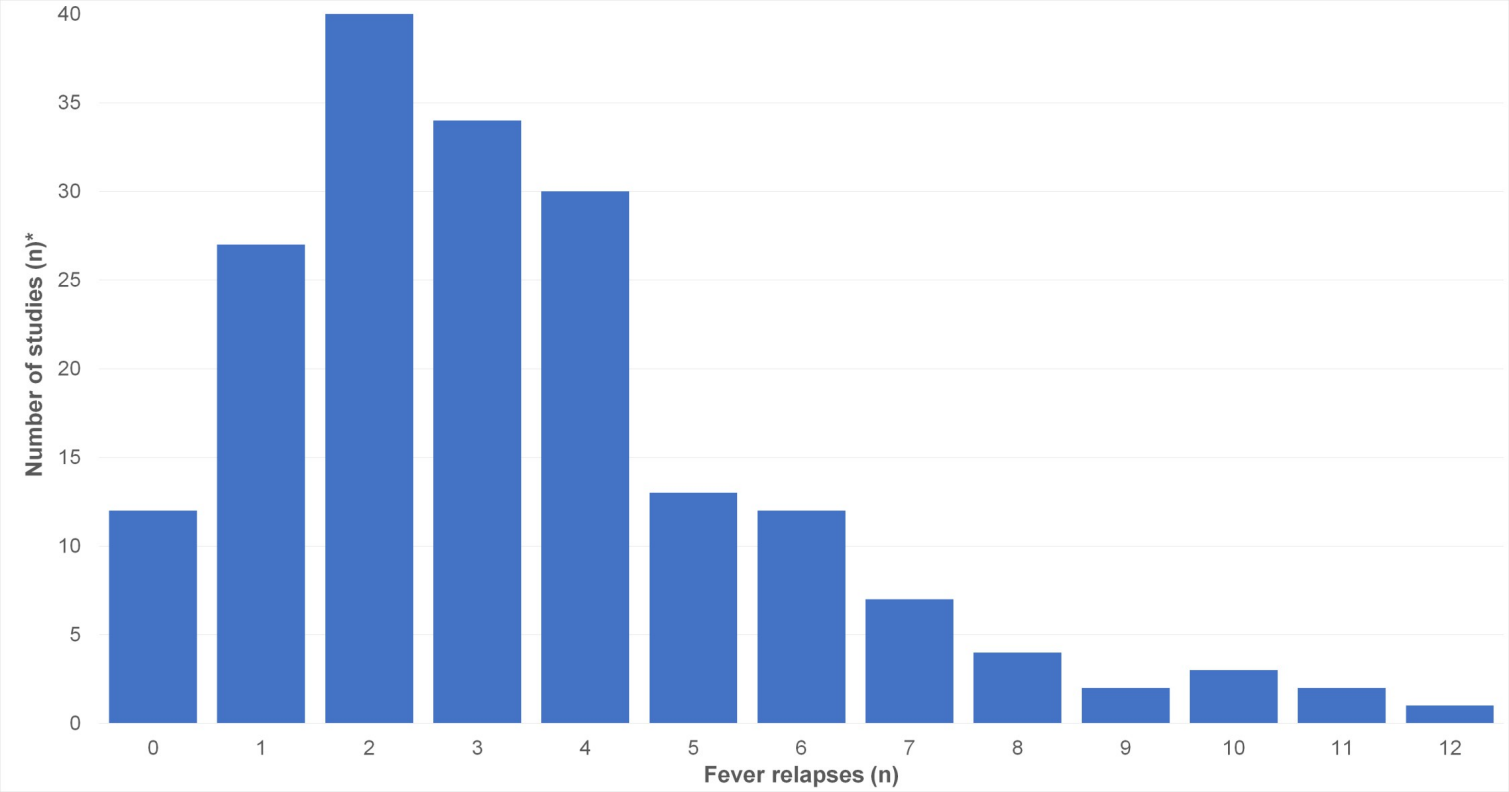
Number of relapsing fever episodes in studies on TBRF (n = 67 studies). * Note: Since the number of relapsing fever episodes within single studies was mostly reported as median, an evaluation per case was not possible.

The number of relapsing fever episodes was reported in 67 publications (Fig 12).

**Fig 13:**
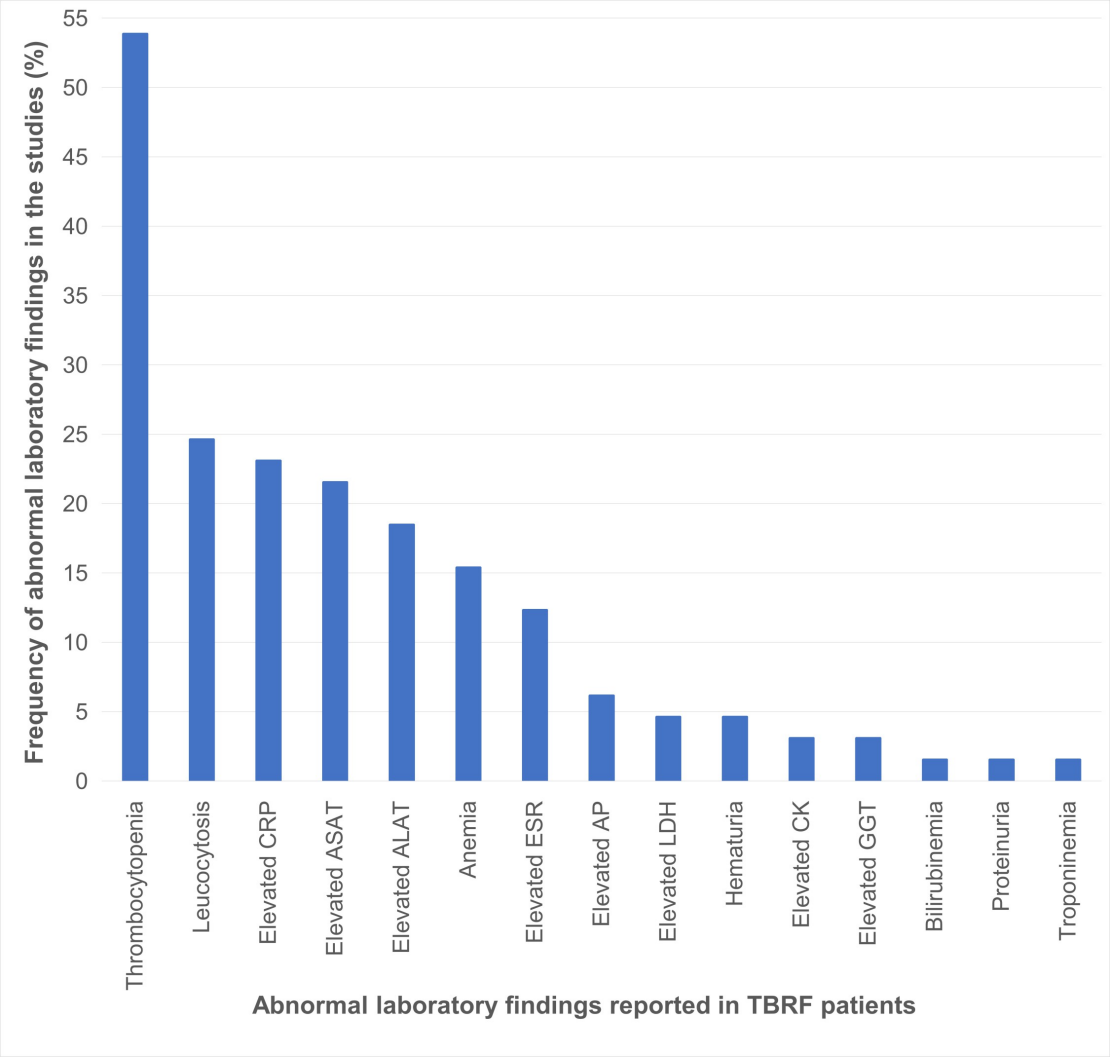
Abnormal laboratory findings related to TBRF (n = 65 studies). ALAT, alanine aminotransferase; AP, alkaline phosphatase; ASAT, aspartate transaminase; CK, creatine kinase; CRP, C-reactive protein; ESR, erythrocyte sedimentation rate; GGT, gamma-glutamyltransferase; LDH, lactate dehydrogenase; TBRF, tick borne relapsing fever.

Abnormal laboratory findings, were described in 65 studies (Fig 13).

**Fig 14:**
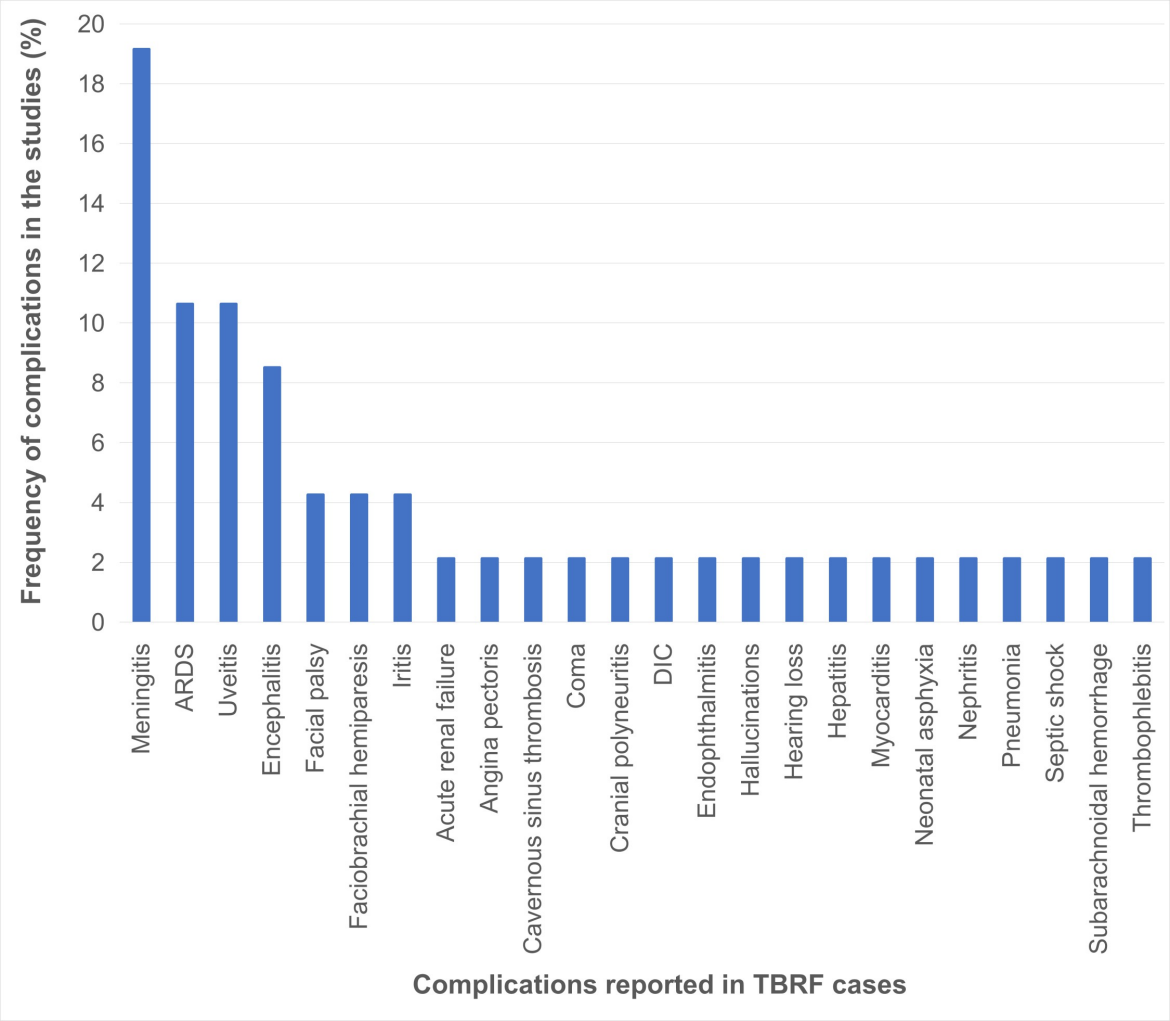
Complications of TBRF (n = 47 studies). ARDS, acute respiratory distress syndrome; DIC, disseminated intravascular coagulation; TBRF, tick borne relapsing fever.

Information on complications other than preterm delivery (61 cases), was available in 47 studies for 433 of the analysed 9,372 TBRF cases (Fig 14).

### Diagnostic

Details on the diagnostic methods used to diagnose TBRF was available for 7,612 (81.2%) of the analysed 9,372 cases (Table 6).

**Table 6:** Diagnostic methods used to diagnose TBRF in 7,612 cases (n = 240 studies).

Diagnostic method	Grade of diagnostic certainty	Number of cases in which this diagnostic method was applied	Number of cases diagnosed only by this method	Number of cases diagnosed by a combination of diagnostic methods	Number of cases where this method was the method with the highest grade of diagnostic certainty
**PCR**	A	3,443	2,051	1,392	3,443
**Microscopy**	B	5,159	2,732	2,427	3,792
**Culture**	B	129	0	129	0
**Animal inoculation**	B	756	0	756	0
**Serology**	C	1,139	377	762	377

PCR, polymerase chain reaction.

Note: in 2,452 (32%) of the 7,612 cases a combination of diagnostic tests was used to establish the diagnosis. Thus, the number of tests exceeds the number of cases.

### Treatment

**Fig 15:**
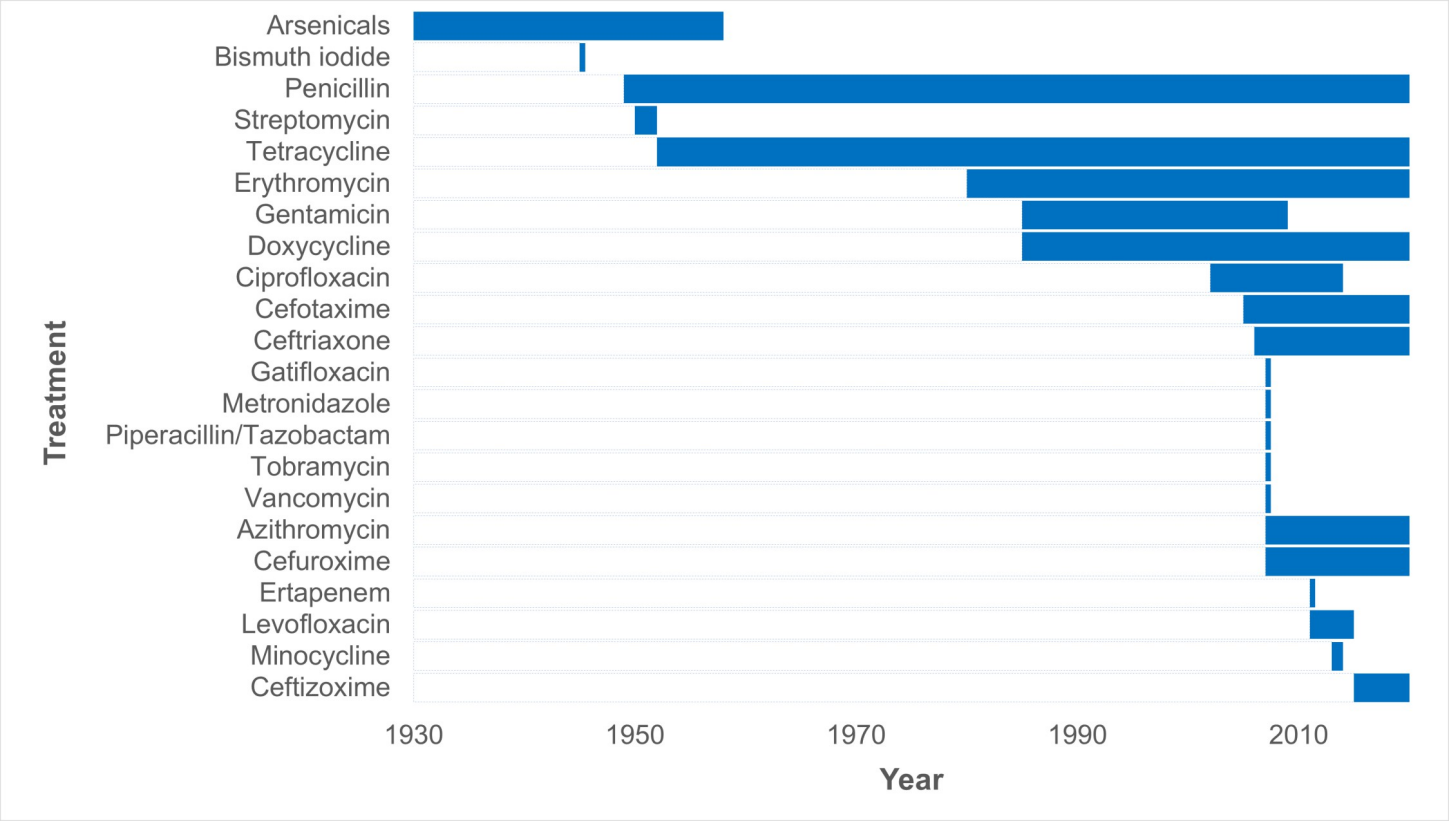
Use of different antimicrobial compounds/drugs to treat TBRF as reported from 1930 until today (n = 172 studies).

Information on antimicrobial treatment was available for 1,274 (13.6%) of the analysed 9,372 TBRF cases. 1,238 patients received antimicrobial treatment, 36 patients received no antimicrobial treatment. Fig 15 shows the use of the different antimicrobial compounds/drugs, as reported in the studies, from 1930 until today. Detailed data on the used treatment regimens (frequency, dosage, length of treatment) can be found in S2 Table. Because of the large heterogeneity and the lack of precise data, a detailed analysis of the used treatment regimens was omitted.

### JHR and outcome

Information on the occurrence of JHR was available for 1,189 (12.7%) of the 9,372 analysed TBRF cases. JHR occurred in 230 (19.3%) of antimicrobially treated patients. Data on antibiotic treatment and the occurrence/absence of JHR was reported in 65 studies (Table 7). JHR was fatal in 15 (6.5%) cases [46,133,188].

**Table 7:** Treatment specific frequency of JHR in TBRF (n = 65 studies).

Antimicrobial treatment regimen	Number of patients with reported treatment regimen and reported occurrence/absence of JHR (N)	Number of reported JHR (N)	Frequency of JHR (%)
**Tetracyclines**	116	28	24.1
***Doxycycline***	*83*	*18*	*21*.*7*
***Tetracycline***	*33*	*10*	*30*.*3*
**β-lactams**	26	4	15.4
***Penicilline***	*12*	*1*	*8*.*3*
***Ceftriaxon***	*12*	*2*	*16*.*7*
***Cefuroxim***	*2*	*1*	*50*.*0*
**Erythromycin**	13	4	30.8

JHR, Jarisch-Herxheimer-reaction.

Information on the clinical outcome was available for 1,454 (15.5%) of the analysed 9,372 TBRF cases. 95 (6.5%) of the 1,454 cases were fatal [39,46,115,133–135,188–198]. 88 fatal cases were reported from Africa (Tanzania 72, Ethiopia 12, Democratic Republic of Congo 1, Egypt 1, Rwanda 1, Senegal 1), 5 from the USA, and 2 from Israel. Table 8 lists the outcome of TBRF in different patient groups.

**Table 8:** Case fatality analysis of TBRF (n = 17 studies).

Patient group	Number of fatal cases (%)	Number of documented fatal cases among cases with documented antimicrobial treatment (N = 629)	Number of documented fatal cases among cases with unknown antimicrobial treatment status (N = 816)	Number of documented fatal cases among documented untreated cases (N = 9)
**All cases of TBRF not infected during pregnancy and not infected in the fetal or peripartal period (N = 992)**	43 (4.3%)	38	5	0
**Pregnant women (N = 231)**	11 (4.8%)	0	11	0
**Fetuses and neonates (N = 231)**				0
***Intrauterine death***	11 (4.8%)	2	9	0
***Postpartal death***	30 (13.0%)	12	18	0
**Total**	95 (6.5%)	52	43	0

CFR, case fatality rate.

## Discussion

### Publications on TBRF

Over the last 30 years, the number of published case studies on TBRF has increased significantly (Fig 4). The increasing number of publications over time might be attributable to the advent of molecular diagnostic tools as well as the increasing awareness and recognition of the disease, which previously was very likely underdiagnosed.

### Epidemiology

With the advent of molecular diagnostic techniques and the resulting identification of multiple new TBRF causing *Borrelia* spp., as well as of previously unknown epidemic areas, the historic classification of TBRF *Borrelia* into Old World and New World TBRF *Borrelia* spp. has been replaced by a more complex picture. This is particularly evident when comparing the map outlining the TBRF endemic areas as assumed by Felsenfeld five decades ago (Fig 1) with maps compiled from confirmed data (Figs 5 and 6). Interestingly, some regions of the world, like e.g. Europe or Japan, emerged as endemic regions. Contrastingly, other regions like South America or central Africa were apparently considered more endemic in the past than they actually are (Figs 5 and 6). However, rating the relevance of TBRF in a particular region of the world demands not only to integrate the mere presence of TBRF *Borrelia* and the occurrence of human cases but also needs to take into account differences in available diagnostic capacities as well as a reporting bias accruing from differences in academic publishing traditions (Table 4). It is to be expected that in the future additional endemic areas, additional *Candidatus*- as well as proven TBRF-*Borrelia* spp., additional animal host reservoirs, and additional transmitting tick species will emerge (Table 3) and further expand the granularity of our picture of TBRF.

The rather recent discovery of *B*. *miyamotoi*’s wide geographic distribution (Figs 5, 7, 9 and 10), as well the surprising finding that this *Borrelia* sp. is not only transmitted by various soft tick species [156,199–202] but also by hard ticks (*Ixodes* spp. [152,203]), which were previously considered non-vector-competent for TBRF *Borrelia*, serve as good example. The demand for an adequate ecological niche serving not only a specific animal host but also the transmitting tick species may be the main reason why TBRF is restricted to certain geographic areas. There are two ways for TBRF *Borrelia* spp. to spread geographically: either the infected host animals move into new areas with locally prevalent vector competent tick species or infected ticks, attached to overland migrating host animals or to migratory birds, translocate into new areas with suitable animal hosts and a suitable habitat allowing the ticks survival. Its potential to infect a broad range of host animals, and especially migratory birds, may be the reason why *B*. *miyamotoi* shows a wide geographic distribution all around the Northern hemisphere (Figs 5, 7, 9 and 10). Since migration by birds demands prolonged attachment of the vector tick species, the unusual vector competence of hard ticks may also play a pivotal role in the extend geographic distribution of *B*. *miyamotoi*. Unlike the rather short attachment and feeding time of soft ticks, the prolonged attachment and feeding of hard ticks would be favourable for migration over large distances [1,204]. In contrast, TBRF *Borrelia* spp. with a narrow and spatially limited host range are rather unlikely to extend their geographic range, as for instance in the case of *B*. *coriaceae*. With deer being the only associated animal host, this *Borrelia* species is to date only reported in the Western part of the USA (California, Nevada, Oregon). Whether and to what extend climatic changes will influence the epidemiology and possible spread of TBRF in the future remains to be seen [205,206]. Reports of TBRF in international travelers are rare. To date, only 42 cases have been published (Table 5). Although underdiagnosing and underreporting is likely, existing surveillance data on infectious diseases in travelers confirm the apparently overall low exposure risk and the rare occurrence of TBRF in travelers [207].

### Signs, symptoms and complications

Presenting similar to a multitude of other febrile infectious diseases without specific signs and symptoms (Fig 11), TBRF may easily be missed or misdiagnosed. Furthermore, the under- or misdiagnosis of TBRF may also be caused by its mostly benign and, even without antimicrobial treatment, self-limiting course. The sensitivity of TBRF *Borrelia* to standard antibiotics widely available and used for empirically treatment may also contribute to underdiagnosing.

Even in endemic regions, cases presenting without the diagnosis-suggestive relapsing fever episodes or without complications leading to a thorough diagnostic work-up, are likely to be missed. The most helpful symptom to differentiate TBRF from other febrile illnesses are the recurrent fever episodes, which are found in the majority of cases (Fig 12). When looking at Fig 12 it needs to be emphasized that the presented data do not reflect the natural course of TBRF as almost all reported and analysed cases received antimicrobial treatment. Thus, the number of fever relapses in untreated TBRF is very likely higher. In addition, it must be kept in mind that the number of fever relapses indirectly reflects the time elapsing before a patient seeks medical help or has access to medical care. This may differ widely across countries and health care systems. Likewise, it is very likely that in endemic areas physicians familiar with the disease will suspect, diagnose and treat TBRF much earlier compared to their colleagues in non-endemic areas.

To date, there is largely insufficient data to compare different TBRF *Borrelia* species with regard to possible differences in their clinical manifestations. We performed a subgroup analysis regarding the signs and symptoms, complications and number of fever relapses reported in TBRF caused by *B*. *hermsii* and *B*. *crocidurae* as for these the most data were available (S2 Fig). However, due to the low number of cases as well as the inhomogeneity of the available data, conclusions regarding possible differences in clinical manifestations of different TBRF *Borrelia* species remain difficult.

The only exception may be the reported rates of neurologic complications: in contrast to LBRF, neurological complications are common in TBRF [26], reported to occur in 10–40% of the cases [33,208]. Our analysis confirms the prominence of central nervous system (CNS) involvement among the reported complications of TBRF (Fig 14). A comprehensive review on neurologic and ophthalmologic involvement and complications in TBRF has been published by Cadavid and Barbour in 1998 [33]. Generalized neurologic symptoms like dizziness, apathy or delirium are considered attributable to spirochetemia and high fever rather than to direct invasion of the CNS by *Borrelia* and were reported in LBRF as well as TBRF. Neuropsychiatric abnormalities not solely attributable to high fevers have been reported for both TBRF and LBRF and cases of encephalitis or encephalopathy are occasionally observed in TBRF and LBRF [33]. Neurologic complications differ in their frequency and pathogenesis among the two diseases. While neurologic complications in LBRF are rare and primarily attributed to CNS hemorrhage and not to direct invasion of the CNS by the pathogen, neurologic complications in TBRF are frequently observed and attributed to direct invasion of the CNS invasion by *Borrelia* [33]. Neurologic manifestations are more likely to present during subsequent, rather than the initial febrile period [33]. A frequently reported complication in TBRF (and not reported in LBRF) is cranial neuritis, most often presenting in the form of facial palsy. Its frequency varies with different TBRF *Borrelia* species between 3% (7/230) of *B*. *hispanica*-related cases to 38% (8/21) of *B*. *turicatae*-related cases [33]. However, as mentioned above, data are limited, inhomogeneous, and come from times when molecular species identification was not possible. Nevertheless, data from animal studies also suggest that the different TBRF *Borrelia* species are neuroinvasive to varying degrees [33].

Reports of *B*. *miyamotoi* associated meningoencephalitis in immunocompromised patients suggests that the pathogens may behave like an opportunistic pathogen in this population [13,15,153]. Whether and to what extend *B*. *miyamotoi* may also cause neurological complications in non-immunocompromised patients is currently unknown.

Limited data suggest possible differences regarding the clinical presentation of soft tick-borne RF and hard tick-borne/*B*. *miyamotoi* RF ("Cases with the characteristic recurring febrile episodes interspersed with non-febrile intervals that typify classical RF have only been described sporadically [in *B*. *miyamotoi* RF]. … Furthermore, unlike RF spirochetes, epistaxis, abortion, jaundice and major organ failure have not appeared as features of *B*. *miyamotoi* infection" [209]), but the currently available data is limited and has yet to prove itself. Pooled clinical data, like presented in Figs 11–14, may thus not necessarily reflect the true picture on species level, but as mentioned above, the overall low number of reported cases and the inhomogeneity of the available data do not allow for robust subgroup analyses. Ocular involvement in TBRF includes iritis, cyclitis, choroiditis, and optic neuritis (Figs 11 and 14). When eye involvement is reported in TBRF, it is bilateral in one-third of the cases and almost always occurs after the third or fourth febrile episode. Involvement of the eyes during LBRF has not been reported [33].

The historical statement that the occurrence of vomiting in TBRF is exclusively related to meningitis [26] cannot be confirmed, as overall, gastrointestinal symptoms are quite common in TBRF (Fig 11).

Interestingly, Erythema migrans, a symptom highly specific for Lyme disease, has been reported in some cases of TBRF (Fig 11). Thus, it may be speculated that coinfections with other locally endemic tick-borne pathogens could lead to overlapping presentations making it difficult to attribute signs and symptoms to a specific pathogen. This speculation is strongly supported by the fact that all reported TBRF cases presenting Erythema migrans were reported from Russia, the Netherlands, and Japan and caused by *B*. *miyamotoi*, the only TBRF *Borrelia* transmitted by hard ticks and thus, by ticks plausibly capable of co-transmitting Lyme *Borrelia*. In analogy, the report of an eschar (Fig 11), a symptom primarily associated with rickettsial infections, suggests coinfection.

The frequency and relevance of such coinfections remains unclear. An outbreak of a febrile illness in West Texas was initially wrongly attributed to Lyme disease, based on a combination of facial palsy and a positive *B*. *burgdorferi* serology in some of the cases. However, in the end the disease was identified as TBRF due to *B*. *turicatae* and the serological results recognized as cross-reactivity [210].

Bleeding signs like petechiae, epistaxis, subconjunctival hemorrhage or hemorrhagic complications like subarachnoidal hemorrhage or disseminated intravascular coagulation (DIC, Fig 14) are only rarely reported in TBRF (Fig 11) when compared to LBRF, where subconjunctival hemorrhages and epistaxis are common (25%) and severe hemorrhage (hemoptysis, gastrointestinal bleeding, retinal hemorrhages) and DIC (leading to intracranial, massive gastrointestinal, pulmonary or peripartum hemorrhage) are feared complications [211].

### Laboratory findings

Like the signs and symptoms, the laboratory findings are rather unspecific. Only thrombocytopenia is a feature present with a rather high frequency, possibly helping to support the tentative diagnosis (Fig 13). However, fever and thrombocytopenia occur in a variety of infections, including malaria, a broad range of common viral infections and notably also in many other tick-borne diseases (e.g., rickettsioses, ehrlichiosis, anaplasmosis, tularemia, Q fever, babesiosis, arboviral infections).

### Diagnostic

To date, microscopy of thin and thick blood smears remains the most frequently reported diagnostic method for diagnosing TBRF (Table 6). Regarding the sensitivity of microscopy, it is important to remember that the positivity thresholds of thin and thick smear preparations are estimated at 10^5^ and 10^4^ spirochetes per mL of blood, respectively [28] and that the number of *Borrelia* in the peripheral blood is considered to be lower in TBRF compared to LBRF (an observation repeatedly quoted, but for which clear evidence is missing) [3]. To improve the sensitivity of microscopy using equipment that is easily available in small health centers, a method based on enrichment of bacteria by centrifugation followed by Giemsa staining was developed. This methods reduces the detection level to fewer than 10 spirochetes per mL of blood [27]. RF *Borrelia* are not infrequently detected in blood smears ordered because of the clinical suspicion of malaria. With the trend to progressively replace microscopy with rapid diagnostic tests (RDTs) to diagnose malaria, the incidental finding of RF *Borrelia* in malaria smears will become less, potentially further increasing the underdiagnosing of this disease.

PCR has grown in importance and is now the second most frequently reported diagnostic method. PCR is the most sensitive and specific diagnostic method available and the only diagnostic method to definitively differentiate between TBRF and LBRF (although, in most cases of microscopically detected RF *Borrelia* the epidemiological circumstances will allow to conclude whether TBRF or LBRF is the more likely diagnosis [3]) and to differentiate the different TBRF *Borrelia* species. However, because *Borrelia* are highly related at the molecular level (16S rRNA gene sequence variability ≤1%), the development of discrimination PCR assays is challenging and they will not always be able to provide species identification [128,182,212,213]. For instance, for *B*. *duttonii* and *B*. *recurrentis*, which are genetically and genomically very closely related, even PCR assays fail to provide species discrimination [214]. Nevertheless, several studies have been conducted using multiplex real-time PCR assays allowing the detection and speciation of several RF *Borrelia* (*B*. *crocidurae*, *B*. *duttonii/B*. *recurrentis*, *B*. *hispanica*) found in Africa [215]. Although the successful introduction of PCR as point-of-care routine diagnostic in rural Senegal has been reported [114], the availability of PCR still remains largely restricted in resource poor settings.

Serology plays no relevant role in diagnosing TBRF for several reasons. Within endemic areas, seroprevalence is high, which may not necessarily reflect acute infection but previous infection (seroscars). Furthermore, the time to seroconversion is too long to influence treatment decisions in acutely ill patients. Additionally, as mentioned above, cross-reactivity of assays may confuse TBRF borrelioses and Lyme borreliosis [216]. The latter issue can be circumvented by using an assay detecting antibodies to the GlpQ protein, which is produced by RF *Borrelia* species, but not by Lyme *Borrelia* species [217].

Culture of TBRF *Borrelia* is restricted to very few laboratories in the world, has primarily been used in the context of research and has no role in routine diagnostic.

### Treatment

Over time, many antibiotic compounds have been evaluated for the treatment of TBRF (Fig 15). However, as with LBRF [218], neither well-designed studies to determine the best treatment regimens nor comparative studies of the efficacy of different antimicrobial agents are available. Data evaluating putative differences between different TBRF *Borrelia* species regarding antimicrobial susceptibility or treatment response are scarce or non-existing.

Due to their successful use in patients with syphilis, the arsenic compounds arsphenamine (salvarsan; the first marketed antibiotic which cured a bacterial infection [219]) and its less toxic derivative neoarsphenamine (neosalvarsan) developed by Ehrlich and Hata [220] were the first antimicrobial compounds used to treat relapsing fever *Borrelia* infections.

In the 1940s, the considerably less toxic and more effective penicillin became available and replaced the arsenical compounds for the treatment of the spirochetal infections syphilis and RF. It quickly became apparent that unlike LBRF, for which a single administration of intramuscular procaine penicillin proved highly effective [218], TBRF required prolonged and sufficiently high-dose penicillin treatments to prevent relapse and achieve cure [221–224]. In this regard, and from the pathogen’s neurotropic persistence demonstrated in animal models, an early analogy between TBRF and syphilis was drawn [225]. This analogy, as well as the marked differences in the treatment response of LBRF and TBRF, strengthened the suggestion that sufficient antibiotic target levels in the CNS are critical to successfully treat TBRF. Due to the lack of emerging resistance in spirochetal infections, penicillin remains an option for these infections up until today. However, the often restricted availability of procaine penicillin for intramuscular injection and the need to dose intravenously administered penicillin several times per day to achieve sufficient blood and tissue levels restricts the drug’s use in clinical practice. Today, the use of β-lactams is mostly restricted to the treatment of TBRF patients with CNS involvement, similar to early CNS involvement in Lyme disease or neurosyphilis, and ceftriaxone (2g once daily for 10–14 days) is preferred over penicillin in these cases [226]. Of note, in vitro data suggesting resistance of *B*. *miyamotoi* to amoxicillin but susceptibility to ceftriaxone have been reported [227]. However, the validity and generalizability of these findings is called into question by the successful treatment of a case of *B*. *miyamotoi* TBRF with amoxicillin (and sultamicillin) [158].

In the 1950s, tetracycline was introduced and became the drug of choice for oral treatment of uncomplicated TBRF. Similar to β-lactams, a correlation between administered dose and length of treatment and relapse rate/treatment success exists [102,228]. In the absence of CNS involvement, oral or parenteral treatment with a tetracycline (tetracycline 500mg every 6 hours for 10 days [226] or doxycycline 100mg every 12 hours for 7–10 days [229]) is the recommended treatment for adults. While tetracycline remains contraindicated in children due to the risk of irreversible dental staining, the administration of doxycycline is considered safe for up to 21 days regardless of age [229–231]. The recommended pediatric dose of doxycycline is 4.4mg/kg body weight/day divided in 2 doses (max. 200mg/day) [229]. Several studies have evaluated antibiotic postexposure prophylaxis/preemptive therapy with doxycycline to prevent TBRF following tick bites within endemic areas. Studies on preemptive therapy with a short course of doxycycline (day 1: 200mg/d, day 2–5: 100mg/d) were found to be highly effective in this regard [40,232,233]. A more recent study suggests, that even a single dose of doxycycline is sufficient and as effective [234].

For patients unable to take a β-lactam or a tetracycline, erythromycin (500mg or 12.5 mg/kg body weight every 6 hours for 7–10 days) is the most widely recommended alternative [226,229]. It is likely that the better tolerated azithromycin is equally effective, but dosing data are lacking [227].

Given the lack of comparative studies on antimicrobial treatment regimens of TBRF, well-designed studies evaluating different therapeutic regimens in the different TBRF species would be desirable.

### JHR, outcome

The pathogenesis and frequency of JHR in spirochete infections has repeatedly been reviewed by several authors [42,235,236]. The reported frequency of JHR in spirochete infections varies widely (Lyme disease: 5–30%, syphilis: 8–75%, leptospirosis: 9–83%, LBRF: 0–100%, TBRF: 1–39%) [42]. However, due to the lack of a uniform definition and a standardised assessment of JHR, reliable data on the true incidence and possible differences in incidence of JHR in spirochete infections remain missing [4]. Thus, a critical appraisal of the incidence of JHR in TBRF is difficult. Nevertheless, compared to LBRF, where we found a JHR incidence rate of 55.8% [4], we found a considerably lower JHR incidence rate of 19.3% in TBRF. Considering the proposed underlying pathomechanisms, the lower incidence of JHR in TBRF may primarily be attributable to the overall lower number of *Borrelia* in the peripheral blood compared to LBRF [3,236]. Experimental animal data suggest that TBRF Borrelia species can differ in their degree of spirochaemia. This suggests that there may also be a species-specific risk of JHR. Unfortunately, the existing data are not sufficient to confirm or refute such an assumption.

The choice of antibiotics used for the treatment of spirochetal infections is considered to affect the incidence and severity of JHR, although studies in this regard provide conflicting views [237–239]. In their systematic review and meta-analysis comparing different antibiotic regimens in LBRF, Guerrier and Doherty found a benefit in favour of penicillin when comparing the rate of JHR (in 3/5 eligible studies) and concluded that treatment with a tetracycline appears to be associated with a higher rate of JHR [218]. Our analysis suggests that this may also be true in TBRF, with penicillin showing a considerably lower rate of reported JHR when compared to tetracyclines or erythromycin (Table 7). Overall, existing data suggest that in RF tetracycline treatment is associated with a higher rate of JHR but a lower relapse rate and penicillin treatment is associated with low rate of JHR but a higher relapse rate [211,218]. Overall, we found a TBRF-related CFR of 6.5%. This is in line with the generally reported TBRF-related CFR range of 2–10% [44]. The frequently encountered postulation that TBRF is less fatal than LBRF [18,240,241] may simply reflect the fact that the CFR of LBRF estimated in the literature has been too high [4]. This assumption is supported by our review on LBRF, were we found a CFR of 4% (treated)– 10.2% (untreated) [4]. Therefore, we speculate that under similar medical conditions the overall CFR of TBRF and LBRF is not significantly different. Mortality in TBRF appears to be primarily due to neurologic complications and ARDS, although reported data on attributable causes of death are largely lacking. It appears that JHR contributes little to the overall death rate, as only 6.5% of cases with JHR (from the 19.3% of antimicrobial-treated patients) die from it. In a clinical trial setting involving 184 patients with LBRF in Ethiopia, the CFR attributed to JHR was 3.3% [242]. Considering the probably above average quality of care in such clinical trial settings, CFR due to JHR in TBRF and LBRF may, overall, not be significantly different.

Regarding adverse pregnancy outcomes in TBRF, rates between 30% and 44% have been reported [192,243–246]. This is in analogy with LBRF, where adverse pregnancy outcomes, primarily in the form of abortions, are reported to occur in at least 70.9% of the cases [4]. Interestingly, our analysis suggests that the CFR for unborn children and for pregnant women does not differ from the CFR of other patients. Only the CFR of newborns appears to be considerably higher compared to non-neonatal cases (Table 8).

Table 9 comparatively summarizes the disease specific characteristics of TBRF and LBRF.

**Table 9:** Summary of characteristics of TBRF compared to LBRF.

	TBRF	LBRF [3,4]
**Causative *Borrelia* spp.**	Various *Borrelia* spp.	*B*. *recurrentis* (only)
**Epidemiology**	Occurrence of sporadic cases (affecting persons exposed to ticks)	Occurrence of outbreaks/epidemics (affecting vulnerable populations exposed to body lice)
**Number of relapsing fever episodes**	Mostly ≥2	Mostly <2
**Duration of febrile episodes**	Mostly ≤7 days	Up to 10 days
**Treatment**	Prolonged antibiotic treatment demanded (7–10 days; in the case of CNS involvement 10–14 days)	Single dose antibiotic treatment sufficient
**Complications**	Neurological complications are common (attributable to direct CNS invasion by *Borrelia*)	Neurologic complications are rare (attributable to hemorrhagic diathesis/bleeding complications rather than direct CNS invasion by *Borrelia*)
	Ocular involvement may occur	No ocular involvement reported
	Hemorrhagic diathesis/bleeding complications are rare	Subconjunctival hemorrhages and epistaxis are common.
**Risk of JHR**	19.3%	55.8%
**Overall CFR**	6.5%	4–10.2%
**Perinatal fatalities**	Primarily postpartal complications/affecting newborns	Primarly prepartal complications/affecting fetuses

TBRF, tick-borne relapsing fever; LBRF, louse-borne relapsing fever; CNS, central nervous system; JHR, Jarisch-Herxheimer reaction; CFR, case fatality rate.

Our analysis has several limitations. First, data and results of studies and case series were often reported as overall numbers, medians or percentages and thus attributing data to individual cases was not possible. Second, the heterogeneity of the reviewed studies from very different geographic regions and clinical settings leads to the inherent problem of incomplete and not always compatible data, limiting the overall validity of the analysis. Third, the overall small number in subgroup analyses limits their validity and results may not reflect the true picture.

### Key learning points

- TBRF is widespread worldwide, with transmission occurring by soft as well as hard ticks
- although only PCR-based methods allow for species identification, microscopy remains the diagnostic gold standard in most clinical settings
- the risk of JHR is apparently lower in TBRF compared to LBRF
- the overall case fatality rate of TBRF and LBRF appears not to differ
- unlike LBRF, where perinatal fatalities are primarily attributable to abortion, TBRF-related perinatal fatalities appear to primarily affect newborns

## Supporting information

S1 Text Systematic review protocol. Established to conduct this systematic review.
(PDF)

S2 Text List of databases with search terms used. Terms used for the study research in the different databases.
(PDF)

S3 Text Reference list. Reference list of included and excluded publications.
(PDF)

S1 Table Data extraction sheet. Used for screening and selecting eligible publications.
(PDF)

S2 Table TBRF treatment details. Treatment details: antimicrobial treatment regimen, dosage and duration.
(PDF)

S1 PRISMA Checklist PRISMA checklist. Twenty-seven-item checklist for systematic reviews. PRISMA, Preferred Reporting Items for Systematic Reviews and Meta-Analyses.
(PDF)

S1 Data List of *Borrelia* spp. and ticks. List of *Borrelia* spp. and ticks associated to TBRF reported worldwide.
(XLSX)

S2 Data Data master sheet. Excel sheet containing the underlying numerical data.
(XLSX)

S1 Fig Supporting maps. Distribution of competent vector ticks for TBRF *Borrelia* spp.
(PDF)

S2 Fig Subgroup analysis. Subgroup analysis of *B*. *crocidurae* and *B*. *hermsii*.
(PDF)
