# Supplementary material for: Tick borne relapsing fever - a systematic review and analysis of the literature
Source: PLoS Negl Trop Dis. 2022 Feb 16;16(2):e0010212. doi: 10.1371/journal.pntd.0010212 (PMC8887751; doi:10.1371/journal.pntd.0010212)
Supplement: S1 Text — Established to conduct this systematic review. (PDF) [file pntd.0010212.s001.pdf]

Tick borne relapsing fever – a systematic review and analysis of the literature

S1 Text

Systematic review protocol

Contents

Review Title: 1
Reviewer roles 2
Centre conducting the review: 2
Background: 2
Results of Scoping searches/summary of existing literature: 3
Study rationale: 4
Review questions/objective: 4
Inclusion criteria: 4
Outcomes, intervention and phenomena of interest: 6
Types of studies: 6
Search and selection strategy: 6
Assessment of methodological quality: 7
Data extraction: 8
Data synthesis, discussion and context: 8
Authors: 9
References 9

Review Title:

What is the current state of knowledge concerning epidemiology and the geographical spread of TBRF? What are Risk Factors to acquiring TBRF and what are Risk Factor for fatal outcome of TBRF? What is the clinical impact of TBRF?

Reviewer roles

Primary reviewers: Ákos Jakab and Pascal Kahlig

Secondary reviewers: Esther Kuenzli and Andreas Neumayr

Quality assessors: Esther Kuenzli and Andreas Neumayr

Centre conducting the review:

Swiss Tropical and Public Health Institute, Department of Medicine

University of Basel, Faculty of Medicine

Background:

Two febrile illnesses related to human pathogenic spirochetes belonging to the genera borrelia present as "relapsing fevers", louse-borne relapsing fever (LBRF) and tick-borne relapsing fever (TBRF). While LBRF is an anthroponotic disease exclusively caused by Borrelia recurrentis, TBRF is a zoonotic disease caused by various Borrelia species. In different regions of the world different Borrelia spp. have been identified to be endemic. They differ in their natural enzootic cycles (involving different tick species and their hosts, mostly small rodents) and they are capable of infecting humans as accidental dead-end hosts [1]. Since LBRF and TBRF present clinically identical and LBRF- and TBRF-borrelia are microscopically indistinguishable, differentiation between the two diseases was historically limited to the epidemiological circumstances (LBRF: outbreaks, epidemics, occurrence in vulnerable populations exposed to body lice; TBRF: sporadic cases in persons exposed to ticks). Finally, the advent of molecular diagnostic techniques not only enabled to distinguish TBRF from LBRF, but also significantly changed the understanding of the diversity and epidemiology of TBRF.

Historically, in most regions of the world TBRF has always been overshadowed by LBRF which was more prominent and epidemiologically relevant because of its epidemic occurrence. With the decline of lice-infested populations in most regions of the world, LBRF became a rare disease, while TBRF received increasing attention, especially in recent years. TBRF has been recognized in Africa since 1904, owing to the researches of Ross, Dutton and others [2]. In the early 1920s, TBRF was also recognized as an endemic disease in the United States of America (USA), although a tick vector was not recognized until 1930 [3]. In the following years, case reports of TBRF showed the extend of endemic areas in the USA and various tick species were identified as vectors [4]. Today, TBRF is reported from all continents except Australia and Antarctica [5] and constitutes an important public

health problem in some parts of the world. In Western Africa, TBRF accounts for about 13% of febrile illnesses [6] and in endemic regions of East Africa, TBRF is one of the diseases with the highest lethality among children [7].

The incubation period of TBRF is 4-18 days. Thereafter, up to 12 recurrent febrile episodes occur. These fever episodes last 2-7 days and are separated by afebrile periods of up to 10 days [8]. A broad range of accompanying unspecific as well as neurologic complications may occur.

Relapsing fever borrelia causes massive, microscopically visible bacteremia during febrile episodes. Therefore, the microscopic examination of blood smears has been the diagnostic method of choice since relapsing fever borrelia were first microscopically detected in the blood of patients by Obermeier in 1873 [9]. With the introduction of polymerase chain reaction (PCR) and sequencing techniques in the 1980s, highly sensitive and specific diagnostic tools became available. However, the availability of these molecular diagnostic tools still remains largely restricted to research institutions and microscopy remains the diagnostic gold standard for TBRF even in affluent countries [10].

In the first half of the 20th century, arsenicals and emetine bismuth iodide were the only available drugs for the treatment of relapsing fever. After the discovery of penicillin, treatment shifted towards this antimicrobial agent in the second half of the century with alternative therapeutic agents becoming available over time (i.e. tetracyclines, macrolides). Today, the preferred antibiotics to treat TBRF are tetracyclines, β-lactams and macrolids [11] to which borrelia are invariably susceptible [12].

Treatment may be complicated by Jarisch-Herxheimer reaction (JHR), which mostly occurs after administering the first dose of the antibiotic. JHR is characterized by intense chills and a rise in temperature about 1-2 hours after initiating antibiotic treatment and may be complicated by hypotension. In TBRF, JHR is reported to occur in up to 54.1% of cases [13].

The lethality of untreated TBRF is reported to be 2–10% [14]. With antibiotic treatment the lethality is reported to be <2% [15]. TBRF infection during pregnancy is associated with an increased risk of death in pregnant women [16]. Infections during pregnancy are claimed to cause up to 10–15% of neonatal deaths worldwide and a perinatal lethality of up to 43.6% has been reported [1,14,17].

Results of Scoping searches/summary of existing literature:

Search on PROSPERO determined no planned or ongoing systematic review on this topic. Literature search about TBRF has shown many studies, although relapsing fever in many cases being a subtopic of Lyme Disease. Early scoping searches on Pubmed with a focus on TBRF have provided about 251 results, with approximately 60-70% of publications relevant to the review question. The snapshot of published evidence shows us rather few relevant studies and information about TBRF in high level

evidence studies such as RCT's. With some authors already mentioning the suspicion that it may be a neglected disease, it gives us further reason for our study rationale.

Study rationale:

The aim of this study is to review and analyse the existing literature on TBRF and to summarize the epidemiological, clinical, diagnostic and treatment aspects of the disease, including its transmission through ticks, its vector reservoir and its clinical outcome.

Review questions/objective:

Quantitative objectives to identify:

What is the current state of knowledge about the epidemiology and the geographical spread of TBRF?
What are Risk Factors associated to acquiring TBRF and for mortality?

What are the typical symptoms, complications, abnormal laboratory findings for TBRF?

What is the current knowledge about treatment of TBRF?

More specifically:
- collect the published literature about the chosen aspects of TBRF
- critically assess the grade of evidence of the collected literature
- summarize the evidence and create a critical overview with stratification by quality of report
- identify relevant research and knowledge gaps

Further aims beyond the research question:
- TBRF being a neglected tropical disease (NTD)
- find Risk Factors for Mortality from TBRF
- symptoms and frequency of JHR, possibly find associations with Risk Factors
- identify possible typical signs or symptoms for TBRF

Inclusion criteria:

All types of studies with good evidence, such as RCTs related to TBRF will be included. Focus will be on epidemiology, clinical impact and outcome. Studies including information about these topics will

be considered. All types of studies with unclear grade of evidence will be furtherly assessed by two independent reviewers. Exclusion criteria is clearly poor evidence, such as newspaper articles. No date limitation will be set. This may be slightly customized during the search and after discussion with the supervisor.

Based on the review questions from above, the Inclusion Criteria will be defined as:
Who/Population = All patients with TBRF
What = Impact of TBRF
How/on what = Epidemiology, clinic, management, outcome
Where = Global
Grade of evidence = Good OR average - after discussion with second reviewer
Study design = RCT's, non-randomized controlled trials, possibly any others after a check with second reviewers and grade of evidence

S1 Table A: Screening and Selection Tool.

Screening and Selection Tool		
Reviewer Name:		Date:
Author name/Study ID:		Year:
Title:		Journal:
	Include	Exclude
**Who/Population**	Patients with TBRF	Patients with other diseases
**What**	Impact of TBRF	Not concerning TBRF
**How/on what**	Epidemiology<br>Clinical information<br>Management<br>Outcome	Not containing any clinical, epidemiological information or information about management or outcome
**Where**	Global	-
**Grade of evidence**	Good OR average (after agreement between reviewers)	Clearly poor
**Study design**	RCT's OR non-randomized controlled trials OR possibly any others after a check with second reviewers and grade of evidence	Newspaper articles
**Date limitation**	All	None

Outcomes, intervention and phenomena of interest:

Clinical information about TBRF, furtherly specified in the data extraction list in the Appendix, are of interest to the review, as well as any information about epidemiology, management and outcome of TBRF. By conducting a wide-open search without geographical limitation, we are interested whether we will find any indicators of TBRF currently thriving in other regions of the world than only limited to Africa. Another phenomenon of interest is the impact caused by TBRF being a rather neglected disease. Finally, any clues about possible typical symptoms, laboratory abnormalities or complications are phenomena of interest.

Types of studies:

The search strategy is broadly set with consideration of all types of published study designs, as RCTs about TBRF are rather few. Quality assessment will be crucial before definite inclusion. In doubt, two independent reviewers will be consulted for further assessment. What types of studies eventually will be included, will become apparent after completing the search and quality assessment. Focus will be on published papers in English language. German, French, Italian and Hungarian papers will be reviewed without constrictions by one of the authors, sufficiently understanding the language and using dictionaries. In doubt, further languages will be managed by outsource translation. We aim to have no language bias in selecting the studies for the review.

Search and selection strategy:

The search strategy aims to identify all relevant published studies using the following approach:

1: Primarily, an electronic search will be conducted on Biosis Citation Index, Biosis Previews, CINAHL, Cochrane, Current Contents Connect, Data Citation Index, Derwent Innovations Index, EMBASE Elsevier, EMBASE Ovid, Inspec, Medline, PMC, PubMed, SciELO Citation Index, Scopus, Web of Science, and Zoological Record electronic databases using following search terms (adapted to the search format of the different databases):
("tick" OR "ticks" OR "tick borne" OR "Ornithodoros" OR "Borrelia" OR "Borrelia miyamotoi" OR "Borrelia turicatae" OR "Borrelia hermsii" OR "Borrelia parkeri" OR "Borrelia persica" OR "Borrelia hispanica" OR "Borrelia crocidurae" OR "Borrelia duttonii" OR "Borrelia caucasica" OR "Borrelia microti" OR "Borrelia brasiliensis" OR "Borrelia mazzottii" OR "Borrelia venezuelensis" OR "Borrelia graingeri" OR "Borrelia latyschewii" OR "Borrelia dugesii" OR "Borrelia Infections" OR "Borrelia") AND ("relapsing fever" OR "recurrent fever" OR "relapsing fever disease")
2: Identified material will be de-duplicated both by automatic search for duplicates by Endnote

software and manual search for duplicates, following this review. Titles and abstracts of material identified via searches using the keywords above (Stage 1: Screening) will be screened and reviewed manually. Ideally, screening will be conducted by two reviewers. Alternatively, screening will be conducted by one reviewer, in doubt, a second reviewer will be consulted. Secondly, the refence lists of identified relevant articles will be manually searched for additional studies or articles. Further identified material will be again screened and reference lists will be searched.

3: Full text papers of potentially eligible articles will be obtained

4: Inclusion criteria will be applied and full text papers selected for the review. Studies that didn't fulfil the criteria for inclusion will be excluded and their bibliographic details will be listed in an Appendix (Stage 2: Selection)

5: results will be reported using PRIMSA diagram

The bibliographic software ENDNOTE will be used for storage and processing.

Assessment of methodological quality:

The selected full text papers (after Stage 2: Selection) will be assessed by one reviewer for methodological validity prior to inclusion into the review. Any disagreements will be resolved through discussion between the reviewers, further disagreement will be intended to be resolved by a third independent reviewer.

6 key steps will be followed to assure a proper quality assessment:

1: The study designs of all the included studies will be evaluated and noted.

2: In case of a variety of designs, design-specific assessment tools will be used.

3: Appropriate quality assessment tools will be chosen

4: Quality assessment will be carried out using the right tool, carefully documenting where in the studies information relating each quality assessment question was found. Records will be kept of how the reviewer made a decision, especially if he was unable to clearly response to a question.

5: The results will be tabulated, summarized, stratified and presented in the quality assessment section if applicable.

6: It will be considered and discussed how the quality assessment results might have an impact on the conclusions and recommendations of the review.

Finally, at the end of writing the review, it will be checked using a systematic review quality assessment tool, the PRISMA checklist.

Data extraction:

Quantitative data will be extracted and included in the review in form of comprehensive tables. The extracted data will include specific details of significance to the review objective. Details about diagnostic measures, treatment regimens and any other information of significance or interest to the review objective will be noted and documented. It is not planned to extract qualitative data.
Deadline for obtaining studies in the review is set on the 04/DEC/2020.
In detail, the data extraction process will contain the following steps:
1: Any data that seems relevant for the review question will be identified and data extraction form and tables will be created. Stylistic rules of the favoured journal for publishing will be followed as far as possible at this stage.
2: The preformed data extraction form will be piloted using 3-5 of the selected studies to assess the viability of the data extraction form and possibly add or remove variables.
3: Data extraction will be conducted electronically using electronic versions of the paper. This process will be set after the quality assessment. Data will be stored in the data extraction form (Excel) using “copy and paste” to minimize data entry errors. This process will be conducted by one reviewer. Alternatively, depending on the availability of a second reviewer, a second reviewer will crosscheck the extracted data for accuracy, or the first reviewer will conduct a second full data extraction at least one week after the first data extraction to ensure proper data entry and that same results are obtained from both extractions.
4: Data extraction tables will be completed and reported in the review.

Data synthesis, discussion and context:

The data will be visually presented in summary tables and synthesized narratively in a sense of an observational analysis. As far as the available data allows, associations and conclusions will be drawn. Where we can, we will attempt to group similar data.

At this stage, the decision will be made whether the data will only be synthesized narratively or if the data is sufficient for a meta-analysis. Four aspects will be assessed whether it is appropriate to combine the results in a meta-analysis.
1: Studies should be similar in terms of the patients (inclusion criteria, patient characteristics)
2: Interventions/Exposures and Comparators should be the same
3: The same outcomes should be reported (primary or secondary, as well as time frames)
4: The results should show that the effects/impacts are generally going into the same direction (visualized by forest plot using a statistical software)

If all four criteria are sufficiently fulfilled by the data from reviewed studies, a meta-analysis will be performed – due to rather expectable lack of sufficient homogenic data, further planning in this direction isn't appropriate now. In case of only some studies meeting all the criteria, it may be considered to perform a meta-analysis only using those studies. In this case, a sensitivity analysis will be carried out, using the remaining studies to test the robustness of the results. If all included studies meet most of the criteria, it may as well be sensible to combine them in a meta-analysis. Both cases would be carefully discussed among the reviewers and considered to be included in the review.

As the scoping search showed a relatively limited number of published RCT studies, we may expect limitations due to a lack of data. It is likely in this review, that we will have to deal with a variety of different study designs with different study aims, resulting in a rather big heterogeneity. Furthermore, the review question isn't just comparing two different interventions on the outcome, we want to get a further picture of TBRF. Examining the epidemiology, the clinical impact, the management and outcome, we will most likely have to deal with a big variety of data or possibly a big lack of data. Since one of the aims of our review is to show the current state of knowledge, discovering possible knowledge gaps, this will be considered and discussed in the "discussion" and "conclusions" section.

The results section will be followed by a "discussion" section.

Authors:

Ákos Jakab, Pascal Kahlig, Esther Kuenzli, Andreas Neumayr

References

1. Rodino KG, Theel ES, Pritt BS. Tick-Borne Diseases in the United States. Clinical chemistry. 2020;66(4):537-48.
2. Nicholson FD. TICK FEVER IN PALESTINE. Br Med J. 1919;2(3077):811.
3. Weller B, Graham GM. RELAPSING FEVER IN CENTRAL TEXAS. Journal of the American Medical Association. 1930;95(24):1834.
4. Davis GE. TICKS AND RELAPSING FEVER IN THE UNITED STATES. Public Health Reports. 1940;55(51):2347-51.
5. Burrascano JJ. Relapsing fever. Clinical Infectious Disease2010. p. 1135-8.
6. Parola P, Diatta G, Socolovschi C, Mediannikov O, Tall A, Bassene H, et al. Tick-borne relapsing fever borreliosis, rural senegal. Emerging Infectious Diseases. 2011;17(5):883-5.
7. Talbert A, Nyange A, Molteni F. Spraying tick-infested houses with lambda-cyhalothrin reduces the incidence of tick-borne relapsing fever in children under five years old. Transactions of the Royal Society of Tropical Medicine and Hygiene. 1998;92(3):251-3.

8. Dworkin MS, Schwan TG, Anderson Jr DE, Borchardt SM. Tick-Borne Relapsing Fever. Infectious Disease Clinics of North America. 2008;22(3):449-68.
9. Kahlig P, Paris DH, Neumayr A. Louse-borne relapsing fever—A systematic review and analysis of the literature: Part 1—Epidemiology and diagnostic aspects. PLOS Neglected Tropical Diseases. 2021;15(3):e0008564.
10. Cutler SJ, Rudenko N, Golovchenko M, Cramaro WJ, Kirpach J, Savic S, et al. Diagnosing Borreliosis. Vector-Borne and Zoonotic Diseases. 2017;17(1):2-11.
11. Hasin T, Davidovitch N, Cohen R, Dagan T, Romem A, Orr N, et al. Postexposure treatment with doxycycline for the prevention of tick-borne relapsing fever. New England Journal of Medicine. 2006;355(2):148-55.
12. Aguero-Rosenfeld Marie E, Stanek G. Borrelia. 12th ed2019.
13. Dworkin MS, Anderson DE, Jr., Schwan TG, Shoemaker PC, Banerjee SN, Kassen BO, et al. Tick-borne relapsing fever in the northwestern United States and southwestern Canada. Clin Infect Dis. 1998;26(1):122-31.
14. Goddard J, Goddard J. Tick-Borne Diseases2018. 91-147 p.
15. Barbour AG. Relapsing fever. Goodman JL, Dennis DT, Sonenshine DE, editors2005. 268-91 p.
16. Jongen VHWM, Van Roosmalen J, Tiems J, Van Holten J, Wetsteyn JCFM. Tick-borne relapsing fever and pregnancy outcome in rural Tanzania. Acta Obstetricia et Gynecologica Scandinavica. 1997;76(9):834-8.
17. Lambert JS. An Overview of Tickborne Infections in Pregnancy and Outcomes in the Newborn: The Need for Prospective Studies. Front Med (Lausanne). 2020;7.
