# Supplementary material for: Tick borne relapsing fever - a systematic review and analysis of the literature
Source: PLoS Negl Trop Dis. 2022 Feb 16;16(2):e0010212. doi: 10.1371/journal.pntd.0010212 (PMC8887751; doi:10.1371/journal.pntd.0010212)
Supplement: S2 Text — Terms used for the study research in the different databases. (PDF) [file pntd.0010212.s002.pdf]

Tick borne relapsing fever – a systematic review and analysis of the literature

S2 Text

List of databases with search terms used.

- **Biosis Citation Index, Biosis Previews, Current Contents Connect, Data Citation Index, Derwent Innovations Index, Inspec (Information Service for Physics, Electronics, and Computing), SciELO Citation Index, Web of Science core collection, Zoological Record:**
(TS=(tick) OR TS=(ticks) OR TS=("tick borne") OR TS=(*Ornithodoros*) OR TS=(*Borrelia*) OR TS=(*Borreliae*) OR TS=("*Borrelia miyamotoi*") OR TS=("*Borrelia turicatae*") OR TS=("*Borrelia hermsii*") OR TS=("*Borrelia parkeri*") OR TS=("*Borrelia persica*") OR TS=("*Borrelia hispanica*") OR TS=("*Borrelia crocidurae*") OR TS=("*Borrelia duttonii*") OR TS=("*Borrelia caucasica*") OR TS=("*Borrelia microti*") OR TS=("*Borrelia brasiliensis*") OR TS=("*Borrelia mazzottii*") OR TS=("*Borrelia venezuelensis*") OR TS=("*Borrelia graingeri*") OR TS=("*Borrelia latyschweii*") OR TS=("*Borrelia dugesii*")) AND (TS=("relapsing fever*") OR TS=("recurrent fever") OR TS=("relapsing fever disease"))

- **CINAHL (Cumulative Index to Nursing and Allied Health Literature):**
(Tick OR ticks OR "tick borne" OR *Ornithodoros* OR *Borrelia* OR *Borreliae* OR "*Borrelia miyamotoi*" OR "*Borrelia turicatae*" OR "*Borrelia hermsii*" OR "*Borrelia parkeri*" OR "*Borrelia persica*" OR "*Borrelia hispanica*" OR "*Borrelia crocidurae*" OR "*Borrelia duttonii*" OR "*Borrelia caucasica*" OR "*Borrelia microti*" OR "*Borrelia brasiliensis*" OR "*Borrelia mazzottii*" OR "*Borrelia venezuelensis*" OR "*Borrelia graingeri*" OR "*Borrelia latyschweii*" OR "*Borrelia dugesii*" OR MH "*Ornithodoros*" OR MH "ticks" OR MH "Tick-Borne Diseases" OR MH "*Borrelia* Infections" OR MH "*Borrelia*") AND ("relapsing fever*" OR "recurrent fever" OR "relapsing fever disease" OR MH "Relapsing Fever")

- **Cochrane Library:**
(Tick OR ticks OR "tick borne" OR *Ornithodoros* OR *Borrelia* OR *Borreliae* OR "*Borrelia miyamotoi*" OR "*Borrelia turicatae*" OR "*Borrelia hermsii*" OR "*Borrelia parkeri*" OR "*Borrelia persica*" OR "*Borrelia hispanica*" OR "*Borrelia crocidurae*" OR "*Borrelia duttonii*" OR "*Borrelia caucasica*" OR "*Borrelia microti*" OR "*Borrelia brasiliensis*" OR "*Borrelia mazzottii*" OR "*Borrelia venezuelensis*" OR "*Borrelia graingeri*" OR "*Borrelia latyschweii*" OR "*Borrelia dugesii*" OR MeSH Terms) AND ("relapsing fever*" OR "recurrent fever" OR "relapsing fever disease" OR MeSH Terms)

- **Embase Elsevier:**
(Tick OR ticks OR 'tick borne' OR *Ornithodoros* OR *Borrelia* OR *Borreliae* OR '*Borrelia miyamotoi*' OR '*Borrelia turicatae*' OR '*Borrelia hermsii*' OR '*Borrelia parkeri*' OR '*Borrelia persica*' OR '*Borrelia hispanica*' OR '*Borrelia crocidurae*' OR '*Borrelia duttonii*'

OR '*Borrelia caucasica*' OR '*Borrelia microti*' OR '*Borrelia brasiliensis*' OR '*Borrelia mazzottii*' OR '*Borrelia venezuelensis*' OR '*Borrelia graingeri*' OR '*Borrelia latyschweii*' OR '*Borrelia dugesii*') AND ('relapsing fever*' OR 'recurrent fever' OR 'relapsing fever disease')

- **Medline:**
  (TS=(tick) OR TS=(ticks) OR TS=("tick borne") OR TS=(*Ornithodoros*) OR TS=(*Borrelia*) OR TS=(*Borreliae*) OR TS=("*Borrelia miyamotoi*") OR TS=("*Borrelia turicatae*") OR TS=("*Borrelia hermsii*") OR TS=("*Borrelia parkeri*") OR TS=("*Borrelia persica*") OR TS=("*Borrelia hispanica*") OR TS=("*Borrelia crocidurae*") OR TS=("*Borrelia duttonii*") OR TS=("*Borrelia caucasica*") OR TS=("*Borrelia microti*") OR TS=("*Borrelia brasiliensis*") OR TS=("*Borrelia mazzottii*") OR TS=("*Borrelia venezuelensis*") OR TS=("*Borrelia graingeri*") OR TS=("*Borrelia latyschweii*") OR TS=("*Borrelia dugesii*") OR MH=(*Ornithodoros*) OR MH=(ticks) OR MH=("Tick-Borne Diseases") OR MH=("*Borrelia* Infections") OR MH=(*Borrelia*)) AND (TS=("relapsing fever*") OR TS=("recurrent fever") OR TS=("relapsing fever disease") OR MH=("Relapsing Fever"))

- **OVID Embase:**
  ((Tick or ticks or "tick borne" or *Ornithodoros* or *Borrelia* or *Borreliae* or "*Borrelia miyamotoi*" or "*Borrelia turicatae*" or "*Borrelia hermsii*" or "*Borrelia parkeri*" or "*Borrelia persica*" or "*Borrelia hispanica*" or "*Borrelia crocidurae*" or "*Borrelia duttonii*" or "*Borrelia caucasica*" or "*Borrelia microti*" or "*Borrelia brasiliensis*" or "*Borrelia mazzottii*" or "*Borrelia venezuelensis*" or "*Borrelia graingeri*" or "*Borrelia latyschweii*" or "*Borrelia dugesii*").mp. [mp=title, abstract, heading word, drug trade name, original title, device manufacturer, drug manufacturer, device trade name, keyword, floating subheading word, candidate term word] OR exp tick borne disease/ OR exp *Borrelia* infection/ OR exp tick/ OR exp *Borrelia*/ OR exp *Ornithodoros*/) AND (("relapsing fever*" or "recurrent fever" or "relapsing fever disease" or "MH Relapsing Fever").mp. [mp=title, abstract, heading word, drug trade name, original title, device manufacturer, drug manufacturer, device trade name, keyword, floating subheading word, candidate term word])

- **PMC (PubMed Central):**
  (Tick OR ticks OR "tick borne" OR *Ornithodoros* OR *Borrelia* OR *Borreliae* OR "*Borrelia miyamotoi*" OR "*Borrelia turicatae*" OR "*Borrelia hermsii*" OR "*Borrelia parkeri*" OR "*Borrelia persica*" OR "*Borrelia hispanica*" OR "*Borrelia crocidurae*" OR "*Borrelia duttonii*" OR "*Borrelia caucasica*" OR "*Borrelia microti*" OR "*Borrelia brasiliensis*" OR "*Borrelia mazzottii*" OR "*Borrelia venezuelensis*" OR "*Borrelia graingeri*" OR "*Borrelia latyschweii*" OR "*Borrelia dugesii*" OR "*Ornithodoros*"[Mesh] OR "Ticks"[Mesh] OR "Tick-Borne Diseases"[Mesh] OR "*Borrelia* Infections"[Mesh] OR "*Borrelia*"[Mesh]) AND ("relapsing fever*" OR "recurrent fever" OR "relapsing fever disease" OR "Relapsing Fever"[Mesh])

- **PubMed:**
  (Tick OR ticks OR "tick borne" OR *Ornithodoros* OR *Borrelia* OR *Borreliae* OR "*Borrelia*

miyamotoi" OR "*Borrelia turicatae*" OR "*Borrelia hermsii*" OR "*Borrelia parkeri*" OR "*Borrelia persica*" OR "*Borrelia hispanica*" OR "*Borrelia crocidurae*" OR "*Borrelia duttonii*" OR "*Borrelia caucasica*" OR "*Borrelia microti*" OR "*Borrelia brasiliensis*" OR "*Borrelia mazzottii*" OR "*Borrelia venezuelensis*" OR "*Borrelia graingeri*" OR "*Borrelia latyschweii*" OR "*Borrelia dugesii*" OR "*Ornithodoros*"[Mesh] OR "Ticks"[Mesh] OR "Tick-Borne Diseases"[Mesh] OR "*Borrelia* Infections"[Mesh] OR "*Borrelia*"[Mesh] OR "*Borrelia miyamotoi*" [Supplementary Concept] OR "*Borrelia turicatae*" [Supplementary Concept] OR "*Borrelia hermsii*" [Supplementary Concept] OR "*Borrelia parkeri*" [Supplementary Concept] OR "*Borrelia persica*" [Supplementary Concept] OR "*Borrelia hispanica*" [Supplementary Concept] OR "*Borrelia crocidurae*" [Supplementary Concept] OR "*Borrelia duttonii*" [Supplementary Concept] OR "*Borrelia caucasica*" [Supplementary Concept] OR "*Borrelia microti*" [Supplementary Concept] OR "*Borrelia brasiliensis*" [Supplementary Concept] OR "*Borrelia mazzottii*" [Supplementary Concept] OR "*Borrelia venezuelensis*" [Supplementary Concept] OR "*Borrelia graingeri*" [Supplementary Concept] OR "*Borrelia latyschweii*" [Supplementary Concept] OR "*Borrelia dugesii*" [Supplementary Concept]) AND ("relapsing fever*" OR "recurrent fever" OR "relapsing fever disease" OR "Relapsing Fever"[Mesh])

- **Scopus:**
  (tick OR ticks OR tick borne OR *Ornithodoros* OR *Borrelia** OR "*Borrelia miyamotoi*" OR "*Borrelia turicatae*" OR "*Borrelia hermsii*" OR "*Borrelia parkeri*" OR "*Borrelia persica*" OR "*Borrelia hispanica*" OR "*Borrelia crocidurae*" OR "*Borrelia duttonii*" OR "*Borrelia caucasica*" OR "*Borrelia microti*" OR "*Borrelia brasiliensis*" OR "*Borrelia mazzottii*" OR "*Borrelia venezuelensis*" OR "*Borrelia graingeri*" OR "*Borrelia latyschweii*" OR "*Borrelia dugesii*") AND ("relapsing fever" OR "recurrent fever" OR "relapsing fever disease")
