# Supplementary material for: Tick borne relapsing fever - a systematic review and analysis of the literature
Source: PLoS Negl Trop Dis. 2022 Feb 16;16(2):e0010212. doi: 10.1371/journal.pntd.0010212 (PMC8887751; doi:10.1371/journal.pntd.0010212)
Supplement: S3 Text — Reference list of included and excluded publications. (PDF) [file pntd.0010212.s003.pdf]

Tick borne relapsing fever – a systematic review and analysis of the literature

S3 Text

Reference list.

Included references

- Database search [1-760]
- Snowball search [761-837]

Excluded references

- Language [838-906]
- Other disease [907-1043]
- Did not provide relevant information [1044-1470]
- Duplicate [1471-1500]
- Not retrievable [1501-1605]

1. Zakham F, Jääskeläinen AJ, Castrén J, Sormunen JJ, Uusitalo R, Smura T, et al. Molecular detection and phylogenetic analysis of Borrelia miyamotoi strains from ticks collected in the capital region of Finland. Ticks Tick Borne Dis. 2020;12(2):101608.
2. Yu S, Modarelli J, Tomeček JM, French JT, Hilton C, Esteve-Gasent MD. Prevalence of common tick-borne pathogens in white-tailed deer and coyotes in south Texas. Int J Parasitol Parasites Wildl. 2020;11:129-35.
3. Yahia SA, Faibis F, Benmoussa M, Lantohasina N, Dupont A, Abdesselam TA. Tick-borne relapsing fever : An unrecognized cause of fever in travellers. Revue De Medecine Interne. 2020;41(6):418-20.
4. Wormser GP. Doxycycline for Prevention of Spirochetal Infections: Status Report. Clinical Infectious Diseases. 2020;71(8):2014-7.
5. Wolf MJ, Watkins HR, Schwan WR. Ixodes scapularis: Vector to an Increasing Diversity of Human Pathogens in the Upper Midwest. Wmj. 2020;119(1):16-21.
6. Tobudic S, Burgmann H, Stanek G, Winkler S, Schotta A-M, Obermuller M, et al. Human Borrelia miyamotoi Infection, Austria. Emerging infectious diseases. 2020;26(9):2201-4.

7. Thomas Sanchez RS, Santodomingo Santodomingo AM, Munoz-Leal S, Carolina Silva-de la Fuente M, Llanos-Soto S, Moreno Salas L, et al. Rodents as potential reservoirs for Borrelia spp. in northern Chile. Revista Brasileira de Parasitologia Veterinaria. 2020;29(2).
8. Talagrand-Reboul E, Raffetin A, Zachary P, Jaulhac B, Eldin C. Immunoserological Diagnosis of Human Borrelioses: Current Knowledge and Perspectives. Frontiers in Cellular and Infection Microbiology. 2020;10.
9. Springer A, Raulf MK, Fingerle V, Strube C. Borrelia prevalence and species distribution in ticks removed from humans in Germany, 2013–2017. Ticks and Tick-borne Diseases. 2020;11(2).
10. Snowden J, Yarrarapu SNS, Oliver TI. Relapsing Fever. StatPearls. Treasure Island (FL): StatPearls Publishing; 2020.
11. Snavely E, Hymas W, Couturier MR, Couturier MR. The brief case: Tick-borne relapsing fever in a returned traveler. Journal of Clinical Microbiology. 2020;58(6).
12. Seto J, Tanaka S, Kawabata H, Ito Y, Ikeda T, Mizuta K. Detection of tick-borne pathogens in ticks from dogs and cats in Yamagata Prefecture, Japan, 2018. Japanese journal of infectious diseases. 2020.
13. Santos CAd, Suzin A, Vogliotti A, Nunes PH, Barbieri ARM, Labruna MB, et al. Molecular detection of a Borrelia sp. in nymphs of Amblyomma brasiliense ticks (Atari: Ixodidae) from Iguacu National Park, Brazil, genetically related to Borrelia from Ethiopia and Cote d'Ivoire. Ticks and Tick-borne Diseases. 2020;11(6):101519.
14. Sambado S, Salomon J, Crews A, Swei A. Mixed transmission modes promote persistence of an emerging tick-borne pathogen. Ecosphere. 2020;11(6):e03171.
15. Röttgerding F, Kraiczy P. Immune Evasion Strategies of Relapsing Fever Spirochetes. Frontiers in Immunology. 2020;11.
16. Rodino KG, Theel ES, Pritt BS. Tick-Borne Diseases in the United States. Clinical chemistry. 2020;66(4):537-48.
17. Rochlin I, Toledo A. Emerging tick-borne pathogens of public health importance: a mini-review. J Med Microbiol. 2020;69(6):781-91.
18. Raileanu C, Tauchmann O, Vasic A, Woehnke E, Silaghi C. Borrelia miyamotoi and Borrelia burgdorferi (sensu lato) identification and survey of tick-borne encephalitis virus in ticks from north-eastern Germany. Parasites & Vectors. 2020;13(1):106.
19. Pineda Cantero A, Pérez de Pedro I, Martín Téllez S, Costo Muriel C, Caballero Martínez LF, Gómez Huelgas R. Borrelia hispanica as a cause of recurrent fever. Medicina Clinica. 2020;154(9):380.
20. Pedersen BN, Jenkins A, Kjelland V. Tick-borne pathogens in Ixodes ricinus ticks collected from migratory birds in southern Norway. PLoS One. 2020;15(4).
21. Pavia CS. Immunologic detection of Lyme disease and the related borrelioses. Methods in Microbiology. 2020;47:41-74.
22. Park JW, Lee SH, Lee GS, Seo JJ, Chung JK. Epidemiological Characteristics of Field Tick-Borne Pathogens in Gwang-ju Metropolitan Area, South Korea, from 2014 to 2018. Osong Public Health Res Perspect. 2020;11(4):177-84.
23. Ouchene N, Nebbak A, Ouchene-Khelifi NA, Dahmani A, Zeroual F, Khelef D, et al. Molecular detection of avian spirochete Borrelia anserina in Argas persicus ticks in Algeria. Comparative Immunology, Microbiology and Infectious Diseases. 2020;68.
24. O’Kelly B, Lambert JS. Vector-borne diseases in pregnancy. Ther Adv Infect Dis. 2020;7.

25. Naddaf SR, Mahmoudi A, Ghasemi A, Rohani M, Mohammadi A, Ziapour SP, et al. Infection of hard ticks in the Caspian Sea littoral of Iran with Lyme borreliosis and relapsing fever borreliae. Ticks and Tick-borne Diseases. 2020;11(6).
26. Muñoz-Leal S, Venzal JM, Nava S, Marcili A, González-Acuña D, Martins TF, et al. Description of a new soft tick species (Acari: Argasidae: Ornithodoros) parasite of Octodon degus (Rodentia: Octodontidae) in northern Chile. Ticks and Tick-borne Diseases. 2020;11(3).
27. Muñoz-Leal S, Martins MM, Nava S, Landulfo GA, Simons SM, Rodrigues VS, et al. Ornithodoros cerradoensis n. sp. (Acari: Argasidae), a member of the Ornithodoros talaje (Guérin-Méneville, 1849) group, parasite of rodents in the Brazilian Savannah. Ticks and Tick-borne Diseases. 2020;11(5).
28. Muñoz-Leal S, Faccini-Martínez Á A, Pérez-Torres J, Chala-Quintero SM, Herrera-Sepúlveda MT, Cuervo C, et al. Novel Borrelia genotypes in bats from the Macaregua Cave, Colombia. Zoonoses Public Health. 2020.
29. Muigg V, Seth-Smith HMB, Goldenberger D, Egli A, Nickel B, Dürig R, et al. Tick-Borne Relapsing Fever Caused by Borrelia persica in Traveler to Central Asia, 2019. Emerging Infectious Diseases. 2020;26(4):424-6.
30. Morales-Diaz J, Colunga-Salas P, Romero-Salas D, Sánchez-Montes S, Estrada-Souza IM, Ochoa-Ochoa LM, et al. Molecular detection of reptile-associated Borrelia in Boa constrictor (Squamata: Boidae) from Veracruz, Mexico. Acta Tropica. 2020;205.
31. Moradi-Asl E, Vatandoost H, Kumar S. Prevalence and seasonal activity of ticks infesting livestock in north west areas of Iran. Pakistan Journal of Medical and Health Sciences. 2020;14(1):455-8.
32. Moradi-Asl E, Jafari S. The habitat suitability model for the potential distribution of Ornithodoros tholozani (Laboulbène et Mégnin, 1882) and Ornithodoros lahorensis (Neumann, 1908) (Acari: Argasidae): the main vectors of tick-borne relapsing fever in Iran. Annals of parasitology. 2020;66(3):357-63.
33. Medkour H, Laidoudi Y, Marié JL, Fenollar F, Davoust B, Mediannikov O. Molecular investigation of vector-borne pathogens in red foxes (Vulpes vulpes) from southern France. Journal of Wildlife Diseases. 2020;56(4):837-50.
34. Masuzawa T, Sakakibara K, Suzuki K, Sato H, Yasuda S. Detection of Asian-Type Borrelia miyamotoi from Ixodes ricinus Inhabiting Tver Province (Russia): A Sympatric Region for I. ricinus and Ixodes persulcatus. Vector borne and zoonotic diseases (Larchmont, NY). 2020.
35. Margos G, Pantchev N, Globokar M, Lopez J, Rodon J, Hernandez L, et al. First Cases of Natural Infections with Borrelia hispanica in Two Dogs and a Cat from Europe. Microorganisms. 2020;8(8).
36. Margos G, Fingerle V, Oskam C, Stevenson B, Gofton A. Comment on: Gupta, 2019, distinction between Borrelia and Borreliella is more robustly supported by molecular and phenotypic characteristics than all other neighbouring prokaryotic genera: Response to Margos' et al. "The genus Borrelia reloaded" (PLoS One 13(12): e0208432). PLoS One 14(8):e0221397. Ticks and Tick-Borne Diseases. 2020;11(2):1320-.
37. Margos G, Fingerle V, Cutler S, Gofton A, Stevenson B, Estrada-Peña A. Controversies in bacterial taxonomy: The example of the genus Borrelia. Ticks and Tick-borne Diseases. 2020;11(2).
38. Marcos LA, Smith K, Reardon K, Weinbaum F, Spitzer ED. Presence of Borrelia miyamotoi infection in a highly endemic area of Lyme disease. Annals of Clinical Microbiology and Antimicrobials. 2020;19(1).

39. Mancini F, Innocenti P, Baumgartner M, Binazzi R, Troi C, Pagani E, et al. Borrelia microti infection in an Italian woman returning from Kyrgyzstan and Tajikistan. Travel Medicine and Infectious Disease. 2020;35.
40. Madison-Antenucci S, Kramer LD, Gebhardt LL, Kauffman E. Emerging tick-borne diseases. Clinical Microbiology Reviews. 2020;33(2).
41. Lau ACC, Qiu Y, Moustafa MAM, Nakao R, Shimozuru M, Onuma M, et al. Detection of borrelia burgdorferi sensu lato and relapsing fever borrelia in feeding ixodes ticks and rodents in Sarawak, Malaysia: New geographical records of borrelia yangtzensis and borrelia miyamotoi. Pathogens. 2020;9(10):1-18.
42. Latas P, Auckland LD, Teel PD, Hamer SA. ARGAS (PERSICARGAS) GIGANTEUS SOFT TICK INFECTION WITH RICKETTSIA HOOGSTRAALI AND RELAPSING FEVER BORRELIA ON WILD AVIAN SPECIES OF THE DESERT SOUTHWEST, USA. Journal of wildlife diseases. 2020;56(1):113-25.
43. Lambert JS. An Overview of Tickborne Infections in Pregnancy and Outcomes in the Newborn: The Need for Prospective Studies. Front Med (Lausanne). 2020;7.
44. Kalmár Z, Dumitrache MO, D'Amico G, Matei IA, Ionică AM, Gherman CM, et al. Multiple Tick-Borne Pathogens in Ixodes ricinus Ticks Collected from Humans in Romania. Pathogens. 2020;9(5).
45. Kaenkan W, Nooma W, Chelong IA, Baimai V, Trinachartvanit W, Ahantarig A. Reptile-associated Borrelia spp. In Amblyomma ticks, Thailand. Ticks and Tick-borne Diseases. 2020;11(1).
46. Hui-Ju H, Jian-Wei L, Hong-Ling W, Ze-Min L, Si-Cong L, Xiang-Rong Q, et al. Pathogenic New World Relapsing Fever Borrelia in a Myotis Bat, Eastern China, 2015. Emerging Infectious Diseases. 2020;26(12):3083-5.
47. Houmansadr F, Soleimani M, Naddaf SR. Development of a loop-mediated isothermal amplification (LAMP) assay for detection of relapsing fever borreliae. Journal of Arthropod-Borne Diseases. 2020;14(1):47-55.
48. Hojgaard A, Osikowicz LM, Eisen L, Eisen RJ. Evaluation of a novel multiplex PCR amplicon sequencing assay for detection of human pathogens in Ixodes ticks. Ticks and Tick-borne Diseases. 2020;11(6).
49. Hepner S, Fingerle V, Duscher GG, Felsberger G, Marosevic D, Rollins RE, et al. Population structure of Borrelia turcica from Greece and Turkey. Infection, Genetics and Evolution. 2020;77.
50. Heglasová I, Rudenko N, Golovchenko M, Zubriková D, Miklisová D, Stanko M. Ticks, fleas and rodent-hosts analyzed for the presence of Borrelia miyamotoi in Slovakia: the first record of Borrelia miyamotoi in a Haemaphysalis inermis tick. Ticks and Tick-borne Diseases. 2020;11(5).
51. Hashavya S, Gross I, Gross M, Hurvitz N, Weiser G, Temper V, et al. Tickborne Relapsing Fever, Jerusalem, Israel, 2004–2018. Emerg Infect Dis. 2020;26(10):2420-3.
52. Han SW, Chae JB, Jo YS, Cho YK, Kang JG, Shin NS, et al. First Report of Newly Identified Ornithodoros Species in the Republic of Korea. Journal of Parasitology. 2020;106(5):546-63.
53. Han S, Hickling GJ, Ogden NH, Ginsberg HS, Kobbekaduwa V, Rulison EL, et al. Seasonality of acarological risk of exposure to Borrelia miyamotoi from questing life stages of Ixodes scapularis collected from Wisconsin and Massachusetts, USA. Ticks Tick Borne Dis. 2020;12(1):101556.
54. Grech-Angelini S, Stachurski F, Vayssier-Taussat M, Devillers E, Casabianca F, Lancelot R, et al. Tick-borne pathogens in ticks (Acari: Ixodidae) collected from various domestic and

wild hosts in Corsica (France), a Mediterranean island environment. Transboundary and Emerging Diseases. 2020;67(2):745-57.
55. Goodrich I, McKee C, Kosoy M. Longitudinal Study of Bacterial Infectious Agents in a Community of Small Mammals in New Mexico. Vector-Borne and Zoonotic Diseases. 2020;20(7):496-508.
56. Gondard M, Delannoy S, Pinarello V, Aprelon R, Devillers E, Galon C, et al. Upscaling the Surveillance of Tick-Borne Pathogens in the French Caribbean Islands. Pathogens. 2020;9(3).
57. Gillingham EL, Hansford KM, Meadows S, Henney J, Wieckowski F, Hernández-Triana LM, et al. Ticks on the Channel Islands and implications for public health. Ticks and Tick-borne Diseases. 2020;11(3).
58. Gillingham EL, Cull B, Pietzsch ME, Phipps LP, Medlock JM, Hansford K. The Unexpected Holiday Souvenir: The Public Health Risk to UK Travellers from Ticks Acquired Overseas. Int J Environ Res Public Health. 2020;17(21).
59. Ghafar A, Cabezas-Cruz A, Galon C, Obregon D, Gasser RB, Moutailler S, et al. Bovine ticks harbour a diverse array of microorganisms in Pakistan. Parasit Vectors. 2020;13.
60. Franck M, Ghozzi R, Pajaud J, Lawson-Hogban NE, Mas M, Lacout A, et al. Borrelia miyamotoi: 43 Cases Diagnosed in France by Real-Time PCR in Patients With Persistent Polymorphic Signs and Symptoms. Frontiers in Medicine. 2020;7.
61. Dykstra EA, Oltean HN, Kangiser D, Marsden-Haug N, Rich SM, Guang X, et al. Ecology and Epidemiology of Tickborne Pathogens, Washington, USA, 2011-2016. Emerging Infectious Diseases. 2020;26(4):648-832.
62. Domínguez MC, Vergara S, Gómez MC, Roldán ME. Epidemiology of tick-borne relapsing fever in endemic area, Spain. Emerging Infectious Diseases. 2020;26(5):849-56.
63. Delaney SL, Murray LA, Aasen CE, Bennett CE, Brown E, Fallon BA. Borrelia miyamotoi Serology in a Clinical Population With Persistent Symptoms and Suspected Tick-Borne Illness. Frontiers in Medicine. 2020;7.
64. Dahmana H, Granjon L, Diagne C, Davoust B, Fenollar F, Mediannikov O. Rodents as Hosts of Pathogens and Related Zoonotic Disease Risk. Pathogens. 2020;9(3).
65. Cotes-Perdomo AP, Oviedo Á, Castro LR. Molecular detection of pathogens in ticks associated with domestic animals from the Colombian Caribbean region. Experimental and Applied Acarology. 2020;82(1):137-50.
66. Costa FB, Martins TF, Muñoz-Leal S, de Azevedo Serpa MC, Ogrzewalska M, Luz HR, et al. Retrospective and new records of ticks (Acari: Argasidae, Ixodidae) from the state of Maranhão, an Amazon-Cerrado transition area of Brazil. Veterinary Parasitology: Regional Studies and Reports. 2020;21.
67. Colunga-Salas P, Sánchez-Montes S, Volkow P, Ruíz-Remigio A, Becker I. Lyme disease and relapsing fever in Mexico: An overview of human and wildlife infections. PLoS ONE. 2020;15(9 September).
68. Cleveland CA, Swanepoel L, Brown JD, Casalena MJ, Williams L, Yabsley MJ. Surveillance for Borrelia spp. in upland game birds in Pennsylvania, USA. Veterinary Sciences. 2020;7(3).
69. Capligina V, Seleznova M, Akopjana S, Freimane L, Lazovska M, Krumins R, et al. Large-scale countrywide screening for tick-borne pathogens in field-collected ticks in Latvia during 2017-2019. Parasites and Vectors. 2020;13(1).
70. Boyer PH, Koetsveld J, Zilliox L, Sprong H, Talagrand-Reboul É, Hansmann Y, et al. Assessment of Borrelia miyamotoi in febrile patients and ticks in Alsace, an endemic area for Lyme borreliosis in France. Parasites and Vectors. 2020;13(1).

71. Binetruy F, Garnier S, Boulanger N, Talagrand-Reboul É, Loire E, Faivre B, et al. A novel Borrelia species, intermediate between Lyme disease and relapsing fever groups, in neotropical passerine-associated ticks. Scientific reports. 2020;10(1):10596.
72. Binenbaum Y, Ben-Ami R, Baneth G, Langford B, Negev Y, Friedlander E, et al. Single dose of doxycycline for the prevention of tick-borne relapsing fever. Clinical Infectious Diseases. 2020;71(7):1768-71.
73. Barraza-Guerrero SI, Meza-Herrera CA, García-De la Peña C, González-Álvarez VH, Vaca-Paniagua F, Díaz-Velásquez CE, et al. General Microbiota of the Soft Tick Ornithodoros turicata Parasitizing the Bolson Tortoise (Gopherus flavomarginatus) in the Mapimi Biosphere Reserve, Mexico. Biology (Basel). 2020;9(9).
74. Aviles ES, Oakes M, Algranati M, Mansoor AM. Tick-borne relapsing fever. BMJ case reports. 2020;13(7).
75. Xu G, Pearson P, Dykstra E, Andrews ES, Rich SM. Human-Biting Ixodes Ticks and Pathogen Prevalence from California, Oregon, and Washington. Vector Borne Zoonotic Dis. 2019;19(2):106-14.
76. Wormser GP, Shapiro ED, Fish D. Borrelia miyamotoi: An Emerging Tick-Borne Pathogen. American Journal of Medicine. 2019;132(2):136-7.
77. Werner SL, Banda BK, Burnsides CL, Stuber AJ. Zoonosis: Update on Existing and Emerging Vector-Borne Illnesses in the USA. Curr Emerg Hosp Med Rep. 2019;7(3):91-106.
78. Vázquez-Guerrero E, Adan-Bante NP, Mercado-Uribe MC, Hernández-Rodríguez C, Villa-Tanaca L, Lopez JE, et al. Case report: A retrospective serological analysis indicating human exposure to tickborne relapsing fever spirochetes in Sonora, Mexico. PLoS Neglected Tropical Diseases. 2019;13(4).
79. Vaculová T, Derdáková M, Špitalská E, Václav R, Chvostáč M, Rusňáková Tarageľová V. Simultaneous Occurrence of Borrelia miyamotoi, Borrelia burgdorferi Sensu Lato, Anaplasma phagocytophilum and Rickettsia helvetica in Ixodes ricinus Ticks in Urban Foci in Bratislava, Slovakia. Acta Parasitologica. 2019;64(1):19-30.
80. Szekeres S, Docters van Leeuwen A, Tóth E, Majoros G, Sprong H, Földvári G. Road-killed mammals provide insight into tick-borne bacterial pathogen communities within urban habitats. Transboundary and Emerging Diseases. 2019;66(1):277-86.
81. Sun Y, Xu R, Liu Z, Wu M, Qin T. Ornithodoros (Ornithodoros) huajianensis sp. nov. (Acari, argasidae), a new tick species from the Mongolian marmot (Marmota bobak sibirica), Gansu province in China. Int J Parasitol Parasites Wildl. 2019;9:209-17.
82. Smith RP, Elias SP, Cavanaugh CE, Lubelczyk CB, Lacombe EH, Brancato J, et al. Seroprevalence of Borrelia burgdorferi, B. miyamotoi, and Powassan Virus in Residents Bitten by Ixodes Ticks, Maine, USA. Emerg Infect Dis. 2019;25(4):804-7.
83. Shah JS, Liu S, Du Cruz I, Poruri A, Maynard R, Shkilna M, et al. Line Immunoblot Assay for Tick-Borne Relapsing Fever and Findings in Patient Sera from Australia, Ukraine and the USA. Healthcare (Basel). 2019;7(4).
84. Sanseverino L, Ortencio HF, Esteve-Gassent M, Jorge TMR. Test for Borrelia spp. in bats in an urban area in the South of Brazil. Figshare; 2019.
85. Sanchez-Vicente S, Tagliafierro T, Coleman JL, Benach JL, Tokarz R, Azad A, et al. Polymicrobial Nature of Tick-Borne Diseases. mBio. 2019;10(5).
86. Rudakova SA, Teslova OE, Kaneshova NE, Shtrek SV, Yakimenko VV, Penyevskaya NA. Genospecies diversity of borrelia in ixodes ticks of the West Siberia. Problemy Osobo Opasnykh Infektsii. 2019;2019(4):92-6.

87. Remesar S, Díaz P, Venzal JM, Prieto A, Estrada-Peña A, López CM, et al. Longitudinal Study of Infection with Borrelia spp. in Questing Ticks from North-Western Spain. Vector-Borne and Zoonotic Diseases. 2019;19(11):785-92.
88. Rar V, Livanova N, Sabitova Y, Igolkina Y, Tkachev S, Tikunov A, et al. Ixodes persulcatus/pavlovskyi natural hybrids in Siberia: Occurrence in sympatric areas and infection by a wide range of tick-transmitted agents. Ticks and Tick-borne Diseases. 2019;10(6).
89. Rampes S, Yates M, Galloway J. Tick-borne relapsing fever: A fever syndrome mimic. Rheumatology Advances in Practice. 2019;3:i23-i4.
90. Qurollo B. Feline Vector-Borne Diseases in North America. Veterinary Clinics of North America - Small Animal Practice. 2019;49(4):687-702.
91. Qiu Y, Nakao R, Hang'ombe BM, Sato K, Kajihara M, Kanchela S, et al. Human Borreliosis Caused by a New World Relapsing Fever Borrelia– like Organism in the Old World. Clinical Infectious Diseases. 2019;69(1):107-12.
92. Pukhovskaya NM, Morozova OV, Vysochina NP, Belozerova NB, Ivanov LI. Prevalence of Borrelia burgdorferi sensu lato and Borrelia miyamotoi in ixodid ticks in the Far East of Russia. International Journal for Parasitology: Parasites and Wildlife. 2019;8:192-202.
93. Petney TN, Saijuntha W, Boulanger N, Chitimia-Dobler L, Pfeffer M, Eamudomkarn C, et al. Ticks (Argasidae, Ixodidae) and tick-borne diseases of continental Southeast Asia. Zootaxa. 2019;4558(1):1-89.
94. Pacheco A, Cordeiro MD, Cepeda MB, Luz HR, Cardozo SV, Berto BP, et al. Hemoparasites in ticks of wild birds of serra dos Órgãos national park, state of Rio de Janeiro, Brazil. Revista Brasileira de Parasitologia Veterinaria. 2019;28(2):238-44.
95. Osborne CJ, Crosbie PR, Van Laar TA. Borrelia parkeri in Ornithodoros parkeri (Ixodida: Argasidae) Collected Using Compact Dry Ice Traps in Madera County, California. Journal of medical entomology. 2019;56(2):579-83.
96. Namina A, Capligina V, Seleznova M, Krumins R, Aleinikova D, Kivrane A, et al. Tick-borne pathogens in ticks collected from dogs, Latvia, 2011–2016. BMC Vet Res. 2019;15.
97. Muñoz-Leal S, Marcili A, Fuentes-Castillo D, Ayala M, Labruna MB. A relapsing fever Borrelia and spotted fever Rickettsia in ticks from an Andean valley, central Chile. Exp Appl Acarol. 2019;78(3):403-20.
98. Muñoz-Leal S, Macedo C, Gonçalves TC, Dias Barreira J, Labruna MB, de Lemos ERS, et al. Detected microorganisms and new geographic records of Ornithodoros rietcorreai (Acari: Argasidae) from northern Brazil. Ticks and Tick-borne Diseases. 2019;10(4):853-61.
99. Muñoz-Leal S, Lopes MG, Marcili A, Martins TF, González-Acuña D, Labruna MB. Anaplasmataceae, Borrelia and Hepatozoon agents in ticks (Acari: Argasidae, Ixodidae) from Chile. Acta Tropica. 2019;192:91-103.
100. Morel N, De Salvo MN, Cicuttin G, Rossner V, Thompson CS, Mangold AJ, et al. The presence of Borrelia theileri in Argentina. Veterinary Parasitology: Regional Studies and Reports. 2019;17.
101. Modarelli JJ, Tomeček JM, Piccione J, Ferro PJ, Esteve-Gasent MD. Molecular prevalence and ecoregion distribution of select tick-borne pathogens in Texas dogs. Transboundary and Emerging Diseases. 2019;66(3):1291-300.
102. Modarelli JJ, Piccione J, Ferro PJ, Esteve-Gasent MD. Novel real-time PCR assays for genomic group identification of tick-borne relapsing fever species Borrelia hermsii. Diagnostic Microbiology and Infectious Disease. 2019;93(1):24-9.

103. Modarelli JJ, Ferro PJ, de León AAP, Esteve-Gasent MD. TickPath Layerplex: adaptation of a real-time PCR methodology for the simultaneous detection and molecular surveillance of tick-borne pathogens. Sci Rep. 2019;9.
104. Malincarne L, Schiaroli E, Ciervo A, Scaglione V, Paciaroni M, Mancini F, et al. Meningitis with cranial polyneuritis and cavernous sinus thrombosis by Borrelia crocidurae: First autochthonous case in Europe. International Journal of Infectious Diseases. 2019;82:30-2.
105. Mafi N, Yaglom HD, Levy C, Taylor A, O'Grady C, Venkat H, et al. Tick-Borne Relapsing Fever in the White Mountains, Arizona, USA, 2013–2018. Emerg Infect Dis. 2019;25(4):649-53.
106. Lernout T, De Regge N, Tersago K, Fonville M, Suin V, Sprong H. Prevalence of pathogens in ticks collected from humans through citizen science in Belgium. Parasit Vectors. 2019;12.
107. Lee SH, Healy JE, Lambert JS. Single core genome sequencing for detection of both borrelia burgdorferi sensu lato and relapsing fever borrelia species. International Journal of Environmental Research and Public Health. 2019;16(10).
108. Lambregts MMC, Bentvelsen RG, Makiello PE, De Wever B, Kuijper EJ, Visser LG. Relapsing fever after traveling in the tropics: A story with a twist. Nederlands Tijdschrift voor Geneeskunde. 2019;163(25).
109. Kuleshov KV, Hoornstra D, Sprong H, Platonov AE, Hovius JW. Draft Whole-Genome Sequences of Two Western European Borrelia miyamotoi Isolates. Microbiol Resour Announc. 2019;8(50).
110. Kubiak K, Dziekońska-Rynko J, Szymańska H, Kubiak D, Dmitryjuk M, Dzika E. Questing Ixodes ricinus ticks (Acari, Ixodidae) as a vector of Borrelia burgdorferi sensu lato and Borrelia miyamotoi in an urban area of north-eastern Poland. Experimental & applied acarology. 2019;78(1):113-26.
111. Krishnavajhala A, Armstrong BA, Lopez JE. Vector Competence of Geographical Populations of Ornithodoros turicata for the Tick-Borne Relapsing Fever Spirochete Borrelia turicatae (vol 84, e01505-18, 2018). Applied and Environmental Microbiology. 2019;85(5):e03091-18.
112. Klitgaard K, Højgaard J, Isbrand A, Madsen JJ, Thorup K, Bødker R. Screening for multiple tick-borne pathogens in Ixodes ricinus ticks from birds in Denmark during spring and autumn migration seasons. Ticks and Tick-borne Diseases. 2019;10(3):546-52.
113. Kim CM, Seo JW, Kim DM, Yun NR, Park JW, Chung JK, et al. Detection of Borrelia miyamotoi in Ixodes nipponensis in Korea. PLoS One. 2019;14(7).
114. Kazemi-Moghaddam V, Dehghani R, Hadei M, Dehqan S, Sedaghat MM, Latifi M, et al. Rodent-borne and rodent-related diseases in Iran. Comparative Clinical Pathology. 2019;28(4):893-905.
115. Kalmár Z, Sándor AD, Matei IA, Ionică A, D'Amico G, Gherman CM, et al. Borrelia spp. in small mammals in Romania. Parasites and Vectors. 2019;12(1).
116. Jaenson TGT, Wilhelmsson P. First records of tick-borne pathogens in populations of the taiga tick Ixodes persulcatus in Sweden. Parasit Vectors. 2019;12.
117. Hosseini-Chegeni A, Tavakoli M, Telmadarraiy Z. The updated list of ticks (Acari: Ixodidae & Argasidae) occurring in Iran with a key to the identification of species. Systematic and Applied Acarology. 2019;24(11):2133-66.
118. Hosseini-Chegeni A, Tavakoli M, Koshki H, Khedri J, Kayedi MH. A new record of Ornithodoros (Pavlovskyella) verrucosus (Acari: Argasidae) from a porcupine burrow in western Iran. Systematic and Applied Acarology. 2019;24(5):771-81.

119. Henningsson AJ, Asgeirsson H, Hammas B, Karlsson E, Parke Å, Hoornstra D, et al. Two Cases of Borrelia miyamotoi Meningitis, Sweden, 2018. Emerg Infect Dis. 2019;25(10):1965-8.
120. Heida J, van Arkel A, Verweij JJ, Tijssen CC. Meningitis due to infection with Borrelia hispanica. Ned Tijdschr Geneeskd. 2019;163.
121. Han S, Lubelczyk C, Hicklingg GJ, Belperron AA, Bockenstedt LK, Tsao JI. Vertical transmission rates of Borrelia miyamotoi in Ixodes scapularis collected from white-tailed deer. Ticks and Tick-Borne Diseases. 2019;10(3):682-9.
122. Guzmán-Cornejo C, Herrera-Mares A, Robbins RG, Rebollo-Hernández A. The soft ticks (Parasitiformes: Ixodida: Argasidae) of Mexico: Species, hosts, and geographical distribution. Zootaxa. 2019;4623(3):485-525.
123. Gras E, Bailly E, Le Brun C, Lemaignen A, Lanotte P. Borrelia crocidurae tick-borne relapsing fever upon return from Senegal. Medecine et Maladies Infectieuses. 2019;49(8):624-5.
124. Fall NS, Diagne N, Mediannikov O, Fenollar F, Parola P, Sokhna C, et al. Detection of Borrelia crocidurae in a vaginal swab after miscarriage, rural Senegal, Western Africa. International Journal of Infectious Diseases. 2019;91:261-3.
125. Eldin C, Jaulhac B, Mediannikov O, Arzouni JP, Raoult D. Values of diagnostic tests for the various species of spirochetes. Medecine et Maladies Infectieuses. 2019;49(2):102-11.
126. Edwards MJ, Russell JC, Davidson EN, Yanushefski TJ, Fleischman BL, Heist RO, et al. A 4-Yr Survey of the Range of Ticks and Tick-Borne Pathogens in the Lehigh Valley Region of Eastern Pennsylvania. J Med Entomol. 2019;56(4):1122-34.
127. Díaz P, Remesar S, Venzal JM, Vázquez-López ME, Fernández G, López C, et al. Occurrence of Borrelia and Borreliella species in Ixodes ricinus collected from roe deer in northwestern Spain. Medical and Veterinary Entomology. 2019;33(3):427-30.
128. Cutler S, Vayssier-Taussat M, Estrada-Peña A, Potkonjak A, Mihalca AD, Zeller H. A new Borrelia on the block: Borrelia miyamotoi – a human health risk? Euro Surveill. 2019;24(18).
129. Cicuttin GL, De Salvo MN, Venzal JM, Nava S. Borrelia spp. in ticks and birds from a protected urban area in Buenos Aires city, Argentina. Ticks and Tick-Borne Diseases. 2019;10(6):1282-.
130. Černý J, Buyannemekh B, Needham T, Gankhuyag G, Oyuntsetseg D. Hard ticks and tick-borne pathogens in Mongolia—A review. Ticks and Tick-borne Diseases. 2019;10(6).
131. Campbell SB, Klioueva A, Taylor J, Nelson C, Tomasi S, Replogle A, et al. Evaluating the risk of tick-borne relapsing fever among occupational cavers-Austin, TX, 2017. Zoonoses Public Health. 2019;66(6):579-86.
132. Buchan BW, Jobe DA, Mashock M, Gerstbrein D, Faron ML, Ledeboer NA, et al. Evaluation of a Novel Multiplex High-Definition PCR Assay for Detection of Tick-Borne Pathogens in Whole-Blood Specimens. J Clin Microbiol. 2019;57(11).
133. Binetruy F, Chevillon C, de Thoisy B, Garnier S, Duron O. Survey of ticks in French Guiana. Ticks and Tick-borne Diseases. 2019;10(1):77-85.
134. Aguirre AAR, Rodrigues VS, Costa IN, Garcia MV, Csordas BG, Andreotti R, et al. Amblyomma scalpturatum neumann, 1906 (Acari: Ixodidae): Confirmation in acre state, Brazil, and description of parasitism in a human. Revista Brasileira de Parasitologia Veterinaria. 2019;28(3):473-8.
135. Abanda B, Paguem A, Abdoulmoumini M, Kingsley MT, Renz A, Eisenbarth A. Molecular identification and prevalence of tick-borne pathogens in zebu and taurine cattle in North Cameroon. Parasit Vectors. 2019;12.

136. Zhai B, Niu Q, Liu Z, Yang J, Pan Y, Li Y, et al. First detection and molecular identification of Borrelia species in Bactrian camel (Camelus bactrianus) from Northwest China. Infection, Genetics and Evolution. 2018;64:149-55.
137. Yousefi A, Rahbari S, Eslami A. Ectoparasites associated with small mammals (orders Insectivora, Eulipotyphla, and Rodentia) in Razan plain, western region of Iran. Comparative Clinical Pathology. 2018;27(3):667-71.
138. Yang Y, Yang Z, Kelly P, Li J, Ren Y, Wang C. Borrelia miyamotoi sensu lato in Père David Deer and Haemaphysalis longicornis Ticks. Emerg Infect Dis. 2018;24(5):928-31.
139. Valle MIGD, Alfaro EM. Infecciones producidas por borrelias: Enfermedad de Lyme y fiebre recurrente. Medicine (Spain). 2018;12(59):3458-64.
140. Tokarz R, Mishra N, Tagliafierro T, Sameroff S, Caciula A, Chauhan L, et al. A multiplex serologic platform for diagnosis of tick-borne diseases. Sci Rep. 2018;8.
141. Talagrand-Reboul E, Boyer PH, Bergström S, Vial L, Boulanger N. Relapsing fevers: Neglected tick-borne diseases. Frontiers in Cellular and Infection Microbiology. 2018;8(APR).
142. Stete K, Rieg S, Margos G, Häcker G, Wagner D, Kern WV, et al. Case report and genetic sequence analysis of candidatus Borrelia Kalaharica, Southern Africa. Emerging Infectious Diseases. 2018;24(9):1659-64.
143. Sonenshine DE. Range Expansion of Tick Disease Vectors in North America: Implications for Spread of Tick-Borne Disease. Int J Environ Res Public Health. 2018;15(3).
144. Schöffel N, Braun M, Volante G, Bendels MHK, Groneberg DA. Relapsing fever borreliosis: A review of the literature. Zentralblatt fur Arbeitsmedizin, Arbeitsschutz und Ergonomie. 2018;68(4):214-7.
145. Savel'eva MV, Krasnova EI, Khokhlova NI, Provorova VV, Filimonova ES, Rar VA, et al. Clinical and laboratory characteristics of diseases caused by Borrelia spp. In the inhabitants of the Novosibirsk region in 2015-2017. Jurnal Infektologii. 2018;10(2):68-75.
146. Sato K, Sakakibara K, Masuzawa T, Ohnishi M, Kawabata H. Case control study: Serological evidence that Borrelia miyamotoi disease occurs nationwide in Japan. Journal of Infection and Chemotherapy. 2018;24(10):828-33.
147. Salkeld DJ, Nieto NC, Bonilla DL, Yoshimizu MH, Padgett KA. Borrelia miyamotoi Infections in Small Mammals, California, USA. Emerg Infect Dis. 2018;24(12):2356-9.
148. Ruyts SC, Tack W, Ampoorter E, Coipan EC, Matthysen E, Heylen D, et al. Year-to-year variation in the density of Ixodes ricinus ticks and the prevalence of the rodent-associated human pathogens Borrelia afzelii and B-miyamotoi in different forest types. Ticks and Tick-Borne Diseases. 2018;9(2):141-5.
149. Rogovskyy A, Batool M, Gillis DC, Holman PJ, Nebogatkin IV, Rogovska YV, et al. Diversity of Borrelia spirochetes and other zoonotic agents in ticks from Kyiv, Ukraine. Ticks and Tick-borne Diseases. 2018;9(2):404-9.
150. Rodriguez-Morales AJ, Bonilla-Aldana DK, Idarraga-Bedoya SE, Garcia-Bustos JJ, Cardona-Ospina JA, Faccini-Martínez Á A. Epidemiology of zoonotic tick-borne diseases in Latin America: Are we just seeing the tip of the iceberg? F1000Res. 2018;7.
151. Ravagnan S, Tomassone L, Montarsi F, Krawczyk AI, Mastrorilli E, Sprong H, et al. First detection of Borrelia miyamotoi in Ixodes ricinus ticks from northern Italy. Parasites and Vectors. 2018;11(1).
152. Piedmonte NP, Shaw SB, Prusinski MA, Fierke MK. Landscape features associated with blacklegged tick (Acari: Ixodidae) density and tick-borne pathogen prevalence at multiple spatial scales in Central New York State. Journal of Medical Entomology. 2018;55(6):1496-508.

153. Pereira A, Parreira R, Cotão AJ, Nunes M, Vieira ML, Azevedo F, et al. Tick-borne bacteria and protozoa detected in ticks collected from domestic animals and wildlife in central and southern Portugal. Ticks and Tick-borne Diseases. 2018;9(2):225-34.
154. Paris DH, Neumayr A. Ticks and tick-borne infections in Asia: Implications for travellers. Travel Medicine and Infectious Disease. 2018;26:3-4.
155. Palomar AM, Portillo A, Santibáñez P, Santibáñez S, Oteo JA. Borrelia miyamotoi: Should this pathogen be considered for the diagnosis of tick-borne infectious diseases in Spain? Enfermedades Infecciosas y Microbiologia Clinica. 2018;36(9):568-71.
156. Page S, Daschkin C, Anniko S, Krey V, Nicolaus C, Maxeiner H-G. First report of Borrelia miyamotoi in an Ixodes ricinus tick in Augsburg, Germany. Experimental and Applied Acarology. 2018;74(2):191-9.
157. Nieto NC, Tanner Porter W, Wachara JC, Lowrey TJ, Martin L, Motyka PJ, et al. Using citizen science to describe the prevalence and distribution of tick bite and exposure to tick-borne diseases in the United States. PLoS ONE. 2018;13(7).
158. Muñoz-Leal S, Terassini FA, Luz HR, Fontana I, Camargo LMA, Labruna MB. First report of Ornithodoros peropteryx in Brazil, and the occurrence of Ornithodoros cavernicolous in the western Brazilian Amazon. Systematic and Applied Acarology. 2018;23(11):2113-21.
159. Muñoz-Leal S, Faccini-Martínez Á A, Costa FB, Marcili A, Mesquita E, Marques EP, Jr., et al. Isolation and molecular characterization of a relapsing fever Borrelia recovered from Ornithodoros rudis in Brazil. Ticks Tick Borne Dis. 2018;9(4):864-71.
160. Middelveen MJ, Shah JS, Fesler MC, Stricker RB. Relapsing fever borrelia in california: A pilot serological study. International Journal of General Medicine. 2018;11:373-82.
161. Marcos L, Smith K, Weinbaum F, Spitzer E. An emerging tick-borne disease in Long Island, New York: Relapsing fever caused by Borrelia miyamotoi. Open Forum Infectious Diseases. 2018;5:S241.
162. MacDonald AJ. Abiotic and habitat drivers of tick vector abundance, diversity, phenology and human encounter risk in southern California. PLoS One. 2018;13(7).
163. Lynn GE, Graham CB, Horiuchi K, Eisen L, Johnson TL, Lane RS, et al. Prevalence and Geographic Distribution of Borrelia miyamotoi in Host-Seeking Ixodes pacificus (Acari: Ixodidae) Nymphs in Mendocino County, California. J Med Entomol. 2018;55(3):711-6.
164. Layzell SJ, Bailey D, Peacey M, Nuttall PA. Prevalence of Borrelia burgdorferi and Borrelia miyamotoi in questing Ixodes ricinus ticks from four sites in the UK. Ticks and Tick-Borne Diseases. 2018;9(2):217-24.
165. Lafri I, Benredjem W, Neffah-Baaziz F, Lalout R, Abdelouahed K, Gassen B, et al. Inventory and update on argasid ticks and associated pathogens in Algeria. New Microbes and New Infections. 2018;23:110-4.
166. Laaksonen M, Klemola T, Feuth E, Sormunen JJ, Puisto A, Mäkelä S, et al. Tick-borne pathogens in Finland: comparison of Ixodes ricinus and I. persulcatus in sympatric and parapatric areas. Parasit Vectors. 2018;11.
167. Kumagai Y, Sato K, Taylor KR, Zamoto-Niikura A, Imaoka K, Morikawa S, et al. A relapsing fever group Borrelia sp. is widely distributed among wild deer in Japan. Ticks and Tick-borne Diseases. 2018;9(3):465-70.
168. Kulkarni M, Kryuchkov R, Statculescu A, Thickstun C, Dibernardo A, Lindsay L, et al. tick distribution and infection rates in Ottawa, Ontario, 2017. Canada Communicable Disease Report. 2018;44(10):237-42.

169. Krause PJ, Carroll M, Fedorova N, Brancato J, Dumouchel C, Akosa F, et al. Human Borrelia miyamotoi infection in California: Serodiagnosis is complicated by multiple endemic Borrelia species. PLoS ONE. 2018;13(2).
170. Koton Y, Bisharat N. Tick-borne relapsing fever with severe Jarisch–Herxheimer reaction. Israel Medical Association Journal. 2018;20(1):62-3.
171. Kingry LC, Anacker M, Pritt B, Bjork J, Respicio-Kingry L, Liu GP, et al. Surveillance for and Discovery of Borrelia Species in US Patients Suspected of Tickborne Illness. Clinical Infectious Diseases. 2018;66(12):1864-71.
172. Kim HC, Park JG, Kwon YS, Kim M, Park CU, Yun SM, et al. Ticks collected from soil/nest litter and live and dead nestlings of migratory seabirds during their breeding season at six uninhabited Islands, Republic of Korea during 2009 and 2014-2017. Systematic and Applied Acarology. 2018;23(8):1519-30.
173. Karan L, Makenov M, Kolyasnikova N, Stukolova O, Toporkova M, Olenkova O. Dynamics of Spirochetemia and Early PCR Detection of Borrelia miyamotoi. Emerg Infect Dis. 2018;24(5):860-7.
174. Kahouli S, Naoui H, Uwingabiye J, Reggad A, Ennibi K, Bouchrik M, et al. Relapsing fever in a Moroccan man. Med Sante Trop. 2018;28(2):141-3.
175. Johnson TL, Graham CB, Maes SE, Hojgaard A, Fleshman A, Boegler KA, et al. Prevalence and distribution of seven human pathogens in host-seeking Ixodes scapularis (Acari: Ixodidae) nymphs in Minnesota, USA. Ticks Tick Borne Dis. 2018;9(6):1499-507.
176. Jiang BG, Jia N, Jiang JF, Zheng YC, Chu YL, Jiang RR, et al. Borrelia miyamotoi Infections in Humans and Ticks, Northeastern China. Emerg Infect Dis. 2018;24(2):236-41.
177. Hoornstra D, Koetsveld J, Sprong H, Platonov AE, Hovius JW. Borrelia miyamotoi disease in an immunocompetent patient, Western Europe. Emerging Infectious Diseases. 2018;24(9):1770-2.
178. Halperin JJ, García-Moncó JC. The human borreliosis: Lyme neuroborreliosis and relapsing fever. CNS Infections: A Clinical Approach: Second Edition2018. p. 233-69.
179. Guiheneuf E, Desjardins N, Guiheneuf R. It is not always malaria: diagnosis of Borrelia recurrent fever on blood smear. Annales de biologie clinique. 2018;76(1):118-9.
180. Goddard J, Goddard J. Tick-Borne Diseases2018. 91-147 p.
181. Faccini-Martínez ÁA, Marco González T, Salim Mattar V. Tick-borne relapsing fever: Another underdiagnosed etiology in tropical Latin America? Revista MVZ Cordoba. 2018;23(1):6399-402.
182. Estrada-Peña A, Álvarez-Jarreta J, Cabezas-Cruz A. Reservoir and vector evolutionary pressures shaped the adaptation of Borrelia. Infection, Genetics and Evolution. 2018;66:308-18.
183. Elelu N. Tick-borne relapsing fever as a potential veterinary medical problem. Veterinary medicine and science. 2018;4(4):271-9.
184. Eldin C, Parola P. Update on Tick-Borne Bacterial Diseases in Travelers. Current Infectious Disease Reports. 2018;20(7).
185. Eisen RJ, Eisen L. The Blacklegged Tick, Ixodes scapularis: An Increasing Public Health Concern. Trends Parasitol. 2018;34(4):295-309.
186. Eisen L. Pathogen transmission in relation to duration of attachment by Ixodes scapularis ticks. Ticks and Tick-borne Diseases. 2018;9(3):535-42.
187. Egizi A, Roegner V, Faraji A, Healy SP, Schulze TL, Jordan RA. A historical snapshot of Ixodes scapularis-borne pathogens in New Jersey ticks reflects a changing disease landscape. Ticks and Tick-borne Diseases. 2018;9(2):418-26.

188. de Oliveira SV, Bitencourth K, Borsoi ABP, de Freitas FSS, Castelo Branco Coelho G, Amorim M, et al. Human parasitism and toxicosis by Ornithodoros rietcorreai (Acari: Argasidae) in an urban area of Northeastern Brazil. Ticks and Tick-borne Diseases. 2018;9(6):1494-8.
189. Cutler SJ, Idris JM, Ahmed AO, Elelu N. Ornithodoros savignyi, the tick vector of “candidatus borrelia kalaharica” in Nigeria. Journal of Clinical Microbiology. 2018;56(9).
190. Bryant K. Tickborne Infections. Principles and Practice of Pediatric Infectious Diseases2018. p. 542-6.e2.
191. Bissett JD, Ledet S, Krishnavajhala A, Armstrong BA, Klioueva A, Sexton C, et al. Detection of tickborne relapsing fever Spirochete, Austin, Texas, USA. Emerging Infectious Diseases. 2018;24(11):2003-9.
192. Bernard Q, Helezen E, Boulanger N. Tick-Borne Bacteria and Host Skin Interface. Skin and Arthropod Vectors2018. p. 293-324.
193. Bergström S, Normark J. Microbiological features distinguishing Lyme disease and relapsing fever spirochetes. Wiener Klinische Wochenschrift. 2018;130(15-16):484-90.
194. Banafshi O, Hanafi-Bojd AA, Karimi M, Faghihi F, Beik-Mohammadi M, Gholami S, et al. Tick Ectoparasites of Animals in Borderline of Iran-Iraq and Their Role on Disease Transmission. J Arthropod Borne Dis. 2018;12(3):252-61.
195. Armstrong BA, Kneubehl A, Krishnavajhala A, Wilder HK, Boyle W, Wozniak E, et al. Seroprevalence for the tick-borne relapsing fever spirochete Borrelia turicatae among small and medium sized mammals of Texas. PLoS Neglected Tropical Diseases. 2018;12(10).
196. Andersson MO, Marga G, Banu T, Dobler G, Chitimia-Dobler L. Tick-borne pathogens in tick species infesting humans in Sibiu County, central Romania. Parasitology Research. 2018;117(5):1591-7.
197. Yamano K, Ito T, Kiyanagi K, Yamazaki H, Sugawara M, Saito T, et al. Case report: Clinical features of a case of suspected borrelia miyamotoi disease in Hokkaido, Japan. American Journal of Tropical Medicine and Hygiene. 2017;97(1):84-7.
198. Wroblewski D, Gebhardt L, Prusinski MA, Meehan LJ, Halse TA, Musser KA. Detection of Borrelia miyamotoi and other tick-borne pathogens in human clinical specimens and Ixodes scapularis ticks in New York State, 2012-2015. Ticks and Tick-Borne Diseases. 2017;8(3):407-11.
199. Wagemakers A, Jahfari S, de Wever B, Spanjaard L, Starink MV, de Vries HJC, et al. Borrelia miyamotoi in vectors and hosts in The Netherlands. Ticks and Tick-borne Diseases. 2017;8(3):370-4.
200. Szekeres S, Lügner J, Fingerle V, Margos G, Földvári G. Prevalence of Borrelia miyamotoi and Borrelia burgdorferi sensu lato in questing ticks from a recreational coniferous forest of East Saxony, Germany. Ticks and Tick-borne Diseases. 2017;8(6):922-7.
201. Straub MH, Roy AN, Martin A, Sholty KE, Stephenson N, Foley JE. Distribution and prevalence of vector-borne diseases in California chipmunks (Tamias spp.). PLoS ONE. 2017;12(12).
202. Shapiro ED. Other Borrelia Species and Spirillum minus. Principles and Practice of Pediatric Infectious Diseases2017. p. 985-7.
203. Shannon AB, Rucinsky R, Gaff HD, Brinkerhoff RJ. Borrelia miyamotoi, Other Vector-Borne Agents in Cat Blood and Ticks in Eastern Maryland. Ecohealth. 2017;14(4):816-20.
204. Scott JD, Foley JE, Young MR, Durden LA. First report of a blacklegged tick, Ixodes scapularis Say (Acari: Ixodidae), parasitizing a raptor in Canada. Systematic and Applied Acarology. 2017;22(2):208-16.

205. Sándor S, András L, Gábor F. Borrelia miyamotoi: A recently identified human pathogenic tick-borne relapsing fever spirochete. Orvosi Hetilap. 2017;158(29):1124-30.
206. Ruyts SC, Frazer-Mendelewska E, Van Den Berge K, Verheyen K, Sprong H. Molecular detection of tick-borne pathogens Borrelia afzelii, Borrelia miyamotoi and Anaplasma phagocytophilum in Eurasian red squirrels (Sciurus vulgaris). European Journal of Wildlife Research. 2017;63(3).
207. Rar V, Livanova N, Tkachev S, Kaverina G, Tikunov A, Sabitova Y, et al. Detection and genetic characterization of a wide range of infectious agents in Ixodes pavlovskyi ticks in Western Siberia, Russia. Parasit Vectors. 2017;10.
208. Raileanu C, Moutailler S, Pavel I, Porea D, Mihalca AD, Savuta G, et al. Borrelia Diversity and Co-infection with Other Tick Borne Pathogens in Ticks. Front Cell Infect Microbiol. 2017;7.
209. Platonov AE, Toporkova MG, Kolyasnikova NM, Stukolova OA, Dolgova AS, Brodovikova AV, et al. Clinical presentation of Ixodes tick-borne borreliosis caused by Borrelia miyamotoi in the context of an immune response to the pathogen. Ter Arkh. 2017;89(11):35-43.
210. Panetta JL, Šíma R, Calvani NED, Hajdušek O, Chandra S, Panuccio J, et al. Reptile-associated Borrelia species in the goanna tick (Bothriocroton undatum) from Sydney, Australia. Parasit Vectors. 2017;10.
211. Oechslin CP, Heutschi D, Lenz N, Tischhauser W, Péter O, Rais O, et al. Prevalence of tick-borne pathogens in questing Ixodes ricinus ticks in urban and suburban areas of Switzerland. Parasit Vectors. 2017;10.
212. Oda R, Kutsuna S, Sekikawa Y, Hongo I, Sato K, Ohnishi M, et al. The first case of imported Borrelia miyamotoi disease concurrent with Lyme disease. Journal of Infection and Chemotherapy. 2017;23(5-6):333-5.
213. Nava S, Venzal JM, González-Acuña D, Martins TF, Guglielmone AA. Ticks of the Southern Cone of America: Diagnosis, Distribution, and Hosts with Taxonomy, Ecology and Sanitary Importance2017. 1-348 p.
214. Naddaf SR, Ghazinezhad B, Kazemirad E, Cutler SJ. Relapsing fever causative agent in Southern Iran is a closely related species to East African borreliae. Ticks and Tick-borne Diseases. 2017;8(6):882-6.
215. Muñoz-Leal S, Toledo LF, Venzal JM, Marcili A, Martins TF, Acosta ICL, et al. Description of a new soft tick species (Acari: Argasidae: Ornithodoros) associated with stream-breeding frogs (Anura: Cycloramphidae: Cycloramphus) in Brazil. Ticks and Tick-borne Diseases. 2017;8(5):682-92.
216. Morshed MG, Drews SJ, Lee MK, Fernando K, Man S, Mak S, et al. Tick-borne relapsing fever in british Columbia: A 10-year review (2006–2015). British Columbia Medical Journal. 2017;59(8):412-7.
217. Leen I, Bruynseels P, Mukadi BK, Van Oort M, Van Den Akker M. A 13-year old girl with pancytopenia at the presentation of a Borrelia hispanica infection: A case report and review of the literature. Journal of Medical Case Reports. 2017;11(1).
218. Lafri I, El Hamzaoui B, Bitam I, Leulmi H, Lalout R, Mediannikov O, et al. Detection of relapsing fever Borrelia spp., Bartonella spp. and Anaplasmataceae bacteria in argasid ticks in Algeria. PLoS Neglected Tropical Diseases. 2017;11(11).
219. Laaksonen M, Sajanti E, Sormunen JJ, Penttinen R, Hänninen J, Ruohomäki K, et al. Crowdsourcing-based nationwide tick collection reveals the distribution of Ixodes ricinus and I. persulcatus and associated pathogens in Finland. Emerg Microbes Infect. 2017;6(5):e31-.

220. Kowalec M, Szewczyk T, Welc-Falęciak R, Siński E, Karbowiak G, Bajer A. Ticks and the city - are there any differences between city parks and natural forests in terms of tick abundance and prevalence of spirochaetes? Parasit Vectors. 2017;10.
221. Koetsveld J, Draga ROP, Wagemakers A, Manger A, Oei A, Visser CE, et al. In vitro susceptibility of the relapsing-fever spirochete borrelia miyamotoi to antimicrobial agents. Antimicrobial Agents and Chemotherapy. 2017;61(9).
222. Kleinerman G, King R, Nachum-Biala Y, Baneth G. Borrelia persica infection in rock hyraxes. Ticks and Tick-borne Diseases. 2017;9(2):382-8.
223. Kim HC, Park CU, Kim M, Kim YM, Yeo J, Kwon YS, et al. Ornithodoros sawaii (Acari: Ixodida: Argasidae) collected from nest soil/litter of Synthliboramphus antiquus, Synthliboramphus wumizusume, and nest soil/litter and nestlings of Hydrobates monorhis on Gugul and Sogugul Islands, Jeollanam Province, Republic of Korea. Systematic and Applied Acarology. 2017;22(11):1959-69.
224. Kim HC, Kim M, Kwon YS, Hong MJ, Park CU, Yun SM, et al. New distribution and host records for Ornithodoros capensis Neumann and Ornithodoros sawaii Kitaoka and Suzuki (Acari: Ixodida: Argasidae) collected from Black-tailed Gull, Larus crassirostris, nestlings and nest soil and litter on Hong and Nan Islands, Republic of Korea. Systematic and Applied Acarology. 2017;22(11):1899-909.
225. Khoo JJ, Lim FS, Tan KK, Chen FS, Phoon WH, Khor CS, et al. Detection in Malaysia of a Borrelia sp. From Haemaphysalis hystricis (Ixodida: Ixodidae). Journal of medical entomology. 2017;54(5):1444-8.
226. Kadkhoda K, Dumouchel C, Brancato J, Gretchen A, Krause PJ. Human seroprevalence of Borrelia miyamotoi in Manitoba, Canada, in 2011-2014: a cross-sectional study. CMAJ Open. 2017;5(3):E690-e3.
227. Jahfari S, Ruyts SC, Frazer-Mendelewska E, Jaarsma R, Verheyen K, Sprong H. Melting pot of tick-borne zoonoses: the European hedgehog contributes to the maintenance of various tick-borne diseases in natural cycles urban and suburban areas. Parasit Vectors. 2017;10.
228. Iwabu-Itoh Y, Bazartseren B, Naranbaatar O, Yondonjamts E, Furuno K, Lee K, et al. Tick surveillance for Borrelia miyamotoi and phylogenetic analysis of isolates in Mongolia and Japan. Ticks and Tick-Borne Diseases. 2017;8(6):850-7.
229. Hosseini-Chegeni A, Telmadarraiy Z, Tavakoli M, Faghihi F. Molecular detection of Borrelia anserina in Argas persicus (Acari: Argasidae) ticks collected from Lorestan province, west of Iran. Persian Journal of Acarology. 2017;6(4):287-97.
230. Honig V, Carolan HE, Vavruskova Z, Massire C, Mosel MR, Crowder CD, et al. Broad-range survey of vector-borne pathogens and tick host identification of Ixodes ricinus from Southern Czech Republic. FEMS Microbiol Ecol. 2017;93(11).
231. Heylen D, Fonville M, Docters van Leeuwen A, Stroo A, Duisterwinkel M, van Wieren S, et al. Pathogen communities of songbird-derived ticks in Europe's low countries. Parasit Vectors. 2017;10.
232. Hamšíková Z, Coipan C, Mahríková L, Minichová L, Sprong H, Kazimírová M. Borrelia miyamotoi and Co-Infection with Borrelia afzelii in Ixodes ricinus Ticks and Rodents from Slovakia. Microbial ecology. 2017;73(4):1000-8.
233. Hall JL, Alpers K, Bown KJ, Martin SJ, Birtles RJ. Use of Mass-Participation Outdoor Events to Assess Human Exposure to Tickborne Pathogens. Emerg Infect Dis. 2017;23(3):463-7.

234. Furuno K, Lee K, Itoh Y, Suzuki K, Yonemitsu K, Kuwata R, et al. Epidemiological study of relapsing fever borreliae detected in Haemaphysalis ticks and wild animals in the western part of Japan. PLoS ONE. 2017;12(3).
235. Fong IW. Emergence of New Tickborne Infections. Emerging Zoonoses: A Worldwide Perspective. Emerging Infectious Diseases of the 21st Century2017. p. 81-100.
236. Fiorito TM, Reece R, Flanigan TP, Silverblatt FJ. Borrelia miyamotoi Polymerase Chain Reaction Positivity on a Tick-Borne Disease Panel in an Endemic Region of Rhode Island: A Case Series. Infectious Diseases in Clinical Practice. 2017;25(5):250-4.
237. Fiecek B, Chmielewski T, Tylewska-Wierzbanowska S. Borrelia miyamotoi - new etiologic agent of neuroborreliosis? Przeglad epidemiologiczny. 2017;71(4):531-8.
238. Esteve-Gasent MD, Snell CB, Adetunji SA, Piccione J. Serological detection of Tick-Borne Relapsing Fever in Texan domestic dogs. PLoS ONE. 2017;12(12).
239. Eisen RJ, Kugeler KJ, Eisen L, Beard CB, Paddock CD. Tick-Borne Zoonoses in the United States: Persistent and Emerging Threats to Human Health. Ilar j. 2017;58(3):319-35.
240. Eickhoff C, Blaylock J. Tickborne diseases other than Lyme in the United States. Cleveland Clinic Journal of Medicine. 2017;84(7):555-67.
241. Ehounoud CB, Fenollar F, Dahmani M, N'Guessan JD, Raoult D, Mediannikov O. Bacterial arthropod-borne diseases in West Africa. Acta Tropica. 2017;171:124-37.
242. Dupraz M, Toty C, Devillers E, Blanchon T, Elguero E, Vittecoq M, et al. Population structure of the soft tick Ornithodoros maritimus and its associated infectious agents within a colony of its seabird host Larus michahellis. Int J Parasitol Parasites Wildl. 2017;6(2):122-30.
243. Díaz P, Arnal JL, Remesar S, Pérez-Creo A, Venzal JM, Vázquez-López ME, et al. Molecular identification of Borrelia spirochetes in questing Ixodes ricinus from northwestern Spain. Parasites and Vectors. 2017;10(1).
244. Diallo MA, Kane BS, Ndiaye M, Dieng M, Diongue K, Badiane AS, et al. Plasmodium falciparum malaria co-infection with tick-borne relapsing fever in Dakar. Malaria Journal. 2017;16(1):1-3.
245. Davari B, Alam FN, Nasirian H, Nazari M, Abdigoudarzi M, Salehzadeh A. Seasonal distribution and faunistic of ticks in the Alashtar county (Lorestan Province), Iran. Pan Afr Med J. 2017;27.
246. Cutler SJ, Rudenko N, Golovchenko M, Cramaro WJ, Kirpach J, Savic S, et al. Diagnosing Borreliosis. Vector-Borne and Zoonotic Diseases. 2017;17(1):2-11.
247. Christensen AM, Pietralczyk E, Lopez JE, Brooks C, Schriefer ME, Wozniak E, et al. Diagnosis and Management of Borrelia turicatae Infection in Febrile Soldier, Texas, USA. Emerg Infect Dis. 2017;23(5):883-4.
248. Butler T. The Jarisch-Herxheimer reaction after antibiotic treatment of spirochetal infections: A review of recent cases and our understanding of pathogenesis. American Journal of Tropical Medicine and Hygiene. 2017;96(1):46-52.
249. Brenner NE, Dolan MC, Replogle AJ, Sexton C, Hojgaard A, Boegler KA, et al. Transmission of Borrelia miyamotoi sensu lato relapsing fever group spirochetes in relation to duration of attachment by Ixodes scapularis nymphs. Ticks and Tick-Borne Diseases. 2017;8(5):677-81.
250. Borde JP, Dobler G, Rieg S. Update on Tick-Borne Diseases in Germany. Deutsche Medizinische Wochenschrift. 2017;142(11):805-10.
251. Bermúdez SE, Castillo E, Pohlenz TD, Kneubehl A, Krishnavajhala A, Domínguez L, et al. New records of Ornithodoros puertoricensis Fox 1947 (Ixodida: Argasidae) parasitizing

humans in rural and urban dwellings, Panama. Ticks and Tick-borne Diseases. 2017;8(4):466-9.
252. Barbour AG, Adeolu M, Gupta RS. Division of the genus Borrelia into two genera (Corresponding to lyme disease and relapsing fever groups) reflects their genetic and phenotypic distinctiveness and will lead to a better understanding of these two groups of microbes (margos et al. (2016) there is inadequate evidence to support the division of the genus borrelia. int. j. syst. evol. microbiol. doi: 10.1099/ ijsem.0.001717). International Journal of Systematic and Evolutionary Microbiology. 2017;67(6):2058-67.
253. Balakrishnan N. Current Scenario of Tick-Borne Diseases in India - A Review. Journal of Communicable Diseases. 2017;49(2):6-13.
254. Wodecka B, Skotarczak B. Identification of host blood-meal sources and Borrelia in field-collected Ixodes ricinus ticks in north-western Poland. Annals of Agricultural and Environmental Medicine. 2016;23(1):59-63.
255. Wodecka B, Michalik J, Lane RS, Nowak-Chmura M, Wierzbicka A. Differential associations of Borrelia species with European badgers (Meles meles) and raccoon dogs (Nyctereutes procyonoides) in western Poland. Ticks and Tick-borne Diseases. 2016;7(5):1010-6.
256. Wieser A, Loscher T, Schunk M, Seilmaier M, Balzer L, Margos G, et al. Relapsing fever: An almost forgotten disease in focus again. [German]. Deutsche Medizinische Wochenschrift. 2016;141(14):1009-13.
257. Venczel R, Knoke L, Pavlovic M, Dzaferovic E, Vaculova T, Silaghi C, et al. A novel duplex real-time PCR permits simultaneous detection and differentiation of Borrelia miyamotoi and Borrelia burgdorferi sensu lato. Infection. 2016;44(1):47-55.
258. Vanstreels RET, Braga EM, Catao-Dias JL. Blood parasites of penguins: a critical review. Parasitology. 2016;143(8):931-56.
259. Torreggiani S, Filocamo G, Esposito S. Recurrent fever in children. International Journal of Molecular Sciences. 2016;17(4).
260. Telmadarraiy Z, Chinikar S, Hashemi jabber K, Yaryan M, Roohnavaz M, Jalali T, et al. Tick infestation rate of sheep, goat and distribution of ticks in Bashagard District, Hormozgan Province, Iran during year 2012. International Journal of Medical Microbiology. 2016;306(8):135.
261. Taylor AJ, Vongphayloth K, Vongsouvath M, Grandadam M, Brey PT, Newton PN, et al. Large-Scale Survey for Tickborne Bacteria, Khammouan Province, Laos. Emerg Infect Dis. 2016;22(9):1635-9.
262. Tadin A, Tokarz R, Markotic A, Margaletic J, Turk N, Habu J, et al. Molecular survey of zoonotic agents in rodents and other small mammals in Croatia. American Journal of Tropical Medicine and Hygiene. 2016;94(2):466-73.
263. Sudhindra P, Wang G, Schriefer ME, McKenna D, Jian Z, Krause PJ, et al. Insights into Borrelia miyamotoi infection from an untreated case demonstrating relapsing fever, monocytosis and a positive C6 Lyme serology. Diagnostic Microbiology and Infectious Disease. 2016;86(1):93-6.
264. Sormunen JJ, Penttinen R, Klemola T, Hänninen J, Vuorinen I, Laaksonen M, et al. Tick-borne bacterial pathogens in southwestern Finland. Parasit Vectors. 2016;9.
265. Skotarczak B, Wodecka B, Rymaszewska A, Adamska M. Molecular evidence for bacterial pathogens in Ixodes ricinus ticks infesting Shetland ponies. Experimental & applied acarology. 2016;69(2):179-89.

266. Siński E, Welc-Falęciak R, Zajkowska J. Borrelia miyamotoi: A human tick-borne relapsing fever spirochete in Europe and its potential impact on public health. Advances in Medical Sciences. 2016;61(2):255-60.
267. Shirani D, Rakhshanpoor A, Cutler SJ, Ghazinezhad B, Naddaf SR. A case of canine borreliosis in Iran caused by Borrelia persica. Ticks and Tick-borne Diseases. 2016;7(3):424-6.
268. Shayeghi M, Piazak N, Gollampuor A, Nasirian H, Abolhassani M. Tick-borne relapsing fever in Sabzevar (Khorasan Razavy Province), North-Eastern Iran. Bangladesh Journal of Medical Science. 2016;15(4):551-5.
269. Sathiamoorthi S, Smith WM. The eye and tick-borne disease in the United States. Current Opinion in Ophthalmology. 2016;27(6):530-7.
270. Sakakibara K, Sen E, Sato K, Kawabata H, Ohashi N, Masuzawa T. Detection and Characterization of the Emerging Relapsing Fever Pathogen, Borrelia miyamotoi, from the Ixodes ricinus Tick in the Rural Trakya (Thrace) Region of Northwestern Turkey. Vector-Borne and Zoonotic Diseases. 2016;16(12):797-9.
271. Raja H, Starr MR, Bakri SJ. Ocular manifestations of tick-borne diseases. Survey of Ophthalmology. 2016;61(6):726-44.
272. Potkonjak A, Kleinerman G, Gutiérrez R, Savić S, Vračar V, Nachum-Biala Y, et al. Occurrence of Borrelia burgdorferi Sensu Lato in Ixodes ricinus Ticks with First Identification of Borrelia miyamotoi in Vojvodina, Serbia. Vector-Borne and Zoonotic Diseases. 2016;16(10):631-5.
273. Piccione J, Levine GJ, Duff CA, Kuhlman GM, Scott KD, Esteve-Gassent MD. Tick-Borne Relapsing Fever in Dogs. Journal of veterinary internal medicine. 2016;30(4):1222-8.
274. Nelder MP, Russell CB, Sheehan NJ, Sander B, Moore S, Li Y, et al. Human pathogens associated with the blacklegged tick Ixodes scapularis: a systematic review. Parasit Vectors. 2016;9.
275. Nazari M, Najafi A. Epidemiological study of endemic relapsing fever in Hamadan province, west of Iran. Journal of Arthropod-Borne Diseases. 2016;10(4):586-94.
276. Muñoz-Leal S, Venzal JM, González-Acuña D, Nava S, Lopes MG, Martins TF, et al. A new species of Ornithodoros (Acari: Argasidae) from desert areas of northern Chile. Ticks and Tick-borne Diseases. 2016;7(5):901-10.
277. Moutailler S, Valiente Moro C, Vaumourin E, Michelet L, Tran FH, Devillers E, et al. Co-infection of Ticks: The Rule Rather Than the Exception. PLoS Negl Trop Dis. 2016;10(3).
278. Mafra C, Montandon CE. Borreliosis. Arthropod Borne Diseases2016. p. 193-204.
279. Lopez JE, Krishnavahjala A, Garcia MN, Bermudez S. Tick-Borne Relapsing Fever Spirochetes in the Americas. Vet Sci. 2016;3(3).
280. Krisztina S, Sándor H. Epidemiological significance of bats (Chiroptera) in Europe, with emphasis on their bloodsucking ectoparasites as potential transmitters of vector-borne pathogens. Magyar Allatorvosok Lapja. 2016;138(1):15-29.
281. Krause PJ, Schwab J, Narasimhan S, Brancato J, Xu G, Rich SM. Hard tick relapsing fever caused by Borrelia miyamotoi in a Child. Pediatric Infectious Disease Journal. 2016;35(12):1352-4.
282. Kotlyar S. Tick-Borne Relapsing Fever in Southwest Colorado: A Case Report. Journal of Emergency Medicine. 2016;52(1):83-5.
283. Kim HC, Kwon YS, Kim MR, Park CU, Yun S, Lee WJ, et al. Ticks collected from Chilbal Island, Jeollanam Province, Republic of Korea, during 2014-2015. Systematic and Applied Acarology. 2016;21(12):1641-50.

284. Khasnatinov MA, Danchinova GA, Takano A, Kawabata H, Ohashi N, Masuzawa T. Prevalence of Borrelia miyamotoi in Ixodes persulcatus in Irkutsk City and its neighboring territories, Russia. Ticks and Tick-Borne Diseases. 2016;7(2):394-7.
285. Kernif T, Leulmi H, Raoult D, Parola P. Emerging tick-borne bacterial pathogens. Microbiology Spectrum. 2016;4(3).
286. Kalmár Z, Sprong H, Mihalca AD, Gherman CM, Dumitrache MO, Coipan EC, et al. Borrelia miyamotoi and Candidatus Neoehrlichia mikurensis in Ixodes ricinus Ticks, Romania. Emerg Infect Dis. 2016;22(3):550-1.
287. Jones JM, Hranac CR, Schumacher M, Horn K, Lee DM, Terriquez J, et al. Tick-borne relapsing fever outbreak among a high school football team at an outdoor education camping trip, Arizona, 2014. American Journal of Tropical Medicine and Hygiene. 2016;95(3):546-50.
288. Johnson TL, Landguth EL, Stone EF. Modeling Relapsing Disease Dynamics in a Host-Vector Community. PLoS Neglected Tropical Diseases. 2016;10(2).
289. Johnson TL, Fischer RJ, Raffel SJ, Schwan TG. Host associations and genomic diversity of Borrelia hermsii in an endemic focus of tick-borne relapsing fever in western North America. Parasites and Vectors. 2016;9(1):1-17.
290. Jobe DA, Lovrich SD, Oldenburg DG, Kowalski TJ, Callister SM. Borrelia miyamotoi Infection in Patients from Upper Midwestern United States, 2014-2015. Emerging Infectious Diseases. 2016;22(8):1471-3.
291. Jahfari S, Hofhuis A, Fonville M, van der Giessen J, van Pelt W, Sprong H. Molecular Detection of Tick-Borne Pathogens in Humans with Tick Bites and Erythema Migrans, in the Netherlands. PLoS Negl Trop Dis. 2016;10(10).
292. Jaenson TGT, Värv K, Fröjdman I, Jääskeläinen A, Rundgren K, Versteirt V, et al. First evidence of established populations of the taiga tick Ixodes persulcatus (Acari: Ixodidae) in Sweden. Parasit Vectors. 2016;9.
293. Inci A, Yildirim A, Duzlu O, Doganay M, Aksoy S. Tick-Borne Diseases in Turkey: A Review Based on One Health Perspective. PLoS Neglected Tropical Diseases. 2016;10(12).
294. Han S, Hickling GJ, Tsao JI. High Prevalence of Borrelia miyamotoi among Adult Blacklegged Ticks from White-Tailed Deer. Emerg Infect Dis. 2016;22(2):316-8.
295. Graves SR, Jackson C, Hussain-Yusuf H, Vincent G, Nguyen C, Stenos J, et al. Ixodes holocyclus Tick-Transmitted Human Pathogens in North-Eastern New South Wales, Australia. Trop Med Infect Dis. 2016;1(1).
296. Gleim ER, Garrison LE, Vello MS, Savage MY, Lopez G, Berghaus RD, et al. Factors associated with tick bites and pathogen prevalence in ticks parasitizing humans in Georgia, USA. Parasit Vectors. 2016;9.
297. Gaither M, Schumacher M, Nieto N, Corrigan J, Murray H, Maurer M. Where Are the Ticks? Solving the Mystery of a Tickborne Relapsing Fever Outbreak at a Youth Camp. Journal of environmental health. 2016;78(8):8-11.
298. Fiorito T, Godding M, Reece R, Flanigan T, Silverblatt F. Utility of borrelia miyamotoi polymerase chain reaction in Rhode island: A case series. Open Forum Infectious Diseases. 2016;3.
299. Fingerle V, Pritsch M, Wächtler M, Margos G, Ruske S, Jung J, et al. "Candidatus Borrelia kalaharica" Detected from a Febrile Traveller Returning to Germany from Vacation in Southern Africa. PLoS Neglected Tropical Diseases. 2016;10(3).
300. Faccini-Martinez AA, Botero-Garcia CA. Regarding Tick-Borne Relapsing Fever in the Americas; Some Historical Aspects of a Forgotten Disease in Colombia. Veterinary sciences. 2016;3(4).

301. Ehounoud CB, Yao KP, Dahmani M, Achi YL, Amanzougaghene N, Kacou N'Douba A, et al. Multiple Pathogens Including Potential New Species in Tick Vectors in Côte d'Ivoire. PLoS Neglected Tropical Diseases. 2016;10(1).
302. Donaldson TG, de Leon AAP, Li AI, Castro-Arellano I, Wozniak E, Boyle WK, et al. Assessment of the Geographic Distribution of Ornithodoros turicata (Argasidae): Climate Variation and Host Diversity. PloS Neglected Tropical Diseases. 2016;10(2):e0004383.
303. Diatta G, Mediannikov O, Boyer S, Sokhna C, Bassène H, Fenollar F, et al. An alternative strategy of preventive control of tick-borne relapsing fever in rural areas of Sine-Saloum, Senegal. American Journal of Tropical Medicine and Hygiene. 2016;95(3):537-45.
304. Cutler SJ, Ruzic-Sabljic E, Potkonjak A. Emerging borreliae – Expanding beyond Lyme borreliosis. Molecular and Cellular Probes. 2016;31:22-7.
305. Cook VJ, Fedorova N, Macdonald WP, Lane RS, Barbour AG. Unique Strain of Borrelia miyamotoi in Ixodes pacificus Ticks, California, USA. Emerg Infect Dis. 2016;22(12):2205-7.
306. Clow KM, Ogden NH, Lindsay LR, Michel P, Pearl DL, Jardine CM. Distribution of Ticks and the Risk of Lyme Disease and Other Tick-Borne Pathogens of Public Health Significance in Ontario, Canada. Vector-Borne and Zoonotic Diseases. 2016;16(4):215-22.
307. Choi E, Pyzocha NJ, Maurer DM. Tick-Borne Illnesses. Current Sports Medicine Reports. 2016;15(2):98-104.
308. Castilla-Guerra L, Marín-Martín J, Colmenero-Camacho MA. Tick-borne relapsing fever, Southern Spain, 2004–2015. Emerging Infectious Diseases. 2016;22(12):2217-9.
309. Castilla-Guerra L, Fernandez-Moreno MC, Vergara-Lopez S, Merino-Rumin M, Colmenero-Camacho MA. Neurological complications of tick-borne relapsing fever. Revista de neurologia. 2016;63(6):252-6.
310. Castilla-Guerra L, Fernandez-Moreno MC, Alvarez-Suero J, Marin-Martin J. Recurrent fever transmitted by ticks: an infrequent cause of meningitis. Revista De Neurologia. 2016;62(3):141-2.
311. Cable RG, Trouern-Trend J. Tickborne infections. Blood Safety and Surveillance2016. p. 399-422.
312. Aubry C, Socolovschi C, Raoult D, Parola P. Bacterial agents in 248 ticks removed from people from 2002 to 2013. Ticks and Tick-borne Diseases. 2016;7(3):475-81.
313. Yu Z, Wang H, Wang T, Sun W, Yang X, Liu J. Tick-borne pathogens and the vector potential of ticks in China. Parasit Vectors. 2015;8.
314. Wormser GP, Pritt B. Update and Commentary on Four Emerging Tick-Borne Infections: Ehrlichia muris-like Agent, Borrelia miyamotoi, Deer Tick Virus, Heartland Virus, and Whether Ticks Play a Role in Transmission of Bartonella henselae. Infectious Disease Clinics of North America. 2015;29(2):371-81.
315. Wilder HK, Wozniak E, Huddleston E, Tata R, Fitzkee NC, Lopez JE. Case Report: A Retrospective Serological Analysis Indicating Human Exposure to Tick-Borne Relapsing Fever Spirochetes in Texas. PloS Neglected Tropical Diseases. 2015;9(4):e0003617.
316. Wagemakers A, Staarink PJ, Sprong H, Hovius JW. Borrelia miyamotoi: a widespread tick-borne relapsing fever spirochete. Trends Parasitol. 2015;31(6):260-9.
317. Tyner HL, Sloan LM, Paskewitz SM, Hoang Johnson DK, Lee X, Pritt BS. Use for a novel real-time fret PCR assay for detection for tick-borne relapsing fever group borrelia from ixodes scapularis ticks collected in Wisconsin (2013-2014). American Journal of Tropical Medicine and Hygiene. 2015;93(4):215.
318. Telford SR, Goethert HK, Molloy PJ, Berardi VP, Chowdri HR, Gugliotta JL, et al. Borrelia miyamotoi Disease: Neither Lyme Disease Nor Relapsing Fever. Clinics in Laboratory Medicine. 2015;35(4):867-82.

319. Szekeres S, Coipan EC, Rigó K, Majoros G, Jahfari S, Sprong H, et al. Eco-epidemiology of Borrelia miyamotoi and Lyme borreliosis spirochetes in a popular hunting and recreational forest area in Hungary. Parasites and Vectors. 2015;8(1).
320. Skar G, Snowden J. Recurrent fever and thrombocytopenia in a 4-year-old girl. Pediatrics in Review. 2015;36(3):130-1.
321. Sarksyan DS, Platonov AE, Karan LS, Shipulin GA, Sprong H, Hovlus JWR. Probability of Spirochete Borrelia miyamotoi Transmission from Ticks to Humans. Emerging Infectious Diseases. 2015;21(12):2273-4.
322. Sarksyan DS, Maleev VV, Platonov AE, Platonova OV, Karan LS. Relapsing (recurrent) disease caused by Borrelia miyamotoi. Terapevticheskiĭ arkhiv. 2015;87(11):18-25.
323. Salkeld DJ, Nieto NC, Carbajales-Dale P, Carbajales-Dale M, Cinkovich SS, Lambin EF. Disease Risk & Landscape Attributes of Tick-Borne Borrelia Pathogens in the San Francisco Bay Area, California. PLoS One. 2015;10(8).
324. Reiter M, Schoetta A-M, Mueller A, Stockinger H, Stanek G. A newly established real-time PCR for detection of Borrelia miyamotoi in Ixodes ricinus ticks. Ticks and Tick-Borne Diseases. 2015;6(3):303-8.
325. Quarsten H, Skarpaas T, Fajs L, Noraas S, Kjelland V. Tick-borne bacteria in Ixodes ricinus collected in southern Norway evaluated by a commercial kit and established real-time PCR protocols. Ticks and Tick-borne Diseases. 2015;6(4):538-44.
326. Oscar Betancur H, Antonio Betancourt E, Cristian Giraldo R. Importance of ticks in the transmission of zoonotic agents. Revista MVZ Cordoba. 2015;20:5053-67.
327. Nunes M, Parreira R, Lopes N, Maia C, Carreira T, Sousa C, et al. Molecular Identification of Borrelia miyamotoi in Ixodes ricinus from Portugal. Vector-Borne and Zoonotic Diseases. 2015;15(8):515-7.
328. Naddaf SR, Ghazinezhad B, Sedaghat MM, Asl HM, Cutler SJ. Tickborne relapsing fever in Southern Iran, 2011–2013. Emerging Infectious Diseases. 2015;21(6):1078-80.
329. Molloy PJ, Telford SR, Chowdri HR, Lepore TJ, Gugliotta JL, Weeks KE, et al. Borrelia miyamotoi Disease in the Northeastern United States A Case Series. Annals of Internal Medicine. 2015;163(2):91-+.
330. Melkert P, Melkert D, Kahema L, Van Der Velden K, Van Roosmalen J. Estimation of changes in maternal mortality in a rural district of northern Tanzania during the last 50 years. Acta Obstetricia et Gynecologica Scandinavica. 2015;94(4):419-24.
331. Kumsa B, Socolovschi C, Raoult D, Parola P. New Borrelia species detected in ixodid ticks in Oromia, Ethiopia. Ticks and Tick-borne Diseases. 2015;6(3):401-7.
332. Krawczyk AI, Van Leeuwen AD, Jacobs-Reitsma W, Wijnands LM, Bouw E, Jahfari S, et al. Presence of zoonotic agents in engorged ticks and hedgehog faeces from Erinaceus europaeus in (sub) urban areas. Parasites and Vectors. 2015;8(1).
333. Krause PJ, Fish D, Narasimhan S, Barbour AG. Borrelia miyamotoi infection in nature and in humans. Clinical Microbiology and Infection. 2015;21(7):631-9.
334. Krause PJ, Barbour AG. Borrelia miyamotoi: The Newest Infection Brought to Us by Deer Ticks. Annals of Internal Medicine. 2015;163(2):141-+.
335. Kjelland V, Rollum R, Korslund L, Slettan A, Tveitnes D. Borrelia miyamotoi is widespread in Ixodes ricinus ticks in southern Norway. Ticks and Tick-borne Diseases. 2015;6(4):516-21.
336. Jones JM, Schumacher M, Peoples M, Souders N, Horn K, Fox L, et al. Tickborne Relapsing Fever Outbreak at an Outdoor Education Camp — Arizona, 2014. MMWR Morb Mortal Wkly Rep. 2015;64(23):651-2.

337. Jasik KP, Okla H, Slodki J, Rozwadowska B, Slodki A, Rupik W. Congenital Tick Borne Diseases: Is This An Alternative Route of Transmission of Tick-Borne Pathogens In Mammals? Vector-Borne and Zoonotic Diseases. 2015;15(11):637-44.
338. Hu LT, Tsibris AM, Branda JA. Case 24-2015: A 28-Year-Old Pregnant Woman with Fever, Chills, Headache, and Fatigue. New England Journal of Medicine. 2015;373(5):468-75.
339. Hansford KM, Fonville M, Jahfari S, Sprong H, Medlock JM. Borrelia miyamotoi in host-seeking Ixodes ricinus ticks in England. Epidemiology and Infection. 2015;143(5):1079-87.
340. Gabriele-Rivet V, Arsenault J, Badcock J, Cheng A, Edsall J, Goltz J, et al. Different ecological niches for ticks of public health significance in Canada. PLoS ONE. 2015;10(7).
341. Fuchs I, Tarabin S, Kafka M. Relapsing Fever: Diagnosis Thanks to a Vigilant Hematology Laboratory. Vector-Borne and Zoonotic Diseases. 2015;15(7):446-8.
342. Fotso Fotso A, Angelakis E, Mouffok N, Drancourt M, Raoult D. Blood-Borne Candidatus Borrelia algerica in a Patient with Prolonged Fever in Oran, Algeria. Am J Trop Med Hyg. 2015;93(5):1070-3.
343. Fotso AF, Drancourt M. Laboratory Diagnosis of Tick-Borne African Relapsing Fevers: Latest Developments. Frontiers in Public Health. 2015;3.
344. Forrester JD, Kjemtrup AM, Fritz CL, Marsden-Haug N, Nichols JB, Tengelsen LA, et al. Tickborne Relapsing Fever — United States, 1990–2011. MMWR Morb Mortal Wkly Rep. 2015;64(3):58-60.
345. Elbir H, Fotsofotso A, Diatta G, Trape JF, Arnathau C, Renaud F, et al. Ubiquitous bacteria Borrelia crocidurae in Western African ticks Ornithodoros sonrai. Parasites and Vectors. 2015;8(1).
346. Eisen RJ, Eisen L, Beard CB. County-Scale Distribution of Ixodes scapularis and Ixodes pacificus (Acari: Ixodidae) in the Continental United States. J Med Entomol. 2015;53(2):349-86.
347. Diatta G, Duplantier J-M, Granjon L, Ba K, Chauvancy G, Ndiaye M, et al. Borrelia infection in small mammals in West Africa and its relationship with tick occurrence inside burrows. Acta Tropica. 2015;152:131-40.
348. Cutler SJ. Relapsing Fever Borreliae: A Global Review. Clinics in Laboratory Medicine. 2015;35(4):847-65.
349. Cochez C, Heyman P, Heylen D, Fonville M, Hengeveld P, Takken W, et al. The Presence of Borrelia miyamotoi, A Relapsing Fever Spirochaete, in Questing Ixodes ricinus in Belgium and in The Netherlands. Zoonoses and Public Health. 2015;62(5):331-3.
350. Christensen J, Fischer RJ, McCoy BN, Raffel SJ, Schwan TG. Tickborne relapsing fever, bitterroot valley, Montana, USA. Emerging Infectious Diseases. 2015;21(2):217-23.
351. Chikeka I, Dumler JS. Neglected bacterial zoonoses. Clinical Microbiology and Infection. 2015;21(5):404-15.
352. Cerdan M, Martínez IS, Cabanes BP, Guarnizo EC, Fernandez PA, Nieto RE, et al. Borrelia hispanica: An emerging infectious agent causing neuroborreliosis. Neurology. 2015;84.
353. Cerar T, Korva M, Avšič-Županc T, Ružić-Sabljić E. Detection, identification and genotyping of Borrellia spp. in rodents in Slovenia by PCR and culture. BMC veterinary research. 2015;11:188.
354. Burazerović J, Ćakić S, Mihaljica D, Sukara R, Ćirović D, Tomanović S. Ticks (Acari: Argasidae, Ixodidae) parasitizing bats in the central Balkans. Experimental and Applied Acarology. 2015;66(2):281-91.

355. Ayazi P, Mahyar A, Oveisi S, Esmailzadehha N, Nooroozi S. Tick-borne Relapsing Fever in Children in the North-west of Iran, Qazvin. Prague medical report. 2015;116(3):193-202.
356. Aslam B, Hussain I, Zahoor MA, Mahmood MS, Rasool MH. Prevalence of Borrelia anserina in Argas ticks. Pakistan Journal of Zoology. 2015;47(4):1125-31.
357. Wang G. Borrelia burgdorferi and Other Borrelia Species. Molecular Medical Microbiology: Second Edition. 32014. p. 1867-909.
358. Takano A, Toyomane K, Konnai S, Ohashi K, Nakao M, Ito T, et al. Tick surveillance for relapsing fever spirochete Borrelia miyamotoi in Hokkaido, Japan. PLoS ONE. 2014;9(8).
359. Souidi Y, Boudebouch N, Ezikouri S, Belghyti D, Jean-François T, Sarih M. Borrelia crocidurae in Ornithodoros ticks from northwestern Morocco: A range extension in relation to climatic change? Journal of Vector Ecology. 2014;39(2):316-20.
360. Schreiber C, Krücken J, Beck S, Maaz D, Pachnicke S, Krieger K, et al. Pathogens in ticks collected from dogs in Berlin/Brandenburg, Germany. Parasit Vectors. 2014;7.
361. Sato K, Takano A, Konnai S, Nakao M, Ito T, Koyama K, et al. Human infections with Borrelia miyamotoi, Japan. Emerging Infectious Diseases. 2014;20(8):1391-3.
362. Sándor AD, Mărcuţan DI, D'Amico G, Gherman CM, Dumitrache MO, Mihalca AD. Do the Ticks of Birds at an Important Migratory Hotspot Reflect the Seasonal Dynamics of Ixodes ricinus at the Migration Initiation Site? A Case Study in the Danube Delta. PLoS One. 2014;9(2).
363. Salkeld DJ, Cinkovich S, Nieto NC. Tick-borne Pathogens in Northwestern California, USA. Emerg Infect Dis. 2014;20(3):493-4.
364. Russart NM, Dougherty MW, Vaughan JA. Survey of ticks (Acari: Ixodidae) and tick-borne pathogens in North Dakota. Journal of Medical Entomology. 2014;51(5):1087-90.
365. Padgett K, Bonilla D, Kjemtrup A, Vilcins I-M, Yoshimizu MH, Hui L, et al. Large Scale Spatial Risk and Comparative Prevalence of Borrelia miyamotoi and Borrelia burgdorferi Sensu Lato in Ixodes pacificus. PLoS One. 2014;9(10):e110853.
366. Olmo Montes FJ, Sojo Dorado J, Peñas Espinar C, Muniáin Ezcurra MA. Borrelia species: Lyme disease and relapsing fever. Medicine (Spain). 2014;11(51):3009-17.
367. Nieto NC, Teglas MB. Relapsing fever group borrelia in southern California rodents. Journal of Medical Entomology. 2014;51(5):1029-34.
368. Mirzaei M, Khedri J. Ixodidae ticks in cattle and sheep in sistan and baluchestan province (Iran). Veterinaria Italiana. 2014;50(1):65-8.
369. Michelet L, Delannoy S, Devillers E, Umhang G, Aspan A, Juremalm M, et al. High-throughput screening of tick-borne pathogens in Europe. Front Cell Infect Microbiol. 2014;4.
370. Mediannikov O, Socolovschi C, Bassene H, Diatta G, Ratmanov P, Fenollar F, et al. High incidence of Borrelia crocidurae in acute febrile patients in Senegal. International Journal of Infectious Diseases. 2014;21:218.
371. McCoy BN, Maïga O, Schwan TG. Detection of Borrelia theileri in Rhipicephalus geigyi from Mali. Ticks Tick Borne Dis. 2014;5(4):401-3.
372. Lommano E, Dvořák C, Vallotton L, Jenni L, Gern L. Tick-borne pathogens in ticks collected from breeding and migratory birds in Switzerland. Ticks and Tick-borne Diseases. 2014;5(6):871-82.
373. Lee K, Takano A, Taylor K, Sashika M, Shimozuru M, Konnai S, et al. A relapsing fever group Borrelia sp. similar to Borrelia lonestari found among wild sika deer (Cervus nippon yesoensis) and Haemaphysalis spp. ticks in Hokkaido, Japan. Ticks and Tick-borne Diseases. 2014;5(6):841-7.

374. Lee JK, Smith WC, McIntosh C, Ferrari FG, Moore-Henderson B, Varela-Stokes A. Detection of a Borrelia species in questing Gulf Coast ticks, Amblyomma maculatum. Ticks and Tick-borne Diseases. 2014;5(4):449-52.
375. Krause PJ, Narasimhan S, Wormser GP, Barbour AG, Platonov AE, Brancato J, et al. Borrelia miyamotoi sensu lato seroreactivity and seroprevalence in the northeastern United States. Emerging Infectious Diseases. 2014;20(7):1183-90.
376. Kiewra D, Stańczak J, Richter M. Ixodes ricinus ticks (Acari, Ixodidae) as a vector of Borrelia burgdorferi sensu lato and Borrelia miyamotoi in Lower Silesia, Poland - Preliminary study. Ticks and Tick-borne Diseases. 2014;5(6):892-7.
377. Kelly AL, Raffel SJ, Fischer R, Bellinghausen M, Stevenson C, Schwan TG. First isolation of the relapsing fever spirochete, Borrelia herrnsii, from a domestic dog. Ticks and Tick-Borne Diseases. 2014;5(2):95-9.
378. Kassiri H, Kasiri A, Karimi M, Kasiri E, Lotfi M. The seven-year longitudinal study on relapsing fever borreliosis in Western Iran. Asian Pacific Journal of Tropical Disease. 2014;4(S2):S679-S83.
379. Kassiri H, Kasiri A, Dostifar K, Lotfi M. The epidemiology of tick-borne relapsing fever in Bijar County, North-Western Iran. Journal of Acute Disease. 2014;3(3):224-7.
380. Hussein H, Showler A, Tan DHS. Tick-borne relapsing fever in pregnancy. CMAJ. 2014;186(2):131-4.
381. Horton JM. Relapsing Fever Caused by Borrelia Species. Mandell, Douglas, and Bennett's Principles and Practice of Infectious Diseases. 22014. p. 2721-4.
382. Halperin JJ, García-Moncó JC. The human borreliosis: Lyme neuroborreliosis and relapsing fever. CNS Infections: A Clinical Approach2014. p. 211-26.
383. Fonville M, Friesema I, Hengeveld PD, Docters van Leeuwen A, Jahfari S, Harms MG, et al. Human Exposure to Tickborne Relapsing Fever Spirochete Borreliamiyamotoi, the Netherlands. Emerg Infect Dis. 2014;20(7):1244-6.
384. Felder H, Hoekstra KA. Borrelia hermsii relapsing fever. Blood. 2014;123(2):160.
385. Fedorova N, Kleinjan JE, James D, Hui LT, Peeters H, Lane RS. Remarkable diversity of tick or mammalian-associated Borreliae in the metropolitan San Francisco Bay Area, California. Ticks and Tick-borne Diseases. 2014;5(6):951-61.
386. Faulde M, Freise J. Public health pests: Arthropods and rodents as causative disease agents as well as reservoirs and vectors of pathogens. Bundesgesundheitsblatt - Gesundheitsforschung - Gesundheitsschutz. 2014;57(5):495-503.
387. Eshoo MW, Crowder CD, Carolan HE, Rounds MA, Ecker DJ, Haag H, et al. Broad-Range Survey of Tick-Borne Pathogens in Southern Germany Reveals a High Prevalence of Babesia microti and a Diversity of Other Tick-Borne Pathogens. Vector Borne Zoonotic Dis. 2014;14(8):584-91.
388. Dobler G, Fingerle V, Hagedorn P, Pfeffer M, Silaghi C, Tomaso H, et al. Threat of transmission of infectious pathogens by Ixodes ricinus ticks in Germany. Bundesgesundheitsblatt - Gesundheitsforschung - Gesundheitsschutz. 2014;57(5):541-8.
389. Dibernardo A, Cote T, Ogden NH, Lindsay LR. The prevalence of Borrelia miyamotoi infection, and co-infections with other Borrelia spp. in Ixodes scapularis ticks collected in Canada. Parasit Vectors. 2014;7:183.
390. Crowder CD, Carolan HE, Rounds MA, Honig V, Mothes B, Haag H, et al. Prevalence of Borrelia miyamotoi in Ixodes ticks in Europe and the United States. Emerging Infectious Diseases. 2014;20(10):1678-82.

391. Croche Santander B, Sánchez Carrión A, Campos E, Toro C, Marcos L, Vargas JC, et al. Tick-borne relapsing fever in a rural area of southern Spain. Anales de Pediatria. 2014;82(1):e73-e7.
392. Cosson J-F, Michelet L, Chotte J, Le Naour E, Cote M, Devillers E, et al. Genetic characterization of the human relapsing fever spirochete Borrelia miyamotoi in vectors and animal reservoirs of Lyme disease spirochetes in France. Parasites & vectors. 2014;7:233.
393. Bonilla D, Kjemtrup A, Vilcins I-M, Hui L, Sola M, Quintana M, et al. Proportion of individual adult and nymphal Ixodes pacificus ticks with Borrelia burgdorferi sensu lato and B. miyamotoi detected in California, 2009-2012. Figshare; 2014.
394. Boinas F, Ribeiro R, Madeira S, Palma M, de Carvalho IL, Núncio S, et al. The medical and veterinary role of Ornithodoros erraticus complex ticks (Acari: Ixodida) on the Iberian Peninsula. Journal of vector ecology : journal of the Society for Vector Ecology. 2014;39(2):238-48.
395. Veena S, Seema V, Babu R. Borreliosis: Recurrent fever due to spirochetes. Annals of Tropical Medicine and Public Health. 2013;6(4):482-4.
396. Vayssier-Taussat M, Moutailler S, Michelet L, Devillers E, Bonnet S, Cheval J, et al. Next Generation Sequencing Uncovers Unexpected Bacterial Pathogens in Ticks in Western Europe. PLoS One. 2013;8(11).
397. Trape JF, Diatta G, Arnathau C, Bitam I, Sarih M, Belghyti D, et al. The epidemiology and geographic distribution of relapsing fever borreliosis in West and North Africa, with a review of the Ornithodoros erraticus complex (Acari: Ixodida). PLoS ONE. 2013;8(11).
398. Sokhna C, Mediannikov O, Fenollar F, Bassene H, Diatta G, Tall A, et al. Point-of-Care Laboratory of Pathogen Diagnosis in Rural Senegal. PLoS Negl Trop Dis. 2013;7(1).
399. Simon RB. Hidden dangers: Non-Lyme tick-borne diseases. Nursing. 2013;43(9):48-54.
400. Shah RG, Sood SK. Clinical approach to known and emerging tick-borne infections other than Lyme disease. Current Opinion in Pediatrics. 2013;25(3):407-18.
401. Seraji-Bozorgzad N, Tselis AC. Non-Lyme tick-borne diseases: A neurological perspective topical collection on infection. Current Neurology and Neuroscience Reports. 2013;13(10).
402. Rustenhoven-Spaan I, Melkert P, Nelissen E, van Roosmalen J, Stekelenburg J. Maternal mortality in a rural tanzanian hospital: Fatal Jarisch-Herxheimer reaction in a case of relapsing fever in pregnancy. Tropical Doctor. 2013;43(4):138-41.
403. Pujalte GGA, Chua JV. Tick-borne infections in the United States. Primary Care - Clinics in Office Practice. 2013;40(3):619-35.
404. Piksa K, Górz A, Nowak-Chmura M, Siuda K. Mass occurrence of Ixodes vespertilionis (Acari: Ixodidae) in caves, on bats roosting in caves and in a nursery colony. International Journal of Acarology. 2013;39(3):257-62.
405. Palma M, de Carvalho IL, Osorio H, Ze-Ze L, Cutler SJ, Nuncio MS. Portuguese Hosts for Ornithodoros erraticus Ticks. Vector-Borne and Zoonotic Diseases. 2013;13(10):775-7.
406. Ncbi. Borrelia hermsii overview. European Nucleotide Archive; 2013.
407. Ncbi. Borrelia turicatae overview. European Nucleotide Archive; 2013.
408. Ncbi. Borrelia duttonii overview. European Nucleotide Archive; 2013.
409. Moran-Gilad J, Levine H, Schwartz E, Bartal C, Huerta-Hartal M, Schwaber MJ, et al. Postexposure prophylaxis of tick-borne relapsing fever: Lessons learned from recent outbreaks in Israel. Vector-Borne and Zoonotic Diseases. 2013;13(11):791-7.
410. Melkert P, Kahema L, van der Velden J, van Roosmalen J. Relapsing fever, a disappearing cause of fever and maternal death in Sengerema, East Africa. East African medical journal. 2013;90(4):137-41.

411. Mediannikov O, Abdissa A, Socolovschi C, Diatta G, Trape J-F, Raoult D. Detection of a New Borrelia Species in Ticks Taken from Cattle in Southwest Ethiopia. Vector-Borne and Zoonotic Diseases. 2013;13(4):266-9.
412. Leydet BF, Liang FT. Detection of human bacterial pathogens in ticks collected from Louisiana black bears (Ursus americanus luteolus). Ticks Tick Borne Dis. 2013;4(3).
413. Lane RS, Fedorova N, Kleinjan JE, Maxwell M. Eco-epidemiological factors contributing to the low risk of human exposure to ixodid tick-borne borreliae in southern California, USA. Ticks and Tick-borne Diseases. 2013;4(5):377-85.
414. Kutsuna S, Kawabata H, Kasahara K, Takano A, Mikasa K. Case report: The first case of imported relapsing fever in Japan. American Journal of Tropical Medicine and Hygiene. 2013;89(3):460-1.
415. Krause PJ, Narasimhan S, Wormser GP, Rollend L, Fikrig E, Lepore T, et al. Human Borrelia miyamotoi Infection in the United States. N Engl J Med. 2013;368(3):291-3.
416. Hovius JWR, De Wever B, Sohne M, Brouwer MC, Coumou J, Wagemakers A, et al. A case of meningoencephalitis by the relapsing fever spirochaete Borrelia miyamotoi in Europe. The Lancet. 2013;382(9892):658.
417. Gholkar N, Lehman D. Borrelia hermsii (relapsing fever). New England Journal of Medicine. 2013;368(3):266.
418. Fritz CL, Payne JR, Schwan TG. Serologic evidence for Borrelia hermsii Infection in rodents on federally owned recreational areas in California. Vector-Borne and Zoonotic Diseases. 2013;13(6):376-81.
419. Elbir H, Raoult D, Drancourt M. Review article: Relapsing fever borreliae in Africa. American Journal of Tropical Medicine and Hygiene. 2013;89(2):288-92.
420. Elbir H, Henry M, Diatta G, Mediannikov O, Sokhna C, Tall A, et al. Multiplex Real-Time PCR Diagnostic of Relapsing Fevers in Africa. PLoS Neglected Tropical Diseases. 2013;7(1).
421. Dotters-Katz SK, Kuller J, Heine RP. Arthropod-borne bacterial diseases in pregnancy. Obstetrical and Gynecological Survey. 2013;68(9):635-49.
422. Chong ST, Kim HC, Lee IY, Kollars TM, Sancho AR, Sames WJ, et al. Seasonal Distribution of Ticks in Four Habitats near the Demilitarized Zone, Gyeonggi-do (Province), Republic of Korea. Korean J Parasitol. 2013;51(3):319-25.
423. Chong ST, Kim HC, Lee IY, Kollars Jr TM, Sancho AR, Sames WJ, et al. Comparison of dragging and sweeping methods for collecting ticks and determining their seasonal distributions for various habitats, gyeonggi province, Republic of Korea. Journal of Medical Entomology. 2013;50(3):611-8.
424. Branda JA, Rosenberg ES. Borrelia miyamotoi: A lesson in disease discovery. Annals of Internal Medicine. 2013;159(1):61-2.
425. Bowles DE, Robbins RG, Harlan HJ, Carpenter TL. New Missouri County records and review of the distribution and disease vector potential of ornithodoros kelleyi (Arachnida: Ixodida: Argasidae) and cimex adjunctus (Insecta: Hemiptera: Cimicidae). Proceedings of the Entomological Society of Washington. 2013;115(2):117-27.
426. Ytrehus B, Vikøren T. Borrelia Infections. Infectious Diseases of Wild Mammals and Birds in Europe2012. p. 345-62.
427. Yossepowitch O, Gottesman T, Schwartz-Harari O, Soroksky A, Dan M. Aseptic meningitis and adult respiratory distress syndrome caused by Borrelia persica. Infection. 2012;40(6):695-7.

428. Yakhchali M, Bahramnejad K, Almasi O. Ticks (Acari: Ixodida: Ixodidae and Argasidae) abundance and associated risk factors for animals in the natural habitat of Sanandaj suburb, Iran. International Journal of Acarology. 2012;38(4):353-61.
429. Yabsley MJ, Parsons NJ, Horne EC, Shock BC, Purdee M. Novel relapsing fever Borrelia detected in African penguins (Spheniscus demersus) admitted to two rehabilitation centers in South Africa. Parasitology Research. 2012;110(3):1125-30.
430. Venzal JM, Nava S, Mangold AJ, Mastropaolo M, Casás G, Guglielmone AA. Ornithodoros quilinensis sp. nov. (Acari, Argasidae), a new tick species from the Chacoan region in Argentina. Acta Parasitologica. 2012;57(3):329-36.
431. Takano A, Sugimori C, Fujita H, Kadosaka T, Taylor KR, Tsubota T, et al. A novel relapsing fever Borrelia sp. infects the salivary glands of the molted hard tick, Amblyomma geoemydae. Ticks and Tick-borne Diseases. 2012;3(4):259-61.
432. Subramanian G, Sekeyova Z, Raoult D, Mediannikov O. Multiple tick-associated bacteria in Ixodes ricinus from Slovakia. Ticks and Tick-borne Diseases. 2012;3(5-6):406-10.
433. Sotelo Cruz N, Valencia Mayoral P. Borreliosis, fiebre recurrente causada por espiroquetas. Informe de un caso. Boletín médico del Hospital Infantil de México. 2012;69(2):121-5.
434. Shemshad K, Rafinejad J, Kamali K, Piazak N, Sedaghat MM, Shemshad M, et al. Species diversity and geographic distribution of hard ticks (Acari: Ixodoidea: Ixodidae) infesting domestic ruminants, in Qazvin Province, Iran. Parasitology Research. 2012;110(1):373-80.
435. Shehab KW, Banaei N. Unexplained fever after a camping trip in the american Southwest. Journal of the Pediatric Infectious Diseases Society. 2012;1(3):254-5.
436. Schwan TG, Anderson JM, Lopez JE, Fischer RJ, Raffel SJ, McCoy BN, et al. Endemic Foci of the Tick-Borne Relapsing Fever Spirochete Borrelia crocidurae in Mali, West Africa, and the Potential for Human Infection. PLoS Neglected Tropical Diseases. 2012;6(11).
437. Rafinejad J, Shemshad K, Banafshi O. EPIDEMIOLOGICAL STUDY ON TICK-BORNE (ACARI: ARGASIDAE) RELAPSING FEVER IN KURDISTAN PROVINCE, IRAN, 2000-2004. Florida Entomologist. 2012;95(3):758-63.
438. Qablan MA, Kubelová M, Široký P, Modrý D, Amr ZS. Stray dogs of northern Jordan as reservoirs of ticks and tick-borne hemopathogens. Parasitology Research. 2012;111(1):301-7.
439. Palma M, de Carvalho IL, Figueiredo M, Amaro F, Boinas F, Cutler SJ, et al. Borrelia hispanica in Ornithodoros erraticus, Portugal. Clinical Microbiology and Infection. 2012;18(7):696-701.
440. Nieto NC, Teglas MB, Stewart KM, Wasley T, Wolff PL. Detection of relapsing fever spirochetes (Borrelia hermsii and Borrelia coriaceae) in free-ranging mule deer (Odocoileus hemionus) from Nevada, United States. Vector-Borne and Zoonotic Diseases. 2012;12(2):99-105.
441. Nieto N, Teglas M. Maintenance of endemic tick-borne relapsing fever (Borrelia hermsii) from the western United States. International Journal of Infectious Diseases. 2012;16:e140.
442. Naddaf SR, Ghazinezhad B, Bahramali G, Cutler SJ. Phylogenetic analysis of the spirochete Borrelia microti, a potential agent of relapsing fever in Iran. Journal of Clinical Microbiology. 2012;50(9):2873-6.
443. Lommano E, Bertaiola L, Dupasquier C, Gern L. Infections and Coinfections of Questing Ixodes ricinus Ticks by Emerging Zoonotic Pathogens in Western Switzerland. Appl Environ Microbiol. 2012;78(13):4606-12.

444. Liu S, Yuan C, Cui YF, Li BX, Wu LJ, Liu Y. Investigation of Borrelia spp. in ticks (Acari: Ixodidae) at the border crossings between China and Russia in Heilongjiang Province, China. Asian Pacific Journal of Tropical Medicine. 2012;5(6):459-64.
445. Krause PJ. Human Infections Caused by Borrelia miyamotoi. Abstracts of the Interscience Conference on Antimicrobial Agents and Chemotherapy. 2012;52:448.
446. Hamer SA, Hickling GJ, Keith R, Sidge JL, Walker ED, Tsao JI. Associations of passerine birds, rabbits, and ticks with Borrelia miyamotoi and Borrelia andersonii in Michigan, U.S.A. Parasites and Vectors. 2012;5(1).
447. Gétaz L, Loutan L, Mezger N. Diseases transmitted by ticks locally and abroad. Revue Medicale Suisse. 2012;8(340):974-6.
448. Geller J, Nazarova L, Katargina O, Järvekülg L, Fomenko N, Golovljova I. Detection and Genetic Characterization of Relapsing Fever Spirochete Borrelia miyamotoi in Estonian Ticks. PLoS ONE. 2012;7(12).
449. Enayati AA, Asgarian F, Amouei A, Esfandiari B, Oshaghi M, Hemingway J, et al. First records of ornithodoros sp. soft ticks from an endemic relapsing fever region in northern Iran. HealthMED. 2012;6(6):1911-6.
450. Embers ME, Lopez JE. Immune resistance by relapsing fever spirochetes. The Pathogenic Spirochetes: Strategies for Evasion of Host Immunity and Persistence. 97814614540452012. p. 173-91.
451. El-Bahnsawy MM, Labib NA, Abdel-Fattah MAH, Ibrahim AM, Morsy TA. Louse and tick borne relapsing fevers. Journal of the Egyptian Society of Parasitology. 2012;42(3):625-38.
452. Drancourt M. Relapsing Fever and Borrelioses. Hunter's Tropical Medicine and Emerging Infectious Disease: Ninth Edition2012. p. 602-6.
453. Diatta G, Souidi Y, Granjon L, Arnathau C, Durand P, Chauvancy G, et al. Epidemiology of Tick-Borne Borreliosis in Morocco. PLoS Neglected Tropical Diseases. 2012;6(9).
454. Centers for Disease C, Prevention. Tickborne relapsing fever in a mother and newborn child--Colorado, 2011. MMWR Morbidity and mortality weekly report. 2012;61(10):174-6.
455. Bottieau E, Verbruggen E, Aubry C, Socolovschi C, Vlieghe E. Meningoencephalitis complicating relapsing fever in traveler returning from Senegal. Emerging Infectious Diseases. 2012;18(4).
456. Teglas MB, Mapes S, Hodzic E, Nieto NC. Co-infection of Ornithodoros coriaceus with the relapsing fever spirochete, Borrelia coriaceae, and the agent of epizootic bovine abortion. Medical and Veterinary Entomology. 2011;25(3):337-43.
457. Takano A, Fujita H, Kadosaka T, Konnai S, Tajima T, Watanabe H, et al. Characterization of reptile-associated Borrelia sp. in the vector tick, Amblyomma geoemydae, and its association with Lyme disease and relapsing fever Borrelia spp. Environ Microbiol Rep. 2011;3(5):632-7.
458. Shapiro ED, Gerber MA. Borrelia Infections: Lyme Disease and Relapsing Fever. Infectious Diseases of the Fetus and Newborn Infant2011. p. 564-76.
459. Reller ME, Clemens EG, Schachterle SE, Mtove GA, Sullivan DJ, Dumler JS. Multiplex 5′ nuclease-quantitative PCR for diagnosis of relapsing fever in a large Tanzanian cohort. Journal of Clinical Microbiology. 2011;49(9):3245-9.
460. Reis C, Cote M, Paul REL, Bonnet S. Questing ticks in suburban forest are infected by at least six tick-borne pathogens. Vector-Borne and Zoonotic Diseases. 2011;11(7):907-16.

461. Platonov AE, Karan LS, Kolyasnikova NM, Makhneva NA, Toporkova MG, Maleev VV, et al. Humans infected with relapsing fever spirochete borrelia miyamotoi, Russia. Emerging Infectious Diseases. 2011;17(10):1816-23.
462. Petri Jr WA. Relapsing Fever and Other Borrelia Infections. Goldman's Cecil Medicine: Twenty Fourth Edition. 22011. p. 1935-7.
463. Parola P, Ryelandt J, Mangold AJ, Mediannikov O, Guglielmone AA, Raoult D. Relapsing fever borrelia in Ornithodoros ticks from Bolivia. Annals of Tropical Medicine and Parasitology. 2011;105(5):407-11.
464. Parola P, Diatta G, Socolovschi C, Mediannikov O, Tall A, Bassene H, et al. Tick-borne relapsing fever borreliosis, rural senegal. Emerging Infectious Diseases. 2011;17(5):883-5.
465. Padgett KA, Bonilla DL. Novel exposure sites for nymphal Ixodes pacificus within picnic areas. Ticks and Tick-borne Diseases. 2011;2(4):191-5.
466. Lopez JE, McCoy BN, Krajacich BJ, Schwan TG. Acquisition and subsequent transmission of borrelia hermsii by the soft tick ornithodoros hermsi. Journal of Medical Entomology. 2011;48(4):891-5.
467. Hubálek Z, Rudolf I. Systematic Survey of Zoonotic and Sapronotic Microbial Agents. Microbial Zoonoses and Sapronoses. 2011:129-297.
468. Hoekstra K, Kelly M. Elevated troponin and Jarish-Herxheimer reaction in tick borne relapsing fever. Clinical Chemistry. 2011;57(10):A165-A6.
469. Garcia-Soler P, Nunez-Cuadros E, Milano-Manso G, Ruiz Sanchez P. Severe Jarisch-Herxheimer reaction in tick-borne relapsing fever. [Spanish]. Enfermedades Infecciosas y Microbiologia Clinica. 2011;29(9):710-1.
470. Fritzen CM, Huang J, Westby K, Freye JD, Dunlap B, Yabsley MJ, et al. Infection Prevalences of Common Tick-borne Pathogens in Adult Lone Star Ticks (Amblyomma americanum) and American Dog Ticks (Dermacentor variabilis) in Kentucky. Am J Trop Med Hyg. 2011;85(4):718-23.
471. Dietrich M, Gómez-Díaz E, McCoy KD. Worldwide distribution and diversity of seabird ticks: Implications for the ecology and epidemiology of tick-borne pathogens. Vector-Borne and Zoonotic Diseases. 2011;11(5):453-70.
472. de Verdiére NC, Hamane S, Assous MV, Sertour N, Ferquel E, Cornet M. Tickborne relapsing fever caused by Borrelia persica, Uzbekistan and Tajikistan. Emerging Infectious Diseases. 2011;17(7):1325-7.
473. Borgoyakov VY, Fomenko NV, Panov VV, Chikova ED. Infestation of taiga ticks with borrelias in the territory of Novosibirsk Scientific Center (Siberian Branch, Russian Academy of Sciences). Entomological Review. 2011;91(3):396-404.
474. Barbour AG. Relapsing Fever and other Borrelia Diseases. Tropical Infectious Diseases2011. p. 295-302.
475. Wilhelmsson P, Fryland L, Börjesson S, Nordgren J, Bergström S, Ernerudh J, et al. Prevalence and Diversity of Borrelia Species in Ticks That Have Bitten Humans in Sweden ▿ . J Clin Microbiol. 2010;48(11):4169-76.
476. Tokarz R, Jain K, Bennett A, Briese T, Lipkin WI. Assessment of Polymicrobial Infections in Ticks in New York State. Vector Borne Zoonotic Dis. 2010;10(3):217-21.
477. Takano A, Goka K, Une Y, Shimada Y, Fujita H, Shiino T, et al. Isolation and characterization of a novel Borrelia group of tick-borne borreliae from imported reptiles and their associated ticks. Environmental Microbiology. 2010;12(1):134-46.
478. Sidi G, Schwartz E. Relapsing Fever. Tropical Diseases in Travelers2010. p. 169-74.

479. Scott MC, Rosen ME, Hamer SA, Baker E, Edwards E, Crowder C, et al. High-Prevalence Borrelia miyamotoi scapin Wild Turkeys (Meleagris gallopavo) in Tennessee. Journal of Medical Entomology. 2010;47(6):1238-42.
480. Schwan TG. Investigations of Relapsing Fever at Home and Abroad. St Georgiev V, Zoon KC, editors2010. 101-6 p.
481. Salim abadi Y, Telmadarraiy Z, Vatandoost H, Chinikar S, Oshaghi M, Moradi M, et al. Hard Ticks on Domestic Ruminants and their Seasonal Population Dynamics in Yazd Province, Iran. Iran J Arthropod Borne Dis. 2010;4(1):66-71.
482. Nasibeh HV, Zakkyeh T, Vatandoost H, Reza YEM, Morteza HV, Ali OM. Survey of tick species parasiting domestic ruminants in Ghaemshahr county, Mazandaran province, Iran. Asian Pacific Journal of Tropical Medicine. 2010;3(10):804-6.
483. Herrero JA, García-Vázquez E, Hernández A, Gómez J. Borrelia infections: Lyme disease and recurrent fever. Medicine. 2010;10(57):3903-9.
484. Cutler SJ. Relapsing fever - A forgotten disease revealed. Journal of Applied Microbiology. 2010;108(4):1115-22.
485. Burrascano JJ. Relapsing fever. Clinical Infectious Disease2010. p. 1135-8.
486. Bouattour A, Garnier M, M'Ghirbi Y, Sarih M, Gern L, Ferquel E, et al. Borrelia crocidurae Infection of Ornithodoros erraticus (Lucas, 1849) Ticks in Tunisia. Vector-Borne and Zoonotic Diseases. 2010;10(9):825-30.
487. Borgoyakov VY, Fomenko NV, Panov VV, Chikova ED. Study on the infection of taiga ticks with borrelia in the territory of Novosibirsk Scientific center SB PAS. Parazitologiya. 2010;44(6):543-56.
488. Bermúdez C SE, Miranda C RJ, Smith C D. Ticks species (Ixodida) in the Summit Municipal Park and adjacent areas, Panama City, Panama. Experimental and Applied Acarology. 2010;52(4):439-48.
489. Barmaki A, Rafinejad J, Vatandoost H, Telmadarraiy Z, Mohtarami F, Leghaei SH, et al. Study on Presence of Borrelia persica in Soft Ticks in Western Iran. Iranian Journal of Arthropod-Borne Diseases. 2010;4(2):19-25.
490. Balicer RD, Mimouni D, Bar-Zeev Y, Levine H, Davidovitch N, Ankol OH, et al. Post exposure prophylaxis of tick-borne relapsing fever. European Journal of Clinical Microbiology and Infectious Diseases. 2010;29(3):253-8.
491. (EFSA) EFSA. Scientific Opinion on Geographic Distribution of Tick-borne Infections and their Vectors in Europe and the other Regions of the Mediterranean Basin. EFSA Journal. 2010;8(9).
492. Takano A, Muto M, Sakata A, Ogasawara Y, Ando S, Hanaoka N, et al. Relapsing fever spirochete in seabird tick, Japan. Emerging Infectious Diseases. 2009;15(9):1528-30.
493. Socolovschi C, Mediannikov O, Raoult D, Parola P. UPDATE ON TICK-BORNE BACTERIAL DISEASES IN EUROPE. Parasite. 2009;16(4):259-73.
494. Schwan TG, Raffel SJ, Schrumpf ME, Webster LS, Marques AR, Spano R, et al. Tick-borne relapsing fever and Borrelia hermsii, Los Angeles County, California, USA. Emerging Infectious Diseases. 2009;15(7):1026-31.
495. Schwan TG, Raffel SJ, Schrumpf ME, Gill JS, Piesman J. Characterization of a novel relapsing fever spirochete in the midgut, coxal fluid, and salivary glands of the bat tick carios kelleyi. Vector-Borne and Zoonotic Diseases. 2009;9(6):643-7.
496. Sarih M, Garnier M, Boudebouch N, Bouattour A, Rihani A, Hassar M, et al. Borrelia hispanica relapsing fever, Morocco. Emerging Infectious Diseases. 2009;15(10):1626-9.

497. Moemenbellah-Fard MD, Benafshi O, Rafinejad J, Ashraf H. Tick-borne relapsing fever in a new highland endemic focus of western Iran. Annals of Tropical Medicine and Parasitology. 2009;103(6):529-37.
498. Million M, Cazorla C, Doudier B, Scola BL, Parola P, Drancourt M, et al. Molecular identification of Borrelia crocidurae in a patient returning from Senegal. BMJ Case Reports. 2009.
499. Meerburg BG, Singleton GR, Kijlstra A. Rodent-borne diseases and their risks for public health Rodent-borne diseases and their risks for public health. Critical Reviews in Microbiology. 2009;35(3):221-70.
500. Masoumi Asl H, Goya MM, Vatandoost H, Zahraei SM, Mafi M, Asmar M, et al. The epidemiology of tick-borne relapsing fever in Iran during 1997-2006. Travel Medicine and Infectious Disease. 2009;7(3):160-4.
501. Martins TF, Spolidorio MG, Batista TCA, Oliveira IAS, Yoshinari NH, Labruna MB. Occurrence of ticks (Acari: Ixodidae) in the municipality of Goiatins, Tocantins. Revista Brasileira de Parasitologia Veterinaria. 2009;18(2):50-2.
502. Mahram M, Ghavami MB. Congenital tick-borne relapsing fever: Report of a case with transplacental transmission in the Islamic Republic of Iran. Eastern Mediterranean Health Journal. 2009;15(3):761-4.
503. Larsson C, Andersson M, Bergström S. Current issues in relapsing fever. Current Opinion in Infectious Diseases. 2009;22(5):443-9.
504. Guo BP, Teneberg S, Münch R, Terunuma D, Hatano K, Matsuoka K, et al. Relapsing fever Borrelia binds to neolacto glycans and mediates rosetting of human erythrocytes. Proceedings of the National Academy of Sciences of the United States of America. 2009;106(46):19280-5.
505. Georgiev VS. Tick-Borne Bacterial, Rickettsial, Spirochetal, and Protozoal Diseases. National Institute of Allergy and Infectious Diseases, NIH. 2009:197-220.
506. Evans NJ, Bown K, Timofte D, Simpson VR, Birtles RJ. Fatal borreliosis in bat caused by relapsing fever spirochete, United Kingdom. Emerging Infectious Diseases. 2009;15(8):1331-3.
507. Dana AN. Diagnosis and treatment of tick infestation and tick-borne diseases with cutaneous manifestations. Dermatologic Therapy. 2009;22(4):293-326.
508. Cutler SJ, Abdissa A, Trape JF. New concepts for the old challenge of African relapsing fever borreliosis. Clinical Microbiology and Infection. 2009;15(5):400-6.
509. Castilla-Guerra L, Alvarez-Suero J, Fernandez-Moreno MDC, Fontana ER. Tick-borne relapsing fever: Conjunctival haemorrhages. BMJ Case Reports. 2009.
510. Bitam I, Raoult D. Other tick-borne diseases in Europe. Curr Probl Dermatol. 2009;37:130-54.
511. Barbour AG, Bunikis J, Travinsky B, Hoen AG, Diuk-Wasser MA, Fish D, et al. Niche partitioning of Borrelia burgdorferi and Borrelia miyamotoi in the same tick vector and mammalian reservoir species. American Journal of Tropical Medicine and Hygiene. 2009;81(6):1120-31.
512. Assous MV, Wilamowski A. Relapsing fever borreliosis in Eurasia - Forgotten, but certainly not gone! Clinical Microbiology and Infection. 2009;15(5):407-14.
513. Socolovschi C, Doudier B, Pages F, Parola P. Ticks and human tick-borne diseases in Africa. [French]. Medecine Tropicale. 2008;68(2):119-33.
514. Pouladfar GR, Alborzi A, Pourabbas B. Tick-borne relapsing fever, a neglected cause of fever in fars province. Iranian Journal of Medical Sciences. 2008;33(3):177-9.

515. Patrat-Delon S, Drogoul AS, Le Ho H, Biziraguzenyuka J, Rabier V, Arvieux C, et al. Recurrent tick-borne fever: A possible diagnosis in patients returning from Senegal. Medecine et Maladies Infectieuses. 2008;38(7):396-9.
516. Larsson C, Bergström S. A novel and simple method for laboratory diagnosis of relapsing Fever borreliosis. Open Microbiol J. 2008;2:10-2.
517. Lak SS, Vatandoost H, Telmadarraiy Z, Mahdi RE, Kia EB. Seasonal Activity of Ticks and their Importance in Tick-Borne Infectious Diseases in West Azerbaijan, Iran. Iranian Journal of Arthropod-Borne Diseases. 2008;2(2):28-34.
518. Gill JS, Ullmann AJ, Loftis AD, Schwan TG, Raffel SJ, Schrumpf ME, et al. Novel relapsing fever spirochete in bat tick. Emerging Infectious Diseases. 2008;14(3):522-3.
519. Fibeger EA, Erickson QL, Weintraub BD, Elston DM. Larval tick infestation: A case report and review of tick-borne disease. Cutis. 2008;82(1):38-46.
520. Dworkin MS, Schwan TG, Anderson Jr DE, Borchardt SM. Tick-Borne Relapsing Fever. Infectious Disease Clinics of North America. 2008;22(3):449-68.
521. De La Fuente J, Estrada-Pena A, Venzal JM, Kocan KM, Sonenshine DE. Overview: Ticks as vectors of pathogens that cause disease in humans and animals. Frontiers in Bioscience. 2008;13(18):6938-46.
522. Blevins SM, Greenfield RA, Bronze MS. Blood smear analysis in babesiosis, ehrlichiosis, relapsing fever, malaria, and Chagas disease. Cleveland Clinic journal of medicine. 2008;75(7):521-30.
523. Badger MS. Tick talk: unusually severe case of tick-borne relapsing fever with acute respiratory distress syndrome--case report and review of the literature. Wilderness Environ Med. 2008;19(4):280-6.
524. Aher A, Shah H, Rastogi V, Tukaram P, Choudhury R. A case report of relapsing fever. Indian Journal of Pathology and Microbiology. 2008;51(2):292-3.
525. Ahantarig A, Trinachartvanit W, Milne JR. Tick-borne pathogens and diseases of animals and humans in Thailand. Southeast Asian Journal of Tropical Medicine and Public Health. 2008;39(6):1015-32.
526. Yparraguirre LA, Machado-Ferreira E, Ullmann AJ, Piesman J, Zeidner NS, Soares CAG. A hard tick relapsing fever group spirochete in a Brazilian Rhipicephalus (Boophilus) microplus. Vector-Borne and Zoonotic Diseases. 2007;7(4):717-21.
527. Whitney MS, Schwan TG, Sultemeier KB, McDonald PS, Brillhart MN. Spirochetemia caused by Borrelia turicatae infection in 3 dogs in Texas. Veterinary Clinical Pathology. 2007;36(2):212-6.
528. Uhlmann EJ, Seed PC, Schwan TG, Storch GA. Polymerase chain reaction of tick-borne relapsing fever caused by Borrelia hermsii. Pediatric Infectious Disease Journal. 2007;26(3):267-9.
529. Nordstrand A, Bunikis I, Larsson C, Tsogbe K, Schwan TG, Nilsson M, et al. Tickborne relapsing fever diagnosis obscured by Malaria, Togo. Emerging Infectious Diseases. 2007;13(1):117-23.
530. Murphy FK, Parker S, Stokich D, Murray M, Fogelman V, Todd R, et al. Acute respiratory distress syndrome in persons with tickborne relapsing fever - Three states, 2004-2005. Morbidity and Mortality Weekly Report. 2007;56(41):1073-6.
531. McCall PJ, Hume JCC, Motshegwa K, Pignatelli P, Talbert A, Kisinza W. Does tick-borne relapsing fever have an animal reservoir in East Africa? Vector-Borne and Zoonotic Diseases. 2007;7(4):659-66.

532. Gallien S, Sarfati C, Haas L, Lagrange-Xelot M, Molina JM. Borreliosis: A rare and alternative diagnosis in travellers' febrile illness. Travel Medicine and Infectious Disease. 2007;5(4):247-50.
533. Ataliba AC, Resende JS, Yoshinari N, Labruna MB. Isolation and molecular characterization of a Brazilian strain of Borrelia anserina, the agent of fowl spirochaetosis. Research in Veterinary Science. 2007;83(2):145-9.
534. Aghighi Z, Assmar M, Piazak N, Javadian E, Rashti MAS, Kia EB, et al. Distribution of Soft Ticks and Their Natural Infection with Borrelia in a Focus of Relapsing Fever in Iran. Iranian Journal of Arthropod-Borne Diseases. 2007;1(2):14-8.
535. Vial L, Durand P, Arnathau C, Halos L, Diatta G, Trape JF, et al. Molecular divergences of the Ornithodoros sonrai soft tick species, a vector of human relapsing fever in West Africa. Microbes and Infection. 2006;8(11):2605-11.
536. Vial L, Diatta G, Tall A, Hadj Ba E, Bouganali H, Durand P, et al. Incidence of tick-borne relapsing fever in west Africa: longitudinal study. Lancet. 2006;368(9529):37-43.
537. Tordini G, Giaccherini R, Corbisiero R, Zanelli G. Relapsing fever in a traveller from Senegal: determination of Borrelia species using molecular methods. Transactions of the Royal Society of Tropical Medicine and Hygiene. 2006;100(10):992-4.
538. Rebaudet S, Parola P. Epidemiology of relapsing fever borreliosis in Europe. FEMS Immunology and Medical Microbiology. 2006;48(1):11-5.
539. Mun J, Eisen RJ, Eisen L, Lane RS. Detection of a Borrelia miyamotoi sensu lato relapsing-fever group spirochete from Ixodes pacificus in California. Journal of Medical Entomology. 2006;43(1):120-3.
540. Loehr VJT, Henen BT, Hofmeyr MD. Tick infestations in the Namaqualand speckled padloper, Homopus signatus signatus (Gmelin, 1789). African Zoology. 2006;41(2):170-7.
541. Lim LL, Rosenbaum JT. Borrelia Hermsii Causing Relapsing Fever and Uveitis. American Journal of Ophthalmology. 2006;142(2):348-9.
542. Larsson C, Andersson M, Pelkonen J, Guo BP, Nordstrand A, Bergström S. Persistent brain infection and disease reactivation in relapsing fever borreliosis. Microbes and Infection. 2006;8(8):2213-9.
543. Larsson C, Andersson M, Guo BP, Nordstrand A, Hägerstrand I, Carlsson S, et al. Complications of pregnancy and transplacental transmission of relapsing-fever borreliosis. Journal of Infectious Diseases. 2006;194(10):1367-74.
544. Koehler W. Killed in action: Microbiologists and clinicians as victims of their occupation part 4: Tick-borne relapsing fever, Malta fever, glanders, SARS. IJMM International Journal of Medical Microbiology. 2006;296(1):1-4.
545. Jensenius M, Parola P, Raoult D. Threats to international travellers posed by tick-borne diseases. Travel Medicine and Infectious Disease. 2006;4(1):4-13.
546. Heerdink G, Petit PLC, Hofwegen H, Van Genderen PJJ. A patient with fever following a visit to the tropics: Tick-borne relapsing fever discovered in a thick blood smear preparation. Nederlands Tijdschrift voor Geneeskunde. 2006;150(43):2386-9.
547. Hasin T, Davidovitch N, Cohen R, Dagan T, Romem A, Orr N, et al. Postexposure treatment with doxycycline for the prevention of tick-borne relapsing fever. New England Journal of Medicine. 2006;355(2):148-55.
548. Halperin T, Orr N, Cohen R, Hasin T, Davidovitch N, Klement E, et al. Detection of relapsing fever in human blood samples from Israel using PCR targeting the glycerophosphodiester phosphodiesterase (GlpQ) gene. Acta Tropica. 2006;98(2):189-95.
549. Fall AL, Ndiaye O, Gueye M, Sylla A, Diouf S, Moreira C, et al. Child fever in tropical area: don't forget Borreliosis. Archives de Pediatrie. 2006;13(11):1461-2.

550. Cutler SJ. Possibilities for relapsing fever reemergence. Emerging Infectious Diseases. 2006;12(3):369-74.
551. Croft AM, Jackson CJ, Darbyshire AH. Doxycycline for the prevention of tick-borne relapsing fever [1]. New England Journal of Medicine. 2006;355(15):1614.
552. Assous MV, Wilamowski A, Bercovier H, Marva E. Molecular characterization of tickborne relapsing fever Borrelia, Israel. Emerging Infectious Diseases. 2006;12(11):1740-3.
553. Wyplosz B, Mihaila-Amrouche L, Baixench M-T, Bigel M-L, Berardi-Grassias L, Fontaine C, et al. Imported tickborne relapsing fever, France. Emerging Infectious Diseases. 2005;11(11):1801-3.
554. Sidi G, Davidovitch N, Balicer RD, Anis E, Grotto I, Schwartz E. Tickborne relapsing fever in Israel. Emerging Infectious Diseases. 2005;11(11):1784-6.
555. Scott JC, Wright DJM, Cutler SJ. Typing African relapsing fever spirochetes. Emerging Infectious Diseases. 2005;11(11):1722-9.
556. Roscoe C, Epperly T. Tick-borne relapsing fever. American Family Physician. 2005;72(10):2039-44.
557. Pichon B, Rogers M, Egan D, Gray J. Blood-meal analysis for the identification of reservoir hosts of tick-borne pathogens in Ireland. Vector Borne Zoonotic Dis. 2005;5(2):172-80.
558. Mayegga E, Ljøstad U, Mygland Å, Monstad P. Absence of focal neurological involvement in tick-borne relapsing fever in northern Tanzania. European Journal of Neurology. 2005;12(6):449-52.
559. Lin T, Gao L, Seyfang A, Oliver JH. 'Candidatus Borrelia texasensis', from the American dog tick Dermacentor variabilis. Int J Syst Evol Microbiol. 2005;55(Pt 2):685-93.
560. Lane RS, Mun J, Parker JM, White M. Columbian black-tailed deer (Odocoileus hemionus columbianus) as hosts for Borrelia spp. in northern California. Journal of Wildlife Diseases. 2005;41(1):115-25.
561. Holzer BR. Tick borne diseases. Therapeutische Umschau. 2005;62(11):757-63.
562. Gil H, Barral M, Escudero R, García-Pérez AL, Anda P. Identification of a new Borrelia species among small mammals in areas of Northern Spain where lyme disease is endemic. Applied and Environmental Microbiology. 2005;71(3):1336-45.
563. Escudero-Nieto R, Guerrero-Espejo A. Diseases produced by Borrelia. Enfermedades Infecciosas y Microbiologia Clinica. 2005;23(4):232-40.
564. Bunikis J, Barbour AG. Third Borrelia species in white-footed mice [4]. Emerging Infectious Diseases. 2005;11(7):1150-1.
565. Buckingham SC. Tick-borne infections in children: Epidemiology, clinical manifestations, and optimal management strategies. Pediatric Drugs. 2005;7(3):163-76.
566. Bratton RL, Corey R. Tick-borne disease. Am Fam Physician. 2005;71(12):2323-30.
567. Brahim H, Perrier-Gros-Claude JD, Postic D, Baranton G, Jambou R. Identifying relapsing fever Borrelia, Senegal. Emerging Infectious Diseases. 2005;11(3):474-5.
568. Barbour AG. Relapsing fever. Goodman JL, Dennis DT, Sonenshine DE, editors2005. 268-91 p.
569. Varela AS, Luttrell MP, Howerth EW, Moore VA, Davidson WR, Stallknecht DE, et al. First Culture Isolation of Borrelia lonestari, Putative Agent of Southern Tick-Associated Rash Illness. J Clin Microbiol. 2004;42(3):1163-9.
570. Telmadarraiy Z, Bahrami A, Vatandoost H. A survey on fauna of ticks in west Azerbaijan province, Iran. Iranian Journal of Public Health. 2004;33(4):65-9.
571. Suss J, Fingerle V, Hunfeld KP, Schrader C, Wilske B. Tick-borne human pathogenic microorganisms found in Europe and those considered nonpathogenic. Part II: Bacteria,

parasites and mixed infections. [German]. Bundesgesundheitsblatt, Gesundheitsforschung, Gesundheitsschutz. 2004;47(5):470-86.
572. Mitani H, Talbert A, Fukunaga M. New world relapsing fever Borrelia found in Ornithodoros porcinus ticks in Central Tanzania. Microbiology and Immunology. 2004;48(7):501-5.
573. Güner ES, Watanabe M, Hashimoto N, Kadosaka T, Kawamura Y, Ezaki T, et al. Borrelia turcica sp. nov., isolated from the hard tick Hyalomma aegyptium in Turkey. International Journal of Systematic and Evolutionary Microbiology. 2004;54(5):1649-52.
574. Gangaidzo IT. Forgotten diseases: relapsing fever. The Central African journal of medicine. 2004;50(7-8):73-5.
575. Fritz CL, Bronson LR, Smith CR, Schriefer ME, Tucker, Jr., Schwan TG. Isolation and characterization of Borrelia hermsii associated with two foci of tick-borne relapsing fever in California. Journal of Clinical Microbiology. 2004;42(3):1123-8.
576. Clark K. Borrelia Species in Host-Seeking Ticks and Small Mammals in Northern Florida. J Clin Microbiol. 2004;42(11):5076-86.
577. Brouqui P, Bacellar F, Baranton G, Birtles RJ, Bjoërsdorff A, Blanco JR, et al. Guidelines for the diagnosis of tick-borne bacterial diseases in Europe. Clinical Microbiology and Infection. 2004;10(12):1108-32.
578. Singh-Behl D, La Rosa SP, Tomecki KJ. Tick-borne infections. Dermatol Clin. 2003;21(2):237-44, v.
579. Schwan TG, Policastro PF, Miller Z, Thompson RL, Damrow T, Keirans JE. Tick-borne relapsing fever caused by Borrelia hermsii, Montana. Emerging Infectious Diseases. 2003;9(9):1151-4.
580. McConnell J. Tick-borne relapsing fever under-reported. The Lancet infectious diseases. 2003;3(10):604.
581. Lecompte Y, Trape JF. West african tick-borne relapsing fever. Annales de Biologie Clinique. 2003;61(5):541-8.
582. Kisinza WN, McCall PJ, Mitani H, Talbert A, Fukunaga M, Kisinza WN, et al. A newly identified tick-borne Borrelia species and relapsing fever in Tanzania. Lancet. 2003;362 North American Edition(9392):1283-4.
583. Güner ES, Hashimoto N, Kadosaka T, Imai Y, Masuzawa T. A novel, fast-growing Borrelia sp. isolated from the hard tick Hyalomma aegyptium in Turkey. Microbiology. 2003;149(9):2539-44.
584. Centers for Disease C, Prevention. Tickborne relapsing fever outbreak after a family gathering: New Mexico, August 2002. Morbidity and Mortality Weekly Report. 2003;52(34):809-12.
585. Barbour AG. Antigenic Variation in Borrelia. Relapsing Fever and Lyme Borreliosis. Antigenic Variation2003. p. 319-56.
586. Webster G, Schiffman JD, Dosanjh AS, Amieva MR, Gans HA, Sectish TC. Jarisch-Herxheimer reaction associated with ciprofloxacin administration for tick-borne relapsing fever. Pediatric Infectious Disease Journal. 2002;21(6):571-3.
587. Wallace MR, Hale BR, Utz GC, Olson PE, Earhart KC, Thornton SA, et al. Endemic infectious diseases of Afghanistan. Clinical Infectious Diseases. 2002;34:S171-S207.
588. Walker RL, Read DH, Hayes DC, Nordhausen RW. Equine abortion associated with the Borrelia parkeri-B-turicatae tick-borne relapsing fever spirochete group. Journal of Clinical Microbiology. 2002;40(4):1558-62.

589. Thomas NJ, Bunikis J, Barbour AG, Wolcott MJ. Fatal spirochetosis due to a relapsing fever-like Borrelia sp. in a northern spotted owl. Journal of wildlife diseases. 2002;38(1):187-93.
590. Schwan TG, Piesman J. Vector interactions and molecular adaptations of Lyme disease and relapsing fever spirochetes associated with transmission by ticks. Emerging Infectious Diseases. 2002;8(2):115-21.
591. Paul WS, Maupin G, Scott-Wright AO, Craven RB, Dennis DT. Outbreak of tick-borne relapsing fever at the North Rim of the Grand Canyon: Evidence for effectiveness of preventive measures. American Journal of Tropical Medicine and Hygiene. 2002;66(1):71-5.
592. Pantanowitz L, Telford Iii SR, Cannon ME. Tick-borne diseases in transfusion medicine. Transfusion Medicine. 2002;12(2):85-106.
593. Mitiku K, Mengistu G. Relapsing fever in Gondar, Ethiopia. East African medical journal. 2002;79(2):85-7.
594. Fraenkel C-J, Garpmo U, Berglund J. Determination of novel Borrelia genospecies in Swedish Ixodes ricinus ticks. Journal of clinical microbiology. 2002;40(9):3308-12.
595. Dworkin MS, Shoemaker PC, Fritz CL, Dowell ME, Anderson Jr DE. The epidemiology of tick-borne relapsing fever in the United States. American Journal of Tropical Medicine and Hygiene. 2002;66(6):753-8.
596. Dworkin MS, Schwan TG, Anderson Jr DE. Tick-borne relapsing fever in North America. Medical Clinics of North America. 2002;86(2):417-33.
597. Davis H, Vincent JM, Lynch J. Tick-Borne relapsing fever caused by Borrelia turicatae. Pediatric Infectious Disease Journal. 2002;21(7):703-5.
598. Arshi S, Majidpoor A, Sadeghi H, Asmar M, Emdadi D, Derakhshan MH. Relapsing fever in Ardabil, a northwestern province of Iran. Archives of Iranian Medicine. 2002;5(3):141-5.
599. Ali Zaidi S, Singer C. Gastrointestinal and hepatic manifestations of tickborne diseases in the United States. Clinical Infectious Diseases. 2002;34(9):1206-12.
600. Parola P, Raoult D. Ticks and tickborne bacterial diseases in humans: An emerging infectious threat. Clinical Infectious Diseases. 2001;32(6):897-928.
601. Gage KL, Eggleston ME, Gilmore RD, Dolan MC, Montenieri JA, Tanda DT, et al. Isolation and characterization of Borrelia parkeri in Ornithodoros parkeri (Ixodida : Argasidae) collected in Colorado. Journal of Medical Entomology. 2001;38(5):665-74.
602. Fukunaga M, Ushijima Y, Aoki LY, Talbert A. Detection of Borrelia duttonii, a tick-borne relapsing fever agent in central Tanzania, within ticks by flagellin gene-based nested polymerase chain reaction. Vector borne and zoonotic diseases (Larchmont, NY). 2001;1(4):331-8.
603. Feldman KA, Gage K, Maupin G, Riddle D, Klouse P, Schriefer M, et al. Tick-borne relapsing fever in Clark County, Nevada, October 2000. Clinical Infectious Diseases. 2001;33(7):1244-.
604. Burgdorfer W. Arthropod-borne spirochetoses: a historical perspective. Eur J Clin Microbiol Infect Dis. 2001;20(1):1-5.
605. Soares CO, Ishikawa MM, Fonseca AH, Yoshinari NH. Borrelioses, agents and vectors: A review. Pesquisa Veterinaria Brasileira. 2000;20(1):1-19.
606. Van Dam AP, Van Gool T, Wetsteyn JCFM, Dankert J. Tick-borne relapsing fever imported from West Africa: Diagnosis by quantitative buffy coat analysis and in vitro culture of Borrelia crocidurae. Journal of Clinical Microbiology. 1999;37(6):2027-30.

607. Shamaei-Tousi A, Martin P, Bergh A, Burman N, Brännström T, Bergström S. Erythrocyte-aggregating relapsing fever spirochete Borrelia crocidurae induces formation of microemboli. Journal of Infectious Diseases. 1999;180(6):1929-38.
608. Estrada-Peña A, Jongejan F. Ticks feeding on humans: A review of records on human-biting Ixodoidea with special reference to pathogen transmission. Experimental and Applied Acarology. 1999;23(9):685-715.
609. Cleary M, Theis J. Identification of a novel strain of Borrelia hermsii in a previously undescribed northern California focus. American Journal of Tropical Medicine and Hygiene. 1999;60(6):883-7.
610. Chatel G, Gulletta M, Matteelli A, Marangoni A, Signorini L, Oladeji O, et al. Diagnosis of tick-borne relapsing fever by the quantitative buffy coat fluorescence method. American Journal of Tropical Medicine and Hygiene. 1999;60(5):738-9.
611. Trevejo RT, Schriefer ME, Gage KL, Safranek TJ, Orloski KA, John Pape W, et al. An interstate outbreak of tick-borne relapsing fever among vacationers at a Rocky Mountain cabin. American Journal of Tropical Medicine and Hygiene. 1998;58(6):743-7.
612. Talbert A, Nyange A, Molteni F. Spraying tick-infested houses with lambda-cyhalothrin reduces the incidence of tick-borne relapsing fever in children under five years old. Transactions of the Royal Society of Tropical Medicine and Hygiene. 1998;92(3):251-3.
613. Schwan TG, Hinnebusch BJ. Bloodstream- versus tick-associated variants of a relapsing fever bacterium. Science. 1998;280(5371):1938-40.
614. Rodhain F. Joseph Désiré Tholozan and the Persian relapsing fever. Histoire des sciences médicales. 1998;32(3):309-13.
615. Radetsky M. The emerging spectrum of tickborne infections. Current Opinion in Infectious Diseases. 1998;11(3):313-8.
616. Dworkin MS, Anderson DE, Jr., Schwan TG, Shoemaker PC, Banerjee SN, Kassen BO, et al. Tick-borne relapsing fever in the northwestern United States and southwestern Canada. Clin Infect Dis. 1998;26(1):122-31.
617. Dennis DT, Palmer SR, Lord S, Simpson DIH. Borreliosis (relapsing fever)1998. 17-21 p.
618. Cadavid D, Barbour AG. Neuroborreliosis during relapsing fever: Review of the clinical manifestations, pathology, and treatment of infections in humans and experimental animals. Clinical Infectious Diseases. 1998;26(1):151-64.
619. Burman N, Shamaei-Tousi A, Bergstrom S. The spirochete Borrelia crocidurae causes erythrocyte rosetting during relapsing fever. Infection and Immunity. 1998;66(2):815-9.
620. Banerjee SN, Banerjee M, Fernando K, Burgdorfer W, Schwan TG. Tick-borne relapsing fever in British Columbia, Canada: First isolation of Borrelia hermsii. Journal of Clinical Microbiology. 1998;36(12):3505-8.
621. Abdul-Wahid S, James BA. Relapsing fever [5]. Laboratory Medicine. 1998;29(7):394.
622. Westenfeld FW. Clinical pathology rounds: Relapsing fever in a recent visitor to Africa. Laboratory Medicine. 1997;28(7):436-8.
623. Van Holten J, Tiems J, Jongen VHWM. Neonatal Borrelia duttoni infection: A report of three cases. Tropical Doctor. 1997;27(2):115-6.
624. Jongen VHWM, Van Roosmalen J, Tiems J, Van Holten J, Wetsteyn JCFM. Tick-borne relapsing fever and pregnancy outcome in rural Tanzania. Acta Obstetricia et Gynecologica Scandinavica. 1997;76(9):834-8.
625. Dupont HT, La Scola B, Williams R, Raoult D. A focus of tick-borne relapsing fever in southern Zaire. Clinical Infectious Diseases. 1997;25(1):139-44.

626. Trape JF, Godeluck B, Diatta G, Rogier C, Legros F, Albergel J, et al. The spread of tick-borne borreliosis in West Africa and its relationship to sub-Saharan drought. American Journal of Tropical Medicine and Hygiene. 1996;54(3):289-93.
627. Schwan TG, Schrumpf ME, Hinnebusch BJ, Anderson DE, Jr., Konkel ME. GlpQ: an antigen for serological discrimination between relapsing fever and Lyme borreliosis. J Clin Microbiol. 1996;34(10):2483-92.
628. Poulsen LW, Iversen G. Relapsing fever: A differential diagnosis to malaria. Scandinavian Journal of Infectious Diseases. 1996;28(4):419-20.
629. Oliver MR, Liles WC, Spach DW. Relapsing fever. Wilderness and Environmental Medicine. 1996;7(1):46-55.
630. Newton JA, Pepper PV. Relapsing fever. New England Journal of Medicine. 1996;335(16):1197.
631. Anda P, SanchezYebra W, Vitutia MD, Pastrana EP, Rodriguez I, Miller NS, et al. A new Borrelia species isolated from patients with relapsing fever in Spain. Lancet. 1996;348(9021):162-5.
632. Stanek G. Borreliosis and Travel Medicine. J Travel Med. 1995;2(4):244-51.
633. Rawlings JA. An overview of tick-borne relapsing fever with emphasis on outbreaks in Texas. Texas medicine. 1995;91(5):56-9.
634. Keung YK, Cobos E, Kimbrough RC, Carver RC. Borreliosis as a Cause of fever in a woman who recently returned from Saudi Arabia. Clinical Infectious Diseases. 1995;21(2):447-8.
635. Gage KL, Ostfeld RS, Olson JG. Nonviral vector-borne zoonoses associated with mammals in the United States. Journal of Mammalogy. 1995;76(3):695-715.
636. Al-Gwaiz LA, Al-Mashhadani SA, Ayoola EA, Al-Khairy KS, Higgy KG, Al-Omair AO. Relapsing fever in Saudi Arabia: Report of two cases. Annals of Saudi Medicine. 1995;15(2):165-7.
637. Tilley PA, Azar R, Banerjee S, Bell A. Three cases of relapsing fever associated with lakeside cabins in Idaho. Canada communicable disease report = Relevé des maladies transmissibles au Canada. 1994;20(4):29-31.
638. Guberman D, Vardy DA, Klapholz L, Klaus SN. Vector-borne infections: a hazard for adventure visitors to Israel. Journal of Wilderness Medicine. 1994;5(3):254-62.
639. Godeluck B, Duplantier J-M, Ba K, Trape J-F. A longitudinal survey of Borrelia crocidurae prevalence in rodents and insectivores in Senegal. American Journal of Tropical Medicine and Hygiene. 1994;50(2):165-8.
640. Diatta G, Trape JF, Legros F, Rogier C, Duplantier JM. A comparative study of three methods of detection of Borrelia crocidurae in wild rodents in Senegal. Transactions of the Royal Society of Tropical Medicine and Hygiene. 1994;88(4):423-4.
641. Spach DH, Liles WC, Campbell GL, Quick RE, Anderson DE, Jr., Fritsche TR. Tick-borne diseases in the United States. N Engl J Med. 1993;329(13):936-47.
642. Liles WC, Spach DH. Late relapse of tick-borne relapsing fever following treatment with doxycycline. West J Med. 1993;158(2):200.
643. Hardy A. Relapsing fever. Kiple KF, editor1993. 967-70 p.
644. Colebunders R, Serrano PD, Gompel AV, Wynants H, Blot K, Van Den Enden E, et al. Imported relapsing fever in european tourists. Scandinavian Journal of Infectious Diseases. 1993;25(4):533-6.
645. Lovett MA, Goldstein EJC, Fleischmann J. Fever in a Couple Vacationing in the Mountains of Southern California. Clinical Infectious Diseases. 1992;14(6):1254-8.

646. Leker RR, Felsenstein I, Raveh D, Klaus S, Mumcuoglu KY. Ornithodoros tholozani bites: A unique clinical picture. Journal of the American Academy of Dermatology. 1992;27(6):1025-6.
647. Evans TG, Kurrus JA, Magarian S. NON-SEASONAL RELAPSING FEVER IN UTAH. Clinical Microbiology Newsletter. 1992;14(14):111-2.
648. Davis RD, Burke JP, Wright LJ, Davis RD, Burke JP, Wright LJ. Relapsing fever associated with ARDS in a parturient woman. A case report and review of the literature. CHEST. 1992;102(2):630-2.
649. Vasil'eva IS, Ershova AS, Mansurov AA, Andrianov VA, Abidov ZI, Ibragimov II, et al. Changes in the village foci of tick-borne relapsing fever in Uzbekistan over a 10-year period. Parazitologiia. 1991;25(4):323-9.
650. Trape JF, Duplantier JM, Bouganali H, Godeluck B, Legros F, Cornet JP, et al. Tick-borne borreliosis in West Africa. Lancet. 1991;337(8739):473-5.
651. Malatre I, Giocanti R, Macaigne F, Ripert C. A study of the Borrelia fever focus of Gisenyi (Rwanda). Medecine Tropicale. 1991;51(1):49-52.
652. Flanigan TP, Schwan TG, Armstrong C, Van Voris LP, Salata RA. Relapsing fever in the US Virgin Islands: a previously unrecognized focus of infection. J Infect Dis. 1991;163(6):1391-2.
653. Anonymous. Outbreak of relapsing fever--Grand Canyon National Park, Arizona, 1990. Mmwr. 1991;Morbidity and mortality weekly report. 40(18):296-7, 303.
654. Nadelman RB, Wormser GP, Sherer C. Blood transfusion-associated relapsing fever. Transfusion. 1990;30(4):380-1.
655. Floch JJ, Ndahiragije A, Nikoyagize E, Nzeyimana H, Kadende P, Kamanfu G, et al. [Relapsing fever in Burundi. First case observed since 30 years]. Bull Soc Pathol Exot. 1990;83(2):286-7.
656. Centers for Disease C. Common source outbreak of relapsing fever--California. MMWR Morbidity and mortality weekly report. 1990;39(34):579, 85-6.
657. Barclay AJG, Coulter JBS. Tick-borne relapsing fever in central Tanzania. Transactions of the Royal Society of Tropical Medicine and Hygiene. 1990;84(6):852-6.
658. Rodhain F, Fontenille D. Tick-borne relapsing fever in Madagascar: An eradicated disease?. [French]. Bulletin de la Societe de Pathologie Exotique et de ses Filiales. 1989;82(2):192-8.
659. Cortés LL, Leon FL, GÓmez-Mateos JM, SÁnchez Porto A, Obrador C. Tick-Borne Relapsing Fever in Intravenous Drug Abusers. Journal of Infectious Diseases. 1989;159(4):804.
660. Wright SW, Trott AT. North American tick-borne diseases. Annals of Emergency Medicine. 1988;17(9):964-72.
661. Melkert PWJ. Relapsing fever in pregnancy: analysis of high-risk factors. BJOG: An International Journal of Obstetrics & Gynaecology. 1988;95(10):1070-2.
662. McNamara JJ, Kay HH. Relapsing fever (Borrelia) in an adolescent tourist in Israel. Journal of Adolescent Health Care. 1988;9(5):421-3.
663. Eyckmans L. Relapsing fevers. Revue Medicale de Liege. 1988;43(15-16):530-3.
664. Vasil'eva IS, Ershova AS, Sharipov MK, Mansurov AA, Usmankhodzhaev MS. [The tick Ornithodoros papillipes count in Namangan and Andizhan Provinces and its determining factors]. Parazitologiia. 1986;20(1):3-10.
665. Spiller GW. Tick-borne relapsing fever due to Borrelia hermsii in British Columbia. Canadian Medical Association Journal. 1986;134(1):46-7.

666. Shaked Y, Maier MK, Samra Y. Relapsing fever and salmonella bacteraemia simultaneously affecting a healthy young man. Journal of Infection. 1986;13(3):308-9.
667. Lepage P, Ntahorutaba M, Bogaerts J. Neonatal relapsing fever in Rwanda. American journal of diseases of children (1960). 1986;140(2):89.
668. Kurtz SK. Relapsing fever/lyme disease multiple sclerosis. Medical Hypotheses. 1986;21(3):335-43.
669. Burgdorfer W. The enlarging spectrum of tick-borne spirochetoses: R. R. Parker Memorial Address. Reviews of infectious diseases. 1986;8(6):932-40.
670. Yagupsky P, Moses S. Neonatal Borrelia species infection (relapsing fever). American Journal of Diseases of Children. 1985;139(1):74-6.
671. Simon JW. Tick borne relapsing fever imported into the United Kingdom. Journal of the Royal Army Medical Corps. 1985;131(2):65-7.
672. Horton JM, Blaser MJ. The spectrum of relapsing fever in the Rocky Mountains. Archives of Internal Medicine. 1985;145(5):871-5.
673. Butler TC. Relapsing fever: new lessons about antibiotic action. Ann Intern Med. 1985;102(3):397-9.
674. Brasseur D. Tick-borne relapsing fever in a premature infant. Annals of Tropical Paediatrics. 1985;5(3):161-2.
675. Wengrower D, Knobler H, Gillis S, Chajek-Shaul T. Myocarditis in tick-borne relapsing fever. Journal of Infectious Diseases. 1984;149(6):1033.
676. Vasil'Eva IS, Sharipov MK, Ershova AS, Mansurov AA, Mukhitdinov AG, Ibragimov Yu I, et al. THE CONTEMPORARY STATE OF NIDI AND SICK-RATE WITH TICK-BORNE RELAPSING FEVER IN THE UZBEK-SSR USSR. Parazitologiya (St Petersburg). 1984;18(1):10-4.
677. Makwabe CM. Tick borne relapsing fever in Tanzanian children. The Central African journal of medicine. 1984;30(8):148, 50.
678. Khalil GM, Helmy N, Hoogstraal H, El-Said A. SEASONAL DYNAMICS OF ORNITHODOROS-ERRATICUS ACARI IXODOIDEA ARGASIDAE AND THE SPIROCHETE BORRELIA-CROCIDURAE IN EGYPT. Journal of Medical Entomology. 1984;21(5):536-9.
679. Rosenthal E. Relapsing fever in Cape Town. A case report. South African Medical Journal. 1982;61(21):801-2.
680. McIntosh N, Madhavan T. Zoonoses at Henry Ford Hospital. Clinical, epidemiologic, and therapeutic aspects. Henry Ford Hospital Medical Journal. 1982;30(1):7-10.
681. Hoogstraal H. Changing patterns of tickborne diseases in modern society. Annu Rev Entomol. 1981;26:75-99.
682. Le CT. Tick-borne relapsing fever in children. Pediatrics. 1980;66(6):963-6.
683. Fihn S, Larson EB. Tick-borne relapsing fever in the Pacific Northwest: An underdiagnosed illness? Western Journal of Medicine. 1980;133(3):203-9.
684. Taber LH, Feigin RD. Spirochetal infections. Pediatric Clinics of North America. 1979;26(2):377-413.
685. Mohr JA, Dimas C, Washburn D. Relapsing fever. The Journal of the Oklahoma State Medical Association. 1979;72(12):430-2.
686. Malison MD. Relapsing fever. Journal of the American Medical Association. 1979;241(26):2819-20.
687. Hoogstraal H. Ticks and spirochetes. Acta Trop. 1979;36(2):133-6.
688. Edell TA, Emerson JK, Maupin GO, Barnes AM, Vernon TM. Tick-borne relapsing fever in Colorado. Historical review and report of cases. JAMA : the journal of the American Medical Association. 1979;241(21):2279-82.

689. Linnemann Jr CC, Barber LC, Dine MS, Body AE. Tick-borne relapsing fever in the Eastern United States. American journal of diseases of children (1960). 1978;132(1):40-2.
690. Janbakhsh B, Ardelan A. The nature of sporadic cases of relapsing fever in Kazeroun area, southern Iran. Bull Soc Pathol Exot Filiales. 1977;70(6):587-9.
691. Espinoza H, McCaig N, Cutler RE. Relapsing fever in New Mexico: report of two cases. Rocky Mountain medical journal. 1977;74(6):321-3.
692. Boyer KM, Munford RS, Maupin GO, Pattison CP, Fox MD, Barnes AM, et al. Tick borne relapsing fever: an interstate outbreak originating at Grand Canyon National Park. American Journal of Epidemiology. 1977;105(5):469-79.
693. Miranpuri GS, Bindra OS, Prasad V. Tick fauna of north-western India (Acarina: Metastigmata). International Journal of Acarology. 1975;1(1):31-54.
694. Gear JHS. Tropical thrombophlebitis. The role of relapsing fever in its causation. South African Medical Journal. 1975;49(49):2057-8.
695. Peirce MA. Distribution and ecology of Ornithodoros moubata porcinus Walton (Acarina) in animal burrows in East Africa. Bulletin of Entomological Research. 1974;64(4):605-19.
696. De Zulueta J, Nasrallah S, Karam JS, Anani AR, Weatman GKS, Muir DA. Finding of tick-borne relapsing fever in jordan by the malaria eradication service. Annals of Tropical Medicine and Parasitology. 1971;65(4):491-5.
697. Thompson RS, Burgdorfer W, Russell R, Francis BJ. Outbreak of tick-borne relapsing fever in Spokane County, Washington. JAMA : the journal of the American Medical Association. 1969;210(6):1045-50.
698. Smith L, Brown TG. Relapsing fever--a case history. California medicine. 1969;110(4):322-4.
699. Goodman RL, Arndt KA, Steigbigel NH. Borrelia in Boston. Jama. 1969;210(4):722-3.
700. Fuchs PC, Oyama AA. Neonatal relapsing fever due to transplacental transmission of Borrelia. JAMA : the journal of the American Medical Association. 1969;208(4):690-2.
701. Marinkelle CJ, Grose ES. Species of borrelia from a Colombian bat (Natalus tumidirostris). Nature. 1968;218(5140):487.
702. Eisenberg S, Gunders AE, Cohen AM. Tick-borne relapsing fever in the Judean hills, including a case with massive haematuria. Transactions of the Royal Society of Tropical Medicine and Hygiene. 1968;62(5):679-81.
703. Felsenfeld O. BORRELIAE, HUMAN RELAPSING FEVER, AND PARASITE-VECTOR-HOST. Bacteriological reviews. 1965;29:46-74.
704. Mooser H. [THE PRESERVATION OF AN EGYPTIAN STRAIN OF BORRELIA CROCIDURAE IN ORNITHODORUS MOUBATA]. Acta Trop. 1963;20:369-72.
705. Anderson IG. A note on relapsing fever occurring in two Europeans. The Central African journal of medicine. 1958;4(10):444-5.
706. Ordman D. Relapsing fever in Africa. The Central African journal of medicine. 1957;3(9):347-56.
707. Babudieri B. Relapsing fever in Jordan. Bulletin of the World Health Organization. 1957;16(5):911-28.
708. Lovett WCD. Eradication of tick-borne relapsing fetter in the Somaliland protectorate by a tick destruction programme. Transactions of the Royal Society of Tropical Medicine and Hygiene. 1956;50(2):157-65.
709. Geigy R, Mooser H, Weyer F. Studies on strains of African relapsing fever from Tanganyika. Acta tropica. 1956;13(3):193-224.

710. Davis GE. The identification of spirochetes from human cases of relapsing fever by Xenodiagnosis with comments on local specificity of tick vectors. Experimental parasitology. 1956;5(3):271-5.
711. Davis GE. A relapsing fever spirochete, Borrelia mazzottii (sp. nov.) from Ornithodoros talaje from Mexico. American journal of hygiene. 1956;63(1):13-7.
712. Walton GA. Relapsing fever in the Digo District of Kenya Colony. East African medical journal. 1955;32(10):377-93.
713. Ordman D. Relapsing fever in South Africa with a record of its occurrence in Europeans. South African medical journal = Suid-Afrikaanse tydskrif vir geneeskunde. 1955;29(22):518-21.
714. Geigy R, Mooser H. Research on the epidemiology of African relapsing fever in Tanganyika. Acta tropica. 1955;12(4):327-45.
715. Davis GE. RELAPSING FEVER SPIROCHETES - THE PRESENT STATUS OF BORRELIA-VENEZUELENSIS BRUMPT AND BORRELIA-NEOTROPICALIS BATES-AND-ST-JOHN. International Bulletin of Bacteriological Nomenclature and Taxonomy. 1955;5(3):107-9.
716. Cherry JK. The prevention and treatment of tick-borne relapsing fever with special reference to aureomycin and terramycin. Transactions of the Royal Society of Tropical Medicine and Hygiene. 1955;49(6):563-73.
717. Schuhardt VT. Treatment of relapsing fever with antibiotics. Annals of the New York Academy of Sciences. 1952;55(6):1209-21.
718. Ilsley ML. Relapsing fever probably caused by Borrelia duttonii. California medicine. 1952;77(3):195-6.
719. Agarwal BL. Relapsing fever in Kashmir. Indian medical gazette. 1951;86(10):446-53.
720. Walton GA. Relapsing fever in the Meru District of Kenya. East African medical journal. 1950;27(2):94-8.
721. Narain S, Kalra SL. Streptomycin in tick-borne relapsing fever of Kashmir. Indian medical gazette. 1950;85(3):87-8.
722. Eads RB, Henderson HE, et al. Relapsing fever in Texas; distribution of laboratory confirmed cases and the arthropod reservoirs. Am J Trop Med Hyg. 1950;30(1):73-6.
723. Charters AD. Tick-borne relapsing fever in Somaliland with special reference to the blood sedimentation rate. Transactions of the Royal Society of Tropical Medicine and Hygiene. 1950;43(4):427-34.
724. McNeil E, Hinshaw WR, Kissling RE. A STUDY OF BORRELIA ANSERINA INFECTION (SPIROCHETOSIS) IN TURKEYS1. J Bacteriol. 1949;57(2):191-206.
725. Kaul S. Relapsing Fever—Tick-Borne. Account of an Outbreak in J.&K. Force, India. Ind Med Gaz. 1949;84(10):433-40.
726. Gambles RM, Coghill NF. Relapsing fever in Cyprus. Annals of tropical medicine and parasitology. 1948;42(3-4):288-303.
727. Coghill NF, Gambles RM. Discussion of methods for differentiating tick- from louse-borne relapsing fever spirochaetes. Ann Trop Med Parasitol. 1948;42(1):113-7.
728. Parsons L. Relapsing fever at Lake Tahoe, California-Nevada. American journal of clinical pathology. 1947;17(5):388-92.
729. Herms WB. VECTOR POTENTIALITIES WITH RESPECT TO THE SPREAD OF INSECT-BORNE DISEASES OF MAN IN CALIFORNIA. Calif Med. 1947;67(2):95-9.
730. Fisher DW, Edelman MH. Tick-borne relapsing fever: a case report. Bulletin of the US Army Medical Department United States Army Medical Department. 1947;7(10):901-3.
731. Coghill NF, Lawrence J, Ballantine ID. Relapsing fever in Cyrenaica. British medical journal. 1947;1(4505):637-40.

732. Tucker WAL. A report on the treatment of tick relapsing fever with sodium penicillin. East African medical journal. 1946;23:13-8.
733. Quin CE, Perkins ES. Tick-borne relapsing fever in East Africa. The Journal of tropical medicine and hygiene. 1946;49:30-2.
734. Muwazi EM. Penicillin in treatment of relapsing fever. East African medical journal. 1946;23:55-64.
735. Calero C. Relapsing fever on the Isthmus of Panama; report of 106 cases. The American journal of tropical medicine and hygiene. 1946;26(6):761-9.
736. Wood RC, Dixon KC. Tick-borne relapsing fever in cyprus. British Medical Journal. 1945;2(4424):526-8.
737. Wisseman CL. RELAPSING FEVER IN DENTON COUNTY, TEXAS - REPORT ON FINDING THE TICK, ORNITHODORUS TURICATA, NATURALLY INFECTED. American Journal of Tropical Medicine. 1945;25(4):339-42.
738. Taft WC, Pike JB. Relapsing fever; report of a sporadic outbreak, including treatment with penicillin. Journal of the American Medical Association. 1945;129:1002-5.
739. Dewar HA, Walmsley R. Relapsing fever with nephritis and subarachnoid haemorrhage. Lancet (London, England). 1945;2(6379):630.
740. Burroughs AL, Holdenried R. Recovery of Relapsing Fever Spirochetes from Ornithodoros turicata (Duges), 1876, in California. J Bacteriol. 1944;48(5):609.
741. Hamilton JB. OCULAR COMPLICATIONS IN RELAPSING FEVER*. Br J Ophthalmol. 1943;27(2):68-80.
742. Davis GE. RELAPSING FEVER: THE TICK ORNITHODOROS TURICATA AS A SPIROCHETAL RESERVOIR. Public Health Reports. 1943;58(22):839-42.
743. Davis GE. ORNITHODOROS PARKERI AND RELAPSING FEVER, SPIROCHETES IN SOUTHERN IDAHO. Public Health Reports. 1942;57(40):1501-3.
744. Davis GE, Wynns HL, Beck MD. RELAPSING FEVER: ORNITHODOROS PARKERI A VECTOR IN CALIFORNIA. Public Health Reports. 1941;56(51):2426-8.
745. Davis GE. ORNITHODOROS TURICATA AND RELAPSING FEVER SPIROCHETES IN NEW MEXICO. Public Health Reports. 1941;56(47):2258-61.
746. Davis GE. ORNITHODOROS HERMSI AND RELAPSING FEVER IN OREGON. Public Health Reports. 1941;56(41):2010-2.
747. Davis GE. ORNITHODOROS PARKERI AND RELAPSING FEVER SPIROCHETES IN UTAH. Public Health Reports. 1941;56(52):2464-8.
748. Davis GE. TICKS AND RELAPSING FEVER IN THE UNITED STATES. Public Health Reports. 1940;55(51):2347-51.
749. Davis GE. RELAPSING FEVER: ORNITHODOROS HERMSI A VECTOR IN COLORADO. Public Health Reports. 1939;54(49):2178-80.
750. Davis GE. ORNITHODOROS PARKERI: DISTRIBUTION AND HOST DATA; SPONTANEOUS INFECTION WITH RELAPSING FEVER SPIROCHETES. Public Health Reports. 1939;54(29):1345-9.
751. Reynolds FC. Relapsing Fever: Comments on Its Incidence in Nevada. Cal West Med. 1937;47(3):170-4.
752. Burns GC. Relapsing Fever in California*. Cal West Med. 1936;44(1):29-33.
753. Adler S, Theodor O. Transmission of relapsing fever by ticks in Palestine. Lancet. 1936;1:448-.
754. Herms WB, Wheeler CM. Tick transmission of california relapsing fever. Journal of Economic Entomology. 1935;28:846-55.
755. Briggs LH. Relapsing Fever*. Cal West Med. 1935;42(5):350-4.

756. Palmer JH, Crawford DJM. RELAPSING FEVER IN NORTH AMERICA, WITH REPORT OF AN OUTBREAK IN BRITISH COLUMBIA. Can Med Assoc J. 1933;28(6):643-7.
757. Nicholson FD. TICK FEVER IN PALESTINE. Br Med J. 1919;2(3077):811.
758. Ali M. Relapsing Fever *Paper read at the All-India Sub-Assistant Surgeons' onference at Agra. Ind Med Gaz. 1918;53(5):178-80.
759. Wellman FC. Human Trypanosomiasis and Spirochaetosis in Portuguese South-West Africa, with suggestions for Preventing their Spread in the Colony. J Hyg (Lond). 1906;6(3):237-45.
760. Lebredo MG. A Case of Recurrent Fever Observed in Havana. Public Health Pap Rep. 1906;32(Pt 1):238-47.
761. Kahlig P, Paris DH, Neumayr A. Louse-borne relapsing fever—A systematic review and analysis of the literature: Part 1—Epidemiology and diagnostic aspects. PLOS Neglected Tropical Diseases. 2021;15(3):e0008564.
762. Kahlig P, Neumayr A, Paris DH. Louse-borne relapsing fever—A systematic review and analysis of the literature: Part 2—Mortality, Jarisch–Herxheimer reaction, impact on pregnancy. PLOS Neglected Tropical Diseases. 2021;15(3):e0008656.
763. Warrell DA. Louse-borne relapsing fever (Borrelia recurrentisinfection). Epidemiology and Infection. 2019;147.
764. Parker CT, Garrity GM, Tindall BJ. International Code of Nomenclature of Prokaryotes. International Journal of Systematic and Evolutionary Microbiology. 2019;69(1A):S1-S111.
765. Aguero-Rosenfeld Marie E, Stanek G. 12th ed2019.
766. Blazejak K, Raulf M-K, Janecek E, Jordan D, Fingerle V, Strube C. Shifts in Borrelia burgdorferi (s.l.) geno-species infections in Ixodes ricinus over a 10-year surveillance period in the city of Hanover (Germany) and Borrelia miyamotoi-specific Reverse Line Blot detection. Parasites & Vectors. 2018;11(1).
767. AG B. Microbiology, pathogenesis, and epidemiology of relapsing fever. UpToDate. 2018.
768. Wroblewski D, Gebhardt L, Prusinski MA, Meehan LJ, Halse TA, Musser KA. Detection of Borrelia miyamotoi and other tick-borne pathogens in human clinical specimens and Ixodes scapularis ticks in New York State, 2012–2015. Ticks and Tick-borne Diseases. 2017;8(3):407-11.
769. Luz HR, Muñoz-Leal S, Almeida JCD, Faccini JLH, Labruna MB. Ticks parasitizing bats (Mammalia: Chiroptera) in the Caatinga Biome, Brazil. Revista Brasileira de Parasitologia Veterinária. 2016;25(4):484-91.
770. Fotso AF, Mediannikov O, Nappez C, Azza S, Raoult D, Drancourt M. Monoclonal antibodies for the diagnosis of borrelia crocidurae. American Journal of Tropical Medicine and Hygiene. 2016;94(1):61-7.
771. Cross R, Ling C, Day NPJ, McGready R, Paris DH. Revisiting doxycycline in pregnancy and early childhood – time to rebuild its reputation? Expert Opinion on Drug Safety. 2016;15(3):367-82.
772. Boden K, Lobenstein S, Hermann B, Margos G, Fingerle V. Borrelia miyamotoi–Associated Neuroborreliosis in Immunocompromised Person. Emerging Infectious Diseases. 2016;22(9):1617-20.
773. Baneth G, Nachum-Biala Y, Halperin T, Hershko Y, Kleinerman G, Anug Y, et al. Borrelia persica infection in dogs and cats: clinical manifestations, clinicopathological findings and genetic characterization. Parasites & Vectors. 2016;9(1).

774. Todd SR, Dahlgren FS, Traeger MS, Beltrán-Aguilar ED, Marianos DW, Hamilton C, et al. No Visible Dental Staining in Children Treated with Doxycycline for Suspected Rocky Mountain Spotted Fever. The Journal of Pediatrics. 2015;166(5):1246-51.
775. CDC. Tick-Borne Relapsing Fever (TBRF). 2015.
776. Bermudez S, Miranda RJ, Cleghorn J, Venzal JM. Ornithodoros (Alectorobius) puertoricensis (Ixodida: Argasidae) parasitizing exotic reptile pets in Panama. FAVE Sección Ciencias Veterinarias. 2015;14(1/2):1-5.
777. Wodecka B, Rymaszewska A, Skotarczak B. Host and pathogen DNA identification in blood meals of nymphal Ixodes ricinus ticks from forest parks and rural forests of Poland. Experimental and Applied Acarology. 2014;62(4):543-55.
778. Mediannikov O, Socolovschi C, Bassene H, Diatta G, Ratmanov P, Fenollar F, et al. Borrelia crocidurae Infection in Acutely Febrile Patients, Senegal. Emerging Infectious Diseases. 2014;20(8):1335-8.
779. Killmaster LF, Loftis AD, Zemtsova GE, Levin ML. Detection of Bacterial Agents inAmblyomma americanum(Acari: Ixodidae) From Georgia, USA, and the Use of a Multiplex Assay to DifferentiateEhrlichia chaffeensisandEhrlichia ewingii. Journal of Medical Entomology. 2014;51(4):868-72.
780. Kelly AL, Raffel SJ, Fischer RJ, Bellinghausen M, Stevenson C, Schwan TG. First isolation of the relapsing fever spirochete, Borrelia hermsii, from a domestic dog. Ticks and tick-borne diseases. 2014;5(2):95-9.
781. Burri C, Schumann O, Schumann C, Gern L. Are Apodemus spp. mice and Myodes glareolus reservoirs for Borrelia miyamotoi, Candidatus Neoehrlichia mikurensis, Rickettsia helvetica, R. monacensis and Anaplasma phagocytophilum? Ticks and Tick-borne Diseases. 2014;5(3):245-51.
782. Wilhelmsson P, Lindblom P, Fryland L, Ernerudh J, Forsberg P, Lindgren P-E. Prevalence, Diversity, and Load of Borrelia species in Ticks That Have Fed on Humans in Regions of Sweden and Åland Islands, Finland with Different Lyme Borreliosis Incidences. PLoS ONE. 2013;8(11):e81433.
783. Taylor KR, Takano A, Konnai S, Shimozuru M, Kawabata H, Tsubota T. Borrelia miyamotoiInfections among Wild Rodents Show Age and Month Independence and Correlation withIxodes persulcatusLarval Attachment in Hokkaido, Japan. Vector-Borne and Zoonotic Diseases. 2013;13(2):92-7.
784. Rollend L, Fish D, Childs JE. Transovarial transmission of Borrelia spirochetes by Ixodes scapularis: A summary of the literature and recent observations. Ticks and Tick-borne Diseases. 2013;4(1-2):46-51.
785. Reck J, Marks FS, Guimarães JA, Termignoni C, Martins JR. Epidemiology of Ornithodoros brasiliensis (mouro tick) in the southern Brazilian highlands and the description of human and animal retrospective cases of tick parasitism. Ticks and Tick-borne Diseases. 2013;4(1-2):101-9.
786. Lopez JE, Wilder HK, Boyle W, Drumheller LB, Thornton JA, Willeford B, et al. Sequence Analysis and Serological Responses against Borrelia turicatae BipA, a Putative Species-Specific Antigen. PLoS Neglected Tropical Diseases. 2013;7(9):e2454.
787. Jensenius M, Schlagenhauf P, Loutan L, Parola P, Schwartz E, Leder K, et al. Acute and Potentially Life-Threatening Tropical Diseases in Western Travelers—A GeoSentinel Multicenter Study, 1996–2011. The American Journal of Tropical Medicine and Hygiene. 2013;88(2):397-404.

788. Gugliotta JL, Goethert HK, Berardi VP, Telford SR. Meningoencephalitis from Borrelia miyamotoi in an Immunocompromised Patient. New England Journal of Medicine. 2013;368(3):240-5.
789. Goutier S, Ferquel E, Pinel C, Bosseray A, Hoen B, Couetdic G, et al. Borrelia crocidurae Meningoencephalitis, West Africa. Emerging Infectious Diseases. 2013;19(2):301-4.
790. Chowdri HR, Gugliotta JL, Berardi VP, Goethert HK, Molloy PJ, Sterling SL, et al. Borrelia miyamotoiInfection Presenting as Human Granulocytic Anaplasmosis. Annals of Internal Medicine. 2013;159(1):21.
791. Socolovschi C, Kernif T, Raoult D, Parola P. Borrelia,Rickettsia, andEhrlichiaSpecies in Bat Ticks, France, 2010. Emerging Infectious Diseases. 2012;18(12):1966-75.
792. Fryxell RTT, Steelman CD, Szalanski AL, Kvamme KL, Billingsley PM, Williamson PC. Survey of Borreliae in ticks, canines, and white-tailed deer from Arkansas, U.S.A. Parasites & Vectors. 2012;5(1):139.
793. CDC. Tickborne Relapsing Fever in a Mother and Newborn Child — Colorado, 2011. MMWR. 2012.
794. Reck J, Soares JF, Termignoni C, Labruna MB, Martins JR. Tick toxicosis in a dog bitten by Ornithodoros brasiliensis. Veterinary Clinical Pathology. 2011;40(3):356-60.
795. Martins, Jr., Doyle R, Barros-Battesti D, Onofrio V, Guglielmone A. Occurrence of Ornithodoros brasiliensis Aragão (Acari: Argasidae) in São Francisco de Paula, RS, Southern Brazil. Neotropical Entomology. 2011;40(1):143-4.
796. Guerrier G, Doherty T. Comparison of antibiotic regimens for treating louse-borne relapsing fever: A meta-analysis. Transactions of the Royal Society of Tropical Medicine and Hygiene. 2011;105(9):483-90.
797. Colin De Verdiere N. Tickborne Relapsing Fever Caused by Borrelia persica, Uzbekistan and Tajikistan. Emerging Infectious Diseases. 2011;17(7):1325-7.
798. Fischer RJ, Johnson TL, Raffel SJ, Schwan TG. Identical Strains ofBorrelia hermsiiin Mammal and Bird. Emerging Infectious Diseases. 2009;15(12):2064-6.
799. Masters EJ, Grigery CN, Masters RW. STARI, or Masters Disease: Lone Star Tick–Vectored Lyme-like Illness. Infectious Disease Clinics of North America. 2008;22(2):361-76.
800. Lescot M, Audic S, Robert C, Nguyen TT, Blanc G, Cutler SJ, et al. The genome of Borrelia recurrentis, the agent of deadly louse-borne relapsing fever, is a degraded subset of tick-borne Borrelia duttonii. PLoS Genetics. 2008;4(9).
801. Hulínská D, Votýpka J, Kříž B, Holínková N, Nováková J, Hulínský V. Phenotypic and genotypic analysis ofBorrelia spp. isolated fromIxodes ricinus ticks by using electrophoretic chips and real-time polymerase chain reaction. Folia Microbiologica. 2007;52(4):315-24.
802. Stackebrandt E, Ebers J. Taxonomic parameters revisited: tarnished gold standards. MICROBIOLOGY TODAY. 2006;33(4):152-5.
803. Reeves WK, Loftis AD, Sanders F, Spinks MD, Wills W, Denison AM, et al. Borrelia, Coxiella, and Rickettsia in Carios capensis (Acari: Argasidae) from a brown pelican (Pelecanus occidentalis) rookery in South Carolina, USA. Experimental and Applied Acarology. 2006;39(3-4):321-9.
804. Guglielmone AA, Beati L, Barros-Battesti DM, Labruna MB, Nava S, Venzal JM, et al. Ticks (Ixodidae) on humans in South America. Experimental and Applied Acarology. 2006;40(2):83-100.
805. Loftis AD, Gill JS, Schriefer ME, Levin ML, Eremeeva ME, Gilchrist MJR, et al. Detection ofRickettsia,Borrelia, andBartonellainCarios kelleyi(Acari: Argasidae). Journal of Medical Entomology. 2005;42(3):473-80.

806. Schwartz RS. Paul Ehrlich's Magic Bullets. New England Journal of Medicine. 2004;350(11):1079-80.
807. Bell-Sakyi L, Koney EBM, Dogbey O, Walker AR. Incidence and prevalence of tick-borne haemoparasites in domestic ruminants in Ghana. Veterinary Parasitology. 2004;124(1-2):25-42.
808. Majidpour A. A case of Borrelia meningitis. Arch of Iranian Med. 2003;6.
809. Cook GC, Zumla A. Manson's Tropical Diseases. 21 ed: Saunders; 2003 2003. p. 1153-61 p.
810. Vatandoost H, Ghaderi A, Javadian E, Nia AH, Rassi Y, Piazak N, et al. Distribution of Soft Ticks and Their Infection with Borrelia in Hamadan Province, Iran. Iran J Public Health. 2002;32.
811. Hovette P, Aubron C, Perrier-Gros-Claude JD, Schieman R, N'Dir MC, Camara P. [Value of Quantitative Buffy Coat (QBC) in borreliasis-malaria co-infection]. Med Trop (Mars). 2001;61(2):196-7.
812. Angela, Liveris D, Gary, Schwartz I, Marisa, Barbara. Borrelia lonestariInfection after a Bite by anAmblyomma americanumTick. The Journal of Infectious Diseases. 2001;183(12):1810-4.
813. Shamaei-Tousi A, Burns MJ, Benach JL, Furie MB, Gergel EI, Bergström S. The relapsing fever spirochaete, Borrelia crocidurae, activates human endothelial cells and promotes the transendothelial migration of neutrophils. Cell Microbiol. 2000;2(6):591-9.
814. Fukunaga M, Okada K, Nakao M, Konishi T, Sato Y. Phylogenetic Analysis of Borrelia Species Based on Flagellin Gene Sequences and Its Application for Molecular Typing of Lyme Disease Borreliae. International Journal of Systematic Bacteriology. 1996;46(4):898-905.
815. Barbour AG, Maupin GO, Teltow GJ, Carter CJ, Piesman J. Identification of an Uncultivable Borrelia Species in the Hard Tick Amblyomma americanum: Possible Agent of a Lyme Disease-like Illness. Journal of Infectious Diseases. 1996;173(2):403-9.
816. Armstrong PM, Rich SM, Smith RD, Hartl DL, Spielman A, Telford SR, 3rd. A new Borrelia infecting Lone Star ticks. Lancet. 1996;347(8993):67-8.
817. Murray RG, Stackebrandt E. Taxonomic note: implementation of the provisional status Candidatus for incompletely described procaryotes. Int J Syst Bacteriol. 1995;45(1):186-7.
818. Fukunaga M, Takahashi Y, Tsuruta Y, Matsushita O, Ralph D, McClelland M, et al. Genetic and Phenotypic Analysis of Borrelia miyamotoi sp. nov., Isolated from the Ixodid Tick Ixodes persulcatus, the Vector for Lyme Disease in Japan. International Journal of Systematic Bacteriology. 1995;45(4):804-10.
819. Ciceroni L, Bartoloni A, Guglielmetti P, Paradisi F, Barahona HG, Roselli M, et al. Prevalence of antibodies to Borrelia burgdorferi, Borrelia parkeri and Borrelia turicatae in human settlements of the Cordillera Province, Bolivia. J Trop Med Hyg. 1994;97(1):13-7.
820. Adeyeye OA, Butler JF. Population Structure and Seasonal Intra-Burrow Movement of Ornithodoros turicata (Acari: Argasidae) in Gopher Tortoise Burrows. Journal of Medical Entomology. 1989;26(4):279-83.
821. Uilenberg G, Hinaidy HK, Perié NM, Feenstra T. Borreliainfections of ruminants in Europe. Veterinary Quarterly. 1988;10(1):63-7.
822. Magnarelli LA, Anderson JF, Johnson RC. Cross-reactivity in serological tests for Lyme disease and other spirochetal infections. Journal of Infectious Diseases. 1987;156(1):183-8.
823. Johnson RC, Burgdorfer W, Lane RS, Barbour AG, Hayes SF, Hyde FW. Borrelia coriaceae sp. nov.: Putative Agent of Epizootic Bovine Abortion. International Journal of Systematic Bacteriology. 1987;37(1):72-4.

824. Felsenfeld O. Borrelia: strains, vectors, human and animal borreliosis1971. xii + 180 pp. p.
825. N.Piazak, Rashti MAS, Assmar M. A SURVEY OF PREVALENCE OF ORNITHODORUS TARTAKOVSKYI AND ITS INFECTION RATE WITH BORRELIA LATYCHEVI IN SARAKHS COUNTY,KHORASSAN PROVINCE. Iranian Journal of Public Health. 1970;29(1-4).
826. Southern PMJ, Sanford JP. RELAPSING FEVER: A Clinical and Microbiological Review. Medicine. 1969;48(2).
827. SOUTHERN PMJ, SANFORD JP. RELAPSING FEVER: A Clinical and Microbiological Review. Medicine. 1969;48(2):129-50.
828. De Vera Andrey R, Maldonado Sampedro M. Study on the use of terramycin in the treatment of Spanish recurrent fever. Revista de sanidad e higiene pública. 1956;30(9-10):598-647.
829. Davis G. The endemic relapsing fevers. Springfield (IL): Charles C Thomas; 1955. 552–65 p.
830. Heisch RB. On a spirochaete isolated from Ornithodoros graingeri. Parasitology. 1953;43(1-2):133-5.
831. EDW G. An Epidemic of Relapsing Fever in Edinburgh In 1843. Edinburgh Medical Journal. 1943;50(11):681-6.
832. Francıs E. Longevity of the Tick Ornithodoros turicata and of Spirochaeta recurrentis within This Tick. Public Health Reports (1896-1970). 1938;53(51):2220.
833. Weller B, Graham GM. RELAPSING FEVER IN CENTRAL TEXAS. Journal of the American Medical Association. 1930;95(24):1834.
834. Dunn LH. Studies on the South American Tick, Ornithodoros venezuelensis Brumpt, in Colombia. Its Prevalence, Distribution, and Importance as an Intermediate Host of Relapsing Fever. The Journal of Parasitology. 1927;13(4):249.
835. Ehrlich P, Hafa S. Die experimentelle Chemotherapie der Spirillosen. Springer-Verlag Berlin, Heidelberg. 1910;VIII:178.
836. W. H. On some of the characters which distinguish the fever at present epidemic from typhus fever. dinburgh Med Surg J 1844;61:201-25.
837. D. C. Notice of a febrile disorder which has prevailed at Edinburgh during the summer of 1843. Edinburgh Med Surg J. 1843;60:410–8.
838. Hoornstra D, Sprong H, Hovius JW. The other tick-bite diseases. Nederlands Tijdschrift voor Geneeskunde. 2020;164(28).
839. Budzáková M, Trna J. Gastrointestinal and hepatic symptoms of tickborne diseases. Vnitrni Lekarstvi. 2020;66(4):232-5.
840. Rudakova SA, Pen'Evskaya NA, Rudakov NV, Pakskina ND, Savel'Ev DA, Blokh AI. Intensity and trends in development of epidemic process of Ixodes tick-borne borrelioses in the Russian Federation in 2002-2018 and forecast for 2019. Problemy Osobo Opasnykh Infektsii. 2019;2019(2):22-9.
841. Tylewska-Wierzbanowska S, Fiecek B, Chmielewski T. Relapsing fevers. Postepy Mikrobiologii. 2018;57(1):41-6.
842. Wiersinga W, van der Meer J, Prins J. Infectieziekten. Leerboek interne geneeskunde. 2017:127-213.
843. Tsubota T. Host-Vector-Pathogen Interactions of Tick-Borne Diseases in Hokkaido Wildlife. Japanese Journal of Zoo and Wildlife Medicine. 2016;21(3):47-51.
844. Hytönen J, Khawaja T, Grönroos JO, Jalava A, Meri S, Oksi J. Relapsing fever. Duodecim; laaketieteellinen aikakauskirja. 2016;132(21):1952-6.

845. Bagautdinova LI, Platonov AE, Sarksyan DS, Stukolova OV, Shipulin GA, Maleev VV, et al. [Follow-up of patients with Ixodes tick-borne borrelioses caused by Borrelia miyamotoi or Borrelia burgdorferi sensu lato]. Ter Arkh. 2016;88(11):43-54.
846. Kutsuna S, Kawabata H, Shiga N, Ujiie M, Takeshita N, Hayakawa K, et al. [Second Japanese case of relapsing fever]. [Japanese]. Kansenshogaku zasshi. 2014;The Journal of the Japanese Association for Infectious Diseases. 88(5):713-4.
847. Sarksyan DS, Platonov AE, Karan LS, Malinin IE, Khalitova LI, Shakhov VI, et al. [Clinical presentation of "new" tick-borne borreliosis caused by Borrelia miyamotoi]. Terapevticheskiĭ arkhiv. 2012;84(11):34-41.
848. Jaenson TGT. Ny fästingöverförd sjukdom. Lakartidningen. 2011;108(40).
849. Platonov AE, Maleev VV, Karan LS. [Relapsing borrelioses fevers: forgotten and new ones]. Terapevticheskiĭ arkhiv. 2010;82(11):74-80.
850. Fomenko NV, Livanova NN, Borgoyakov VY, Kozlova IV, Shulaykina IV, Pukhovskaya NM, et al. Detection of borrelia miyamotoi in ticks ixodes persulcatus from Russia. Parazitologiya. 2010;44(3):201-11.
851. van den Broek P, Koten JW. Infectieziekten. Boekblok Arbeid en Belastbaarheid. 2009:193-230.
852. Masuda G. Relapsing fever. Nippon rinsho Japanese journal of clinical medicine. 2003;61 Suppl 2:547-50.
853. Alypova II, Korenberg EI, Vorob'eva NN. Risk of human infection with Ixodes tick-borne relapsing fever in different regional subzones of the Perm region. Meditsinskaia parazitologiia i parazitarnye bolezni. 2002(1):37-40.
854. Pervakova GV, Chastikov OY. Epidemiological analysis of the incidence of tick-borne relapsing fever in the Kirovo-Chepetsky District, Kirov Region in 1996-1998. Meditsinskaya Parazitologiya i Parazitarnye Bolezni. 2000(3):16-8.
855. Ito A. Relapsing fever. Ryōikibetsu shōkōgun shirīzu. 1999(24 Pt 2):297.
856. Vasil'eva IS, Gutova VP, Dremova VP, Ershova AS, Labzin VV. The activity of cyfluthrin and cypermethrin-based drugs against tick-borne relapsing fever carrier. Meditsinskaya Parazitologiya i Parazitarnye Bolezni. 1995;0(2):8-12.
857. Litvinjenko S. How the epidemics of typhus and relapsing fever were stopped in Serbia in 1915 year. Srpski arhiv za celokupno lekarstvo. 1995;123(11-12):328-30.
858. Verbenko EV, Kryazheva SS. Atrophic acrodermatitis: A manifestation of tick-borne relapsing fever. Vestnik Dermatologii i Venerologii. 1994;0(1):28-9.
859. Vasil'eva IS, Gutova VP, Dremova VP, Abidov ZI, Labzin VV. The sensitivity of the vector of tick-borne relapsing fever to pyrethroids. Meditsinskaia parazitologiia i parazitarnye bolezni. 1994(1):47-52.
860. Vasil'eva IS, Gutova VP. Effects of communal insecticide aerosols and crayons on the vector of tick-borne relapsing fever. Meditsinskaya Parazitologiya i Parazitarnye Bolezni. 1993;0(4):49-51.
861. Vasil'eva IS, Gutova VP. Comparative assessment of the sensitivity of tick-borne relapsing fever carrier to pesticides with the use of standard test paper. Meditsinskaya Parazitologiya i Parazitarnye Bolezni. 1993;0(1):31-2.
862. Abidov ZI, Vasil'eva IS, Rakhimov NR, Gutova VP, Parpiev AM. The incidence of tick-borne relapsing fever in the town of Namangan. Meditsinskaya Parazitologiya i Parazitarnye Bolezni. 1993;0(1):32-5.
863. Abidov ZI, Vasil'eva IS, Rakhimov NR, Gutova VP, Parpiev AM. Tick-borne relapsing fever morbidity in Namangan. Meditsinskaia parazitologiia i parazitarnye bolezni. 1993(1):32-5.

864. Vasil'eva IS, Ershova AS, Shoismatulloev B, Topchin Iu A, Kiebekov I. [Tick-borne relapsing fever morbidity in the western Pamirs]. Med Parazitol (Mosk). 1991(3):22-4.
865. Vasilyeva IS, Ershova AS, Vilisov GM, Khizhinsky PG, Shoismatulloev Sh B, Ikbolov A, et al. Present-day situation in the foci of tick-borne relapsing fever in the Western Pamirs. Meditsinskaya Parazitologiya i Parazitarnye Bolezni. 1990(6):31-4.
866. Vasilyeva IS, Byzova Yu B, Ershova AS. Effect of causative agent of relapsing fever on respiration of its vector Ornithodoros papillipes (Birula, 1895) (Ixodoidea, Argasidae). Izevestiya Akademii Nauk SSSR - Seriya Biologicheskaya. 1990(5):717-27.
867. Vandemoortele E, Avonds W. [African tick fever; fever, exanthema and skin ulcer]. Ned Tijdschr Geneeskd. 1989;133(46):2305-6.
868. Vasil'eva IS, Ershova AS, Mansurov AA, Khozhaev AK, Norbadalov TT. Current status of foci of tick-borne relapsing fever in Kashka-Darya Province. Meditsinskaia parazitologiia i parazitarnye bolezni. 1987(6):47-51.
869. Méndez E, Chaniotis B. [Outline of principal diseases transmitted by ticks in Panama]. Rev Med Panama. 1987;12(3):217-23.
870. Pchelkin AP, Dzhurakhodzhaev AK. The modern clinical course of tick-borne relapsing fever. Meditsinskaia parazitologiia i parazitarnye bolezni. 1986(1):51-5.
871. Ustimenko N, Musatova AI, Makarova LS, Cherneva LI, Ofitserova NF. [Tick-borne relapsing fever in Fergana Province, Uzbekistan]. Med Parazitol (Mosk). 1985(5):85-6.
872. Megiddo D. A case survey of acute relapsing fever in Israel. Harefuah. 1984;107(5-6):142-5.
873. Sirnes KE. Relapsing fever. Tidsskrift for den Norske Laegeforening. 1982;102(32):1710-1+21.
874. Kriuchechnikov VN, Korenberg EI, Shcherbakov SV. Attempt at detecting strain differences in the causative agent of tick-borne relapsing fever. Meditsinskaia parazitologiia i parazitarnye bolezni. 1982;51(4):74-9.
875. Ershova AS, Vasil'eva IS. Infestation of village populations of Ornithodoros papillipes Bir. with the causative agent of tick-borne relapsing fever in Uzbekistan. Meditsinskaia parazitologiia i parazitarnye bolezni. 1982;51(3):23-8.
876. Gromov OA, Khudobets AA. Interrelations of Borrelia and Ornithodorus in connection with the maintenance of natural foci of tick-borne relapsing fever. Trudy Instituta imeni Pastera. 1980;55:91-4.
877. Dagan R, Gradus D, Milman G, Hammer R, Kaplan H. Relapsing fever in children in the northern Negev. [Hebrew]. Harefuah. 1979;96(5):234-5.
878. Lerman Y, Koufman Z, Egoz N. The clinical picture of relapsing fever in the Israel Defense Forces, 1966--76. Harefuah. 1978;95(11):365-7.
879. Egoz N, Lerman Y, Koufman Z. The epidemiology of relapsing fever in the Israel Defense Forces, 1966--76. Harefuah. 1978;95(11):367-70.
880. van der Heide RM. A case of recurrent fever imported into the Netherlands. Nederlands tijdschrift voor geneeskunde. 1971;115(14):607-8.
881. Ovnanian KO. Electron microscopic study of the causative agents of tick relapsing fever. Meditsinskaia parazitologiia i parazitarnye bolezni. 1969;38(2):223-6.
882. Slesarenko AS, Slesarenko VV. [On the classification of the causative agent of "Ukrainian" tick relapsing eer]. Med Parazitol (Mosk). 1968;37(4):488-90.
883. Sato Y, Washio M, Omata N, Majima T, Hirasawa Y, Hayakawa H, et al. A CASE COMPLAINING OF RELAPSING FEVER AND SUBCUTANEOUS NODULES. Newsletter International College of Dentists India Section. 1964;78:284-92.

884. AN ISOLATED CASE OF RELAPSING FEVER ACCOMPANIED BY DYS- AND PARAPROTEINEMIA. Przegląd epidemiologiczny. 1964;18:447-52.
885. Sofiev MS, Shtyreva LV, Shcheulov AP. APROPOS OF THE DEVELOPMENT OF THE SPIROCHETE OF TICK RECURRENT FEVER. Meditsinskaia parazitologiia i parazitarnye bolezni. 1963;32:655-9.
886. VIa KM, Nasibulina FK. [Data on the study of the epidemiology of tick recurrent fever in the Bostandykski region]. Izv Seriia Fiziol Meditsiny Qazaq SSR Ghylum Akad. 1960;2:32-40.
887. Garcia Diaz A. "Ornithodoros" as reservoirs and vectors of relapsing fever. Revista de sanidad e higiene pública. 1960;34:325-72.
888. Pospelov-Shtrom MV. [Tick-borne spirochetosis and the public health problem in the USSR]. Med Parazitol (Mosk). 1959;28(3):335-42.
889. Netrebko ID. Observations on foci of tick-spirochetosis in Kherson and adjacent regions of the Ukrainian Republic. Meditsinskaia parazitologiia i parazitarnye bolezni. 1959;28:571-5.
890. Il'Ina NA. Neurological complications of relapsing fever. Klinicheskaia meditsina. 1954;32(7):66-9.
891. Munoz Cosin F. Vector and epidemiology of Hispano-African recurrent fever. La Medicina colonial. 1953;22(5):487-505.
892. De Dias AC, De Faia MM. Hispano-African relapsing fever; epidemiological and laboratory notes. Anais do Instituto de Medicina Tropical. 1953;10(3 :1):641-3.
893. Cambournac FJ, Soares AC, De Roque RA, Res JF, Queiroz JS. Relapsing fever in Portugal. Anais do Instituto de Medicina Tropical. 1953;10(3 :1):645-54.
894. Miljkovic AM. Clinical and laboratory studies in epidemics of recurrent fever. Higijena; časopis za higijenu, mikrobiologiju, epidemiologiju i sanitarnu tehniku. 1950;2(4):321-38.
895. Lipinski W. Studies on recurrent fever. Polski tygodnik lekarski. 1950;5(42):1465-6.
896. Kapnik GM, Brenner EV. Pathogenesis of bradycardia and hypotonia in relapsing fever. Terapevticheskiĭ arkhiv. 1950;22(4):73-9.
897. Iarygin NE. Prodromal period in European recurrent fever. Klinicheskaia meditsina. 1950;3:83-5.
898. Ehrenkrantz DI. [Neurologic symptoms in relapsing fever]. Nevropatol Psikhiatriia. 1950;19(2):45-7.
899. Dujardin J. [Some cases of recurrent eruptive fever]. Maroc Med. 1950;29(302):641.
900. Krakowski L, Edelstein A. A brief survey of 25 cases of tick-borne relapsing fever. Harefuah. 1949;36(9):101-3.
901. Stuart G. [Recurrent fever in North Africa and Europe 1943-1945]. Rev Sanid. 1946;20:164-76.
902. De Cisneros JM, Garcia Gatturno J. [Avian spirochetosis in Spain]. Rev Sanid. 1946;20(9):887-95.
903. Anonymous. [Recurrent fever in Spain and Spanish Morocco]. Rev Sanid. 1946;20:177-88.
904. Pavlovsky EN, Kuzmina LA. On the possibility of the transmission of the spirochetes of tick recurrent fever by Ornithodorus lahorensis to monkeys and man. Meditsinskaia parazitologiia i parazitarnye bolezni. 1945;14(3):66-70.
905. Pavlovsky EN. [Natural endemicity of tick recurrent fever in the Turkoman Socialist Soviet Republic]. Meditsinskaia parazitologiia i parazitarnye bolezni. 1945;14(3):56-9.
906. Maruashvili GM. [On tick-borne relapsing fever]. Meditsinskaia parazitologiia i parazitarnye bolezni. 1945;14(1):24-7.

907. Yuan Q, Llanos-Soto SG, Gangloff-Kaufmann JL, Lampman JM, Frye MJ, Benedict MC, et al. Active surveillance of pathogens from ticks collected in New York State suburban parks and schoolyards. Zoonoses and Public Health. 2020;67(6):684-96.
908. Stuen S. Haemoparasites—Challenging and Wasting Infections in Small Ruminants: A Review. Animals (Basel). 2020;10(11).
909. Seo MG, Kwon OD, Kwak D. Molecular Identification of Borrelia afzelii from Ticks Parasitizing Domestic and Wild Animals in South Korea. Microorganisms. 2020;8(5).
910. Requena-Méndez A, Bisoffi Z, Vives-Corrons JL, Gascon J, Plasència A. European expert network on rare communicable diseases and other rare diseases linked to mobility and globalisation focused on health care provision (EURaDMoG): a feasibility study. Orphanet J Rare Dis. 2020;15.
911. Oumarou Hama H, Barbieri R, Guirou J, Chenal T, Mayer A, Ardagna Y, et al. An outbreak of relapsing fever unmasked by microbial paleoserology, 16th century, France. American Journal of Physical Anthropology. 2020.
912. Munoz-Leal S, Ramirez DG, Luz HR, Faccini JLH, Labruna MB. "Candidatus Borrelia ibitipoquensis," a Borrelia valaisiana-Related Genospecies Characterized from Ixodes paranaensis in Brazil. Microbial Ecology. 2020;80(3):682-9.
913. Masatani T, Hayashi K, Morikawa M, Ozawa M, Kojima I, Okajima M, et al. Molecular detection of tick-borne protozoan parasites in sika deer (Cervus nippon) from western regions of Japan. Parasitology International. 2020;79.
914. Ly TDA, Louni M, Hoang VT, Dao TL, Badiaga S, Brouqui P, et al. Epidemiological serosurvey of vector-borne and zoonotic pathogens among homeless people living in shelters in Marseille: cross-sectional one-day surveys (2005–2015). European Journal of Clinical Microbiology and Infectious Diseases. 2020;39(9):1663-72.
915. Lowery K, Rosen T. Probable African Tick Bite Fever in the United States. Yale J Biol Med. 2020;93(1):49-54.
916. Liu Y, Zong Z. Prolonged intermittent fever and massive splenomegaly in a miner working in the tropical jungle, China. PLoS Negl Trop Dis. 2020;14(7):e0008278.
917. Khoobdel M, Dehghan O, Bakhshi H, Moradi M. Control and management of vector-borne diseases in disaster conditions. Journal of Military Medicine. 2020;22(8):778-98.
918. Keita ML, Medkour H, Sambou M, Dahmana H, Mediannikov O. Tabanids as possible pathogen vectors in Senegal (West Africa). Parasit Vectors. 2020;13.
919. Jado I, Escudero R, Espigares B, Lara E, Rodriguez-Vargas M, Garcia-Amil C, et al. Rapid and Highly Sensitive DNA Flow Technology Platform to Detect Tick-Borne Bacterial Pathogens in Clinical Samples. Vector-Borne and Zoonotic Diseases. 2020;20(2):107-16.
920. Head JR, Bumburidi Y, Mirzabekova G, Rakhimov K, Dzhumankulov M, Salyer SJ, et al. Risk Factors for and Seroprevalence of Tickborne Zoonotic Diseases among Livestock Owners, Kazakhstan. Emerg Infect Dis. 2020;26(1):70-80.
921. Fudge JM, Boyanowski B, Page B, Liu S, Rogovskyy AS. Serological prevalence of six vector-borne pathogens in dogs presented for elective ovariohysterectomy or castration in the South central region of Texas. BMC Vet Res. 2020;16.
922. Crump A, Tanimoto T. Severe Fever with Thrombocytopenia Syndrome: Japan under Threat from Life-threatening Emerging Tick-borne Disease. Jma j. 2020;3(4):295-302.
923. Colunga-Salas P, Sánchez-Montes S, Ochoa-Ochoa LM, Grostieta E, Becker I. Molecular detection of the reptile-associated Borrelia group in Amblyomma dissimile, Mexico. Medical and Veterinary Entomology. 2020.

924. Colella V, Nguyen VL, Tan DY, Lu N, Fang F, Zhijuan Y, et al. Zoonotic Vectorborne Pathogens and Ectoparasites of Dogs and Cats in Eastern and Southeast Asia. Emerg Infect Dis. 2020;26(6):1221-33.
925. Thapa S, Zhang Y, Allen MS. Bacterial microbiomes of Ixodes scapularis ticks collected from Massachusetts and Texas, USA. BMC Microbiol. 2019;19.
926. Shetty AK. Infectious Diseases among Refugee Children. Children (Basel). 2019;6(12).
927. Sazmand A, Harl J, Eigner B, Hodžić A, Beck R, Hekmatimoghaddam S, et al. Vector-borne bacteria in blood of camels in Iran: New data and literature review. Comp Immunol Microbiol Infect Dis. 2019;65:48-53.
928. Rose I, Yoshimizu MH, Bonilla DL, Fedorova N, Lane RS, Padgett KA. Phylogeography of Borrelia spirochetes in Ixodes pacificus and Ixodes spinipalpis ticks highlights differential acarological risk of tick-borne disease transmission in northern versus southern California. PLoS One. 2019;14(4).
929. Read JS. Tickborne Diseases in Children in the United States. Pediatrics in Review. 2019;40(8):381-97.
930. Jiménez-Morillas F, Gil-Mosquera M, García-Lamberechts EJ. Fever in travellers returning from the tropics☆. Med Clin (Engl Ed). 2019;153(5):205-12.
931. Gautret P, Parola P, Wilson ME. Fever in Returned Travelers. Travel Medicine. 2019:495-504.
932. Dehhaghi M, Kazemi Shariat Panahi H, Holmes EC, Hudson BJ, Schloeffel R, Guillemin GJ. Human Tick-Borne Diseases in Australia. Front Cell Infect Microbiol. 2019;9.
933. Dall'Agnol B, Schott D, Padilha T, Antunes P, Souza UA, Webster A, et al. Clinical Findings Associated with Ornithodoros brasiliensis Tick Parasitism in Travelers, Southern Brazil. Wilderness and Environmental Medicine. 2019;30(4):437-40.
934. Antony S. Mosquito and tick-borne illnesses in the United States. Guidelines for the recognition and empiric treatment of zoonotic diseases in the wilderness. Infectious Disorders - Drug Targets. 2019;19(3):244-57.
935. Alghamdi SQ, Alagaili AN, Stekolnikov AA, Mc Garry JW, Darby AC, Makepeace BL. The vector biology of ectoparasites on rodent from the Asir region of Saudi Arabia. Transactions of the Royal Society of Tropical Medicine and Hygiene. 2019;113:S100.
936. Ware B, Ulrich R, Punjabi P. Relapsing fevers leading to neurologic collapse: A case of west nile encephalitis. Journal of General Internal Medicine. 2018;33(2):608-9.
937. Tomaso H, Otto P, Peters M, Süss J, Karger A, Schamoni H, et al. Francisella tularensis and other bacteria in hares and ticks in North Rhine-Westphalia (Germany). Ticks and Tick-borne Diseases. 2018;9(2):325-9.
938. Stanek G. Lyme borreliosis, ticks and Borrelia species. Wiener Klinische Wochenschrift. 2018;130(15-16):459-62.
939. Skotarczak B. The role of companion animals in the environmental circulation of tick-borne bacterial pathogens. Annals of Agricultural and Environmental Medicine. 2018;25(3):473-80.
940. Shuker O, Subran M, Hardie R, Ghitan M, Chapnick EK, Lin YS. An 85-Year-Old Man With Recurrent Fever and Multiple Splenic Infarcts. Infect Dis Clin Pract (Baltim Md). 2018;26(5):300-2.
941. Shah SZ, Jabbar B, Ahmed N, Rehman A, Nasir H, Nadeem S, et al. Epidemiology, Pathogenesis, and Control of a Tick-Borne Disease- Kyasanur Forest Disease: Current Status and Future Directions. Front Cell Infect Microbiol. 2018;8.
942. Scaggs Huang FA, Schlaudecker E. Fever in the Returning Traveler. Infect Dis Clin North Am. 2018;32(1):163-88.

943. Rosenberg R, Lindsey NP, Fischer M, Gregory CJ, Hinckley AF, Mead PS, et al. Vital Signs: Trends in Reported Vectorborne Disease Cases — United States and Territories, 2004–2016. MMWR Morb Mortal Wkly Rep. 2018;67(17):496-501.
944. Roczeń-Karczmarz M, Dudko P, Demkowska-Kutrzepa M, Meisner M, Studzińska M, Junkuszew A, et al. Comparison of the occurrence of tick-borne diseases in ticks collected from vegetation and animals in the same area. Medycyna Weterynaryjna. 2018;74(8):484-8.
945. Płusa T. Azithromycin in therapy of patients with borreliosis. International Review of Allergology and Clinical Immunology in Family Medicine. 2018;24(3):93-9.
946. Phuentshok Y, Dorji K, Zangpo T, Davidson SA, Takhampunya R, Tenzinla T, et al. Survey and Phylogenetic Analysis of Rodents and Important Rodent-Borne Zoonotic Pathogens in Gedu, Bhutan. Korean J Parasitol. 2018;56(5):521-5.
947. Park KH, Choi YJ, Kim J, Park HJ, Song D, Jang WJ. Reclassification of borrelia spp. Isolated in South Korea using multilocus sequence typing. Japanese Journal of Infectious Diseases. 2018;71(5):350-3.
948. Middelveen MJ, Cruz ID, Fesler MC, Stricker RB, Shah JS. Detection of tick-borne infection in morgellons disease patients by serological and molecular techniques. Clinical, Cosmetic and Investigational Dermatology. 2018;11:561-9.
949. Koirala KD, Chappuis F, Verdonck K, Rijal S, Boelaert M. Persistent febrile illnesses in Nepal: A systematic review. Indian J Med Res. 2018;148(4):385-95.
950. Kim TY, Kwak YS, Kim JY, Nam SH, Lee IY, Mduma S, et al. Prevalence of Tick-Borne Pathogens from Ticks Collected from Cattle and Wild Animals in Tanzania in 2012. Korean J Parasitol. 2018;56(3):305-8.
951. Khoo JJ, Ishak SN, Lim FS, Mohd-Taib FS, Khor CS, Loong SK, et al. Detection of a Borrelia sp. From Ixodes granulatus Ticks Collected From Rodents in Malaysia. Journal of medical entomology. 2018;55(6):1642-7.
952. Karasartova D, Gureser AS, Gokce T, Celebi B, Yapar D, Keskin A, et al. Bacterial and protozoal pathogens found in ticks collected from humans in Corum province of Turkey. PLoS Negl Trop Dis. 2018;12(4).
953. Guellil M, Kersten O, Namouchi A, Bauer EL, Derrick M, Jensen AO, et al. Genomic blueprint of a relapsing fever pathogen in 15th century Scandinavia. Proceedings of the National Academy of Sciences of the United States of America. 2018;115(41):10422-7.
954. Galay RL, Manalo AAL, Dolores SLD, Aguilar IPM, Sandalo KAC, Cruz KB, et al. Molecular detection of tick-borne pathogens in canine population and Rhipicephalus sanguineus (sensu lato) ticks from southern Metro Manila and Laguna, Philippines. Parasit Vectors. 2018;11.
955. Ackermann N, Marosevic D, Hörmansdorfer S, Eberle U, Rieder G, Treis B, et al. Screening for infectious diseases among newly arrived asylum seekers, Bavaria, Germany, 2015. Euro Surveill. 2018;23(10).
956. Verdugo C, Jiménez O, Hernández C, Álvarez P, Espinoza A, González-Acuña D. Infection with Borrelia chilensis in Ixodes stilesi ticks collected from Pudu puda deer. Ticks and Tick-borne Diseases. 2017;8(5):733-40.
957. VanBik D, Lee SH, Seo MG, Jeon BR, Goo YK, Park SJ, et al. Borrelia species detected in ticks feeding on wild Korean water deer (Hydropotes inermis) using molecular and genotypic analyses. Journal of Medical Entomology. 2017;54(5):1397-402.
958. Margos G, Fedorova N, Kleinjan JE, Hartberger C, Schwan TG, Sing A, et al. Borrelia lanei sp. nov. extends the diversity of Borrelia species in California. Int J Syst Evol Microbiol. 2017;67(10):3872-6.

959. Li Y, Wang J, Gao M, Fang L, Liu C, Lyu X, et al. Geographical Environment Factors and Risk Assessment of Tick-Borne Encephalitis in Hulunbuir, Northeastern China. Int J Environ Res Public Health. 2017;14(6).
960. Guven O, Satilmis D, Sonmez FT, Demir B, Erdogan Ö. TICK INFESTATION: A 200-PATIENTS' SERIES. Afr J Infect Dis. 2017;11(2):62-7.
961. Guadagnino G, Fasciana T, Caputo V, Scarlata F, Giammanco A, Di Carlo P. Revisiting anthropozoonoses in the mediterranean basin. A single-centre perspective. A southern italian experience. Pharmacologyonline. 2017;1(Special Issue):2-11.
962. Dedkov VG, Simonova EG, Beshlebova OV, Safonova MV, Stukolova OA, Verigina EV, et al. The burden of tick-borne diseases in the Altai region of Russia. Ticks and Tick-borne Diseases. 2017;8(5):787-94.
963. Bermúdez SE, Gottdenker N, Krishnvajhala A, Fox A, Wilder HK, González K, et al. Synanthropic mammals as potential hosts of tick-borne pathogens in Panama. PLoS ONE. 2017;12(1).
964. Basile RC, Yoshinari NH, Mantovani E, Bonoldi VN, Macoris Dda G, Queiroz-Neto A. Brazilian borreliosis with special emphasis on humans and horses. Braz J Microbiol. 2017;48(1):167-72.
965. Antinori S, Colombo V, Corbellino M. Relapsing fever in young refugees from East Africa. Critical Care. 2017;21(1).
966. Taragel'ová VR, Mahríková L, Selyemová D, Václav R, Derdáková M. Natural foci of Borrelia lusitaniae in a mountain region of Central Europe. Ticks and Tick-borne Diseases. 2016;7(2):350-6.
967. Korenberg EI, Sirotkin MB, Kovalevskii YV. A general scheme of circulation of ixodid tick-borne borrelioses pathogens in the natural foci of Eurasia. Entomological Review. 2016;96(4):484-99.
968. Kesztyüs B, Cornish D. Relapsing fever in refugee. International Journal of Medical Microbiology. 2016;306(8):134.
969. Guo XG, Dong WG, Men XY, Qian TJ, Wu D, Ren TG, et al. Species Abundance Distribution of Ectoparasites on Norway Rats (Rattus norvegicus) from a Localized Area in Southwest China. J Arthropod Borne Dis. 2016;10(2):192-200.
970. Faccini-Martínez ÁA, Pérez-Díaz CE, Botero-García CA, Benítez-Baracaldo FC, Rodríguez-López AE, Rodríguez-Morales AJ. Role of the blood smear in febrile returning travelers: Beyond malaria. Travel Medicine and Infectious Disease. 2016;14(5):515-6.
971. Diuk-Wasser MA, Vannier E, Krause PJ. Coinfection by Ixodes Tick-Borne Pathogens: Ecological, Epidemiological, and Clinical Consequences. Trends in Parasitology. 2016;32(1):30-42.
972. Costescu Strachinaru DI, Cambier J, Kandet-Yattara H, Konopnicki D. Relapsing fever in asylum seekers from Somalia arriving in Belgium in August 2015. Acta Clinica Belgica: International Journal of Clinical and Laboratory Medicine. 2016;71(5):353-5.
973. Brum WM, da Costa Pereira MAV, Vita GF, Ferreira I, Mello ER, Aurnheimer RCM, et al. Parasitism in migratory and resident wild birds of marambaia island, rio de janeiro state. Pesquisa Veterinaria Brasileira. 2016;36(11):1101-8.
974. Bessas A, Leulmi H, Bitam I, Zaidi S, Ait-Oudhia K, Raoult D, et al. Molecular evidence of vector-borne pathogens in dogs and cats and their ectoparasites in Algiers, Algeria. Comparative Immunology, Microbiology and Infectious Diseases. 2016;45:23-8.
975. Pavia C. Microbiological and clinical aspects of the pathogenic spirochetes. Practical Handbook of Microbiology, Third Edition2015. p. 353-70.

976. Nejezchlebová H, Bečárová K, Žákovská A. Tick-borne disease transmission risks between mother and fetus/child. Pediatrie pro Praxi. 2015;16(3):154-6.
977. Margos G, Binder K, Dzaferovic E, Hizo-Teufel C, Sing A, Wildner M, et al. PubMLST.org - The new home for the Borrelia MLSA database. Ticks and Tick-borne Diseases. 2015;6(6):869-71.
978. Korenberg EI, Kovalevskii YV, Gorelova NB, Nefedova VV. Comparative analysis of the roles of Ixodes persulcatus and I. trianguliceps ticks in natural foci of ixodid tick-borne borrelioses in the Middle Urals, Russia. Ticks and Tick-borne Diseases. 2015;6(3):316-21.
979. Vanstreels RET, Miranda FR, Ruoppolo V, Reis AOA, Costa ES, Pessôa ARL, et al. Investigation of blood parasites of pygoscelid penguins at the King George and Elephant Islands, South Shetlands Archipelago, Antarctica. Polar Biology. 2014;37(1):135-9.
980. Tveten AK. Exploring Diversity among Norwegian Borrelia Strains Originating from Ixodes ricinus Ticks. Int J Microbiol. 2014;2014.
981. Silaghi C, Pfister K, Overzier E. Molecular investigation for bacterial and protozoan tick-borne pathogens in wild boars (sus scrofa) from southern Germany. Vector-Borne and Zoonotic Diseases. 2014;14(5):371-3.
982. Nakayima J, Hayashida K, Nakao R, Ishii A, Ogawa H, Nakamura I, et al. Detection and characterization of zoonotic pathogens of free-ranging non-human primates from Zambia. Parasites and Vectors. 2014;7(1).
983. Chen Z, Liu Q, Liu JQ, Xu BL, Lv S, Xia S, et al. Tick-borne pathogens and associated co-infections in ticks collected from domestic animals in central China. Parasit Vectors. 2014;7:237.
984. Brett ME, Hinckley AF, Zielinski-Gutierrez EC, Mead PS. U.S. healthcare providers' experience with Lyme and other tick-borne diseases. Ticks Tick Borne Dis. 2014;5(4):404-8.
985. Berenji F, Marvi-Moghadam N, Naghibozakerin Meibodi P. A Retrospective Study of Ectoparasitosis in Patients Referred to Imam Reza Hospital of Mashhad, Iran. Biomed Res Int. 2014;2014.
986. Baranova E, Solovev P, Panfertsev E, Baranova A, Feduykina G, Kolombet L, et al. Rational design of antigens to improve the serodiagnosis of tick-borne borreliosis in central regions of Russia. Advances in Experimental Medicine and Biology2014. p. 9-21.
987. Sah RP, Wilson ME, Seningen J, Bhagra A. Relapsing fevers and lymphadenopathy in a young woman. BMJ Case Rep. 2013;2013.
988. Inci A, Yazar S, Tuncbilek AS, Canhilal R, Doganay M, Aydin L, et al. Vectors and vector-borne diseases in Turkey. Ankara Universitesi Veteriner Fakultesi Dergisi. 2013;60(4):281-96.
989. Hasle G. Transport of ixodid ticks and tick-borne pathogens by migratory birds. Front Cell Infect Microbiol. 2013;3.
990. Goltz L, Varela-Stokes A, Goddard J. Survey of adult Ixodes scapularis Say for disease agents in Mississippi. Journal of Vector Ecology. 2013;38(2):401-3.
991. Socolovschi C, Honnorat E, Consigny PH, Dougados J, Passeron A, Parola P, et al. Tick-borne relapsing fever with cutaneous eschar and radiculopathy, ethiopia. Journal of Travel Medicine. 2012;19(4):261-3.
992. Adjemian J, Weber IB, McQuiston J, Griffith KS, Mead PS, Nicholson W, et al. Zoonotic infections among employees from Great Smoky Mountains and Rocky Mountain National Parks, 2008-2009. Vector-Borne and Zoonotic Diseases. 2012;12(11):922-31.
993. Takano A, Nakao M, Masuzawa T, Takada N, Yano Y, Ishiguro F, et al. Multilocus Sequence Typing Implicates Rodents as the Main Reservoir Host of Human-Pathogenic Borrelia garinii in Japan ▿ †. J Clin Microbiol. 2011;49(5):2035-9.

994. Spolidorio MG, Labruna MB, Machado RZ, Moraes-Filho J, Zago AM, Donatele DM, et al. Survey for Tick-Borne Zoonoses in the State of Espirito Santo, Southeastern Brazil. Am J Trop Med Hyg. 2010;83(1):201-6.
995. Lubelczyk C, Cahill BK, Hanson T, Turmel J, Lacombe E, Rand PW, et al. Tick (Acari: Ixodidae) infestation at two rural, seasonal camps in Maine and Vermont. Journal of Parasitology. 2010;96(2):442-3.
996. Stanek G. Pandora's Box: Pathogens in Ixodes ricinus ticks in Central Europe. Wiener Klinische Wochenschrift. 2009;121(21-22):673-83.
997. Animut A, Mekonnen Y, Shimelis D, Ephraim E. Febrile illnesses of different etiology among outpatients in four health centers in northwestern Ethiopia. Japanese Journal of Infectious Diseases. 2009;62(2):107-10.
998. Borrelia recurrentis (Relapsing fever). Infectious Diseases in Obstetrics and Gynecology, Sixth Edition2008. p. 305-8.
999. Stowell CP, Gelfand JA, Shepard J, Kratz A, Stowell CP, Gelfand JA, et al. Case records of the Massachusetts General Hospital. Case 17-2007. A 25-year-old woman with relapsing fevers and recent onset of dyspnea. New England Journal of Medicine. 2007;356(22):2313-9.
1000. Hashimoto S, Kawado M, Murakami Y, Izumida M, Ohta A, Tada Y, et al. Epidemics of Vector-borne Diseases Observed in Infectious Disease Surveillance in Japan, 2000-2005. J Epidemiol. 2007;17(Suppl):S48-55.
1001. Barandika JF, Hurtado A, García-Esteban C, Gil H, Escudero R, Barral M, et al. Tick-Borne Zoonotic Bacteria in Wild and Domestic Small Mammals in Northern Spain ▿ . Appl Environ Microbiol. 2007;73(19):6166-71.
1002. Swanson SJ, Neitzel D, Reed KD, Belongia EA. Coinfections Acquired from Ixodes Ticks. Clin Microbiol Rev. 2006;19(4):708-27.
1003. Rodriguez-Framil M, Martinez-Rey C, Lopez-Rodriguez R, Alende-Sixto R. Relapsing fever and Staphylococcus aureus bacteriemia. [Spanish]. Enfermedades Infecciosas y Microbiologia Clinica. 2006;24(7):463-4.
1004. Younsi H, Sarih M, Jouda F, Godfroid E, Gern L, Bouattour A, et al. Characterization of Borrelia lusitaniae isolates collected in Tunisia and Morocco. Journal of Clinical Microbiology. 2005;43(4):1587-93.
1005. Reisinger EC, Fritzsche C, Tomaso H, Gasser R. Tick transmitted diseases. [German]. Medizinische Welt. 2005;56(1-2):25-9.
1006. Brouqui P, Stein A, Dupont HT, Gallian P, Badiaga S, Rolain JM, et al. Ectoparasitism and vector-borne diseases in 930 homeless people from Marseilles. Medicine. 2005;84(1):61-8.
1007. Jensenius M, Fournier PE, Kelly P, Myrvang B, Raoult D. African tick bite fever. Lancet Infectious Diseases. 2003;3(9):557-64.
1008. Auer J, Berent R, Punzengruber C, Eber B. Relapsing fever following a trip around the world. [German]. Tagliche Praxis. 2003;44(4):697-704.
1009. Picken RN, Picken MM. Molecular characterization of Borrelia spp. isolates from greater metropolitan Chicago reveals the presence of Borrelia bissettii. Preliminary report. Journal of Molecular Microbiology and Biotechnology. 2000;2(4):505-7.
1010. Golightly MG, Benach J. Tick-borne diseases. Reviews in Medical Microbiology. 1999;10(1):1-10.
1011. Zuger A. Relapsing fever in an HIV-infected man. AIDS clinical care. 1995;7(7):56, 9.
1012. Daniel E, Beyene H, Tessema T. Relapsing fever in children: Demographic, social and clinical features. Ethiopian Medical Journal. 1992;30(4):207-14.

1013. Komonguid DG, Diouf A, Rwakabayiza P, Ndiaye PD, Bah MD, Diadhiou F. Borreliosis and pregnancy. (Review of the literature apropos of 1 case). Dakar médical. 1990;35(1):14-7.
1014. Rummens JL, Louwagie A, Van Hoof A, Boelaert J, Gordts B, Van Landuyt HW. Relapsing fever imported into Belgium: A case report. Acta Clinica Belgica. 1987;42(3):210-4.
1015. Markovitz A. Thrombocytopenia in Colorado tick fever. West J Med. 1984;140(4):618.
1016. Galun E, Ben-Chetrit E. Possible prevention of tick-borne relapsing fever in patients infected with Borrelia recurrentis. The Journal of infectious diseases. 1984;150(4):617.
1017. Teklu B, Habte Michael A, Warrell DA. Meptazinol diminishes the Jarisch-Herxheimer reaction of relapsing fever. Lancet. 1983;1(8329):835-9.
1018. NEW TICK TROUBLES IN THE U.S.A. The Lancet. 1979;314(8140):450.
1019. Perine PL, Reynolds DF. Letter: Relapsing-fever epidemic in the Sudan and Ethiopia. Lancet. 1974;2(7892):1324-5.
1020. Abdalla RE. Some studies on relapsing fever in the Sudan. The Journal of tropical medicine and hygiene. 1969;72(5):125-8.
1021. Sparrow H. Study of the Ethiopian source of recurrent fever. Bulletin of the World Health Organization. 1958;19(4):673-710.
1022. Juarez E. New evaluation of the treatment of Spanish relapsing fever with aureomycin. Revista de sanidad e higiene pública. 1956;30(6-8):489-98.
1023. Anderson TR, Zimmerman LE. Relapsing Fever in Korea: A Clinicopathologic Study of Eleven Fatal Cases with Special Attention to Association with Salmonella Infections*. Am J Pathol. 1955;31(6):1083-109.
1024. Sparrow H. Mutation of a Spanish relapsing fever strain. Comptes rendus hebdomadaires des séances de l'Académie des sciences. 1954;239(15):922-4.
1025. Hirschboeck MM. The use of chloramphenicol in relapsing fever. The American journal of tropical medicine and hygiene. 1954;3(4):712-3.
1026. Gilges W. Relapsing fever simulating poliomyelitis; report of a case. South African medical journal = Suid-Afrikaanse tydskrif vir geneeskunde. 1951;25(35):622.
1027. Beet EA. Transmission of T. Duttoni. Br Med J. 1949;1(4602):501-2.
1028. Ashbel R. Notes on Spirochaeta persica from Palestine and spirochaetes of relapsing fever from the Western Desert (Tobruk area). Trans R Soc Trop Med Hyg. 1949;42(4):409.
1029. Loutfy Abul N. Sterile splenic abscess after relapsing fever. Lancet (London, England). 1948;1(6502):555-8.
1030. Gaud M, Morgan MT. Epidemiological Study on Relapsing Fever in North Africa (1943-1945). Bull World Health Organ. 1948;1(1):69-92.
1031. Friedmann I. Diagnosis of relapsing fever. British medical journal. 1948;1(4552):663.
1032. Sarrouy C, Groscolas R. [Recurrent fever in children; Epidemic 1944-1915]. Arch Fr Pediatr. 1947;4(2):121-6.
1033. Wolff BP. Asiatic relapsing fever; report of 134 cases treated with mapharsen. Annals of internal medicine. 1946;24:203-16.
1034. Grenoilleau G. [The epidemic of recurrent fever in Algeria in 1944]. Bull Acad Med. 1946;130(6-8):144.
1035. Gerard F. [Clinical Notes On 857 Cases Of Recurrent Worldwide Fever (Epidemic 1944-45)]. Maroc Med. 1945;24:138-42.
1036. Benhamou E. [Current aspects of the recurrent epidemic fever in North Africa]. Bull Acad Med. 1945;129(25-29):530-2.
1037. Greig EDW. An Epidemic of Relapsing Fever in Edinburgh in 1843. Edinb Med J. 1943;50(11):681-6.
1038. Robinson P. Relapsing Fever in Addis Ababa. Br Med J. 1942;2(4259):216-7.

1039. Legge RT. Relapsing Fever: A New Etiological Observation*: With Case Report of a Field Worker. Cal West Med. 1933;38(5):370-1.
1040. Varden AE. Relapsing Fever: Report of Case. Cal West Med. 1932;36(5):344-6.
1041. Nabarro D. AFRICAN TICK FEVER. Br Med J. 1931;1(3676):1095.
1042. Mallannah S. Relapsing Fever in Raichur. Ind Med Gaz. 1923;58(4):168.
1043. Newcomb C. On an Outbreak of Relapsing Fever in Turkey in 1918. Ind Med Gaz. 1920;55(6):208-17.
1044. Mendoza-Roldan JA, Ribeiro SR, Castilho-Onofrio V, Marcili A, Simonato BB, Latrofa MS, et al. Molecular detection of vector-borne agents in ectoparasites and reptiles from Brazil. Ticks and Tick-borne Diseases. 2021;12(1).
1045. Cutler SJ, Vayssier-Taussat M, Estrada-Peña A, Potkonjak A, Mihalca AD, Zeller H. Tick-borne diseases and co-infection: Current considerations. Ticks and Tick-borne Diseases. 2021;12(1).
1046. Wagemakers A, Sprong H, Platonov A, Hovius JW. Commentary: Borrelia miyamotoi: 43 Cases Diagnosed in France by Real-Time PCR in Patients With Persistent Polymorphic Signs and Symptoms. Front Med (Lausanne). 2020;7.
1047. Stewart PE, Bloom ME. Sharing the Ride: Ixodes scapularis Symbionts and Their Interactions. Front Cell Infect Microbiol. 2020;10.
1048. Snyman A, Vanstreels RET, Nell C, Schaefer AM, Stracke T, Parsons NJ, et al. Determinants of external and blood parasite load in African penguins (Spheniscus demersus) admitted for rehabilitation. Parasitology. 2020;147(5):577-83.
1049. Snavely E, Hymas W, Couturier MR, Couturier MR. Closing the brief case: Tick-borne relapsing fever in a returned traveler. Journal of Clinical Microbiology. 2020;58(6).
1050. Shokri A, Sabzevari S, Hashemi SA. Impacts of flood on health of Iranian population: Infectious diseases with an emphasis on parasitic infections. Parasite Epidemiol Control. 2020;9.
1051. Shakir SM, Mansfield CR, Hays ED, Couturier MR, Hillyard DR. Evaluation of a Novel High-Definition PCR Multiplex Assay for Simultaneous Detection of Tick-Borne Pathogens in Human Clinical Specimens. J Clin Microbiol. 2020;58(3).
1052. Rataud A, Dupraz M, Toty C, Blanchon T, Vittecoq M, Choquet R, et al. Evaluating Functional Dispersal in a Nest Ectoparasite and Its Eco-Epidemiological Implications. Front Vet Sci. 2020;7.
1053. Pollet T, Sprong H, Lejal E, Krawczyk AI, Moutailler S, Cosson JF, et al. The scale affects our view on the identification and distribution of microbial communities in ticks. Parasit Vectors. 2020;13.
1054. Pace EJ, O’Reilly M, O'Reilly M. Tickborne Diseases: Diagnosis and Management. American Family Physician. 2020;101(9):530-40.
1055. Nakayama S, Kobayashi T, Nakamura A, Yoshitomi H, Song Y, Ashizuka Y. Detection of Borrelia DNA in tick species collected from vegetation and wild animals in Fukuoka, Japan. Japanese Journal of Infectious Diseases. 2020;73(1):61-4.
1056. Munson E. Moving Targets of Bacterial Taxonomy Revision: What Are They and Why Should We Care? Clinical Microbiology Newsletter. 2020;42(14):111-20.
1057. Mendoza-Roldan JA, Modry D, Otranto D. Zoonotic Parasites of Reptiles: A Crawling Threat. Trends Parasitol. 2020;36(8):677-87.
1058. Mawanda P, Rwego I, Kisakye JJ, Sheil D. Rodents as potential hosts and reservoirs of parasites along the edge of a central african forest: Bwindi impenetrable national park, South Western Uganda. African Health Sciences. 2020;20(3):1168-78.

1059. Marks M, Johnston V, Brown M. Fever in the Returned Traveler. Hunter's Tropical Medicine and Emerging Infectious Diseases. 2020:1077-86.
1060. Lee-Lewandrowski E, Chen Z, Branda J, Baron J, Kaufman HW. Laboratory blood-based testing for non-lyme disease tick-borne infections at a national reference laboratory: A seven-year experience. American Journal of Clinical Pathology. 2020;153(1):139-45.
1061. Krishnavajhala A, Armstrong BA, Lopez JE. The impact of in vitro cultivation on the natural life cycle of the tick-borne relapsing fever spirochete Borrelia turicatae. PLoS ONE. 2020;15(10 October 2020).
1062. Krawczyk AI, van Duijvendijk GLA, Swart A, Heylen D, Jaarsma RI, Jacobs FHH, et al. Effect of rodent density on tick and tick-borne pathogen populations: consequences for infectious disease risk. Parasit Vectors. 2020;13.
1063. Hopkins H, Bassat Q, Chandler CI, Crump JA, Feasey NA, Ferrand RA, et al. Febrile Illness Evaluation in a Broad Range of Endemicities (FIEBRE): protocol for a multisite prospective observational study of the causes of fever in Africa and Asia. BMJ Open. 2020;10(7).
1064. Filatov S, Krishnavajhala A, Armstrong BA, Kneubehl AR, Nieto NC, De León AAP, et al. Isolation and Molecular Characterization of Tick-Borne Relapsing Fever Borrelia Infecting Ornithodoros (Pavlovskyella) verrucosus Ticks Collected in Ukraine. Journal of Infectious Diseases. 2020;221(5):804-11.
1065. Espinaze MPA, Hui C, Waller L, Matthee S. Nest-type associated microclimatic conditions as potential drivers of ectoparasite infestations in African penguin nests. Parasitology Research. 2020;119(11):3603-16.
1066. Elven J, Dahal P, Ashley EA, Thomas NV, Shrestha P, Stepniewska K, et al. Non-malarial febrile illness: a systematic review of published aetiological studies and case reports from Africa, 1980–2015. BMC Med. 2020;18.
1067. Colunga-Salas P, Sanchez-Montes S, Volkow P, Ruiz-Remigio A, Becker I. Mammalian species associated to Borrelia in Mexico. Figshare; 2020.
1068. Baron SA, Eldin C, Gouriet F, Dubourg G. Diagnostic tools for bacterial infections in travellers: Current and future options. Travel Medicine and Infectious Disease. 2020;37.
1069. Akhoundi M, Sereno D, Marteau A, Bruel C, Izri A. Who Bites Me? A Tentative Discriminative Key to Diagnose Hematophagous Ectoparasites Biting Using Clinical Manifestations. Diagnostics (Basel). 2020;10(5).
1070. Wisely SM, Glass GE. Advancing the Science of Tick and Tick-Borne Disease Surveillance in the United States. Insects. 2019;10(10).
1071. Vazquez-Guerrero E, Adan-Bante NP, Mercado-Uribe MC, Hernandez-Rodriguez C, Villa-Tanaca L, Lopez JE, et al. Localization of relapsing fever cases in Sonora, Mexico. Figshare; 2019.
1072. Vazquez-Guerrero E, Adan-Bante NP, Mercado-Uribe MC, Hernandez-Rodriguez C, Villa-Tanaca L, Lopez JE, et al. Case report: A retrospective serological analysis indicating human exposure to tick-borne relapsing fever spirochetes in Sonora, Mexico - Fig 2. Figshare; 2019.
1073. Thille KN, Rametta NF, Fitzpatrick DM, Springer CC, Tiwari K, Pinckney RD, et al. Ectoparasites of brown rats (Rattus norvegicus) in Grenada, West Indies. Vet World. 2019;12(9):1390-4.
1074. Telford SR, Goethert HK, Molloy PJ, Berardi V. Blood smears have poor sensitivity for confirming borrelia miyamotoi disease. Journal of Clinical Microbiology. 2019;57(3).

1075. Ramos JM, Pérez-Tanoira R, Martín-Martín I, Prieto-Pérez L, Tefasmariam A, Tiziano G, et al. Arthropod-Borne Bacteria Cause Nonmalarial Fever in Rural Ethiopia: A Cross-Sectional Study in 394 Patients. Vector Borne Zoonotic Dis. 2019;19(11):815-20.
1076. Portillo A, Palomar AM, de Toro M, Santibáñez S, Santibáñez P, Oteo JA. Exploring the bacteriome in anthropophilic ticks: To investigate the vectors for diagnosis. PLoS One. 2019;14(3).
1077. Porter WT, Motyka PJ, Wachara J, Barrand ZA, Hmood Z, McLaughlin M, et al. Citizen science informs human-tick exposure in the Northeastern United States. International journal of health geographics. 2019;18(1):9.
1078. Petersen LR, Beard CB, Visser SN. Combatting the Increasing Threat of Vector-Borne Disease in the United States with a National Vector-Borne Disease Prevention and Control System. Am J Trop Med Hyg. 2019;100(2):242-5.
1079. Parry EHO, Griffin GE. Relapsing fever. The Ecology of Health and Disease In Ethiopia2019. p. 399-406.
1080. Martins TF, Luz HR, Muñoz-Leal S, Ramirez DG, Milanelo L, Marques S, et al. A new species of Amblyomma (Acari: Ixodidae) associated with monkeys and passerines of the Atlantic rainforest biome, Southeastern Brazil. Ticks and Tick-borne Diseases. 2019;10(6).
1081. Mans BJ, Featherston J, Kvas M, Pillay KA, de Klerk DG, Pienaar R, et al. Argasid and ixodid systematics: Implications for soft tick evolution and systematics, with a new argasid species list. Ticks and Tick-borne Diseases. 2019;10(1):219-40.
1082. Lopez JE, Krishnavajhala A, Garcia MN, Bermudez S. Erratum: Lopez, J.E., et al. Tick-Borne Relapsing Fever Spirochetes in the Americas. Vet. Sci. 2016, 3, 16. Vet Sci. 2019;6(4).
1083. Linske MA, Stafford KC, Williams SC, Lubelczyk CB, Welch M, Henderson EF. Impacts of Deciduous Leaf Litter and Snow Presence on Nymphal Ixodes scapularis (Acari: Ixodidae) Overwintering Survival in Coastal New England, USA. Insects. 2019;10(8).
1084. Krishnavajhala A, Armstrong BA, Lopez JE. Erratum for Krishnavajhala et al., "Vector Competence of Geographical Populations of Ornithodoros turicata for the Tick-Borne Relapsing Fever Spirochete Borrelia turicatae". Applied and environmental microbiology. 2019;85(5).
1085. Kidd L. Optimal Vector-borne Disease Screening in Dogs Using Both Serology-based and Polymerase Chain Reaction–based Diagnostic Panels. Veterinary Clinics of North America - Small Animal Practice. 2019;49(4):703-18.
1086. Khan A, Nasreen N, Niaz S, Sajjad Ali Shah S, Mitchell RD, Ayaz S, et al. Tick burden and tick species prevalence in small ruminants of different agencies of the Federally Administered Tribal Areas (FATA), Pakistan. International Journal of Acarology. 2019;45(6-7):374-80.
1087. Haake DA, Yang XF. Spirochetes. Encyclopedia of Microbiology2019. p. 283-98.
1088. Gupta RS. Distinction between Borrelia and Borreliella is more robustly supported by molecular and phenotypic characteristics than all other neighbouring prokaryotic genera: Response to Margos' et al. "The genus Borrelia reloaded" (PLoS ONE 13(12): e0208432). PLoS One. 2019;14(8).
1089. Espinaze MPA, Hui C, Waller L, Dreyer F, Matthee S. Parasite diversity associated with African penguins (Spheniscus demersus) and the effect of host and environmental factors. Parasitology. 2019.
1090. Embers ME, Krishnavajhala A, Armstrong BA, Curtis MW, Pahar B, Wilder HK, et al. Immunological responses to the relapsing fever spirochete borrelia turicatae in infected rhesus macaques: Implications for pathogenesis and diagnosis. Infection and Immunity. 2019;87(4).

1091. Duncan R, Grigorenko E, Fisher C, Hockman D, Lanning B. Advances in multiplex nucleic acid diagnostics for blood-borne pathogens: promises and pitfalls - an update. Expert Review of Molecular Diagnostics. 2019;19(1):15-25.
1092. Díaz-Sánchez S, Hernández-Jarguín A, Torina A, de Mera IGF, Blanda V, Caracappa S, et al. Characterization of the bacterial microbiota in wild-caught Ixodes ventalloi. Ticks and Tick-borne Diseases. 2019;10(2):336-43.
1093. Dantas-Torres F, Fernandes Martins T, Muñoz-Leal S, Onofrio VC, Barros-Battesti DM. Ticks (Ixodida: Argasidae, Ixodidae) of Brazil: Updated species checklist and taxonomic keys. Ticks and Tick-borne Diseases. 2019;10(6).
1094. Bourret TJ, Boyle WK, Zalud AK, Valenzuela JG, Oliveira F, Lopez JE. The relapsing fever spirochete Borrelia turicatae persists in the highly oxidative environment of its soft-bodied tick vector. Cell Microbiol. 2019;21(2).
1095. Boulanger N, Boyer P, Talagrand-Reboul E, Hansmann Y. Ticks and tick-borne diseases. Medecine et Maladies Infectieuses. 2019;49(2):87-97.
1096. Boardman K, Rosenke K, Safronetz D, Feldmann H, Schwan TG. Host competency of the multimammate rat Mastomys natalensis demonstrated by prolonged spirochetemias with the African relapsing fever spirochete Borrelia crocidurae. American Journal of Tropical Medicine and Hygiene. 2019;101(6):1272-5.
1097. Bilbija B, Auer M, Široký P. Long term persistence of introduced Amblyomma geoemydae tick population under indoor conditions in Austria. Medical and Veterinary Entomology. 2019;33(2):317-21.
1098. Asante J, Noreddin A, El Zowalaty ME. Systematic Review of Important Bacterial Zoonoses in Africa in the Last Decade in Light of the 'One Health' Concept. Pathogens. 2019;8(2).
1099. Zolnik CP, Falco RC, Daniels TJ, Kolokotronis SO. Transient influence of blood meal and natural environment on blacklegged tick bacterial communities. Ticks and Tick-borne Diseases. 2018;9(3):563-72.
1100. Wilson KD, Elston D. What's eating you? Ixodes tick and related diseases, part 3: Coinfection and tick-bite prevention. Cutis. 2018;101(5):328-30.
1101. Wikel SK. Ticks and Tick-Borne Infections: Complex Ecology, Agents, and Host Interactions. Vet Sci. 2018;5(2).
1102. Sakamoto JM. Progress, challenges, and the role of public engagement to improve tick-borne disease literacy. Current Opinion in Insect Science. 2018;28:81-9.
1103. Rodríguez Y, Rojas M, Gershwin ME, Anaya JM. Tick-borne diseases and autoimmunity: A comprehensive review. Journal of Autoimmunity. 2018;88:21-42.
1104. Rabiee MH, Mahmoudi A, Siahsarvie R, Kryštufek B, Mostafavi E. Rodent-borne diseases and their public health importance in Iran. PLoS Negl Trop Dis. 2018;12(4).
1105. Portillo A, Ruiz-Arrondo I, Oteo JA. Arthropods as vectors of transmissible diseases in Spain☆. Med Clin (Engl Ed). 2018;151(11):450-9.
1106. Parsons NJ, Gous TA, Cranfield MR, Cheng LI, Schultz A, Horne E, et al. Novel vagrant records and occurrence of vector-borne pathogens in King Penguins (Aptenodytes patagonicus) in South Africa. Polar Biology. 2018;41(1):79-86.
1107. Palmer C, Landguth E, Stone E, Johnson T. The dynamics of vector-borne relapsing diseases. Mathematical Biosciences. 2018;297:32-42.
1108. Otranto D. Arthropod-borne pathogens of dogs and cats: From pathways and times of transmission to disease control. Veterinary Parasitology. 2018;251:68-77.
1109. Moon KL, Chown SL, Loh SM, Oskam CL, Fraser CI. Australian penguin ticks screened for novel Borrelia species. Ticks and Tick-Borne Diseases. 2018;9(2):410-4.

1110. Moodley A. Tick fever. The 5-Minute Pediatric Consult, 8th Edition2018. p. 932-3.
1111. Mitsakakis K, D'Acremont V, Hin S, von Stetten F, Zengerle R. Diagnostic tools for tackling febrile illness and enhancing patient management. Microelectron Eng. 2018;201:26-59.
1112. Maze MJ, Bassat Q, Feasey NA, Mandomando I, Musicha P, Crump JA. The epidemiology of febrile illness in sub-Saharan Africa: implications for diagnosis and management. Clin Microbiol Infect. 2018;24(8):808-14.
1113. Margos G, Gofton A, Wibberg D, Dangel A, Marosevic D, Loh S-M, et al. The genus Borrelia reloaded. PLoS One. 2018;13(12):e0208432.
1114. khosravani M. The fauna and perspective of rodentia ectoparasites in Iran relying on their roles within public health and veterinary characteristics. J Parasit Dis. 2018;42(1):1-18.
1115. Jiménez-Cortés JG, García-Contreras R, Bucio-Torres MI, Cabrera-Bravo M, Córdoba-Aguilar A, Benelli G, et al. Bacterial symbionts in human blood-feeding arthropods: Patterns, general mechanisms and effects of global ecological changes. Acta Tropica. 2018;186:69-101.
1116. Hagen RM, Frickmann H, Ehlers J, Krueger A, Margos G, Hizo-Teufel C, et al. Presence of Borrelia spp. DNA in ticks, but absence of Borrelia spp. and of Leptospira spp. DNA in blood of fever patients in Madagascar. Acta Tropica. 2018;177:127-34.
1117. Greay TL, Gofton AW, Paparini A, Ryan UM, Oskam CL, Irwin PJ. Recent insights into the tick microbiome gained through next-generation sequencing. Parasit Vectors. 2018;11.
1118. Garcia-Marti I, Zurita-Milla R, Harms MG, Swart A. Using volunteered observations to map human exposure to ticks. Sci Rep. 2018;8.
1119. Elelu N, Idris JM, Ahmed AO, Cutler SJ. Application of whole genome sequencing to study the genetic diversity of field collected ornithodorus ticks and the pathogens they carry from Nigeria. American Journal of Tropical Medicine and Hygiene. 2018;99(4):259.
1120. Doi K, Kato T, Hayama SI. Infestation of introduced raccoons (Procyon lotor) with indigenous ixodid ticks on the Miura Peninsula, Kanagawa Prefecture, Japan. International Journal for Parasitology: Parasites and Wildlife. 2018;7(3):355-9.
1121. Dantas-Torres F. Species Concepts: What about Ticks? Trends in Parasitology. 2018;34(12):1017-26.
1122. Breuner NE, Hojgaard A, Replogle AJ, Boegler KA, Eisen L. Transmission of the relapsing fever spirochete, Borrelia miyamotoi, by single transovarially-infected larval Ixodes scapularis ticks. Ticks and Tick-borne Diseases. 2018;9(6):1464-7.
1123. Varela-Stokes AS, Park SH, Kim SA, Ricke SC. Microbial Communities in North American Ixodid Ticks of Veterinary and Medical Importance. Front Vet Sci. 2017;4.
1124. Takahashi M, Misumi H, Tamura H. First record in Japan of tortoise tick Amblyomma Geoemydae (Cantor 1847) (Acari: Ixodidae) parasitizing Pryer's keelback snake Hebius Pryeri (Boulenger, 1887) (Reptile: Colubridae). International Journal of Acarology. 2017;43(4):314-9.
1125. Stone BL, Brissette CA. Host immune evasion by lyme and relapsing fever borreliae: Findings to lead future studies for Borrelia miyamotoi. Frontiers in Immunology. 2017;8(JAN).
1126. Sokhna C, Gaye O, Doumbo O. Developing Research in Infectious and Tropical Diseases in Africa: The Paradigm of Senegal. Clinical Infectious Diseases. 2017;65:S64-S9.
1127. Phe T, Lim K, Leng L, Verdonck K, Barbe B, Jacobs J, et al. Prevalence of neglected infectious diseases in patients with persistent fever in Sihanouk Hospital Center of HOPE, Cambodia. Tropical Medicine and International Health. 2017;22:42-3.

1128. Munderloh U. Comparative Studies in Tick-Borne Diseases in Animals and Humans. Vet Sci. 2017;4(2).
1129. Marcinkiewicz AL, Kraiczy P, Lin YP. There is a method to the madness: Strategies to study host complement evasion by lyme disease and relapsing fever spirochetes. Frontiers in Microbiology. 2017;8(MAR).
1130. Lager M, Faller M, Wilhelmsson P, Kjelland V, Andreassen A, Dargis R, et al. Results from panel III consisting of DNA extracted from 15 Borrelia strains and five specificity controls containing two relapsing fever strains (B. hermsii and B. miyamotoi), Treponema phagedenis and Leptospira. Figshare; 2017.
1131. Koetsveld J, Kolyasnikova NM, Wagemakers A, Toporkova MG, Sarksyan DS, Oei A, et al. Development and optimization of an in vitro cultivation protocol allows for isolation of Borrelia miyamotoi from patients with hard tick-borne relapsing fever. Clinical Microbiology and Infection. 2017;23(7):480-4.
1132. Kada S, McCoy KD, Boulinier T. Impact of life stage-dependent dispersal on the colonization dynamics of host patches by ticks and tick-borne infectious agents. Parasit Vectors. 2017;10.
1133. Jahfari S, Sarksyan DS, Kolyasnikova NM, Hovius JW, Sprong H, Platonov AE. Evaluation of a serological test for the diagnosis of Borrelia miyamotoi disease in Europe. Journal of Microbiological Methods. 2017;136:11-6.
1134. Gondard M, Cabezas-Cruz A, Charles RA, Vayssier-Taussat M, Albina E, Moutailler S. Ticks and Tick-Borne Pathogens of the Caribbean: Current Understanding and Future Directions for More Comprehensive Surveillance. Front Cell Infect Microbiol. 2017;7.
1135. Furuno K, Lee K, Itoh Y, Suzuki K, Yonemitsu K, Kuwata R, et al. Host-seeking ticks collected in Wakayama Prefecture. Figshare; 2017.
1136. Furuno K, Lee K, Itoh Y, Suzuki K, Yonemitsu K, Kuwata R, et al. Host-seeking ticks collected in Shimonoseki, Yamaguchi Prefecture. Figshare; 2017.
1137. Furuno K, Lee K, Itoh Y, Suzuki K, Yonemitsu K, Kuwata R, et al. Prevalence of Borrelia sp. in sika deer collected from Shimonoseki, Yamaguchi Prefecture. Figshare; 2017.
1138. Furuno K, Lee K, Itoh Y, Suzuki K, Yonemitsu K, Kuwata R, et al. Prevalence of Borrelia sp. in wild boars collected from Shimonoseki, Yamaguchi Prefecture. Figshare; 2017.
1139. Estrada-Peña A, Pfäffle M, Baneth G, Kleinerman G, Petney TN. Ixodoidea of the Western Palaearctic: A review of available literature for identification of species. Ticks and Tick-borne Diseases. 2017;8(4):512-25.
1140. Esfandiari B, Nahrevanian H, Pourshafie MR, Gouya MM, Khaki P, Mostafavi E, et al. Epidemiological Distribution of Rodents as Potent Reservoirs for Infectious Diseases in the Provinces of Mazandaran, Gilan and Golestan, Northern Iran. Infect Dis Rep. 2017;9(2).
1141. Delgado JD, Abreu-Yanes E, Abreu-Acosta N, Flor MD, Foronda P. Vertebrate ticks distribution and their role as vectors in relation to road edges and underpasses. Vector-Borne and Zoonotic Diseases. 2017;17(6):376-83.
1142. Boggild AK, Liles WC. Travel-Acquired Illnesses Associated with Fever. The Travel and Tropical Medicine Manual. 2017:271-99.
1143. Warwick BT, Bak E, Baldassarre J, Gregg E, Martinez R, Kioko J, et al. Abundance estimations of ixodid ticks on Boran cattle and Somali sheep in Northern Tanzania. International Journal of Acarology. 2016;42(1):12-7.
1144. Van Duijvendijk G, Coipan C, Wagemakers A, Fonville M, Ersöz J, Oei A, et al. Larvae of Ixodes ricinus transmit Borrelia afzelii and B. miyamotoi to vertebrate hosts. Parasites and Vectors. 2016;9(1).

1145. Trinachartvanit W, Hirunkanokpun S, Sudsangiem R, Lijuan W, Boonkusol D, Baimai V, et al. Borrelia sp. phylogenetically different from Lyme disease- and relapsing fever-related Borrelia spp. in Amblyomma varanense from Python reticulatus. Parasites and Vectors. 2016;9(1).
1146. Paulauskas A, Galdikaitė-Brazienė E, Radzijevskaja J, Aleksandravičienė A, Galdikas M. Genetic diversity of Ixodes ricinus (Ixodida: Ixodidae) ticks in sympatric and allopatric zones in Baltic countries. Journal of Vector Ecology. 2016;41(2):244-53.
1147. Nunes M, Parreira R, Maia C, Lopes N, Fingerle V, Vieira ML. Molecular identification of Borrelia genus in questing hard ticks from Portugal: Phylogenetic characterization of two novel Relapsing Fever-like Borrelia sp. Infection, Genetics and Evolution. 2016;40:266-74.
1148. Mohammad Saleh MS, Morsy ATA, Ismail MAM, Morsy TA. TICK-BORNE INFECTIOUS DISEASES WITH REFERENCE TO EGYPT. Journal of the Egyptian Society of Parasitology. 2016;46(2):273-98.
1149. Lopez JE, Krishnavajhala A. Vector competency of tick-borne relapsing fever spirochetes. American Journal of Tropical Medicine and Hygiene. 2016;95(5):408-9.
1150. Jacquot M, Abrial D, Gasqui P, Bord S, Marsot M, Masseglia S, et al. Multiple independent transmission cycles of a tick-borne pathogen within a local host community. Sci Rep. 2016;6.
1151. Inci A, Yildirim A, Duzlu O, Doganay M, Aksoy S. Tick-borne pathogens (TBPs), their hosts, and vector ticks in Turkey. Figshare; 2016.
1152. Hotez PJ. Southern Europe's Coming Plagues: Vector-Borne Neglected Tropical Diseases. PLoS Negl Trop Dis. 2016;10(6).
1153. Ehlers J, Ganzhorn JU, Silaghi C, Krüger A, Pothmann D, Yedidya Ratovonamana R, et al. Tick (Amblyomma chabaudi) infestation of endemic tortoises in southwest Madagascar and investigation of tick-borne pathogens. Ticks and Tick-borne Diseases. 2016;7(2):378-83.
1154. Donaldson TG, De Leon AAP, Li AI, Castro-Arellano I, Wozniak E, Boyle WK, et al. Collection summary of O. turicata in Texas and Florida. Figshare; 2016.
1155. Connally NP, Hinckley AF, Feldman KA, Kemperman M, Neitzel D, Wee SB, et al. TESTING PRACTICES AND VOLUME OF NON-LYME TICKBORNE DISEASES IN THE UNITED STATES. Ticks Tick Borne Dis. 2016;7(1):193-8.
1156. Vayssier-Taussat M, Kazimirova M, Hubalek Z, Hornok S, Farkas R, Cosson JF, et al. Emerging horizons for tick-borne pathogens: From the 'one pathogen-one disease' vision to the pathobiome paradigm. Future Microbiology. 2015;10(12):2033-43.
1157. Sharp SE. High Fevers in the High Desert in Oregon Borrelia Species Causing Recurrent Fever. Journal of Clinical Microbiology. 2015;53(8):2395-+.
1158. Platonov AE, Sarksyan DS, Karan LS, Shipulin GA, Gordygina EV, Malinin OV, et al. The blood coagulation system and microcirculatory disorders in ixodid tick-borne borreliosis caused by Borrelia miyamotoi. Terapevticheskii Arkhiv. 2015;87(11):26-32.
1159. Paddock CD, Goddard J. The evolving medical and veterinary importance of the gulf coast tick (Acari: Ixodidae). Journal of Medical Entomology. 2015;52(2):230-52.
1160. Kulkarni MA, Berrang-Ford L, Buck PA, Drebot MA, Lindsay LR, Ogden NH. Major emerging vector-borne zoonotic diseases of public health importance in Canada. Emerg Microbes Infect. 2015;4(6):e33-.
1161. Krause PJ, Hendrickson JE, Steeves TK, Fish D. Blood transfusion transmission of the tick-borne relapsing fever spirochete Borrelia miyamotoi in mice. Transfusion. 2015;55(3):593-7.

1162. Krajacich BJ, Lopez JE, Raffel SJ, Schwan TG. Vaccination with the variable tick protein of the relapsing fever spirochete Borrelia hermsii protects mice from infection by tick-bite. Parasites and Vectors. 2015;8(1).
1163. Hook SA, Nelson CA, Mead PS. U.S. public's experience with ticks and tick-borne diseases: Results from national HealthStyles surveys. Ticks Tick Borne Dis. 2015;6(4):483-8.
1164. Brites-Neto J, Duarte KMR, Martins TF. Tick-borne infections in human and animal population worldwide. Vet World. 2015;8(3):301-15.
1165. Avšič-Županc T. New vector-transmitted pathogens. Clinical Microbiology and Infection. 2015;21(7):611-3.
1166. Araya-Anchetta A, Busch JD, Scoles GA, Wagner DM. Thirty years of tick population genetics: A comprehensive review. Infection, Genetics and Evolution. 2015;29:164-79.
1167. Vu Hai V, Almeras L, Socolovschi C, Raoult D, Parola P, Pagès F. Monitoring human tick-borne disease risk and tick bite exposure in Europe: Available tools and promising future methods. Ticks and Tick-borne Diseases. 2014;5(6):607-19.
1168. Trape JF, Diatta G, Arnathau C, Bitam I, Sarih M, Belghyti D, et al. Correction: The Epidemiology and Geographic Distribution of Relapsing Fever Borreliosis in West and North Africa, with a Review of the Ornithodoros erraticus Complex (Acari: Ixodida). PLoS One. 2014;9(1).
1169. Tijsse-Klasen E, Koopmans MPG, Sprong H. Tick-Borne Pathogen – Reversed and Conventional Discovery of Disease. Front Public Health. 2014;2.
1170. Teegler A, Herzberger P, Margos G, Fingerle V, Kraiczy P. The relapsing fever spirochete Borrelia miyamotoi resists complement-mediated killing by human serum. Ticks and Tick-borne Diseases. 2014;5(6):898-901.
1171. Sakamoto JM, Goddard J, Rasgon JL. Population and Demographic Structure of Ixodes scapularis Say in the Eastern United States. PLoS One. 2014;9(7).
1172. Perronne C. Lyme and associated tick-borne diseases: global challenges in the context of a public health threat. Front Cell Infect Microbiol. 2014;4.
1173. Nguyen AP. Other tick-borne diseases. Harwood-Nuss' Clinical Practice of Emergency Medicine: Sixth Edition2014.
1174. Mukhacheva TA, Kovalev SY. Borrelia spirochetes in Russia: Genospecies differentiation by real-time PCR. Ticks and Tick-borne Diseases. 2014;5(6):722-6.
1175. Mathison BA, Pritt BS. Laboratory Identification of Arthropod Ectoparasites. Clin Microbiol Rev. 2014;27(1):48-67.
1176. Marshall GS. Prolonged and recurrent fevers in children. Journal of Infection. 2014;68(SUPPL1):S83-S93.
1177. Lopez JE, Vinet-Oliphant H, Wilder HK, Brooks CP, Grasperge BJ, Morgan TW, et al. Real-time monitoring of disease progression in rhesus macaques infected with borrelia turicatae by tick bite. Journal of Infectious Diseases. 2014;210(10):1639-48.
1178. Elbir H, Abi-Rached L, Pontarotti P, Yoosuf N, Drancourt M. African relapsing Fever borreliae genomospecies revealed by comparative genomics. Front Public Health. 2014;2:43.
1179. Diatta G, Durand P, Duplantier JM, Granjon L, Mahe G, Renaud F, et al. Impact of climate change on tick-borne relapsing fever borreliosis distribution in West Africa. American Journal of Tropical Medicine and Hygiene. 2014;91(5):30.
1180. Conover MR, Vail RM. Human diseases from wildlife2014. 1-516 p.
1181. Chitanga S, Gaff H, Mukaratirwa S. Tick-borne pathogens of potential zoonotic importance in the southern African region. Journal of the South African Veterinary Association. 2014;85(1).

1182. Boyle WK, Wilder HK, Lawrence AM, Lopez JE. Transmission Dynamics of Borrelia turicatae from the Arthropod Vector. PLoS Neglected Tropical Diseases. 2014;8(4).
1183. Bonilla D, Kjemtrup A, Vilcins I-M, Hui L, Sola M, Quintana M, et al. California Ixodes pacificus adult and nymphal ticks, tested in pools or tested individually, 2000-2012. Figshare; 2014.
1184. Barbour AG. Phylogeny of a relapsing fever Borrelia species transmitted by the hard tick Ixodes scapularis. Infection, Genetics and Evolution. 2014;27:551-8.
1185. Adeolu M, Gupta RS. A phylogenomic and molecular marker based proposal for the division of the genus Borrelia into two genera: The emended genus Borrelia containing only the members of the relapsing fever Borrelia, and the genus Borreliella gen. nov. containing the members of the Lyme disease Borrelia (Borrelia burgdorferi sensu lato complex). Antonie van Leeuwenhoek, International Journal of General and Molecular Microbiology. 2014;105(6):1049-72.
1186. Wu XB, Na RH, Wei SS, Zhu JS, Peng HJ. Distribution of tick-borne diseases in China. Parasit Vectors. 2013;6:119.
1187. Uilenberg G, Estrada-Peña A, Thal J. Ticks of the Central African Republic. Experimental and Applied Acarology. 2013;60(1):1-40.
1188. Sapi E, Pabbati N, Datar A, Davies EM, Rattelle A, Kuo BA. Improved Culture Conditions for the Growth and Detection of Borrelia from Human Serum. Int J Med Sci. 2013;10(4):362-76.
1189. Ratmanov P, Mediannikov O, Raoult D. Vectorborne diseases in West Africa: Geographic distribution and geospatial characteristics. Transactions of the Royal Society of Tropical Medicine and Hygiene. 2013;107(5):273-84.
1190. Pfäffle M, Littwin N, Muders SV, Petney TN. The ecology of tick-borne diseases. International Journal for Parasitology. 2013;43(12-13):1059-77.
1191. Obsomer V, Wirtgen M, Linden A, Claerebout E, Heyman P, Heylen D, et al. Spatial disaggregation of tick occurrence and ecology at a local scale as a preliminary step for spatial surveillance of tick-borne diseases: general framework and health implications in Belgium. Parasit Vectors. 2013;6:190.
1192. Mohammadi M, Ghavami MB, Mohammadi J. Study of phenotypic variability and borrelia infectivity in different populations of ornithodoros tholozani in zanjan province. Journal of Zanjan University of Medical Sciences and Health Services. 2013;21(87):83-93.
1193. Mihalca AD, Sándor AD. The role of rodents in the ecology of ixodes ricinus and associated pathogens in central and Eastern Europe. Frontiers in Cellular and Infection Microbiology. 2013;4(OCT).
1194. Liveris D, Hanincová K, Schwartz I. Borreliae. Molecular Typing in Bacterial Infections2013. p. 353-69.
1195. Liu DY. Argasidae (Soft Ticks). Liu D, editor2013. 733-40 p.
1196. Fish D. Borrelia miyamotoi: More lessons on disease discovery. Annals of Internal Medicine. 2013;159(9):648.
1197. Eisen L, Wong D, Shelus V, Eisen RJ. What is the Risk for Exposure to Vector-Borne Pathogens in United States National Parks? J Med Entomol. 2013;50(2):221-30.
1198. Steven Pray W, Pray GE. Preventing and recognizing tick-borne diseases. US Pharmacist. 2012;37(8):7.
1199. Reye AL, Arinola OG, Hübschen JM, Muller CP. Pathogen Prevalence in Ticks Collected from the Vegetation and Livestock in Nigeria. Appl Environ Microbiol. 2012;78(8):2562-8.

1200. Marcsisin RA, Campeau SA, Lopez JE, Barbour AG. Alp, an arthropod-associated outer membrane protein of borrelia species that cause relapsing fever. Infection and Immunity. 2012;80(5):1881-90.
1201. Gutiérrez RL, Decker CF. Prevention of Tick-Borne Illness. Disease-a-Month. 2012;58(6):377-87.
1202. Dantas-Torres F, Chomel BB, Otranto D. Ticks and tick-borne diseases: A One Health perspective. Trends in Parasitology. 2012;28(10):437-46.
1203. Charles RA, Kjos S, Ellis AE, Dubey J, Shock BC, Yabsley MJ. Parasites and vector-borne pathogens of southern plains woodrats (Neotoma micropus) from southern Texas. Parasitol Res. 2012;110(5):1855-62.
1204. Campeau SA, Barbour AG. Antigenic Variation in the Relapsing Fever Agent Borrelia parkeri from Northern California. Abstracts of the General Meeting of the American Society for Microbiology. 2012;112:2197.
1205. Bryant K. Tickborne Infections. Principles and Practice of Pediatric Infectious Diseases: Fourth Edition2012. p. 531-6.e3.
1206. Bermúdez C SE, Castro A, Esser H, Liefting Y, García G, Miranda RJ. Ticks (Ixodida) on humans from central Panama, Panama (2010-2011). Experimental and Applied Acarology. 2012;58(1):81-8.
1207. Auwaerter PG, Flynn JA. Lyme disease and other tick-borne illnesses. Principles of Ambulatory Medicine: Seventh Edition2012. p. 560-70.
1208. Aarsland SJ, Castellanos-Gonzalez A, Lockamy KP, Mulu-Droppers R, Mulu M, White AC, et al. Treatable Bacterial Infections Are Underrecognized Causes of Fever in Ethiopian Children. Am J Trop Med Hyg. 2012;87(1):128-33.
1209. Oshaghi MA, Rafinejad J, Choubdar N, Piazak N, Vatandoost H, Telmadarraiy Z, et al. Discrimination of relapsing fever Borrelia persica and Borrelia microtti by diagnostic species-specific primers and polymerase chain reaction-restriction fragment length polymorphism. Vector-Borne and Zoonotic Diseases. 2011;11(3):201-7.
1210. Naddaf SR, Khajevand M, Ghazinezhad B. Identification of relapsing fever Borrelia persica and Borrelia microtti by diagnostic species-specific PCR based on flagellin (flaB) gene. Tropical Medicine and International Health. 2011;16:248.
1211. Kestler AM. Mosquito- and tick-borne diseases of north america. Emergency Medicine Secrets2011. p. 360-7.
1212. Igreja RP. Infectious diseases associated with caves. Wilderness & environmental medicine. 2011;22(2):115-21.
1213. Georgiades K, Raoult D. Defining pathogenic bacterial species in the genomic era. Frontiers in Microbiology. 2011;1(JAN).
1214. Foley JE, Nieto NC. The ecology of tick-transmitted infections in the redwood chipmunk (Tamias ochrogenys). Ticks and Tick-borne Diseases. 2011;2(2):88-93.
1215. Fleer KA, Foley P, Calder L, Foley JE. Arthropod vectors and vector-borne bacterial Pathogens in Yosemite National Park. Journal of Medical Entomology. 2011;48(1):101-10.
1216. Cutler S, Adamu H, Abdissa A, Tolosa T, Gashaw A. Argas persicus Ethiopian soft ticks as disease vectors. Clinical Microbiology and Infection. 2011;17:S212.
1217. Toledo A, Anda P, Escudero R, Larsson C, Bergstrom S, Benach JL. Phylogenetic analysis of a virulent Borrelia species isolated from patients with relapsing fever. Journal of Clinical Microbiology. 2010;48(7):2484-9.
1218. Smith WCS, Mwanthi MA. Tick-borne disease - changing patterns and effective interventions. Environmental Medicine2010. p. 447-51.

1219. Safdie G, Farrah IY, Yahia R, Marva E, Wilamowski A, Sawalha SS, et al. Molecular characterization of borrelia persica, the agent of tick borne relapsing fever in israel and the palestinian authority. PLoS ONE. 2010;5(11).
1220. Pages F, Faulde M, Orlandi-Pradines E, Parola P. The past and present threat of vector-borne diseases in deployed troops. Clinical Microbiology and Infection. 2010;16(3):209-24.
1221. Lundqvist J, Larsson C, Nelson M, Andersson M, Bergström S, Persson C. Concomitant infection decreases the malaria burden but escalates relapsing fever borreliosis. Infection and Immunity. 2010;78(5):1924-30.
1222. Lopez JE, Schrumpf ME, Schwan TG. Acquisition and Transmission of Relapsing Fever Spirochetes, Borrelia hermsii, by the Soft Tick Ornithodoros hermsi. Abstracts of the General Meeting of the American Society for Microbiology. 2010;110:D-665.
1223. Lopez JE, Schrumpf ME, Nagarajan V, Raffel SJ, McCoy BN, Schwan TG. A novel surface antigen of relapsing fever spirochetes can discriminate between relapsing fever and lyme borreliosis. Clinical and Vaccine Immunology. 2010;17(4):564-71.
1224. Cutler SJ, Bonilla EM, Singh RJ. Population structure of East African relapsing fever Borrelia spp. Emerg Infect Dis. 2010;16(7):1076-80.
1225. Clemens EG, Schachterle S, Reller ME, Mtove G, Sullivan D, Dumler J. Multiplex 5 ' Nuclease qPCR for the Diagnosis of Relapsing Fever in Large Clinical Cohorts. Abstracts of the General Meeting of the American Society for Microbiology. 2010;110:Y-1494.
1226. Vial L. Biological and ecological characteristics of soft ticks (Ixodida: Argasidae) and their impact for predicting tick and associated disease distriblftion. Parasite. 2009;16(3):191-202.
1227. Tokarz R, Kapoor V, Samuel JE, Bouyer DH, Briese T, Lipkin WI. Detection of Tick-Borne Pathogens by MassTag Polymerase Chain Reaction. Vector Borne Zoonotic Dis. 2009;9(2):147-51.
1228. Magnarelli LA. Global Importance of Ticks and Associated Infectious Disease Agents. Clinical Microbiology Newsletter. 2009;31(5):33-7.
1229. Lopez JE, Porcella SF, Schrumpf ME, Raffel SJ, Hammer CH, Zhao M, et al. Identification of conserved antigens for early serodiagnosis of relapsing fever Borrelia. Microbiology. 2009;155(8):2641-51.
1230. Isrctn. Insecticide-treated bednets for control of domestic ticks and prevention of African tick-borne relapsing fever. http://wwwwhoint/trialsearch/Trial2aspx?TrialID=ISRCTN04521623. 2009.
1231. Hotez PJ, Kamath A. Neglected Tropical Diseases in Sub-Saharan Africa: Review of Their Prevalence, Distribution, and Disease Burden. PLoS Negl Trop Dis. 2009;3(8).
1232. Heaulme M, Couvreur P, Sicard JM. Tick bite fever in West Africa: Difficulties in diagnosis of an emerging disease. [French]. Medecine Tropicale. 2009;69(6):595-8.
1233. Georgiev VS. Emerging and Re-emerging Infectious Diseases. National Institute of Allergy and Infectious Diseases, NIH. 2009:23-8.
1234. Enayati A, Asgarian F, Amouei A, Hemingway J. The first report of two Ornithodoros tick species, the main vectors of relapsing fever from North of Iran, and a review of the disease situation in the country. Tropical Medicine and International Health. 2009;14:222.
1235. Cutler SJ. Myths, legends and realities of relapsing fever borreliosis. Clinical Microbiology and Infection. 2009;15(5):395-6.
1236. Cutler S. Uncovering the secrets of East African relapsing fever. Clinical Microbiology and Infection. 2009;15:S261.

1237. Cadavid D, Londoño D. Understanding tropism and immunopathological mechanisms of relapsing fever spirochaetes. Clinical Microbiology and Infection. 2009;15(5):415-21.
1238. Borrelia [bə-rel′e-ə]. Emerg Infect Dis. 2009;15(7):1025.
1239. Tabuchi N, Kataoka-Ushijima Y, Talbert A, Mitani H, Fukunaga M. Absence of transovarial transmission of Borrelia duttonii, a tick-borne relapsing fever agent, by the vector tick Ornithodoros moubata. Vector-Borne and Zoonotic Diseases. 2008;8(5):607-13.
1240. Sylla PM, Molez JF, Cornet JP, Camicas JL. Climate change and distribution of ticks (Acari: Ixodida) in Senegal and Mauritania. Acarologia. 2008;48(3-4):137-53.
1241. McCall PJ. TBRF IN EAST AFRICA: EPIDEMIOLOGY AND CLINICAL DIAGNOSIS IN CENTRAL TANZANIA. American Journal of Tropical Medicine and Hygiene. 2008;79(6, Suppl. S):229-30.
1242. Marseille-Nice G. Causes tick-borne relapsing fever in east Africa. European Nucleotide Archive; 2008.
1243. Larsson C, Lundqvist J, Bergström S. Residual brain infection in murine relapsing fever borreliosis can be successfully treated with ceftriaxone. Microbial Pathogenesis. 2008;44(3):262-4.
1244. Kisinza WN, Talbert A, Mutalemwa P, McCall PJ. Community knowledge, attitudes and practices related to tick-borne relapsing fever in dodoma rural district, central Tanzania. Tanzania journal of health research. 2008;10(3):131-6.
1245. Francischetti IMB, Mans BJ, Meng ZJ, Gudderra N, Veenstra TD, Pham VM, et al. An insight into the sialome of the soft tick, Ornithodorus parkeri. Insect Biochemistry and Molecular Biology. 2008;38(1):1-21.
1246. Ticks: Biology, Disease and Control. Bowman AS, Nuttall PA, editors2008.
1247. Infectious Diseases and Anthropods, 2nd Edition. Goddard J, editor2008.
1248. Schwan TG, Raffel SJ, Schrumpf ME, Porcella SF. Diversity and distribution of Borrelia hermsii. Emerging Infectious Diseases. 2007;13(3):436-42.
1249. Reeves WK, Durden LA, Ritzi CM, Beckham KR, Super PE, Oconnor BM. Ectoparasites and other ectosymbiotic arthropods of vertebrates in the Great Smoky Mountains National Park, USA. Zootaxa. 2007(1392):31-68.
1250. Ogden NH, Bigras-Poulin M, O'Callaghan CJ, Barker IK, Kurtenbach K, Lindsay LR, et al. Vector seasonality, host infection dynamics and fitness of pathogens transmitted by the tick Ixodes scapularis. Parasitology. 2007;134(2):209-27.
1251. Meri T, Pusa E, Cutler S, Jokiranta TS. A novel classical pathway evasion mechanism by relapsing fever Borreliae. Molecular Immunology. 2007;44(1-3, Sp. Iss. SI):215.
1252. Andersson M, Nordstrand A, Shamaei-Tousi A, Jansson A, Bergström S, Guo BP. In situ immune response in brain and kidney during early relapsing fever borreliosis. Journal of Neuroimmunology. 2007;183(1-2):26-32.
1253. Rocky Mountain L. Causes tick-borne relapsing fever. European Nucleotide Archive; 2006.
1254. Pope V, Ari MD, Schriefer ME, Levett PN. Immunologic Methods for Diagnosis of Spirochetal Diseases. Detrick B, Hamilton RG, Folds JD, editors2006. 477-92 p.
1255. McCall PJ. New developments in the epidemiology and control of tick-borne relapsing fever in east Africa. American Journal of Tropical Medicine and Hygiene. 2006;75(5, Suppl. S):1-2.
1256. Laboratory of Human Bacterial P. Causes tick-borne relapsing fever. European Nucleotide Archive; 2006.
1257. Hovis KM, Schriefer ME, Bahlani S, Marconi RT. Immunological and molecular analyses of the Borrelia hermsii factor H and factor H-like protein 1 binding protein, FhbA:

Demonstration of its utility as a diagnostic marker and epidemiological tool for tick-borne relapsing fever. Infection and Immunity. 2006;74(8):4519-29.
1258. Horak IG, McKay IJ, Henen BT, Heyne H, Hofmeyr MD, De Villiers AL. Parasites of domestic and wild animals in South Africa. XLVII. Ticks of tortoises and other reptiles. Onderstepoort Journal of Veterinary Research. 2006;73(3):215-27.
1259. Halos L, Mavris M, Vourc'h G, Maillard R, Barnouin J, Boulouis HJ, et al. Broad-range PCR-TTGE for the first-line detection of bacterial pathogen DNA in ticks. Veterinary Research. 2006;37(2):245-53.
1260. Gratz NG. The vector- and rodent-borne diseases of Europe and North America: Their distribution and public health burden2006. 1-393 p.
1261. Genchi C. Ecology and epidemiology of tick-borne diseases: Which role for the control? Parassitologia. 2006;48(1-2):137-8.
1262. Cadavid D, Sondey M, Garcia E, Lawson CL. Residual brain infection in relapsing-fever borreliosis. Journal of Infectious Diseases. 2006;193(10):1451-8.
1263. Cadavid D. The mammalian host response to borrelia infection. Wiener Klinische Wochenschrift. 2006;118(21-22):653-8.
1264. Ullmann AJ, Gabitzsch ES, Schulze TL, Zeidner NS, Piesman J. Three multiplex assays for detection of Borrelia burgdorferi sensu lato and Borrelia miyamotoi sensu lato in field-collected Ixodes nymphs in North America. J Med Entomol. 2005;42(6):1057-62.
1265. Smith RP. Tick-Borne Diseases of Humans. Emerg Infect Dis. 2005;11(11):1808-9.
1266. Schwan TG, Raffel SJ, Schrumpf ME, Policastro PF, Rawlings JA, Lane RS, et al. Phylogenetic analysis of the spirochetes Borrelia parkeri and Borrelia turicatae and the potential for tick-borne relapsing fever in Florida. Journal of Clinical Microbiology. 2005;43(8):3851-9.
1267. Ruscio M. Laboratory medicine response in transmitted tick-borne diseases. Rivista Italiana della Medicina di Laboratorio. 2005;1(SUPPL. 3):103-7.
1268. Nct. Post Exposure Treatment With Doxycycline for the Prevention of Relapsing Fever. https://clinicaltrialsgov/show/NCT00237016. 2005.
1269. Márquez-Jiménez FJ, Hidalgo-Pontiveros A, Contreras-Chova F, Rodríguez-Liébana JJ, Muniain-Ezcurra MA. Ticks (Acarina: Ixodidae) as vectors and reservoirs of pathogen microorganims in Spain. Enfermedades Infecciosas y Microbiologia Clinica. 2005;23(2):94-102.
1270. Londoño D, Bai Y, Zückert WR, Gelderblom H, Cadavid D. Cardiac apoptosis in severe relapsing fever borreliosis. Infection and Immunity. 2005;73(11):7669-76.
1271. American Academy of Family P. Information from your family doctor. Tick-borne relapsing fever: what you should know. American family physician. 2005;72(10):2046.
1272. Shao R, Aoki Y, Mitani H, Tabuchi N, Barker SC, Fukunaga M. The mitochondrial genomes of soft ticks have an arrangement of genes that has remained unchanged for over 400 million years. Insect Molecular Biology. 2004;13(3):219-24.
1273. Sambri V, Marangoni A, Storni E, Cavrini F, Moroni A, Sparacino M, et al. Tick borne zoonosis: selected clinical and diagnostic aspects. [Italian]. Parassitologia. 2004;46(1-2):109-13.
1274. Lin T, Oliver JH, Jr., Gao L. Molecular characterization of Borrelia isolates from ticks and mammals from the southern United States. J Parasitol. 2004;90(6):1298-307.
1275. Calza L, Manfredi R, Chiodo F. Tick-borne infections. [Italian]. Recenti Progressi in Medicina. 2004;95(9):403-13.
1276. Bunikis J, Tsao J, Garpmo U, Berglund J, Fish D, Barbour AG. Typing of Borrelia relapsing fever group strains. Emerging Infectious Diseases. 2004;10(9):1661-4.

1277. Smith RP. Tick-borne infections. Medical Management of Infectious Disease2003. p. 599-619.
1278. Richter D, Schlee DB, Matuschka FR. Relapsing fever-like spirochetes infecting European vector tick of Lyme disease agent. Emerging Infectious Diseases. 2003;9(6):697-701.
1279. Esposito AL, Abraham G. Infections in the patient with animal contact. Medical Management of Infectious Disease2003. p. 709-22.
1280. Cousins JK, Bono MJ. Clinician's guide to tick-borne disease. Emergency Medicine. 2003;35(6):42-50.
1281. Cornish NE, Romero JR, Iwen PC. Rapid identification of Borrelia hermsii infection in a pediatric patient utilizing a molecular-based approach. Abstracts of the General Meeting of the American Society for Microbiology. 2003;103:C-215.
1282. Nct. Post Exposure Treatment With Doxycycline for the Prevention of Relapsing Fever. Https://clinicaltrialsgov/show/nct00237016. 2002.
1283. George JC, Chastel C. Tick-borne diseases and ecosystem changes in Lorraine. Bulletin de la Societe de Pathologie Exotique. 2002;95(2):95-9.
1284. Da Costa IP, Bonoldi VLN, Yoshinari NH. Search for Borrelia sp. in ticks collected from potential reservoirs in an urban forest reserve in the State of Mato Grosso do Sul, Brazil: A short report. Memorias do Instituto Oswaldo Cruz. 2002;97(5):631-5.
1285. Barbour A, Craig NL, Craigie R, Gellert M, Lambowitz AM. Antigenic variation by relapsing fever Borrelia species and other bacterial pathogens2002. 972-94 p.
1286. Scoles GA, Papero M, Beati L, Fish D. A relapsing fever group spirochete transmitted by Ixodes scapularis ticks. Vector borne and zoonotic diseases (Larchmont, NY). 2001;1(1):21-34.
1287. Rich SM, Armstrong PM, Smith RD, Telford, Sr. Lone Star tick-infecting Borreliae are most closely related to the agent of bovine borreliosis. Journal of Clinical Microbiology. 2001;39(2):494-7.
1288. Parola P, Raoult D. Tick-borne bacterial diseases emerging in Europe. Clinical Microbiology and Infection. 2001;7(2):80-3.
1289. Cobey FC, Goldbarg SH, Levine RA, Patton CL. Short report: Detection of Borrelia (relapsing fever) in rural Ethiopia by means of the quantitative buffy coat technique. American Journal of Tropical Medicine and Hygiene. 2001;65(2):164-5.
1290. Cimolai N, Oksi J, Viljanen MK. Borrelioses. Laboratory Diagnosis of Bacterial Infections2001. p. 747-76.
1291. Alciati S, Belligni E, Del Colle S, Pugliese A. Human infections tick-transmitted. Panminerva Medica. 2001;43(4):295-304.
1292. Roberts DM, Carlyon JA, Theisen M, Marconi RT. The bdr gene families of the Lyme disease and relapsing fever spirochetes: Potential influence on biology, pathogenesis, and evolution. Emerging Infectious Diseases. 2000;6(2):110-22.
1293. Niren AS, Sundararaj T, Jayapal V, Subramanian S. Studies on the reemerging Borrelial infection in man. Biomedicine. 2000;20(4):236-42.
1294. Korenberg EI, Nikolenko VV, Vorobyeva NN, Frizen TN, Moskvitina GG. Indirect immunofluorescence assay in laboratory diagnosis of ixodid tick-borne relapsing fever. Meditsinskaya Parazitologiya i Parazitarnye Bolezni. 2000(3):9-16.
1295. Hartmann P, Schulz H, Romer K, Engert A, Salzberger B. Relapsing fever (Borrelia spec.): An important differential diagnosis in patients returning from Africa. Onkologie. 2000;23(Sonderheft 7):186.

1296. Cutler SJ, Jones SE, Wright DJM, Zhang H. Cultivation of East African relapsing fever Borrelia and review of preceding events. Journal of Spirochetal and Tick-borne Diseases. 2000;7(FALL):52-8.
1297. Zyuzya YP, Yefimova NS, Vorobyeva NN, Klitsunova NV, Gosteva VV. Clinical and morphologic manifestations of migrating erythema in patients with ixodes tick-borne relapsing fever. Meditsinskaya Parazitologiya i Parazitarnye Bolezni. 1999(4):36-41.
1298. Trape JE. Climatic changes and infectious diseases: Malaria and tick-borne relapsing borreliosis. Medecine Et Maladies Infectieuses. 1999;29(5):296-300.
1299. Sparagano OAE, Allsopp MTEP, Mank RA, Rijpkema SGT, Figueroa JV, Jongejan F. Molecular detection of pathogen DNA in ticks (Acari: Ixodidae): A review. Experimental and Applied Acarology. 1999;23(12):929-60.
1300. Soumare M, Diop BM, Ndour CT, Dieng Y, Badiane S. Epidemiological aspects of borreliosis in a hospital setting in Dakar, Senegal. Medecine Et Maladies Infectieuses. 1999;29(3):191-4.
1301. Goddard J. Tick-Borne Relapsing Fever. Infections in Medicine. 1999;16(10):632-4.
1302. Faul JL, Doyle RL, Kao PN, Ruoss SJ. Tick-borne pulmonary disease: Update on diagnosis and management. Chest. 1999;116(1):222-30.
1303. Cobey FC, Goldbarg SH, Levine RA, Charette L, Patton CL, Tiruha D. Borreliosis relapsing fever, diagnosed by quantitative buffy coat (QBC) fluorescent microscopy. American Journal of Tropical Medicine and Hygiene. 1999;61(3 SUPPL.):419-20.
1304. Anonymous. Relapsing fever: How borrelia outsmart the immune system. [German]. Deutsche Apotheker Zeitung. 1999;139(9):38-9.
1305. Yoder JA, Dutton AM. Water requirements of relapsing fever tick larvae, Ornithodoros turicata (Acari: Argasidae), and the relative humidity of hatching eggs. International Journal of Acarology. 1998;24(1):87-91.
1306. Wright DJM. The relapsing fever spirochaete: Recognition of fulfillment of Koch's postulates. Journal of Medical Microbiology. 1998;47(5):463.
1307. Van Dam AP, Van Gool T, Wetsteyn JCFM, Lommerse E, Dankert J. Borrelia crocidurae relapsing fever diagnosed by the quantitative buffy coat (QBC) technique and in vitro culture. Abstracts of the General Meeting of the American Society for Microbiology. 1998;98:164.
1308. Talbert A. Correction: Spraying tick-infested houses with lambda-cyhalothrin reduces the incidence of tick-borne relapsing fever in children under five years old (Transactions of The Royal Society of Tropical Medicine and Hygiene (1998) 92 (251-253). Transactions of the Royal Society of Tropical Medicine and Hygiene. 1998;92(4):450.
1309. Alugupalli KR, Barnard MR, Schwan TG, Coburn J, Michelson AD, Leong JM. Integrin binding and platelet activation by Borrelia hermsii, an agent of tick-borne relapsing fever. Abstracts of the General Meeting of the American Society for Microbiology. 1998;98:108.
1310. Shapiro ED. Tick-borne diseases. Adv Pediatr Infect Dis. 1997;13:187-218.
1311. Sawae Y. [Microbiological tests for determination of specific microbes--Treponema , Leptospira and Borrelia]. Rinsho Byori. 1997;Suppl 105:135-40.
1312. Gratz NG. The burden of rodent-borne diseases in Africa south of the Sahara. Belgian Journal of Zoology. 1997;127(SUPPL.):71-84.
1313. Cutler SJ, Moss J, Fukunaga M, Wright DJM, Fekade D, Warrell D. Borrelia recurrentis characterization and comparison with relapsing- fever, lyme-associated, and other Borrelia spp. International Journal of Systematic Bacteriology. 1997;47(4):958-68.
1314. Byrd RP, Jr., Vasquez J, Roy TM. Respiratory manifestations of tick-borne diseases in the Southeastern United States. South Med J. 1997;90(1):1-4.

1315. Anderson DE, Jr., Hoffmann ML, Schwan TG. Diagnosis of tick-borne relapsing fever (TBRF) by Darkfield microscopy. Abstracts of the Interscience Conference on Antimicrobial Agents and Chemotherapy. 1997;37:111.
1316. Ras NM, Lascola B, Postic D, Cutler SJ, Rodhain F, Baranton G, et al. Phylogenesis of relapsing fever Borrelia spp. Int J Syst Bacteriol. 1996;46(4):859-65.
1317. Phillips JS, Adeyeye OA. Reproductive bionomics of the soft tick, Ornithodoros turicata (Acari: Argasidae). Experimental and Applied Acarology. 1996;20(7):369-80.
1318. Dworkin MS, Anderson D, Jr., Hoffmann M, Schwan T, Shoemaker P, Kobayashi J, et al. Tick-borne relapsing fever in the Northwest: 1980-1995. Abstracts of the Interscience Conference on Antimicrobial Agents and Chemotherapy. 1996;36(0):289.
1319. Livesley MA, Thompson IP, Rainey PB, Nuttall PA. Comparison of Borrelia isolated from UK foci of Lyme disease. FEMS Microbiology Letters. 1995;130(2-3):151-7.
1320. Anderson DE, Jr., Schwan TG, Kassen BO, Dhingra V, Hoffmann ML, Dworkin M, et al. Endemic Borrelia hermsii (Bh) relapsing fever (RF) in the northwestern United States. Abstracts of the General Meeting of the American Society for Microbiology. 1995;95(0):37.
1321. Kawabata H, Tashibu H, Yamada K, Masuzawa T, Yanagihara Y. Polymerase Chain Reaction Analysis of Borrelia Species Isolated in Japan. Microbiology and Immunology. 1994;38(8):591-8.
1322. Breitschwerdt EB, Nicholson WL, Kiehl AR, Steers C, Meuten DJ, Levine JF. Natural infections with Borrelia spirochetes in two dogs from Florida. J Clin Microbiol. 1994;32(2):352-7.
1323. Tissot Dupont H, Raoult D. Tick-borne diseases. La Revue de medecine interne. 1993;14(5):300-6.
1324. Reed Jr GH. Lyme disease and other tick-borne diseases: A review. Journal of Environmental Health. 1993;55(8):6-10.
1325. Oldfield EC, Rodier GR, Gray GC. The Endemic Infectious Diseases of Somalia. Clinical Infectious Diseases. 1993;16:S132-S57.
1326. Morshed MG, Konishi H, Nishimura T, Nakazawa T. Evaluation of agents for use in medium for selective isolation of Lyme disease and relapsing fever Borrelia species. European Journal of Clinical Microbiology and Infectious Diseases. 1993;12(7):512-8.
1327. Schwan TG, Gage KL, Karstens RH, Schrumpf ME, Hayes SF, Barbour AG. Identification of the tick-borne relapsing fever spirochete Borrelia hermsii by using a species-specific monoclonal antibody. Journal of Clinical Microbiology. 1992;30(4):790-5.
1328. Rath PM, Rogler G, Schonberg A, Pohle HD, Fehrenbach FJ. Relapsing fever and its serological discrimination from lyme borreliosis. Infection. 1992;20(5):283-6.
1329. Picken RN. Polymerase chain reaction primers and probes derived from flagellin gene sequences for specific detection of the agents of Lyme disease and North American relapsing fever. Journal of Clinical Microbiology. 1992;30(1):99-114.
1330. Huminer D, Hardy B, Pitlik S. Hydrochlorothiazide-induced relapsing fever. Isr J Med Sci. 1992;28(12):880-2.
1331. Taylor JP, Moore GM, Cheek JE. OUTBREAK OF RELAPSING FEVER MASQUERADING AS LYME BORRELIOSIS. Program and Abstracts of the Interscience Conference on Antimicrobial Agents and Chemotherapy. 1991;31:152.
1332. Burman N, Bergstrom S, Restrepo BI. ANTIGENIC VARIATION IN RELAPSING FEVER BORRELIA. Abstracts of the General Meeting of the American Society for Microbiology. 1991;91:66.

1333. Barbour AG. Molecular biology of antigenic variation in Lyme borreliosis and relapsing fever: A comparative analysis. Scandinavian Journal of Infectious Diseases, Supplement. 1991;22(77):88-93.
1334. Anonymous. UTHSCSA scientists study relapsing fever. Texas medicine. 1991;87(10):38.
1335. Matton P, Van Melckebeke H. Bovine borreliosis: Comparison of simple methods for detection of the spirochaete in the blood. Tropical Animal Health and Production. 1990;22(3):147-52.
1336. Barbour AG. Antigenic variation of a relapsing fever Borrelia species. Annual Review of Microbiology. 1990;44:155-71.
1337. Barbour AG. MULTIPHASIC ANTIGENIC VARIATION IN THE BACTERIUM THAT CAUSES RELAPSING FEVER1990. 183-200 p.
1338. Vasil'eva IS, Ershova AS. Effect of Borrelia--the causative agent of tick-borne relapsing fever--on the vector. Meditsinskaia parazitologiia i parazitarnye bolezni. 1989(3):16-20.
1339. Logan JS. Trench fever in Belfast, and the nature of the 'relapsing fevers' in the United Kingdom in the nineteenth century. The Ulster medical journal. 1989;58(1):83-8.
1340. Barbour A. ANTIGENIC VARIATION IN RELAPSING FEVER BORRELIA SPECIES GENETICS ASPECTS1989. 783-90 p.
1341. Warrell DA. RELAPSING FEVERS1988. 90-2 p.
1342. Nassif X, Dupont B, Fleury J, Lapresle C. Ceftriaxone in relapsing fever. Lancet. 1988;2(8607):394.
1343. Gutman LT. THE SPIROCHETES1988. 555-71 p.
1344. Burgdorfer W. CURRENT STATUS OF TICK-BORNE DISEASES IN NORTH AMERICA. Journal of the American Veterinary Medical Association. 1988;192(12):1769.
1345. Rawlings JA, Fournier PV, Teltow GJ. Isolation of Borrelia spirochetes from patients in Texas. J Clin Microbiol. 1987;25(7):1148-50.
1346. Melkert PW. Prognostic value of the Borrelia-index in relapsing fever. East African medical journal. 1987;64(4):284-6.
1347. Barbour AG. Immunobiology of relapsing fever. Contributions to microbiology and immunology. 1987;8:125-37.
1348. Plasterk RHA, Simon MI, Barbour AG. MOLECULAR BASIS FOR ANTIGENIC VARIATION IN A RELAPSING FEVER BORRELIA. Birkbeck, T H And C W Penn. Special Publications of the Society for General Microbiology1986. p. 127-46.
1349. Krampitz HE. In vivo isolation and maintenance of some wild strains of European hard tick spirochetes in mammalian and arthropod hosts: A parasitologist's view. Zentralblatt fur Bakteriologie Mikrobiologie und Hygiene - Abt 1 Orig A. 1986;263(1-2):21-8.
1350. Kehl KS, Farmer SG, Komorowski RA, Knox KK. Antigenic variation among Borrelia spp. in relapsing fever. Infect Immun. 1986;54(3):899-902.
1351. Barbour AG. Cultivation of Borrelia: a historical overview. Zentralbl Bakteriol Mikrobiol Hyg A. 1986;263(1-2):11-4.
1352. Barbour AG. MOLECULAR BASIS FOR ANTIGENIC VARIATION IN A RELAPSING FEVER BORRELIA-SP. Journal of Cellular Biochemistry Supplement. 1986(10 PART A):116.
1353. Meier JT, Simon MI, Barbour AG. Antigenic variation is associated with DNA rearrangements in a relapsing fever Borrelia. Cell. 1985;41(2):403-9.
1354. Kehl KS. Relapsing fever: Role of borrelial antigens. Clinical Microbiology Newsletter. 1985;7(4):25-7.
1355. Carter GD, Alaghband-Zadeh J, Wright DJ, Teklu B, Habte-Michael A. Thyroid function tests in relapsing fever. Archives of internal medicine. 1985;145(3):576-7.

1356. Barbour AG. CLONAL POLYMORPHISM OF SURFACE ANTIGENS IN A RELAPSING FEVER BORRELIA SPECIES. Jackson, G G And H Thomas. Bayer-Symposium1985. p. 235-45.
1357. Gaber MS, Aboul-Nasr AE. Borrelia crocidurae localization and transmission in Ornithodoros erraticus and O. savignyi. Parasitology. 1984;88(3):403-13.
1358. Kryuchechnikov VN, Korenberg EI, Shcherbakov SV. EXPERIENCE IN DETECTING STRAIN DIFFERENCES OF THE CAUSAL AGENT OF TICK RECURRENT FEVER. Meditsinskaya Parazitologiya i Parazitarnye Bolezni. 1982;51(4):74-9.
1359. Gaber MS, Khalil GM, Hoogstraal H. Borrelia crocidurae: Venereal transfer in Egyptian Ornithodoros erraticus ticks. Experimental Parasitology. 1982;54(2):182-4.
1360. Ackermann R. Tick-borne infectious diseases. [German]. H+G Zeitschrift fur Hautkrankheiten. 1981;56(23):1489-96.
1361. Lane RS, Murray RA. SURVEILLANCE OF TICK-BORNE DISEASES OF ACTUAL OR POTENTIAL PUBLIC HEALTH IMPORTANCE IN CALIFORNIA USA. Bulletin of the Society of Vector Ecologists. 1980;5:57-62.
1362. Chin J. DISEASES TRANSMITTED PRIMARILY BY ARTHROPOD VECTORS BACTERIAL INFECTIONS RELAPSING FEVER1980. P367-402 p.
1363. Rodhain F. Borreliosis, or recurrent fever. Concours Medical. 1977;99(1-2):41-6.
1364. Newson HD. Arthropod problems in recreation areas. Annu Rev Entomol. 1977;22:333-53.
1365. Diab FM, Soliman ZR. An experimental study of Borrelia anserina in four species of Argas ticks - 1. Spirochete localization and densities. Zeitschrift für Parasitenkunde. 1977;53(2):201-12.
1366. Pegram RG. Ticks (Acarina, Ixodoidea) of the northern regions of the Somali Democratic Republic. Bulletin of Entomological Research. 1976;66(2):345-63.
1367. Szalay GC. Tick-borne Diseases. West J Med. 1974;121(4):328.
1368. Sutton RNP, Templer MJ. Some thoughts on tick borne fevers in the Middle East. Journal of the Royal Army Medical Corps. 1973;119(3):157-61.
1369. Smibert RM, Johnson RC. Spirochaetales, a review. Critical Reviews in Microbiology. 1973;2(4):491-552.
1370. Burgdorfer W, Mavros AJ. Susceptibility of Various Species of Rodents to the Relapsing Fever Spirochete, Borrelia hermsii. Infect Immun. 1970;2(3):256-9.
1371. Warrell DA, Pope H, Bryceson AD, Parry EH, Perine LL. Respiratory and acid-base changes during the crises in relapsing fever. Clinical science. 1969;37(2):567.
1372. Chohan IS. Tick-borne relapsing fever in Kashmir: mice inoculation--a diagnostic method of choice. Indian journal of pathology & bacteriology. 1967;10(3):289-94.
1373. Ranque J. On the possibility of transmission of relapsing fever by blood transfusion. Transfusion. 1963;6:163-4.
1374. Banwell JG, Kibukamusoke JW. Haemolytic anaemia and relapsing fever. East African medical journal. 1963;40:124-6.
1375. McRobert GR. Ticks as Vectors. Br Med J. 1962;2(5311):1034-5.
1376. McCullough F, Friis-Hansen B. A parasitological survey in three selected communities in Luapula Province, Northern Rhodesia. Bull World Health Organ. 1961;24(2):213-9.
1377. Geigy R, Sarasin G. Environment-related factors in habitat and behavior of the pathogen of relapsing fever Borrelia duttonii. Acta tropica. 1961;18:359-65.
1378. Larribaud J. Apropos of a case of tick-borne relapsing fever. Bulletin mensuel - Société de médecine militaire française. 1960;54:170-2.
1379. Heisch RB, Harvey AE. Is Ornithodoros savignyi (Audouin) a vector of relapsing fever in Africa? Annals of tropical medicine and parasitology. 1960;54:205-7.

1380. Hatem J, Chidiac J. Inquiry apropos of a case of recurrent fever. Revue médicale du Moyen-Orient. 1960;17:504-5.
1381. Chippaux C. [Geographical distribution of exotic diseases. IX. Recurrent fevers]. Concours Med. 1959;81(11):1179-80 passim.
1382. Abdussalam M. Significance of ecological studies of wild animal reservoirs of zoonoses. Bull World Health Organ. 1959;21(2):179-86.
1383. Symposium on Arthropod-Borne and Other Forms of Encephalitis in the Tropics. Proc R Soc Med. 1959;52(3):193-202.
1384. International Sanitary Conventions and Regulations, plague and relapsing fever. Bull World Health Organ. 1959;20(4):735-48.
1385. Zumpt F. A Preliminary Survey of the Distribution and Host-specificity of Ticks (Ixodoidea) in the Bechuanaland Protectorate. Bulletin of Entomological Research. 1958;49(2):201-23.
1386. Walton GA. Studies on Ornithodorus moubata Murray (Argasidae) in East Africa. I. Observations on distribution, food and climatic requirements in relation to relapsing fever as evidence of biological variation. East African medical journal. 1958;35(2):57-84.
1387. Mareschal P, Noblet J. Relapsing fever in the treatment of general paralysis. Annales médico-psychologiques. 1958;116 , Vol 2(2):268-78.
1388. Martin JD. Zoonoses in the south. Public Health Rep. 1957;72(3):210-6.
1389. Addamiano L, Babudieri B. Crossed immunity among various strains of relapsing fever spirochete. Rendiconti - Istituto superiore di sanità. 1957;20(4):440-8.
1390. Addamiano L, Babudieri B. Research on spirochaetal strains isolated in Jordan. Bull World Health Organ. 1957;17(3):483-5.
1391. Varma MG. Comparative studies on the transmission of two strains of Spirochaeta duttoni by Ornithodoros moubata and of S. turicatae by O. turicata. Annals of tropical medicine and parasitology. 1956;50(1):1-17.
1392. Varma MG. Infections of Ornithodoros ticks with relapsing fever spirochaetes, and the mechanisms of their transmission. Annals of tropical medicine and parasitology. 1956;50(1):18-31.
1393. Otto H. Clinical aspects and modern treatment of recurrent fever; personal experiences. Zeitschrift für die gesamte innere Medizin und ihre Grenzgebiete. 1956;11(20):946-51.
1394. Heisch RB. Zoonoses as a study in ecology; with special reference to plague, relapsing fever, and leishmaniasis. Br Med J. 1956;2(4994):669-73.
1395. Davis GE, Burgdorfer W. Relapsing fever spirochetes: an aberrant strain of Borrelia parkeri from Oregon. Experimental parasitology. 1955;4(2):100-6.
1396. Colas-Belcour J, Vervent G. Transmission of various relapsing fever causing Spirochaetae by a Sudanese strain of Ornithodoros erraticus Lucas (O.E. var. sonrai J. Sautet, H. Marneffe and M. Witkowsky). Bulletin de la Société de pathologie exotique et de ses filiales. 1955;48(5):747-57.
1397. Ozsan K, Akyay N. Relapsing fever in Turkey; presence in the South (Turko-Syrian border) of Ornithodorus erraticus infected with a spirochete of the Crocidurae group. Bulletin de la Société de pathologie exotique et de ses filiales. 1954;47(4):501-3.
1398. Burgdorfer W, Davis GE. Experimental infection of the African relapsing fever tick, Ornithodoros moubata (Murray), with Borrelia latychevi (Sofiev). The Journal of parasitology. 1954;40(4):456-60.
1399. Lieb FL. Identification of Relapsing Fever Spirochaetes. Ind Med Gaz. 1953;88(4):192-4.

1400. Holmes JWE. Tick Control and Relapsing Fever. The Journal of the Royal Society for the Promotion of Health. 1953;73(3):262-5.
1401. Geigy R. The tick as reservoir and carrier of African relapsing fever. Schweizerische Zeitschrift für Pathologie und Bakteriologie Revue suisse de pathologie et de bactériologie. 1953;16(5):821-3.
1402. Davis GE, Mazzotti L. The non-transmission of the relapsing fever spirochete, Borrelia dugesii (Mazzotti, 1949) by the argasid tick Ornithodoros turicata (Dugès, 1876). The Journal of parasitology. 1953;39(6):663-6.
1403. Bell S. Predisposing factors in tick-borne relapsing fever in Meru district, Kenya. Transactions of the Royal Society of Tropical Medicine and Hygiene. 1953;47(4):309-17.
1404. Teichler GH. African relapsing fever. Zeitschrift für Tropenmedizin und Parasitologie. 1952;4(1):121-4.
1405. Sergent E. The long duration of the metacritical latent infection and the correlative warning in Spanish North African relapsing fever. Comptes rendus hebdomadaires des séances de l'Académie des sciences. 1952;234(5):494-6.
1406. Monnerot-Dumaine M. [Recurrent fevers]. Presse Med. 1952;60(4):69-70.
1407. Davis GE. The relapsing fevers: Tick-spirochete specificity studies. Experimental Parasitology. 1952;1(4):406-10.
1408. Annecke S, Quin P. Relapsing fever in South Africa; its control. South African medical journal = Suid-Afrikaanse tydskrif vir geneeskunde. 1952;26(22):455-60.
1409. Longanecker DS. Laboratory and field studies on the biology of the relapsing fever tick vector (Ornithodoros hermsi Wheeler) in the high mountains of California. The American journal of tropical medicine and hygiene. 1951;31(3):373-80.
1410. Burgdorfer W. Analysis of the infection course in Ornithodorus moubata (Murray) and natural transmission of Spirochaeta duttoni. Acta tropica. 1951;8(3):193-262.
1411. Knowles FA, Terry ED. Prevention of tick-borne relapsing fever. East African medical journal. 1950;27(2):88-93.
1412. Cossar B, Antonini F. Broncho-pleuro-pulmonary complications in cosmopolitan recurrent fever in Ethiopia. Minerva medica. 1950;41(55):889-92.
1413. Valdivieso Conde L. [Clinical study of recurrent fever]. Med Cir Guerra. 1949;11(9):379-88.
1414. Staemmler M. [Relapsing Fever Anatomical Findings]. Frankf Z Pathol. 1949;60(3-4):560-92, illust.
1415. Royo Montanes M. [Recurrent fever cogenita]. Rev Esp Pediatr. 1949;5(1):1-16.
1416. Rao KNA, Kalra SL. Tick-borne relapsing fever in Kashmir. Current science. 1949;18(7):249.
1417. Moorman WW, Kennamer SR. Relapsing fever; report of two cases. Texas state journal of medicine. 1949;45(11):771-3.
1418. Konitzer L. Relapsing fever in ambulatory practice. Acta medica Orientalia. 1949;8(3-4):48-53.
1419. Wolstenholme B, Gear JH. A complement fixation test for the diagnosis of relapsing fever. Transactions of the Royal Society of Tropical Medicine and Hygiene. 1948;41(4):513-7.
1420. Pastor Botija F. [Considerations about the ocular sequelae in recurrent fever; epidemic of the years 1945-1946 in the Spanish protectorate zone in Morocco]. Med Colon. 1948;11(2):86-97.
1421. Lerace F. [The ticks that transmit recurrent fevers]. Acta Med Ital Mal Infett Parassit. 1948;3(3):73-5.

1422. Herms WB. Ticks, Acarina-Ixodoidea, in the causation of animal disorders and as vectors of disease, with some observations on relapsing fever in California. Abstracts International Congress on Tropical Medicine and Malaria (4th : 1948 : Washington, D C). 1948;56(4 Congr):134.
1423. Babudieri B, Bocciarelli D. Electron-microscope studies on relapsing fever spirochaetes. The Journal of hygiene. 1948;46(4):438.
1424. Smith A. A case of relapsing fever. Proceedings Transvaal Mine Medical Officers' Association. 1947;26(291):129.
1425. Sabalette R, Dominguez M, Iglesias R. [Clinical, hematological and therapeutic study of a recurrent fever outbreak]. Med Colon. 1947;9(3):207-21.
1426. O'Keefe JP. A case for diagnosis [relapsing fever?]. Proceedings Transvaal Mine Medical Officers' Association. 1947;26(291):128.
1427. Nunez B, Jeghers H. [Relapsing fever with hemorrhage into the skin, spleen, peritoneal cavity, and wall of bowel and congestion of the pia-arachnoid]. The Medical annals of the District of Columbia. 1947;16(5):250-4.
1428. Mohteda SN. Avian spirochetosis. Indian Vet J. 1947;23(5):395-7.
1429. Merskey C. Relapsing fever in Cullinan (Transvaal) with a short reference to penicillin therapy. Clinical proceedings. 1947;6(4):113-24.
1430. Jepson WF. Economic control of the relapsing fever tick in African houses. Nature. 1947;160(4077):874.
1431. Boiron H. [Contribution to the diagnosis and treatment of the recurrent fever in Spirochaeta duttoni in the agglomeration of Dakar]. Ann Inst Pasteur (Paris). 1947;73(1):49-57.
1432. Binaghi G. [Recurrent fever in Cagliari]. Acta Med Ital Mal Infett Parassit. 1947;2(9):293.
1433. Aubin H, Gachkel, et al. [Mental disorders in the relapsing fever]. Alger Medicale. 1947;50(5):408-16.
1434. Schuhardt VT, Hemphill EC. Brain involvement as a possible cause of relapse after treatment in spirochetal relapsing fever. Science (New York, NY). 1946;103(2675):422.
1435. Randolph NM. DDT to control the relapsing fever tick. Journal of economic entomology. 1946;39:396.
1436. RELAPSING fever. The Journal of the Royal Faculty of Medicine of Iraq. 1946;10(3):32.
1437. Lofgren R, Soule MH. The Effect of Low Temperature on the Spirochetes of Relapsing Fever: II. The Structure and Motility of Spirochaeta novyi. J Bacteriol. 1945;50(3):313-21.
1438. Lebedeva NI. [Role of the nurse in control of relapsing fever]. Meditsinskaia sestra. 1945(1-2):13-5.
1439. Hynd RS. The construction of Army huts to limit infestation by Ornithodoros moubata (relapsing fever tick). East African medical journal. 1945;22:337-42.
1440. Stein GJ. THE SEROLOGICAL DIAGNOSIS OF RELAPSING FEVER. J Exp Med. 1944;79(1):115-28.
1441. Pavlovsky EN, Cheskis AF. Susceptibility of the domestic pig to Central Asiatic tick relapsing fever spirochaete. Comptes Rendus De L Academie Des Sciences De L Urss. 1943;38:55-6.
1442. Pavlovsky EN. Dyromys nitedulus Pall as a possible reservoir of the virus of tick relapsing fever. Comptes Rendus De L Academie Des Sciences De L Urss. 1943;39:286-8.
1443. Pavlovshy EN. A new vector of the tick relapsing fever - Ornithodo nereensis pavl in Turkmenia. Comptes Rendus De L Academie Des Sciences De L Urss. 1941;31:408-10.

1444. Davis GE. ORNITHODOROS TURICATA: THE MALE; FEEDING AND COPULATION HABITS, FERTILITY, SPAN OF LIFE, AND THE TRANSMISSION OF RELAPSING FEVER SPIROCHETES. Public Health Reports. 1941;56(36):1799-802.
1445. Davis GE. RELAPSING FEVER: THE GUINEA PIG AS AN EXPERIMENTAL ANIMAL IN THE STUDY OF ORNITHODOROS TURICATA, O. PARKERI, AND O. HERMSI STRAINS OF SPIROCHETES. Public Health Reports. 1939;54(38):1721-7.
1446. Wynns HL, Beck MD. Epidemiological Studies on Relapsing Fever in California *. Am J Public Health Nations Health. 1935;25(3):270-6.
1447. Latham DV. Undulant Fever and its Synonyms. Br Med J. 1935;1(3885):1294.
1448. Hearle E. VECTORS OF RELAPSING FEVER IN RELATION TO AN OUTBREAK OF THE DISEASE IN BRITISH COLUMBIA. Can Med Assoc J. 1934;30(5):494-7.
1449. Coleman GE. Relapsing Fever Problem of California *. Am J Public Health Nations Health. 1934;24(10):1056-61.
1450. Megaw J. A Note on Professor Nicolle's Views on the Typhus and Relapsing Fevers. Ind Med Gaz. 1933;68(8):462-4.
1451. Porter GS, Beck MD, Stevens IM. Relapsing Fever in California. Am J Public Health Nations Health. 1932;22(11):1136-40.
1452. Relapsing Fever Therapy in General Paralysis: Clinical Notes on a Case of Paresis treated with African Tick Fever. Journal of Mental Science. 1924;70(288):89-92.
1453. Relapsing Fever in Panama. Ind Med Gaz. 1923;58(3):123.
1454. Woodcock HM. TICKS AND RELAPSING FEVER. Br Med J. 1920;1(3087):310-1.
1455. Willcox WH. Typhus and Relapsing Fever in Mesopotamia and Northern Persia 1. Proc R Soc Med. 1920;13(Med Sect):59-81.
1456. Treves WW. TICKS AND RELAPSING FEVER. Br Med J. 1920;1(3085):235-6.
1457. Mackenzie JW. TICKS AND RELAPSING FEVER. Br Med J. 1920;1(3084):200.
1458. Dunlop JL. TICKS AND RELAPSING FEVER. Br Med J. 1920;1(3082):132.
1459. Drake-Brockman RE. TICKS AND RELAPSING FEVER. Br Med J. 1920;1(3086):273.
1460. Balfour AK. Ticks and relapsing fever. Bmj-British Medical Journal. 1920;1920:97-8.
1461. Browse GV. A Special Type of Recurrent Fever Due to a Spirochæta. Ind Med Gaz. 1912;47(10):387-90.
1462. Kenrick WH. Spirochæte Fever. Ind Med Gaz. 1908;43(4):138-9.
1463. Cox WH. Relapsing Fever in the 109th Infantry. Ind Med Gaz. 1906;41(4):138.
1464. Nuttall GHF. Ticks and Tick-Transmitted Diseases. Trans Epidemiol Soc Lond. 1905;24:12-32.
1465. Shah JS, Liu S, inventors; ID-FISH TECHNOLOGY INC (IDFI-Non-standard) ID-FISH TECHNOLOGIES INC (IDFI-Non-standard) ID-FISH TECHNOLOGY INC (IDFI-Non-standard) ID-FISH TECHNOLOGIES INC (IDFI-Non-standard), assignee. Composition for tick-borne relapsing fever comprises one or more labeled and/or tagged and/or bound amino acid sequences patent WO2019173569-A1; US2019277847-A1; US10718767-B2; CA3092156-A1; US2020348301-A1.
1466. Norris SJ, inventor; UNIV TEXAS SYSTEM (TEXA-C) UNIV TEXAS SYSTEM (TEXA-C) NORRIS S J (NORR-Individual) UNIV TEXAS SYSTEM (TEXA-C) UNIV TEXAS SYSTEM (TEXA-C) UNIV TEXAS SYSTEM (TEXA-C), assignee. New isolated nucleic acid molecules encoding Variable Major Protein-like polypeptides of Borrelia, useful for diagnosing, preventing or treating Borrelia infections such as Lyme disease or relapsing fever patent WO2004058181-A2; AU2003299872-A1; EP1572714-A2; AU2003299872-A8; US2006240035-A1; US7847084-B2; EP2292762-A2; US2011124047-A1; US8076470-B2; WO2004058181-A3; US2012088289-A1; US8283458-B2; EP2292762-A3; US2013102057-A1; US8722871-B2; US2014220605-A1;

US9115193-B2; US2016102124-A1; US9670254-B2; EP1572714-A4; US2018022782-A1; US10323070-B2; US2020040043-A1; US10759833-B2.
1467. Lewis K, Imai Y, Wu XW, Donofrio A, inventors; Univ Northeastern (Unen-C), assignee. Treating disease associated with a spirochete comprising e.g. leptospirosis, lyme disease, relapsing fever, syphilis, yaws, gingivitis and periodontitis in animal, comprises administering substituted (E)-2-methyl-3-phenyl-propenal compounds patent WO2020041179-A1.
1468. Driebe E, Keim P, Engelthaler D, Bowers J, Nieto N, inventors; Translational Genomics Res Inst (City-C) Univ Arizona State (Uyas-C) Translational Genomics Res Inst (City-C) Univ Arizona State (Uyas-C), assignee. Detecting Borrelia species causing Lyme disease or tick-borne relapsing fever (TBRF) within sample from subject, comprises subjecting DNA and/or RNA to PCR amplification reaction using primer pairs, and analyzing amplification products patent WO2017139715-A1; US2019040455-A1.
1469. Cutler S, Abdissa A, Adamu H, Tolosa T, Gashaw A. Borrelia in Ethiopian ticks. Ticks and Tick borne Diseases.
1470. Clokie RJM, Shan J, Teulieres LC, inventors; UNIV LEICESTER (CEST-C) PHELIX RES & DEV LTD (PHEL-Non-standard) PHELIX RES & DEV LTD (PHEL-Non-standard), assignee. Determining presence or absence of Borrelia burgdorferi sensu lato or Relapsing Fever Borrelia in sample involves detecting presence or absence of phage specific for Borrelia burgdorferi sensu lato or Relapsing Fever Borrelia in sample patent WO2018083491-A1; CN110168110-A; US2019276877-A1; EP3535412-A1.
1471. Sánchez RST, Santodomingo AMS, Muñoz-Leal S, Silva-De la Fuente MC, Llanos-Soto S, Salas LM, et al. Rodents as potential reservoirs for Borrelia spp. In northern Chile. Revista Brasileira de Parasitologia Veterinaria. 2020;29(2):1-10.
1472. Hama HO, Barbieri R, Guirou J, Chenal T, Mayer A, Ardagna Y, et al. An outbreak of relapsing fever unmasked by microbial paleoserology, 16th century, France. American Journal of Physical Anthropology. 2020;173(4):784-9.
1473. Delaney SL, Murray LA, Aasen CE, Bennett CE, Brown E, Fallon BA. Table_1_Borrelia miyamotoi Serology in a Clinical Population With Persistent Symptoms and Suspected Tick-Borne Illness.DOCX. Figshare; 2020.
1474. Cantero AP, de Pedro IP, Tellez SM, Muriel CC, Martinez LFC, Huelgas RG. Borrelia hispanica as a cause of recurrent fever. Medicina Clinica. 2020;154(9):380-.
1475. Ahmed Yahia S, Faibis F, Benmoussa M, Lantohasina N, Dupont A, Ait Abdesselam T. Tick-borne relapsing fever: An unrecognized cause of fever in travellers. Revue de Medecine Interne. 2020;41(6):418-20.
1476. Lopez JE, Krishnavajhala A, Garcia MN, Bermudez S. Tick-Borne Relapsing Fever Spirochetes in the Americas (vol 3, 16, 2016). Veterinary Sciences. 2019;6(4):98-.
1477. Hashavya S. Pediatric borreliosis is not just small borreliosis: Similarities and differences in tick borne relapsing fever in children and adults. European Journal of Pediatrics. 2019;178(11):1710.
1478. EAP 2019 Congress and Master Course. Eur J Pediatr. 2019;178(11):1613-800.
1479. Kassiri H, Dostifar K, Karimi M. 112: SITUATION OF RELAPSING FEVER BORRELIOSIS IN BIJAR, IRAN. BMJ Open. 2017;7(Suppl 1).
1480. Strachinaru DIC, Cambier J, Kandet-Yattara H, Konopnicki D. Relapsing fever in asylum seekers from Somalia arriving in Belgium in August 2015. Acta Clinica Belgica. 2016;71(5):353-5.
1481. Lopez JE, Krishnavajhala A, Garcia MN, Bermudez S. Erratum: Tick-Borne Relapsing Fever Spirochetes in the Americas. [Vet. Sci. (2016), 3, (16)] doi: 10.3390/vetsci3030016. Veterinary Sciences. 2016;6(4).

1482. Sin J, Hartill-Dadson K, Garvey MB, Hussain Z, Ghaffar H. An unexpected diagnosis encountered in the hematology laboratory : Peripheral blood findings in a patient with relapsing fever. International Journal of Laboratory Hematology. 2013;35:105.
1483. Mediannikov O, Cutler SJ, Drancourt M, Henry M, Elbir H, Socolovschi C, et al. Multiplex Real-Time PCR Diagnostic of Relapsing Fevers in Africa. Figshare; 2013.
1484. Diatta G. Epidemiological and geographic distribution of tick-borne relapsing fever borreliosis in west and North Africa. American Journal of Tropical Medicine and Hygiene. 2013;89(5):147.
1485. Arnathau C, Elguero E, Trape J-F, Pugnolle F, Bitam I, Vial L, et al. The Epidemiology and Geographic Distribution of Relapsing Fever Borreliosis in West and North Africa, with a Review of the Ornithodoros erraticus Complex (Acari: Ixodida). Figshare; 2013.
1486. Cruz NS, Mayoral PV. Borreliosis: Recurrent fever due to spirochetes. Case report. Boletin Medico del Hospital Infantil de Mexico. 2012;69(2):114-8.
1487. Hasin T, Davidovitch N, Cohen D. Doxycycline for the prevention of tick-borne relapsing fever - Reply. New England Journal of Medicine. 2006;355(15):1614-5.
1488. Anonymous. Tick-borne relapsing fever: What you should know. American Family Physician. 2005;72(10):2046-1--2.
1489. Cousins JK, Bono MJ. Clinician's guide to tick-borne disease...first of two parts. Emergency Medicine (00136654). 2003;35(6):42-50.
1490. Parola P, Raoult D. Ticks and Tickborne Bacterial Diseases in Humans: An Emerging Infectious Threat. Science Translational Medicine. 2001;3(91).
1491. BANERJEE SN. Spirochete hits Canada. Cmaj. 1999;160(2):177.
1492. Marti Ras N, Lascola B, Postic D, Cutler SJ, Rodhain F, Baranton G, et al. Phylogenesis of relapsing fever Borrelia spp. International Journal of Systematic Bacteriology. 1996;46(4):859-65.
1493. Aoki SK, Holland PV. Blood transfusion-associated relapsing fever (I: Reply). Transfusion. 1990;30(4):380-1.
1494. López-Cortés L, Lozano de León F, Gómez-Mateos JM, Sánchez-Porto A, Obrador C. Tick-borne relapsing fever in intravenous drug abusers. The Journal of infectious diseases. 1989;159(4):804.
1495. Vasil'eva IS, Sharipov MK, Ershova AS, Mansurov AA, Mukhitdinov AG. Current status of the foci and morbidity of tick-borne relapsing fever in Uzbekistan. Parazitologiia. 1984;18(1):10-4.
1496. Goubau PF, Munyangeyo C. [Congenital relapsing fever caused by Borrelia duttoni. Apropos of a case in Rwanda]. Ann Soc Belg Med Trop. 1983;63(4):367-9.
1497. Anonymous. Relapsing fever, 1973, in Grand Canyon National Park. Clinical pediatrics. 1974;13(5):408 passim.
1498. Babudieri B. [Electron microscope observations on the spirochetes of recurrent fever]. Rend Ist Sup Sanit. 1948;11(4):1067-70.
1499. Hindle E. AFRICAN TICK FEVER. Br Med J. 1931;1(3677):1140.
1500. Willcox WH. Typhus and Relapsing Fever in Mesopotamia and Northern Persia 1 (duplicate). Proc R Soc Med. 1920;13(Med Sect):59-81.
1501. Nesga D, Addissie A. Relapsing fever cases surveillance data analysis from 2012-2016 in Addis Ketema Sub-City, Addis Ababa, Ethiopia. Transactions of the Royal Society of Tropical Medicine and Hygiene. 2019;113:S99.
1502. Conover MR, Vail RM, Conover MR, Vail RM. Tick-Borne Relapsing Fever2015. 213-25 p.

1503. Koenen F, Pascucci I, Jaenson TGT, Madder M, de Sousa R, Estrada-Pena A, et al. Tick-borne Infections (Including Zoonoses) in Europe and the Mediterranean Basin2013. 33-75 p.
1504. Hussein H, Showler A, Tan DHS. Cabin fever: A case of borrelia hermsii in pregnancy. Canadian Journal of Infectious Diseases and Medical Microbiology. 2013;24:36B.
1505. EstradaPena A, Farkas R, Jaenson TGT, Koenen F, Madder M, Pascucci I, et al. Ticks and Tick-borne Diseases: Geographical Distribution and Control Strategies in the Euro-Asia Region2013.
1506. Christian TE. Tick-borne diseases. Koda-Kimble and Young's Applied Therapeutics: The Clinical Use of Drugs2013. p. 1846-62.
1507. Warrell DA. Relapsing fevers. Infectious Diseases: Third Edition. 22010. p. 1243-6.
1508. Lipford EH, Lackey PC, Tolba LM, Sautter RL. A Case of Borrelia sp Infection following Travel to Guatemala and Belize. Abstracts of the General Meeting of the American Society for Microbiology. 2009;109.
1509. Anderson JM, Lopez J, Sogoba N, Schrumpf ME, Raffel SJ, Schwan TG. Tick borne relapsing fever in Mali. American Journal of Tropical Medicine and Hygiene. 2009;81(5):4.
1510. Martínez-Baylach J, García Rodríguez L, Cabot Dalmau A, Sauca G, Díez R. Relapsing fever due to borreliosis. Acta Pediatrica Espanola. 2007;65(1):29-31.
1511. Weiss J. Tick-borne relapsing fever: Postexposure prophylaxis with doxycycline is effective. [German]. Deutsche Medizinische Wochenschrift. 2006;131(36):1919.
1512. Barbour AG. Relapsing Fever and Other Borrelia Diseases. Tropical Infectious Diseases. 12006. p. 499-510.
1513. Anonymous. Doxycycline prophylaxis effective for tick-borne relapsing fever. South African Family Practice. 2006;48(8):12.
1514. Guggenheim JN, Haverkamp AD. Tick-borne relapsing fever during pregnancy: A case report. Journal of Reproductive Medicine for the Obstetrician and Gynecologist. 2005;50(9):727-9.
1515. Hansen K, Pfister HW. Lyme Neuroborreliosis, Leptospirosis, and Relapsing Fever. Neurological Disorders: Course and Treatment: Second Edition2003. p. 583-93.
1516. Gao L, Oliver JH, Jr., Lin T. Description of a novel relapsing fever Borrelia isolate from a Dermacentor variabilis tick feeding on a coyote. Abstracts of the General Meeting of the American Society for Microbiology. 2003;103:D-148.
1517. Barbour AG. Borrelia infections: Relapsing fever and lyme disease. Cunningham MW, Fujinami RS, editors2000. 57-70 p.
1518. Billings AN, Rawlings JA, Walker DH. Tick-borne diseases in Texas: a 10-year retrospective examination of cases. Texas medicine. 1998;94(12):66-76.
1519. Sánchez-Yebra W, Díaz E, Molina P, Sedeño J, Giner P, Del Mar Vitutia M, et al. Tick-Borne recurrent fever. Description of 5 cases. Enfermedades Infecciosas y Microbiologia Clinica. 1997;15(2):78-81.
1520. Sánchez Porto A, Crespo Anguita M, Iglesias C, Lucio Villegas MC, Duarte C. [Outbreak of borreliosis. Apropos of 7 cases]. Enferm Infecc Microbiol Clin. 1991;9(2):125.
1521. Perine PL. RELAPSING FEVER1991. 666-7 p.
1522. Paul WS, Scott-Wright A, Maupin G, Craven R, Dennis D. TICK-BORNE RELAPSING FEVER GRAND CANYON COLORADO USA 1990. Program and Abstracts of the Interscience Conference on Antimicrobial Agents and Chemotherapy. 1991;31:151.
1523. Melkert PW, Stel HV. Neonatal Borrelia infections (relapsing fever): report of 5 cases and review of the literature. East African medical journal. 1991;68(12):999-1005.
1524. Melkert PW. Mortality in high risk patients with tick-borne relapsing fever analysed by the Borrelia-index. East African medical journal. 1991;68(11):875-9.

1525. Grossman M. RELAPSING FEVER1991. 600 p.
1526. Kaufmann M, Frei A. A case from practice (194). Relapsing fever. Schweizerische Rundschau für Medizin Praxis = Revue suisse de médecine Praxis. 1990;79(43):1311-3.
1527. Johnson WDJ. BORRELIA SPECIES RELAPSING FEVER1990. 1816-9 p.
1528. Butler T. Relapsing fever1990. 914-8 p.
1529. Peters VB, Schussheim A, Rubin LG. Fever of unknown origin in an adolescent girl due to Borrelia infection (relapsing fever). Children's Hospital Quarterly. 1989;1(4):353-7.
1530. Johnson RC. RELAPSING FEVER1989. 1596-8 p.
1531. Goubau P. [Relapsing fever seen in Europe: epidemiology, diagnosis, course and treatment]. Rev Prat. 1989;39(15):1304-7.
1532. Melkert PW. Fatal-Jarisch Herxheimer reaction in a case of relapsing fever misdiagnosed as lobar pneumonia. Trop Geogr Med. 1987;39(1):92-3.
1533. Leon de Lope M, Leal Luna A, Galvez Acebal J. Borreliosis: A report of 4 cases. Medicina Clinica. 1985;85(3):111-3.
1534. Huber M, Eichenlaub D. [Relapsing fever in a tourist to Africa]. MMW Munch Med Wochenschr. 1984;126(7):178-80.
1535. Goubau PF. Relapsing fevers. A review. Annales de la Société belge de médecine tropicale. 1984;64(4):335-64.
1536. Goshen J, Koren A, Ben-Ami M, Katzuni E. Endemic relapsing fever in children. Harefuah. 1984;106(2):65-8+104.
1537. Coene J. Relapsing fever in Sichili. Medical journal of Zambia. 1984;18(1):10-4.
1538. Alkan M, Kaplan H, Baumgarten A. Hypoglycemia, jaundice and shock complicating relapsing fever. Harefuah. 1984;106(11):508-9+39.
1539. Yagupsky P, Moses S, Kaplan E. NEO NATAL RELAPSING FEVER. Israel Journal of Medical Sciences. 1983;19(11):1037.
1540. Janssens PG. Tick-borne relapsing fever in Central Africa. [French]. Annales de la Societe belge de medecine tropicale. 1983;63(4):277-80.
1541. Goubau PFJ, Munyangeyo C. A case of congenital Borrelia duttoni relapsing fever in Rwanda. Annales de la Societe Belge de Medecine Tropicale. 1983;63(4):367-9.
1542. Goubau PF, Munyangeyo C. Tick-borne relapsing fever and pregnancy. A clinical study in Rwanda. [French]. Annales de la Societe belge de medecine tropicale. 1983;63(4):347-55.
1543. Steenbarger JR. Congenital tick-borne relapsing fever: Report of a case with first documentation of transplacental transmission. Birth Defects: Original Article Series. 1982;18(3 A):39-45.
1544. Southern PMJ. RELAPSING FEVER1982. P953-62 p.
1545. Olchovsky D, Pines A, Sadeh M. Multifocal neuropathy and vocal cord paralysis in relapsing fever. European Neurology. 1982;21(5):340-2.
1546. Rodhain F. BORRELIA AND RELAPSING FEVERS - TODAY EPIDEMIOLOGICAL FEATURES. Bulletin De L Institut Pasteur. 1976;74(2):173-218.
1547. Katznelson D, Brook U, Gross S. Relapsing fever in the area of Kefar-Saba. Harefuah. 1976;90(5):231.
1548. de Clercq AG, Meheus AZ, de Pierpont E, Nyirashema C. Single-dose doxycycline treatment of tick-borne relapsing fever. East Afr Med J. 1975;52(8):428-9.
1549. Felsenfeld O. The problem of relapsing fever in the Americas. IMS, Industrial medicine and surgery. 1973;42(3):7-10.
1550. Sezi CL. Relapsing fever at Masaka Hospital. East African medical journal. 1970;47(3):176-8.

1551. Teesdale C. Tick-borne relapsing fever. The present position in Kenya. East African medical journal. 1965;42(10):529-34.
1552. Reynaud R, Massat R, Picca M, Revil H. APROPOS OF 12 CASES OF TICK-BORNE RELAPSING FEVER. Bulletin de la Société médicale d'Afrique noire de langue française. 1965;10:1-4.
1553. Neuman S, Schick G. OBSERVATIONS ON RELAPSING FEVER. Harefuah. 1964;66:402-4.
1554. Klein M. Relapsing Fever—Successful Treatment with Demethylchlortetracycline (Declomycin). Calif Med. 1964;100(4):283-5.
1555. Gefel A, Anzarut A, Pruzanski W. CLINICAL PICTURE AND THERAPY OF TICK-BORNE RELAPSING FEVER; SUMMARY OF 49 CASES. Israel medical journal. 1964;23:211-9.
1556. Dutta RN. A STUDY ON INDIAN TICK-BORNE RELAPSING FEVER. The Journal of the Association of Physicians of India. 1964;12:341-5.
1557. Correa P, Baylet RJ, Bourgoin P. APROPOS OF A CASE OF RECURRENT FEVER DUE TO BORELIA DUTTONI IN A NEWBORN WHERE TRANSPLACENTAL INFECTION APPEARS INCONTESTABLE. Bulletin de la Société médicale d'Afrique noire de langue française. 1964;18:215-7.
1558. Mooser H. The relapsing fever. Ergebnisse der Mikrobiologie, Immunitätsforschung und experimentellen Therapie. 1958;31:184-228.
1559. Mooser H. Erythrocyte adhesion and hemagglomeration by relapsing fever spirochetes. Zeitschrift für Tropenmedizin und Parasitologie. 1958;9(2):93-111.
1560. Fen CT. ON THE STUDY OF THE VECTORS OF TICK RECURRENT FEVER IN CHINA. Doklady Akademii Nauk Sssr. 1958;121(4):766-8.
1561. Sergent E. Coexistence of relapsing fever & paludism in the same patient. Zeitschrift für Tropenmedizin und Parasitologie. 1957;8(1-2):242-5.
1562. Diaz Ferron E. Recurrent fever and tetracyn. Revista clínica española. 1957;64(2):97-101.
1563. Gimeno De Sande A. Campaign with gammexane for prevention of the Hispano-African relapsing fever. Revista de sanidad e higiene pública. 1956;30(1-2):102-17.
1564. Ercoli N, Bobalik G, Stubbs R. Antibiotic resistance of relapsing fever spirochetes. Antibiotics annual. 1956:601-15.
1565. Martini E. History of relapsing fever in Europe. Ergebnisse der Hygiene, Bakteriologie, Immunitätsforschung und experimentellen Therapie. 1955;29:213-47.
1566. Baltazard M, Bahmanyar M, Chamsa M. Relapsing fever in Afghanistan. Bulletin de la Société de pathologie exotique et de ses filiales. 1955;48(2):159-61.
1567. Juarez E, Fernandez E. Treatment of relapsing fever in Spain by aureomycin. Revista de sanidad e higiene pública. 1954;28(3-4):133-59.
1568. Jaurez E. Relapsing fever in Spain and its treatment with terramycin. Revista de sanidad e higiene pública. 1954;28(5-6):356-61.
1569. Gimeno De Sandre A. Aureomycin in relapsing fever in Spain. Revista de sanidad e higiene pública. 1954;28(5-6):342-55.
1570. Davis GE, Burgdorfer W. On the susceptibility of the guinea pig to the relapsing fever spirochete Borrelia duttonii. Bulletin de la Société de pathologie exotique et de ses filiales. 1954;47(4):498-501.
1571. Burgdorfer W. On the occult infection in relapsing fevers. Bulletin de la Société de pathologie exotique et de ses filiales. 1954;47(5):664-7.
1572. Baltazard M, Pournaki R, Chabaud AG. Relapsing fevers caused by Ornithodoros. Bulletin de la Société de pathologie exotique et de ses filiales. 1954;47(4):589-97.

1573. Baltazard M, Bahmanyar M, Chamsa M. Use of the guinea pig in the differentiation of relapsing fever spirocheta; the individuality of the Borrelia crocidurae group. Bulletin de la Société de pathologie exotique et de ses filiales. 1954;47(6):864-77.
1574. Utrera Rivas R. Relapsing fever and aureomycin. Medicamenta. 1953;11(233):255-6.
1575. Mac Keith R. [Recurrent fever in children]. Arch Fr Pediatr. 1953;10(2):176-8.
1576. Gefel A, Rubenow R. Terramycin in the treatment of relapsing fever. Harefuah. 1953;44(12):263-6.
1577. Fischer L. The problem of relapsing fever in Afghanistan. Zeitschrift für Tropenmedizin und Parasitologie. 1953;4(3):339-43.
1578. Bertrand A, Leonard C. Two cases of relapsing fever. L'unión médicale du Canada. 1953;82(11):1227-32.
1579. Theodoulou M. Tick-borne relapsing fever in Cyprus. Cyprus medical journal. 1952;5(8-9):860-2.
1580. Parhami Y. 200 Cases of relapsing fever in children. Archives françaises de pédiatrie. 1952;9(8):844-7.
1581. Pages R. Ocular complications in relapsing fever. La semaine des hôpitaux : organe fondé par l'Association. 1952;28(94):3824-7.
1582. Dubois A. Arsenical treatment of tick-borne relapsing fever. Annales de la Société belge de médecine tropicale. 1952;32(1):35-9.
1583. Colas-Belcour J, Neel R, Vervent G. Transmission of the spirochete of Madagascar relapsing fever (Borrelia duttoni) by the Ornithodoros moubata of Madagascar. Bulletin de la Société de pathologie exotique et de ses filiales. 1952;45(1):69-78.
1584. Bertazzi CG. Treatment of African relapsing fever. Acta medica Italica di malattie infettive e parassitarie. 1952;7(3):57-62.
1585. Trowell HC. The treatment of tick-borne relapsing fever in East Africa with special reference to aureomycin. East African medical journal. 1951;28(10):402-12.
1586. Manson-Bahr P. Antibiotics in the treatment of tropical disease. II. Relapsing fevers. Medical world. 1951;74(17):490-1; passim.
1587. Kalra SL, Rao KN. Observations on the epidemiology of relapsing fever in Kashmir. The Indian journal of medical research. 1951;39(3):313-21.
1588. Innerfield I. Aureomycin in relapsing fever. New York state journal of medicine. 1951;51(8):1057-8.
1589. Harrison IB, Whittington RM. Antibiotics in the treatment of relapsing fever. United States Armed Forces medical journal. 1951;2(12):1859-62.
1590. Coghill NF. Treatment of tick-borne relapsing fever. Neoarsphenamine and other remedies. Lancet. 1951;1(11):604-5.
1591. Yeo RM. Tick-borne relapsing fever on the Witwatersrand gold mines; its treatment with aureomycin. South African medical journal = Suid-Afrikaanse tydskrif vir geneeskunde. 1950;24(24):457-9.
1592. Senecal J, Ahmad A. Treatment of recurrent fever with penicillin. La semaine des hôpitaux : organe fondé par l'Association d'enseignement médical des hôpitaux de Paris. 1950;26(35):1634-8.
1593. Ravina A, Pecher Y, Avril J. Relapsing fever contracted in Paris. Bulletins et mémoires de la Société médicale des hôpitaux de Paris. 1950;66:508-10.
1594. Mazzotti L. Aureomycin the treatment of human relapsing fever caused by Spirochaeta turicatae. Revista del Instituto de Salubridad y Enfermedades Tropicales. 1950;11(1):53-5.

1595. Legerton CW, Chambers WL. Spontaneous rupture of the spleen in relapsing fever. United States Armed Forces medical journal. 1950;1(1):88-90.
1596. Aruwa WA. A case of relapsing fever in a child of nine days old. East African medical journal. 1950;27(4):176-7.
1597. Coghill NF. Clinical manifestations of tick-borne relapsing fever with special. Journal of the Royal Army Medical Corps. 1949;93(1):2-33.
1598. Viennot B. [Penicillin and recurrent fever]. Maroc Med. 1948;27(272):25.
1599. Shaul JF, Saferstein TH. Penicillin therapy in relapsing fever; report of four cases. United States naval medical bulletin. 1947;47(2):238-43.
1600. Baltazard, Bahmanyar, Mofidi C. [Recurrent fevers transmitted by both ornithodores and lice]. Ann Inst Pasteur (Paris). 1947;73(11):1066-71.
1601. Young WA, Farr AG, McKendrick AJ. Relapsing fever in the Lake Province of Tanganyika with an account of a case in an eight day old infant. East African medical journal. 1946;23(11):345-7.
1602. Fischer I. Penicillin therapy in relapsing fever. The American journal of tropical medicine and hygiene. 1946;26:483-8.
1603. Sarrouy C, Berthault F, Groscolas R. [Recurrent fever in a newborn baby of probably congenital origin]. Bull Mem Soc Med Hop Paris. 1945;61(26-31):356.
1604. Wheeler CM, Herms WB, Meyer KF. A new tick vector of relapsing fever in California. Proceedings of the Society for Experimental Biology and Medicine. 1935;32(8):1290-2.
1605. Todd J. TREATMENT OF RELAPSING FEVER. Br Med J. 1930;1(3606):312.
