# Supplementary material for: Tick borne relapsing fever - a systematic review and analysis of the literature
Source: PLoS Negl Trop Dis. 2022 Feb 16;16(2):e0010212. doi: 10.1371/journal.pntd.0010212 (PMC8887751; doi:10.1371/journal.pntd.0010212)
Supplement: S1 Table — Used for screening and selecting eligible publications. (PDF) [file pntd.0010212.s004.pdf]

Tick borne relapsing fever – a systematic review and analysis of the literature

S1 Table

Data extraction sheet.

Screening and Selecting Tool – Data extraction Sheet			
**Reviewer name:**			**Date:**
**Author name/study ID/country:**			**Year:**
**Title:**			**Journal:**
**Studies excluded because:**			
Not about TBRF (other disease)	Language	No relevant information (epidemiological, clinical or diagnostical information)	other

	**Case report:**
	**Case series:**
	**Cohort study**:
	**Randomized controlled trial**:
	**Epidemiological study**:
	**Non-randomized controlled trial**:
	**Other**:
	**Study period:**
	**Number of diagnosed patients for TBRF**:
	**Gender**:
	**Median age**:
	**Age range**:
	**Country of diagnosis**:
	**Country of manifestation**:
	**Laboratory confirmed A (molecular)**:
	**Laboratory confirmed B (microscopy)**:
	**Laboratory suspected B (culture)**:
	**Laboratory suspected B (animal inoculation)**:
	**Laboratory suspected C (serology)**:
	**Vectors:**
	**Tick species:**
	***Borrelia*:**
	**Fever relapses in absolute number (n)**:
	**Ticks infected with *Borrelia* (%):**
	**Vectors infested with ticks (%):**
	**Major laboratory abnormalities**:
	**Name of Hospital where treated:**
	**Number of patients treated**:
	**Number of patients untreated**:
	**Treatment regimens (drugs, dosage, duration)**:

	**Therapy outcome**:
	**Frequency of JHR (absolute number)**:
	**Frequency of JHR (%)**:
	**Complications**:
	**Concomitant pregnancy:**
	**Mortality treated (absolute number)**:
	**Mortality treated (%)**:
	**Mortality untreated (absolute number)**:
	**Mortality untreated (%)**:
	**Cause of death**:
	**Co-infections**:
	**Interesting points/quotes/conclusions**:
	**Comments/Other**:

JHR, Jarisch-Herxheimer reaction; TBRF, tick borne relapsing fever.
