# Supplementary material for: Tick borne relapsing fever - a systematic review and analysis of the literature
Source: PLoS Negl Trop Dis. 2022 Feb 16;16(2):e0010212. doi: 10.1371/journal.pntd.0010212 (PMC8887751; doi:10.1371/journal.pntd.0010212)
Supplement: S2 Table — Treatment details: antimicrobial treatment regimen, dosage and duration. (PDF) [file pntd.0010212.s005.pdf]

Tick borne relapsing fever – a systematic review and analysis of the literature

S2 Table

TBRF treatment details: antimicrobial treatment regimen, dosage and duration.

Antimicrobial treatment regimen	Reported in number of studies (n)	Daily dosage	Duration (days)
**Doxycycline**	85	100-400 mg i.v./p.o.	5-21
**Penicillin**	41	1,000-3,000 mg p.o.;<br>300,000-4,000,000 U i.m.;<br>300,000-18,000,000 U i.v.	5-14
**Tetracycline**	37	500-2,000 mg i.v./p.o.	1-15
**Ceftriaxone**	28	1,000-2,000 mg i.v.	5-30
**Erythromycin**	16	750-2,000 mg i.v./p.o.	5-14
**Arsenicals**	12	unclear	unclear
**Ciprofloxacin**	5	unclear	unclear
**Azithromycin**	3	unclear	unclear
**Cefuroxime**	2	unclear	unclear
**Gentamicin and penicillin**	2	unclear	unclear
**Levofloxacin**	2	unclear	unclear
**Minocycline**	2	unclear	unclear
**Bismuth iodide**	1	unclear	unclear
**Cefotaxime**	1	unclear	unclear
**Ceftizoxime**	1	unclear	unclear
**Ertapenem**	1	unclear	unclear
**Gatifloxacin**	1	unclear	unclear
**Metronidazole**	1	unclear	unclear
**Piperacillin/tazobactam**	1	unclear	unclear
**Streptomycin**	1	unclear	unclear
**Tobramycin**	1	unclear	unclear
**Vancomycin**	1	unclear	unclear

i.v., intravenous; p.o., per os; i.m., intramuscular.
