# Supplementary material for: Tick borne relapsing fever - a systematic review and analysis of the literature
Source: PLoS Negl Trop Dis. 2022 Feb 16;16(2):e0010212. doi: 10.1371/journal.pntd.0010212 (PMC8887751; doi:10.1371/journal.pntd.0010212)
Supplement: S1 Fig — Distribution of competent vector ticks for TBRF Borrelia spp. (PDF) [file pntd.0010212.s009.pdf]

Tick borne relapsing fever – a systematic review and analysis of the literature

S1 Fig

Supporting maps: distribution of competent vector ticks for TBRF *Borrelia* spp.

S1A Fig Distribution of competent vector ticks for tick borne relapsing fever (TBRF) *Borrelia* in America.

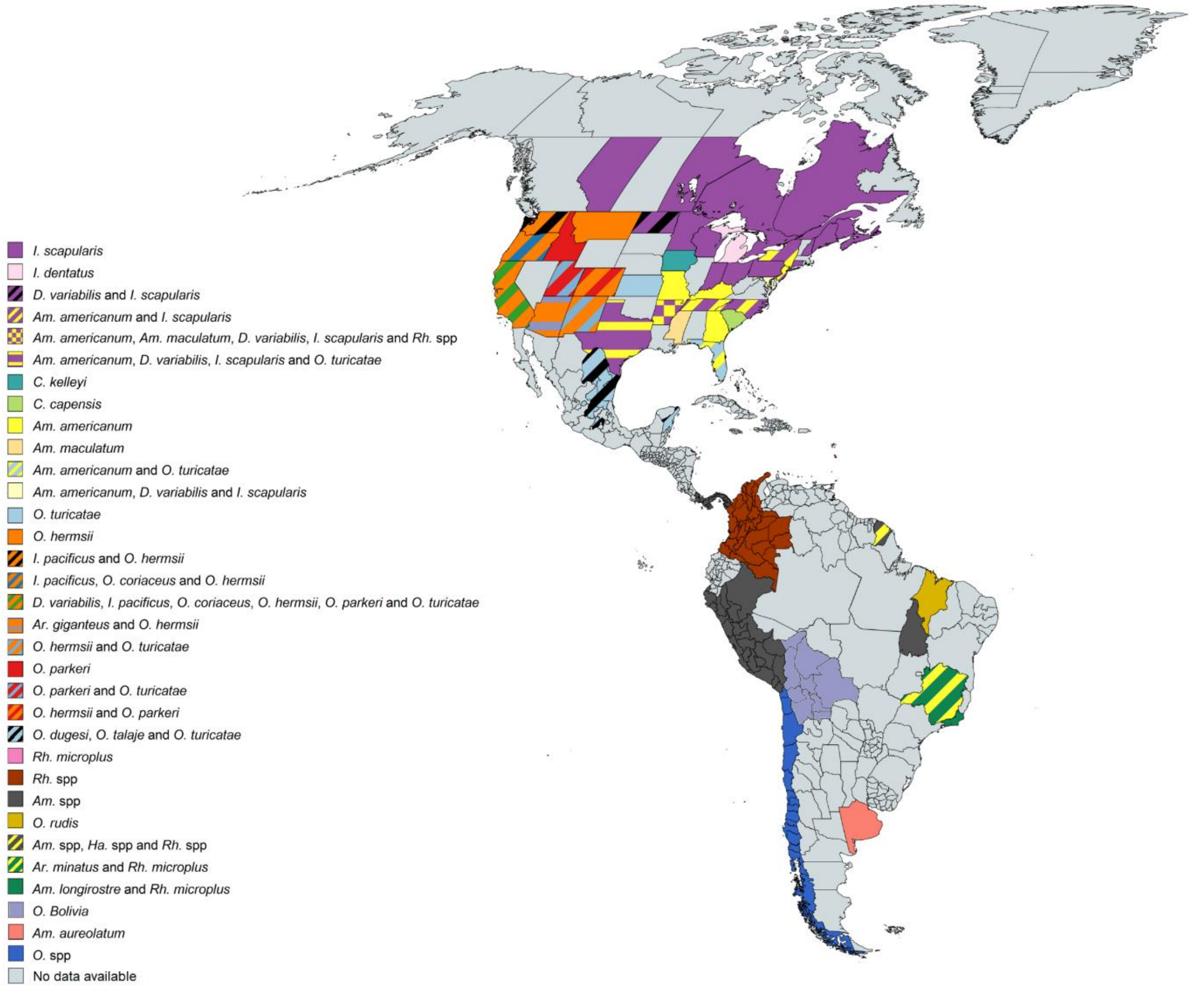

Am., *Amblyomma*; Ar., *Argas*; C., *Carios*; D., *Dermacentor*; Ha, *Haemaphysialis*; I., *Ixodes*; O., *Ornithodoros*; Rh., *Rhipicephalus*; sp., species (singular); spp., species (plural).

Map created on www.mapchart.net.

S1B Fig Distribution of competent vector ticks for TBRF *Borrelia* in Africa.

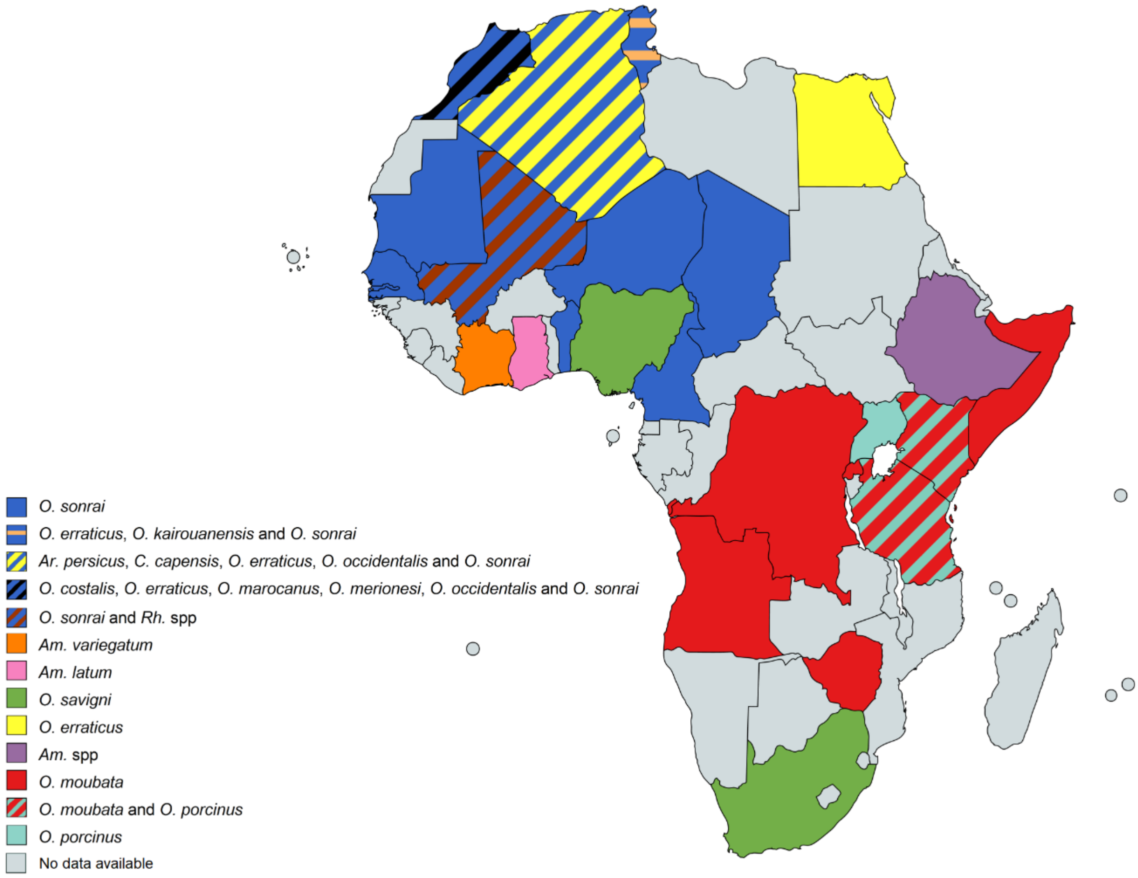

Am., *Amblyomma*; Ar., *Argas*; C., *Carios*; O., *Ornithodoros*; Rh. *Rhipicephalus*; spp., species (plural).

Map created on www.mapchart.net.

S1C Fig Distribution of competent vector ticks for TBRF *Borrelia* in Europe.

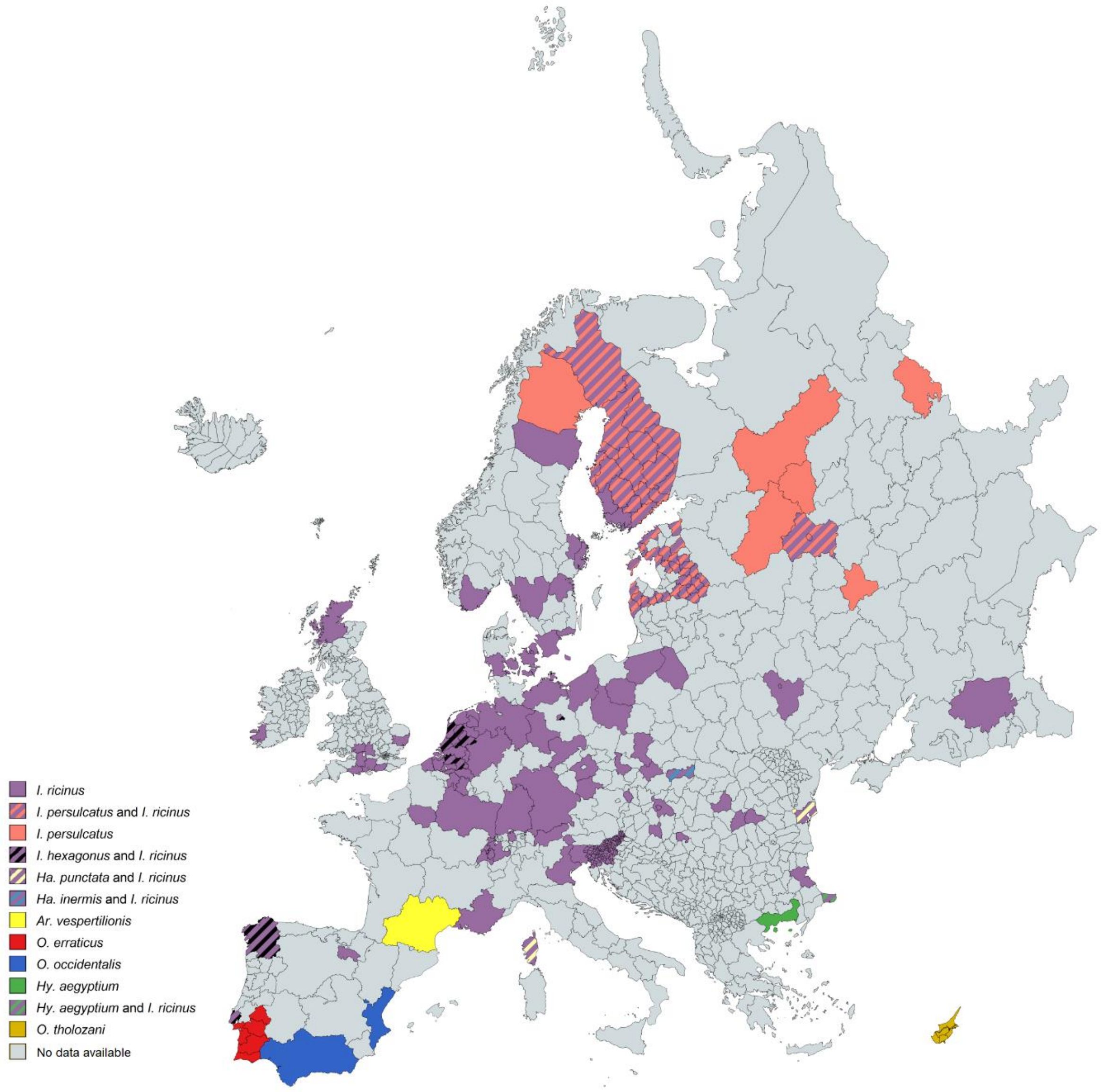

Ar., *Argas*; Ha., *Haemaphysalis*; Hy., *Hyalomma*; I., *Ixodes*; O., *Ornithodoros*.

Map created on www.mapchart.net.

S1D Fig **Distribution of competent vector ticks for TBRF *Borrelia* in Asia.**

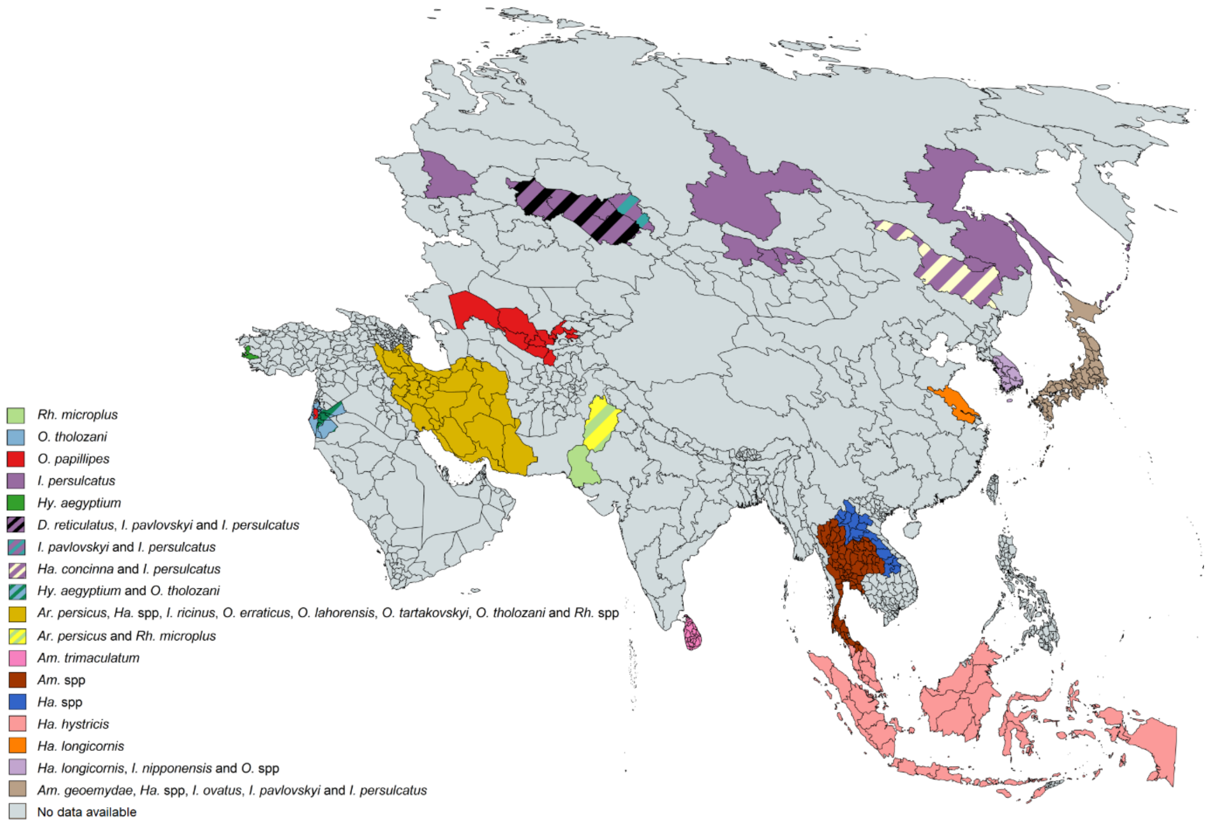

Am., *Amblyomma*; Ar., *Argas*; D., *Dermacentor*; Ha., *Haemaphysalis*; Hy., *Hyalomma*; I., *Ixodes*; O., *Ornithodoros*; Rh., *Rhipicephalus*; spp., species (plural).

Map created on www.mapchart.net.
