# Supplementary material for: Tick borne relapsing fever - a systematic review and analysis of the literature
Source: PLoS Negl Trop Dis. 2022 Feb 16;16(2):e0010212. doi: 10.1371/journal.pntd.0010212 (PMC8887751; doi:10.1371/journal.pntd.0010212)
Supplement: S2 Fig — Subgroup analysis of B. crocidurae and B. hermsii. (PDF) [file pntd.0010212.s010.pdf]

Tick borne relapsing fever – a systematic review and analysis of the literature

S2 Fig

Subgroup analysis of *B. crocidurae* and *B. hermsii*.

S2A Fig **Reported number of relapsing fever episodes in studies on TBRF caused by *B. crocidurae* or *B. hermsii*.**

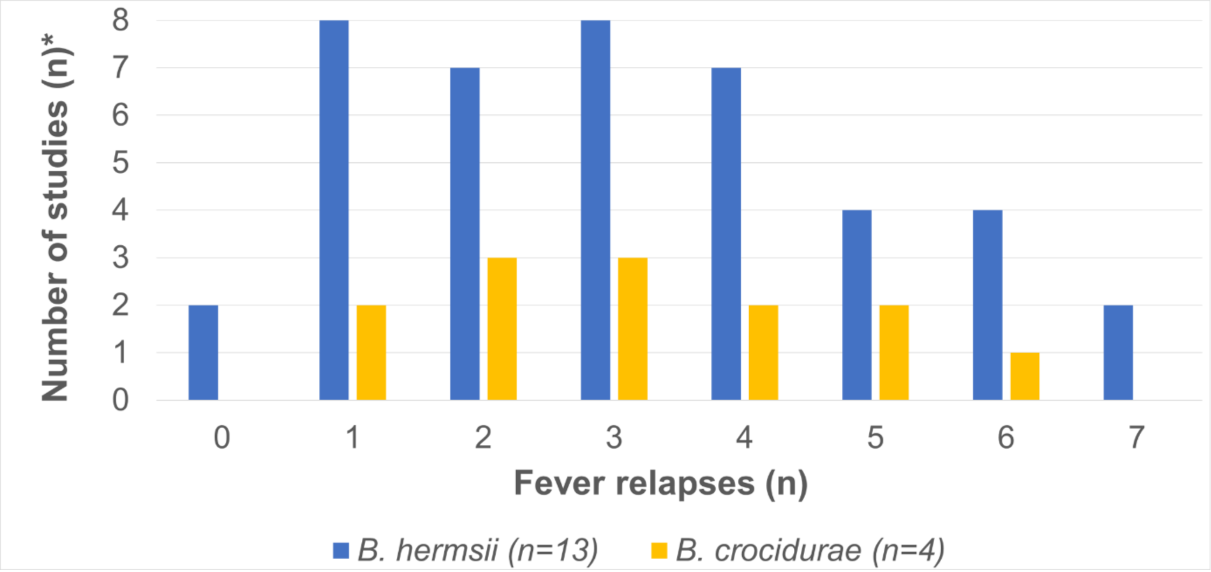

S2B Fig **Reported relative frequency of signs and symptoms (in %) related to *B. crocidurae* and *B. hermsii* TBRF infections.**

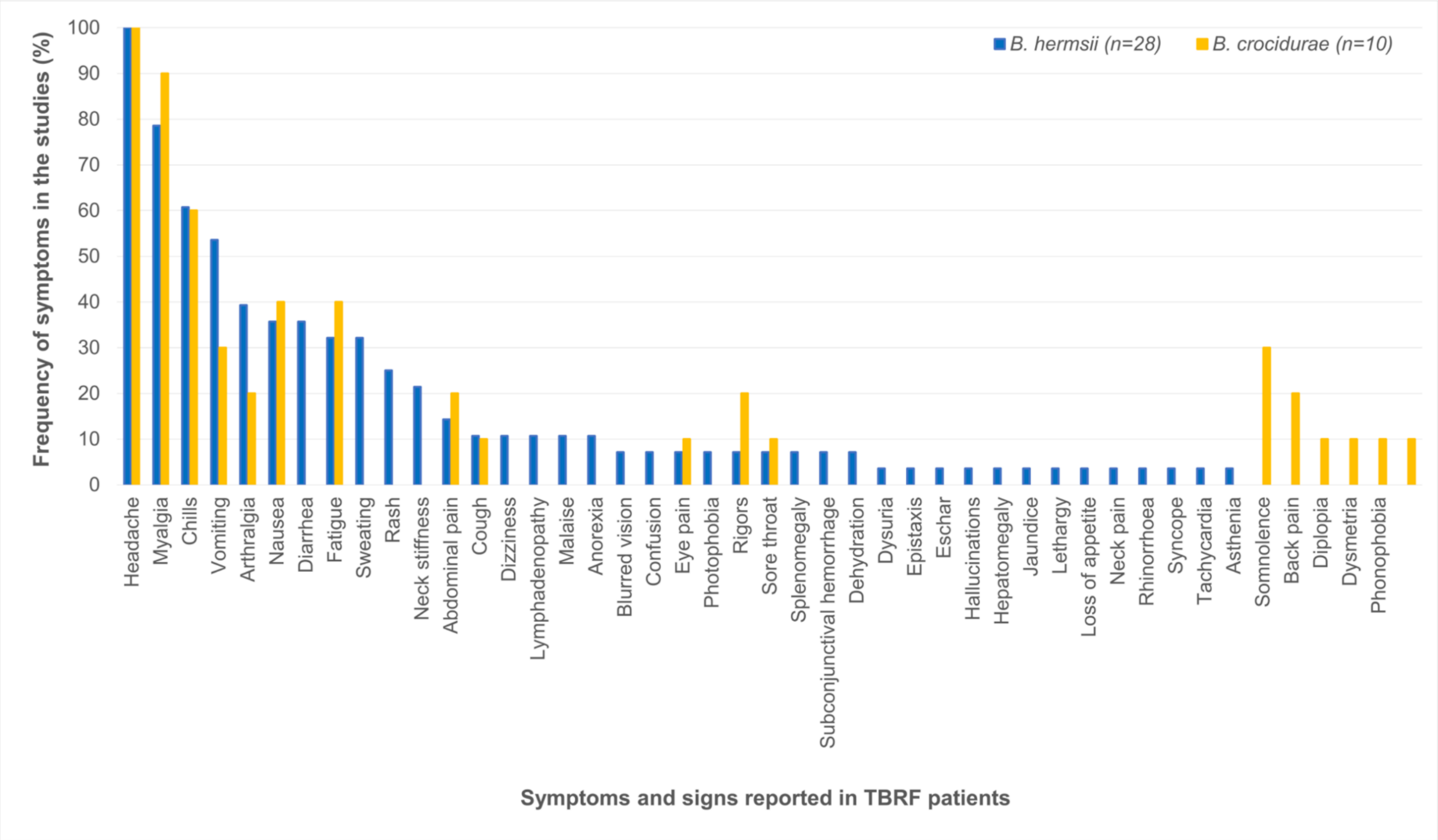

S2C Fig **Complications (in %) related to TBRF cases caused by *B. crocidurae* or *B. hermsii*, as reported by the authors.**

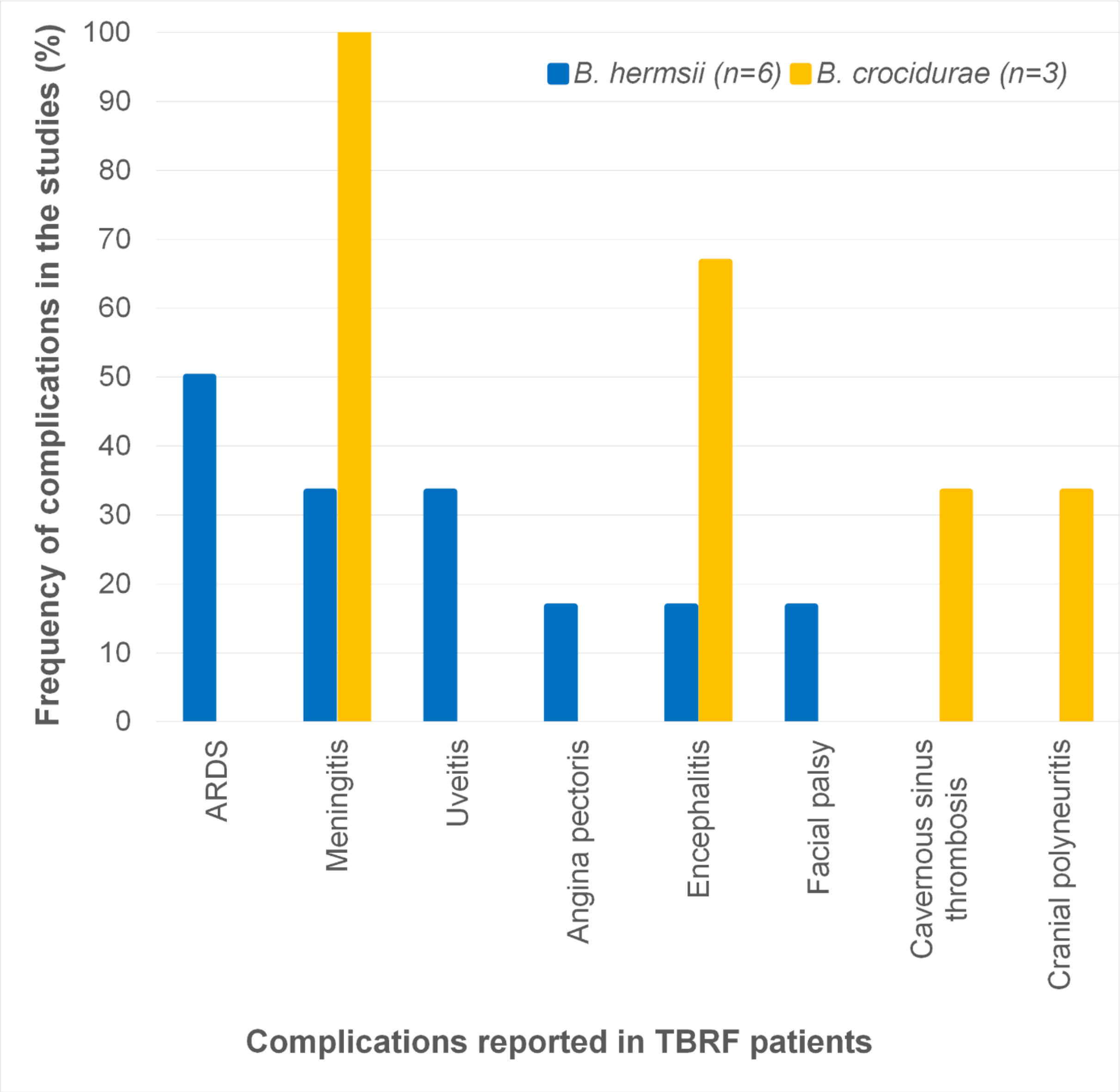

ARDS, acute respiratory distress syndrome.
